# Epistatic determinism of durum wheat resistance to the wheat spindle streak mosaic virus

**DOI:** 10.1007/s00122-017-2904-6

**Published:** 2017-04-27

**Authors:** Yan Holtz, Michel Bonnefoy, Véronique Viader, Morgane Ardisson, Nicolas O. Rode, Gérard Poux, Pierre Roumet, Véronique Marie-Jeanne, Vincent Ranwez, Sylvain Santoni, David Gouache, Jacques L. David

**Affiliations:** 1 Montpellier SupAgro, UMR AGAP, 34060 Montpellier, France; 2 INRA, UMR AGAP, 34060 Montpellier, France; 3 Montpellier SupAgro, UMR BGPI, 34398 Montpellier Cedex 5, France; 4 Arvalis, Institut du Végétal, Paris, France

## Abstract

**Key message:**

**The resistance of durum wheat to the Wheat spindle streak mosaic virus (WSSMV) is controlled by two main QTLs on chromosomes 7A and 7B**, **with a huge epistatic effect.**

**Abstract:**

*Wheat spindle streak mosaic virus* (WSSMV) is a major disease of durum wheat in Europe and North America. Breeding WSSMV-resistant cultivars is currently the only way to control the virus since no treatment is available. This paper reports studies of the inheritance of WSSMV resistance using two related durum wheat populations obtained by crossing two elite cultivars with a WSSMV-resistant emmer cultivar. In 2012 and 2015, 354 recombinant inbred lines (RIL) were phenotyped using visual notations, ELISA and qPCR and genotyped using locus targeted capture and sequencing. This allowed us to build a consensus genetic map of 8568 markers and identify three chromosomal regions involved in WSSMV resistance. Two major regions (located on chromosomes 7A and 7B) jointly explain, on the basis of epistatic interactions, up to 43% of the phenotypic variation. Flanking sequences of our genetic markers are provided to facilitate future marker-assisted selection of WSSMV-resistant cultivars.

**Electronic supplementary material:**

The online version of this article (doi:10.1007/s00122-017-2904-6) contains supplementary material, which is available to authorized users.

## Introduction

*Wheat spindle streak mosaic virus* (WSSMV) infects bread and durum wheats in Europe and North America (Ordon et al. 2009). WSSMV belongs to the *Bymovirus* genus in the *Potyviridae* family (Barnett 1991; Hariri et al. 1996; Xiaoyun et al. 1988). Symptoms of WSSMV include yellow-striped mosaic patterns on leaves and stunted spring growth, resulting in extensive damage and yield loss (Miller et al. 1992). WSSMV is transmitted by *Polymyxa graminis* (Adams 1990), a Plasmodiophoridae (Schwelm et al. 2015) that is soil-borne. *P. graminis* is an obligate parasite of wheat and other Poaceae roots. It produces resting spores that inhabit the soil and can host the virus for 10 years or more (Kanyuka et al. 2003; Driskel et al. 2004). WSSMV infection starts in the roots, and then progresses to the aerial parts of the plant including the leaves (Carroll et al. 1997). Mosaic symptoms are mostly visible in leaves and characterized by cytoplasmic inclusions in infected cells (Sohn et al. 1995).

*Polymyxa graminis* is the vector of all bymoviruses, including *Wheat yellow mosaic virus* (WYMV), which is mainly reported in Asia (Liu et al. 2016). These two bymoviruses (WSSMV and WYMV) exhibit about 70% identity at the nucleotidic level of their capsid genes (Namba et al. 1998; Xiaoyun et al. 1988; Clover and Henry 1999; Liu et al. 2016). *Polymyxa graminis* also transmits some furoviruses to wheat, such as *Soil*-*borne cereal mosaic virus* (SBCMV) and *Soil*-*borne wheat mosaic virus* (SBWMV) (Kanyuka et al. 2003). These viruses have similar life cycles as well as structural similarities in the viral proteins involved in their transmission by *P. graminis* (Adams et al. 2001). WSSMV and SBCMV co-infections of wheat often occur.

Since breeding resistant cultivars is currently the only way to control the disease (Kanyuka et al. 2003), it is crucial to find resistant progenitors and understand the genetic determinism of their resistance. A major QTL was detected in bread wheat on chromosome 2DL for resistance to both WSSMV (Khan et al. 2000) and WYMV (Kojima et al. 2015). This source of resistance is not easily exploitable in durum wheat breeding due to its location on the D genome. As far as we know, genetic control of WSSMV resistance in durum wheat has never been reported and Soldur is the only durum wheat cultivar reported to be resistant. Soldur is, therefore, intensively used by breeders and identifying new sources of resistance could help develop sustainable WSSMV control. The genetic control of the resistance to WSSMV in Soldur has yet to be published.

This lack of diversity in sources of resistance to WSSMV among durum wheat cultivars is probably due to the severe bottleneck that durum wheat experienced over the past millennia (Thuillet et al. 2005; Haudry et al. 2007). We thus broadened the search for WSSMV resistance by screening the diversity of wild (*T. turgidum* ssp. *dicoccoides*) and cultivated taxa (*T. t. dicoccum*, *T. t. polonicum*, *T. t. carthlicum*, *T. t. turgidum*, and *T. t. durum* from different breeding programs made to broaden the genetic basis). Between 2006 and 2008, we screened 226 accessions and identified very few accessions exhibiting stable WSSMV resistance among years. One of the best accessions, hereafter named Dic2, was used as a source of resistance for this study.

As routine screening of numerous lines through WSSMV inoculation are not conducted in glasshouses or growth chambers, field experiments are essential to score the genetic material. Visual scoring is an efficient phenotyping method, but WSSMV infected fields are frequently infected by other related viruses, such as SBCMV, which may lead to some confusion (Carroll et al. 1997; Vallega et al. 2003). Thus, the use of more discriminant viral content assessment methods such as DAS-ELISA (double antibody sandwich-enzyme-linked immunosorbent assay) based on specific serum (Clark and Adams 1977), and PCR, based on specific primers (Vaïanopoulos et al. 2006) is necessary. Although ELISA and PCR are more precise, they are more prone to sampling bias when the viral infection is heterogeneous within the plot. The spatial heterogeneity of virus distribution in the field may, during sensitivity assessments, lead to major variations amongst repeats at the same site, since some sensitive genotypes have erroneously been recorded as being resistant when observed at sites where soil inoculum is weak or absent (Cadle-Davidson et al. 2006). Climate conditions greatly influence symptom severity at a given site, which could range from no apparent symptoms to heavy damage on the plants (Cadle-Davidson et al. 2006). Breeding for resistance to WSSMV based on phenotypic analysis is thus quite costly and time-consuming. Marker-assisted selection (MAS) seems to be a promising approach for the pre-breeding of virus-resistant genitors from exotic germplasm.

In this study, we investigated the genetic basis of WSSMV resistance of Dic2 via two RIL populations obtained by crossing the elite cultivars Silur and Lloyd with Dic2. RILs were phenotyped in 2012 and 2015, then genotyped using targeted locus capture (Holtz et al. 2016). We accounted for the spatial heterogeneity of symptoms to improve RIL BLUP (best linear unbiased predictor) estimates. A consensus genetic map and a joint-QTL analysis led to the identification and localization of major QTLs with stable resistance to WSSMV.

## Materials and methods

### Plant material and field trials

Two F_6_ RIL populations were used in this study, both derived from a cross between a cultivated emmer wheat (*Triticum turgidum* ssp *dicoccum*) named Dic2 and a durum elite cultivar (either Silur or Lloyd). Dic2 was introduced into our collection at the end of the 1980s. Its origin was uncertain, although its SSR profile is close to the accession Tri 2215, available in the IPK (Institut für Pflanzgenetik und Kulturpflanzenforschung) collection and originated from Spain (https://gbis.ipk-gatersleben.de/GBIS_I/). The Dic2 × Silur (DS) and Dic2 × Lloyd (DL) mapping populations consisted of 165 and 189 RILs, respectively. Dic2 has been classified as resistant to WSSMV based on observations in field experiments conducted in multiple locations and years. In contrast, Silur and Lloyd are highly susceptible cultivars. All trials were carried out in Pray, France, (47°40′27″N 1°07′58″E) in a field with high WSSMV prevalence. The field showed regular WSSMV infection and the absence of SBCMV was controlled by the absence of visual symptoms on the bread wheat cultivar Aztec, known to be resistant to WSSMV and susceptible to SBCMV (M. Bonnefoy, pers. com).

The two RIL populations were observed in 2012 and 2015. The corresponding experiments are hereafter called DS-2012, DL-2012, DS-2015 and DL-2015 (Table 1). Genotypes were grown according to a randomized complete block design with 10 lines and 75 columns in 2012 and 17 lines and 41 columns in 2015. For each genotype, about 30 plants were grown in an observation unit consisting of two adjacent 1-m long manually-seeded rows. Several genotypes were repeated twice for DS-2012, DS-2015 and DL-2015 (81, 54 and 54 repeated genotypes, respectively), but no repetition was available for DL-2012. Pescadou—a highly susceptible check— was regularly included in the trials to control spatial heterogeneity in the distribution using 149 phenotyped plants in 2012 and 100 phenotyped plants in 2015. The three parents were also randomly included in the trial (Dic2: 1 and 2 samples, Silur: 4 and 2 samples, Lloyd: 5 and 3 samples in 2012 and 2015, respectively). Experimental fields were sown in early October and managed with agronomic practices commonly implemented in the area. In 2013, DS and DL RILs were also sown at the DIASCOPE experimental station (Mauguio, Southern France, 43°36′55″N 4°0′36″E) to monitor plant heights and flowering dates.

**Table 1:** Population name, number of recombinant inbred lines (RILs), number of repeated genotypes and phenotyping method used in the four experiments

Exeriments^a^	DS-2012	DL-2012	DS-2015	DL-2015
No. of RILs	165	187	164	189
No. of duplicated RILs	81	0	54	54
Total no. of plants	246	187	218	243
Visual assessment of symptom severity (SS)	Yes	Yes	Yes	Yes
ELISA	Yes	Yes	Yes	Yes
qPCR	No	No	Yes	Yes

^a^DS-2012, DL-2012, DS-2015, and DL-2015 represent the two RIL populations DS (Dic2 × Silur) and DL (Dic2 × Lloyd) evaluated in 2012 and 2015, respectively

### Genotyping

Two RIL (F_6_) populations were genotyped in 2015 and used for QTL detection based on the 2012 and 2015 data. We used locus targeted genotyping according to the protocol of Holtz et al. (2016). Briefly, two sets of 120 bp baits were designed to target 6240 and 10,027 SNPs previously detected in the RNAseq data of Dic2, Lloyd and Silur genitors. RIL DNA was extracted in 2015 and captured according to Rohland and Reich (2012). Compared to Holtz et al. (2016), blocking oligos were added to limit the capture of microsatellite-like sequences (Online Resource 1). Captured DNA was sequenced with two runs of HiSeq 3000 (150 bp paired end reads). Reads were cleaned and mapped on a durum wheat reference transcriptome (DWr) (Ranwez et al. 2013; David et al. 2014) using cutAdapt (Martin 2011) and bwa-mem (Li and Durbin 2009).

Putative chromosomal assignment and physical positions of the DWr contigs were obtained by blasting them on the bread wheat chromosome survey sequence for cv. Chinese Spring (BWr) generated by IWGSC (Ensembl database release 28), (Mayer et al. 2014; Chapman et al. 2015). Genotypes were called using *Reads2snp* (Galtier et al. 2009) and SNPs were filtered according to the following criteria: (1) inbreeding coefficients, *Fis*, above 0.8 corresponding to a low probability of being heterozygotic, as 1.5% heterozygosity is expected on average after six successive selfing generations, (2) at least 100 RILs genotyped for any given SNP, and (3) balanced frequencies with a minimum expected heterozygosity (Nei 1978) of 0.34 so as to avoid strong segregation distortion, which is undesirable for genetic map building.

Four SSR markers known to be on the distal part of chromosome 7B were also used to genotype the DS RILs: *Xbarc1068*, *Xbarc323*, *Xgwm400* and *Xgwm46* (http://wheat.pw.usda.gov/). These four markers are linked to SBCMV resistance (Maccaferri et al. 2011). They were not used for the linkage map construction, but were positioned on it afterward. SNPs showing the highest linkage disequilibrium with those SSR markers were used to position the SSRs on our consensus genetic map. This allowed us to test whether or not WSSMV and SBCMV resistance genes were collocated.

### Linkage map construction

SNPs from the DS population (DS-SNPs) and the DL population (DL-SNPs) were used to build two individual maps (DS map and DL-map). The DS map construction was described in (Holtz et al. 2016), with a focus on the capture technology. The DL-map was constructed using the same procedure that is briefly described hereafter. Carthagene (de Givry et al. 2005) was used to assemble initial linkage groups (LGs) using LOD score thresholds of 7 and 8 for DS and DL, respectively, and maximum two-point distances of 0.14 and 0.1 for DS and DL, respectively. As the SNPs were already assigned to a BWr chromosome [best blast procedure (Holtz et al. 2016)], each LG was then assigned to the most frequent carrier chromosome of its SNPs. Markers on LGs assigned to the same chromosome were pooled. Orders and distances between adjacent markers within chromosomes were finally determined using the *build*, *annealing*, *greedy* and *flips* algorithms implemented in Carthagene.

Then common markers between the DS- and DL-maps were used to build a consensus map containing all the markers using the Carthagene *dsmergen* function. Genetic maps were characterized and compared using the genetic map comparator web application (Holtz et al. 2017). Once the marker positions were set, missing data were attributed using the CallParentAllelesPlugin of Tassel (Glaubitz et al. 2014). Finally, we estimated the number of recombination events accumulated during the fixation of each RIL by counting the number of switches between stretches with successive parental allelic status.

### Phenotyping WSSMV resistance

In 2012 and 2015, symptom severity (SS) was visually scored twice during periods when disease symptoms were of maximum intensity. The first notation (SS1) was performed on April 18th in 2012 and on March 27th in 2015, during the Z32 stage of Zadoks’ growth scale (Zadoks et al. 1974). The second notation (SS2) was performed on May 15th in 2012 and April 14th in 2015, which, respectively, corresponded to stages Z49 and Z37. SS was scored using a 0–5 scale, where 0 represents no symptoms and 5 represents high mottling and stunting. Within years, SS1 and SS2 were highly correlated, but SS2 notations were slightly more discriminant and better reflected WSSMV resistance (result not shown). We hence only consider SS2 hereafter, which will be referred to as SS.

The virus concentration was quantified using DAS-ELISA (Clark and Adams 1977). For each observation unit, we randomly collected approximately 20 leaves at various developmental stages from different plants selected at random. We ground 0.4 g of the pooled bundle of those leaves in 4 mL of a custom grinding buffer (Online Resource 2). We used the antiserum of Marie-Jeanne et al. (1999) provided by SEDIAG (WSSM-SRA, Bretenière, France, http://www.sediag.com/) and followed the manufacturer’s instructions, except for the grinding buffer. A positive control was obtained using a pooled bundle of leaves collected on Pescadou plants with high SS scores, which were ground to give a single positive control extract that was aliquoted and frozen before use. Blank, negative and positive controls were included in each ELISA plate. Each sample was deposited in two successive pits on the plate. Absorbance was read at 405 nm in a micro-plate reader (Tecan, infinite F200, Männedorf, Switzerland). The ELISA score of each sample was quantified using the following formula:

$${\text{Elisa}} = \frac{{\left( {{\text{AbsS}} - {\text{blank}}} \right)}}{{({\text{AbsPos}} - {\text{blank}})}}$$

where *Elisa* is the quantitative ELISA score for a sample, *AbsS* is the absorbance of the sample, *blank* is the absorbance value of the blank of the ELISA plate, and *AbsPos* is the absorbance of the positive control of the plate.

Quantitative-PCR (qPCR) was performed on 2015 samples. The numbers of viral copies per mg of leaves were estimated as follows. RNA was extracted from 100 mg of the same leaf bundles used for ELISA. Purified RNA was then diluted ten-fold and cDNA was produced by random hexamer priming. Real-time quantitative RT-PCR was performed using the SYBR-Green Chemistry method according to the protocol described by Vaïanopoulos et al. (2006) with some modifications. Briefly, amplification was conducted with diluted cDNA and primers WSSMVc1-F and WSSMVc1-R. PCR was conducted using 40 cycles (95 °C, 15 s, 60 °C, 30 s) on an Applied Biosystems StepOne Plus device. As DNA standard, we used a purified PCR product (WSSMVc2-F–WSSMVc1-R) spanning a five-point calibration range (10^4^, 10^5^, 10^6^, 10^7^, 10^8^ copies) in the initial template (3 replicates per plate). A temperature range of 60–95 °C (0.3 °C steps) was used for melting curves. Two technical replicates per cDNA were amplified. Numbers of viral copies per mg of leaves were obtained using the calibration. After all considerations, copy numbers are given for 22.8 µg of fresh leaves. All technical details are provided in Online Resource 2. Analyses were carried out on the log of the copy numbers.

### Statistical analysis

We partitioned the variance of each of the three resistance phenotypes (SS, ELISA and qPCR) into genetic and environmental variance components using the ASReml-R package in R (asreml 3.0, VSN International, Hempel Hemstead, UK; Gilmour et al. 2009) and R 3.3.2 (http://www.r-project.org/; R Development Core Team 2013). The data was analyzed using the following full linear mixed model with a normal error distribution:

1 $$\varvec{z} = \varvec{Xb} + \varvec{Zu} +\varvec{\varepsilon},$$

where ***z*** is a vector of individual plant observations for a given trait; ***b*** is a vector of fixed effects; ***u*** is a vector of RIL random genetic effects; $\varvec{\varepsilon}$ is a vector of random errors; and ***X*** and ***Z*** are incidence matrices relating the observations to the fixed and random effects, respectively (see Online Resource 3 for details).

Fixed effects in ***b*** for each trait consisted of: (1) the overall mean of this trait, (2) two linear covariates to account for possible linear environmental trends in virus concentration in the row and column directions, and (3) a type effect which accounts for average differences between the two RIL populations taken as a whole and the susceptible check (two levels: DL and DS RILs vs. Pescadou). For SS and ELISA only, fixed effects also included a year effect (two levels: 2012 or 2015) and an interaction between type and year effects. We used conditional Wald F-tests with a 5% significance level on the model with the lowest AICc to test fixed effects (Gilmour et al. 1997). The corrected Akaike Information Criterion (AICc) allows comparison of models even when they are non-nested (AICc, Burnham and Anderson 2002).

For random genetic effects, variation in resistance across RILs was tested using different models for the distribution of ***u***: correlated genotypic effects between-years (model A), uncorrelated genotypic effects between years (model B), constant genotypic effects between years (model C). For qPCR in 2015, a simple random genotype effect was estimated (model D). Comparing models A and C allows testing for the existence of genotype-by-year interactions. For environmental effects, heterogeneity in symptom spatial distribution was tested using several models assuming only spatially uncorrelated errors (model 1, estimating a $\sigma_{\text{uncor}}^{ 2}$ variance), only spatially correlated errors (model 2, $\sigma_{\text{cor}}^{ 2}$ variance) or both types of error (model 3, the error variance is partitioned between $\sigma_{\text{cor}}^{ 2}$ and $\sigma_{\text{uncor}}^{ 2}$) (Gilmour et al. 1997).

Random effects were tested based on their AICc. Each model was ranked according to the difference between its AICc and the AICc of the model with the lowest AICc. Following Burnham and Anderson (2002), we considered that models with $\Delta {\text{AICc }} < 2$ were strongly supported by the data. The BLUP of each genotype was computed based on the model with lowest AICc and used for QTL analyses (see “QTL analyses” below). Individual heritabilities were computed for each trait using estimates from the best model and their standard errors were computed using the delta method (Lynch and Walsh 1998). All details are provided in Online Resource 3.

### QTL analyses

We used the QTLRel program (Cheng et al. 2011), implemented in R, for QTL analysis of the two DS and DL datasets. Sister lines (similarity >80%) were detected among our RILs after genotyping (27 in DS and 13 in DL), probably resulting from confusion during the fixation process in nurseries. Although these lines were removed from the map construction, we considered their phenotypes as worthy for increasing the QTL detection power as long as the line relatedness was explicitly declared. QTLRel implements mixed models allowing a random polygenic effect using different kinship matrices, while taking the inherent relatedness among individuals into account (Cheng et al. 2010). We used the *GenMatrix* option of QTLRel, which uses the simple kinship coefficient matrix in the model and estimates it directly from the marker data.

We performed a single marker analysis on BLUP values to compute a LOD score per marker. QTL detection was first performed independently for each population and each year. A joint-QTL analysis was then performed using both populations on markers found to be polymorphic in both DS and DL populations, but all markers were used to compute the kinship correlation matrix. Finally, a last analysis was computed, taking together both years and populations, to evaluate QTL × year interaction. The LOD threshold for declaring a QTL as significant for a trait in an experiment was obtained by permutations of genotypes relative to phenotypes. We used the value of the 95% maximum LOD score values obtained from 1000 independent permutations as the significance threshold (Churchill and Doerge 1994). Since this threshold was always between 3.24 and 3.61, a conservative LOD threshold of 3.61 was used for all traits. QTL confidence interval regions (in cM) were defined as the ±1.5 LOD-interval around the peak LOD values of each QTL (Mangin et al. 1994). Allelic effects were defined as half the difference between the BLUP means of favorable and unfavorable homozygous genotypes.

All R scripts and data used for those statistical and QTL analyses are available in Online Resource 4 and on Github (https://github.com/holtzy) for reproducibility.

## Results

### Genotyping and consensus linkage map constructions

An average of 2.5 and 4 million reads per sample were obtained for DS and DL, respectively, and 86 and 75% of these reads were mapped successfully on DWr, the durum wheat reference transcriptome. After filtering on Fis, coverage and He, we retained 3734 SNPs for DS and 6887 SNPs for DL. Their parental allelic states were consistent with previous RNAseq data on these parents.

We assembled 22 and 31 linkage groups (LGs) for DS and DL, respectively. Those assemblies contain 14 and 16 large LGs (≥100 markers), respectively, which is consistent with the 14 chromosomes of durum wheat. The remaining LGs, although having fewer markers, were assigned to known chromosomes and assembled in the genetic maps. For DS, five markers were not linked to any other SNP and were not used in the genetic map (only one for DL). Finally, the individual DS and DL genetic maps consisted of 3729 and 6886 markers, respectively. The consensus genetic map contained 8568 markers, with 2047 markers in common between the maps. The main features for each chromosome of the three maps are provided in Table 2, and the mapping positions of individual markers are given in Online Resource 5.

**Table 2:** Chromosome assignments, number of SNPs, and sizes of chromosome linkage groups of the DS, DL and consensus durum wheat genetic maps

Chr.	DS map (Dic2 × Silur)			DL map (Dic2 × Lloyd)			Consensus map				
	#SNPs	Size (cM)	Unique pos.	#SNPs	Size (cM)	Unique pos.	#SNPs	Size (cM)	Unique pos.	Avg. inter marker distance	Spearman r with phys. pos.
1A	231	175.5	90	394	239.6	142	496	206.6	166	0.42	0.97
1B	298	181.4	121	597	277.3	228	750	230.8	266	0.31	0.94
2A	403	218.3	132	644	325.3	209	820	271.7	228	0.33	0.76
2B	324	234.9	141	638	341.3	219	785	293.3	269	0.37	0.96
3A	204	199.6	97	414	294.2	182	504	255.2	213	0.51	0.98
3B	337	229.7	142	615	310.2	231	747	271.1	263	0.36	0.93
4A	231	229.3	115	428	236.3	155	520	232.4	206	0.45	0.94
4B	281	163.7	100	541	227.4	157	659	193.5	178	0.29	0.83
5A	279	288.2	134	512	359.5	214	649	319.7	265	0.49	0.99
5B	280	246.4	134	499	308.8	199	660	278.5	257	0.42	0.99
6A	172	178.2	96	345	239.2	152	434	208.7	190	0.48	0.99
6B	253	183.4	114	483	241	176	564	213.5	205	0.38	0.94
7A	292	237.6	135	451	334.2	185	587	285.4	228	0.49	0.99
7B	144	197.3	73	325	253.2	139	393	227.7	160	0.58	0.96
Mean	266.4	211.7	116.0	491.9	284.8	184.9	612.0	249.2	221.0	0.42	0.94
Total	3729	2964	1624	6886	3988	2588	8568	3488	3094		

For each chromosome and each map, the number of SNPs (#SNPs), total chromosome size in centimorgans (Size (cM)), and the number of unique marker positions (unique pos.) are given. For the consensus genetic map, the average distance between two adjacent SNPs is also provided (Avg. inter marker distance = Size(cM)/#SNPs) as well as the Spearman’s rank correlation coefficients between this consensus genetic map and the putative physical positions (Spearman *r* with phys. pos.)

A majority (77%) of DL markers grouped in the same LG also had a common chromosomal putative assignment on BWr. In both cases, assignment inconsistencies were mainly due to homeologous competitive genome assignment (e.g., assignment to 1A instead of 1B). As putative assignment was determined by blast hits on BWr, a high degree of similarity between homeologous genes (or the absence of a homeologous copy in the reference transcriptome) may lead to erroneous chromosome assignment. Translocations observed in the DS map were confirmed in the DL map. Indeed, 20 markers of the LG group representing chromosome 7A were initially putatively assigned by blast to chromosome 4A (reciprocally 15 on 4A initially assigned on 7A) and 14 markers of chromosome 4B were putatively assigned to chromosome 5A (reciprocally 14). This validates the translocation hypothesis proposed in Holtz et al. (2016).

**Fig. 1:**
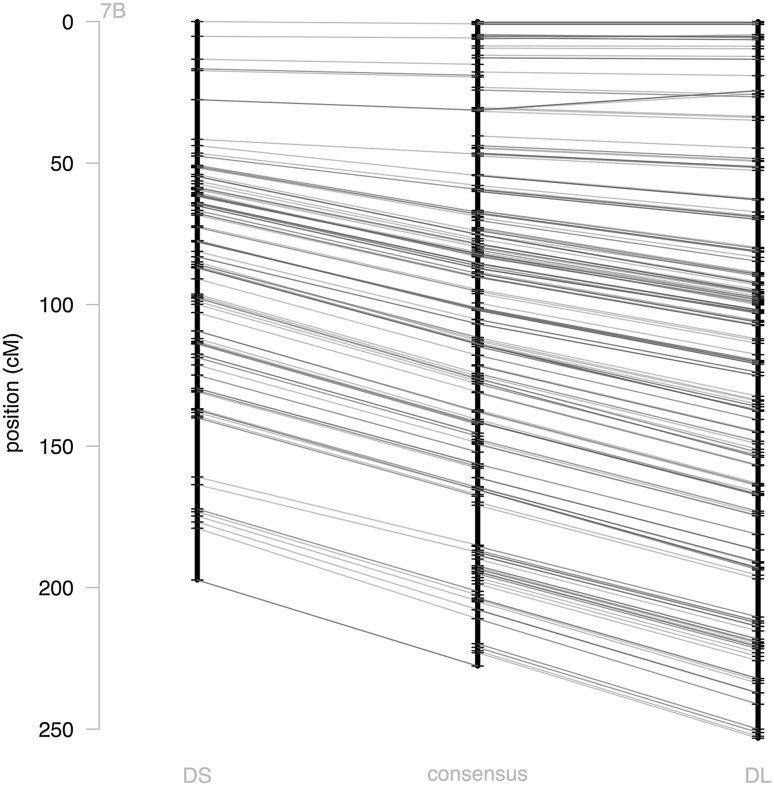
Consensus and individual map representation. The three genetic maps of the chromosome 7B: DL map on the *left*, DS map on the *right* and the consensus map in the *middle*. Common *markers* of adjacent maps are linked by *blue lines* (color figure online)

The DL map was 1.35-fold longer than the DS map, with 3988 cM (for 6886 SNPs) versus 2964 cM (for 3729 SNPs). The consensus map length was 3488 cM (for 8568 SNPs) and well resolved with a high number of markers per chromosome (average of 612, ranging from 393 for chromosome 7B to 820 for chromosome 2A). The average inter marker genetic distance was 0.42 cM and ranged from 0.29 (chromosome 4B) to 0.58 cM (chromosome 7B). The longest gap per chromosome was 12 cM long on average, but was always smaller than 20.3 cM (chromosome 1A). This consensus map contained 3094 unique positions, well spread among and along the chromosomes. The marker distribution along the three maps and the colinearity between markers of the three maps are represented in Fig. 1 for chromosome 7B, and in Online Resource 6 for all other chromosomes. These figures illustrate the high density of the three maps as well as their consistency.

The genetic ordering of the SNPs within the consensus map was highly consistent with the physical positions of their contigs in the BWr. Indeed, the Spearman’s rank correlations between those two SNP orders were 0.9 on average (min 0.76 for chromosome 2A, max 0.99 for 5A). The four SSR markers were linked to SNP markers of the 7B chromosome, at positions 0.0, 5.3, 27.6, and 54.7 cM for *Xbarc1068*, *Xbarc323*, *Xgwm40*0, and *Xgwm46*, respectively.

### Evaluation of WSSMV resistance

Details on the values for the selection of the best models according to their AICc and likelihoods are reported in the tables of Online Resource 7.

#### Phenotypic variation

No significant year effect was observed, except on Pescadou. Genitors of the two RIL populations presented the expected phenotype: Dic2, the resistant genitor, showed high resistance and elite genitors Silur and Lloyd were highly affected in both years, with a mean SS ranging from 4.1 to 4.7. The DS and DL RIL populations showed marked segregation, with the sensitivity of WSSMV accessions ranging from very low (SS ~0) to very high (SS ~5) (Table 3).

**Table 3:** Symptom severity and virus concentration and qPCR value for the two recombinant inbred line (RIL) populations evaluated in field trials in 2012 and 2015

	DS-2012		DL-2012		DS-2015			DL-2015		
	SS	ELISA	SS	ELISA	SS	ELISA	qPCR	SS	ELISA	qPCR
Mean	2.01	0.87	2.28	0.87	2.07	0.62	8.83	2.48	0.72	10.06
Min	0.00	0.04	0.00	0.03	0.00	0.01	−4.57	0.00	0.01	−1.98
Max	5.00	2.76	4.50	2.39	5.00	1.35	17.55	5.00	1.39	18.45
CV	0.51	0.56	0.48	0.54	0.66	0.71	56.90	0.56	0.59	49.87
*H* ^2^	0.67	0.41	–	–	0.66	0.79	0.61	0.66	0.77	0.61

SS and ELISA are mean visual assessment of symptom severity and ELISA absorbance value (virus concentration), respectively. Min, Max, CV, and *H* ^2^ are the measurement range (minimum and maximum), phenotypic coefficient of variation, and broad sense heritability values, respectively (no repetition available for DL 2012)

Pearson coefficients of correlation between SS and ELISA infection evaluations were 0.53 for 2012 and 0.66 for 2015. ELISA and qPCR correlations were high (0.72). This denoted a slight discrepancy between the three WSSMV infection evaluation methods. The heritability of ELISA was higher than SS (Table 3) in 2015, but lower in 2012.

#### Spatial heterogeneity of WSSMV infection

For all traits, model 3 (with the environmental error being partly and spatially correlated and uncorrelated) always had the lowest AICc (Table S1A, S2A and S3A, respectively). The two variance components, $\sigma_{\text{cor}}^{ 2}$ and $\sigma_{\text{uncor}}^{ 2}$ had a similar within-year order of magnitude (e.g., ELISA 2012, $\sigma_{\text{cor}}^{ 2}$ = 0.1; $\sigma_{\text{uncor}}^{ 2}$ = 0.12, ELISA 2015, $\sigma_{\text{cor}}^{ 2}$ = 0.02; $\sigma_{\text{uncor}}^{ 2}$ = 0.04, Table S2D). This suggests that virus infection was not uniform within the experimental field (Online Resource 8). In addition, the best models consistently included spatial auto-correlations both in the row and column directions of the field. The column auto-correlation parameter, $\rho_{\text{c}}$, was always higher than the row auto-correlation parameter, $\rho_{\text{r}}$, (e.g., $\rho_{\text{c}}$ = 1, $\rho_{\text{r}}$ = 0.47, ELISA 2012, Table S2D). This difference was consistent with smaller distances (and hence higher correlations) between adjacent columns (20 cm apart) than adjacent rows (1.5 m apart). As no significant general row and column effects were detected, this heterogeneity was fine grained and patchy (Table S1B and S2B). For qPCR, models 3 and 1 (without spatial auto-correlation) had similar AICc values (Table S3A), with a slightly higher likelihood for model 3. Furthermore, we detected a trend for $\rho_{\text{c}}$ and $\rho_{\text{r}}$ similar to that observed in SS and ELISA.

For random genetic effects in SS or ELISA, model A (correlated genotypic effects between years) always had the lowest AICc (Tables S1A and S2A, respectively). Genetic variances were consistently higher in 2015 compared to 2012 (e.g., for ELISA, 0.07 *vs*. 0.13 in DL and 0.08 vs. 0.14 in DS, Table S2D). ELISA heritabilities were higher in 2015 (0.78 in DS and 0.77 in DL) than in 2012 (0.41 in DS and 0.41 in DL), whereas SS heritabilities were similar for the 2 years and populations (0.65 for both DS and DL, Table 3). This indicates that, in 2015, the genetic variance in viral load increased relatively more than $\sigma_{\text{uncor}}^{ 2}$, whereas the genetic variance in SS increased by the same order of magnitude as $\sigma_{\text{uncor}}^{ 2}$. For qPCR, heritabilities were equal between the two RIL populations (0.62 for both DS and DL, Table 3). For SS and ELISA, genetic correlations between 2012 and 2015 were positive and consistently higher than 0.6 (e.g., for ELISA, 0.78 for both DL and DS, Table S2D). The stronger support for model A compared to model C indicates the existence of some genotype-by-year interactions. However, for both DS and DL, the genetic covariances between RILs observed in the 2 years were very close to the genetic variances (OR7, Table S2D). This indicates that genotype-by-year interactions are very low and that the between-year RIL rank correlation is very high.

#### Resistance: relationship with plant height and earliness

Phenotypic correlations between WSSMV resistance and plant height or precocity were always very low. Pearson correlations between SS, ELISA and qPCR BLUPs and heading or earliness ranged from 0.02 to 0.14 for DS, or −0.01 to 0.03 for DL. No correlations were obtained between WSSMV resistance and plant height (Pearson correlation in the [−0.24; −0.15] range for DS, and [−0.12; 0.09] for DL). This suggests that neither height nor earliness affected WSSMV resistance.

### Genetic control of WSSMV resistance

**Fig. 2:**
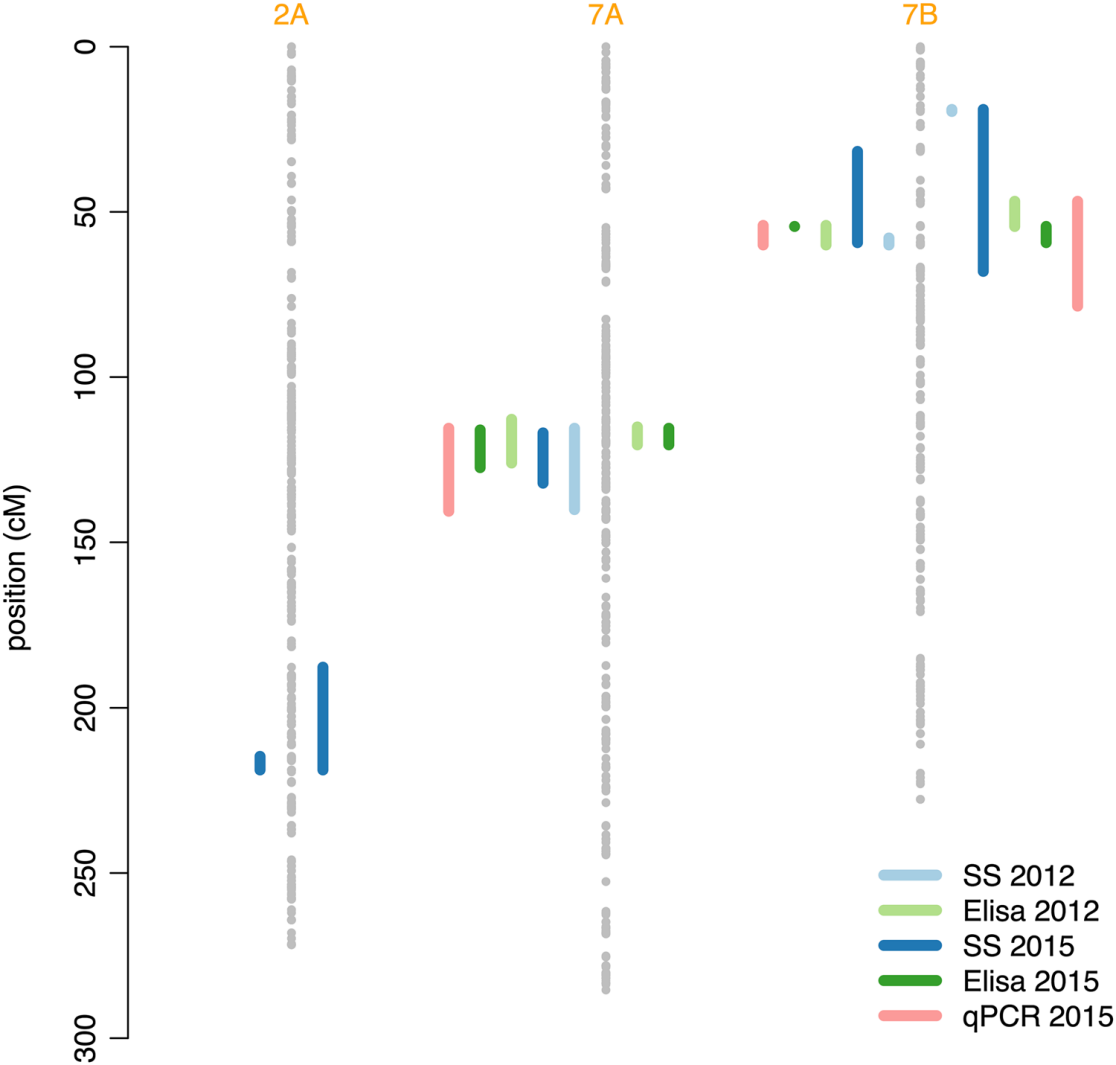
Consensus genetic map and detected QTLs. Chromosome 2A, 7A and 7B are represented by three lines of *grey points*. Each point is a marker of the consensus genetic map. The genetic scale (in cM) is provided on the *left side*. Locations of QTL confidence intervals are indicated by *colored lines*: *light* (2012) and *dark* (2015) *blue lines* for SS; *light* (2012) and *dark* (2015) *greens lines* for ELISA, and *pink* for qPCR (2015). *Colored lines* on the *left* of the chromosome depict QTLs found within the DS population, while *colored lines* on the *right* depict QTLs identified within the DL population (color figure online)

**Fig. 3:**
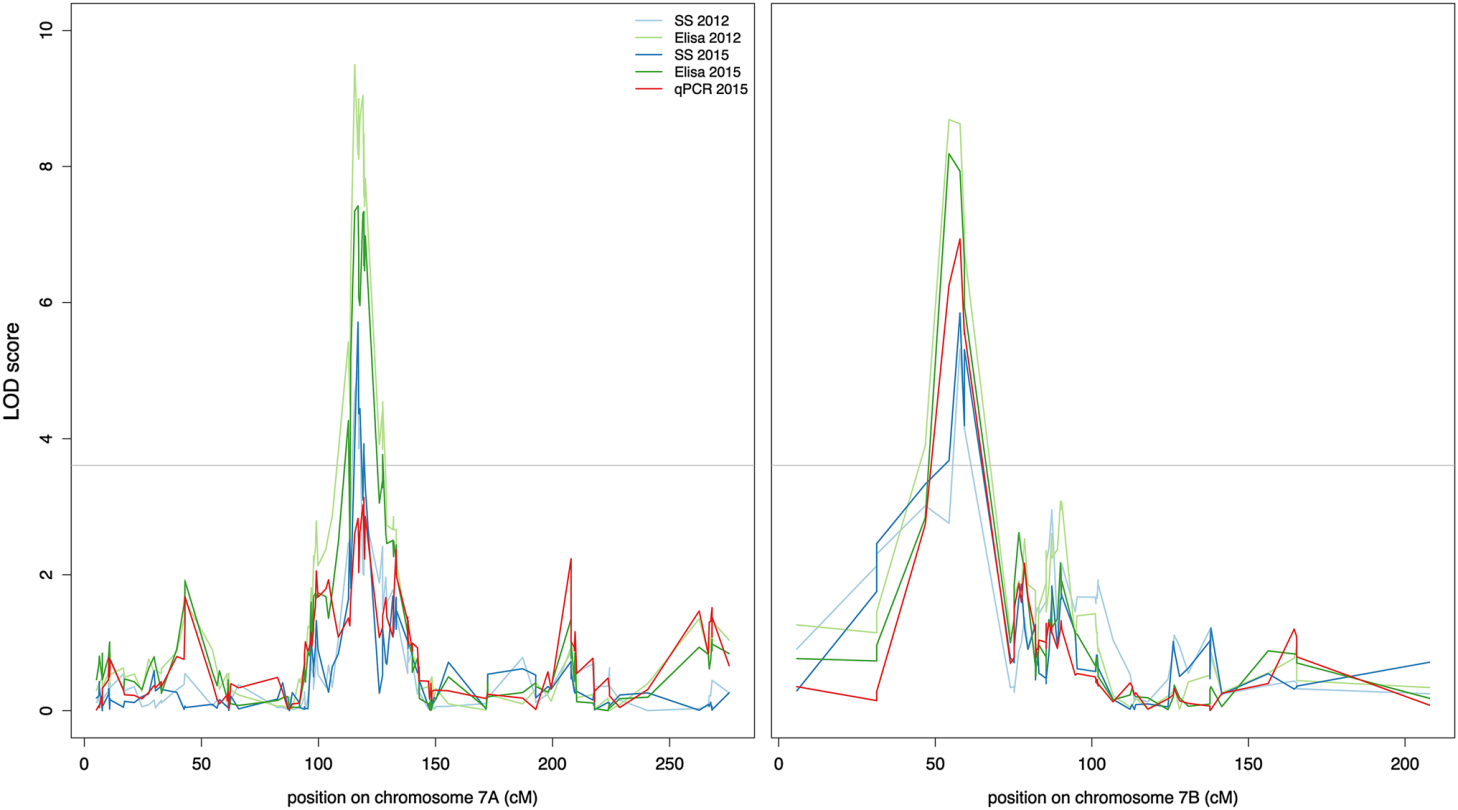
LOD-scores for markers on chromosomes 7A and 7B. LOD scores for association with WSSMV resistance detected by simple interval mapping with QTLRel are represented for all markers of chromosomes 7A and 7B. To improve the readability, points representing LOD scores of each phenotypic trait are linked by *colored lines* (according to the color convention used in Fig. 2) (color figure online)

The joint-QTL detection combined the 165 and 189 RILs of DS and DL populations genotyped for 2047 common SNPs. Significant QTL features are summarized per population in Online Resource 9 and located on our consensus genetic map in Fig. 2. A first major QTL, hereafter called *Qssm*-*mtpsa*-*7BS*, was detected in the distal area of the short arm of chromosome 7B. A second QTL was observed on chromosome 7A in the sub-centromeric area, hereafter called *Qssm*-*mtpsa*-*7A*. All WSSMV resistance traits were partly explained by these QTLs for both years (Fig. 3).

For *Qssm*-*mtpsa*-*7BS*, the LOD scores ranged from 5.32 (SS in 2012) to 8.69 (ELISA in 2012). This QTL explained between 5.79 and 9.56% of the total phenotypic variation. For all phenotypic traits, the LOD scores were highly congruent. The ±1.5 LOD score confidence interval highlighted a locus spanning ~5 cM [54.4–59.3 cM] on chromosome 7B. This confidence interval contained 10 SNPs. Eight of them were spread on a putative physical zone ranging from 36.6 to 56 Mb, with the two remaining distal markers being located at 111 and 142 Mb in the BWr. The *Qssm*-*mtpsa*-*7BS* showed a significant interaction effect with year (*P* < 0.001) for ELISA, but not for SS. This interaction effect was due to a scaling effect, the Dic2 allele being the resistant allele in both years.

For *Qssm*-*mtpsa*-*7A*, LOD scores ranged from 5 (SS 2012) to 9.5 (ELISA in 2012). This QTL explained between 5.42 and 10.42% of the BLUP variation. For all phenotypic traits, the LOD scores were highly congruent and highlighted a small area of 3.9 cM [115.5–119.4 cM]. The confidence interval contained 11 SNP markers distributed in a putative physical zone ranging from 43.7 to 55 Mb. As for *Qssm*-*mtpsa*-*7BS*, the *Qssm*-*mtpsa*-*7A* showed a significant interaction effect with year (*P* < 0.001) for ELISA, but not for SS. As observed for *Qssm*-*mtpsa*-*7BS*, the interaction effect of *Qssm*-*mtpsa*-*7A* was due to a scaling effect, the Dic2 allele being still the resistant allele in both years.

A minor QTL (*Qssm*-*mtpsa*-*2AL*) was detected on the long arm of chromosome 2A for SS 2015, SS 2012 and ELISA 2015. The LOD scores ranged from 3.61 to 5.82 and explained from 3.8 to 6.29% of the BLUP variance. The QTL was in the [214.7–227.1] cM confidence interval containing 18 markers spanning a relatively short putative physical area ranging from 237.1 to 241.3 Mb. An interaction effect between year and *Qssm*-*mtpsa*-*2AL* was detected for both SS and ELISA (*P* < 0.001), underlying a stronger effect of the favorable Dic2 allele in 2015 than in 2012.

These QTLs were also detected on individual maps (Fig. 2), but with larger confidence intervals (Online Resource 10) and no additional population-specific QTLs were detected. This denoted the very high consistency of these QTL between years, populations and phenotyping methods. The favorable allele conferring resistance was inherited from Dic2 in all cases.

#### Epistatic interaction between the two major QTLs

**Fig. 4:**
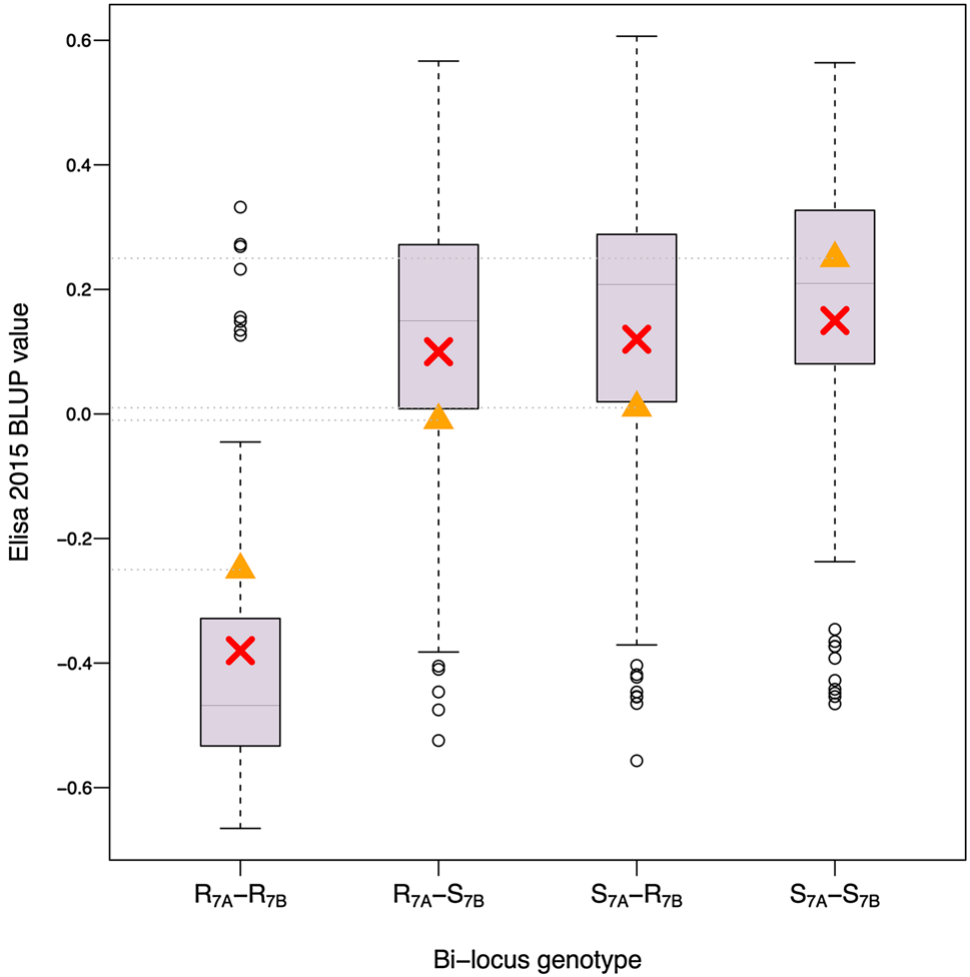
Epistatic interaction between *Qssm*-*mtpsa*-*7BS* and *Qssm*-*mtpsa*-*7A* QTL for ELISA in 2015 on 345 RILs. Let R_7A_ (resp. R_7B_) denote the resistant allele at *Qssm*-*mtpsa*-*7A* (resp. *Qssm*-*mtpsa*-*7BS*) and S_7A_ (resp. S_7B_) the corresponding sensitive alleles. Individual BLUP value distributions are summarized by *box-plots* for R_7A_-R_7B_, R_7A_-S_7B_, S_7A_-R_7B_, S_7A_-S_7B_ haplotypes. Plants having a single resistance allele were almost as sensitive as those having none, while plants bearing both favorable alleles at *Qssm*-*mtpsa*-*7BS* and *Qssm*-*mtpsa*-*7A* were highly resistant. DS and DL variances were similar in 2012 and 2015. Thus DS and DL populations were pooled together to maximize the power of detection of additive and interaction effects

Interaction effects between *Qssm*-*mtpsa*-*7BS* and *Qssm*-*mtpsa*-*7A* were tested using a simple linear model declaring additive and interaction effects by marker pairs. For simplicity, we used a single combination of markers for the analyses. Each QTL was represented by its best marker (i.e., having a peak LOD score in the single marker analysis). A significant (*P* < 10^−6^) interaction was observed for all trials and resistance phenotypes (Online Resource 9). For ELISA 2015, for example, the actual difference between the double resistant R_7A_-R_7B_ (resistant alleles at the two loci *Qssm*-*mtpsa*-*7A* and *Qssm*-*mtpsa*-*7BS*) and the double susceptible S_7A_-S_7B_ (corresponding sensitive alleles) was −0.53, while the difference between the values predicted by additive effects was −0.25 only (with a_7A_ = −0.075 and a_7B_ = −0.06, Online Resource 9). Plants having a single resistance allele were almost as sensitive to WSSMV as those having none, while plants carrying both the favorable alleles at *Qssm*-*mtpsa*-*7BS* and *Qssm*-*mtpsa*-*7A* were highly resistant (Fig. 4). First and second order interactions involving *Qssm*-*mtpsa*-*2A* were not significant (result not shown). Epistasis was sought on all the rest of the genome exploring all 5 cM × 5 cm possible pairs for the five phenotypes. Among all pairs, only four were detected as significant at a q value < 0.05 (1A-92.8 cM + 6B-152.4 cM for SS-2012, 4A-24.5 cM + 5B-37 cM for ELISA 2012, 2A-170.7 cM + 4B-156.9 cM for Elisa-2015 and 3A-14.6 cM + 5A-231.7 cM for ELISA-2015), but with low *r* ^*2*^ and no consistency among years and we decided not to consider them for further discussion. Considering the high number of comparisons, we considered them as likely false positive pairs.

## Discussion

### Locus targeted capture: high efficiency for genotyping and map building

In a previous study, we demonstrated that genotyping by capture is well suited for durum wheat genotyping and genetic map construction (Holtz et al. 2016). Major improvements were carried out for the DL map: only two baits per SNP were designed for greater productivity, SNPs within or nearby microsatellite-like regions were not used for bait design and blocking oligos were used during the capture phase. Overall, these measures significantly decreased the undesired capture of repeated sequences and allowed almost doubling of the number of useful SNPs that passed our quality check filters—from 3729 for DS to 6886 for DL, i.e., an 84% increase in productivity. The resulting DL map contains 2588 unique positions, more than most of the durum wheat maps published to date, including those built with the 90 K wheat array (Maccaferri et al. 2014).

Our DS and DL-maps share 2047 markers, i.e., 55% of the DS and 30% of the DL markers. The genetic order of the common markers are in close agreement in the two maps: the Spearman’s rank correlation between both map orders along the 14 durum wheat chromosomes ranges from 0.99 to 1. This allowed us to build a highly reliable consensus map containing 3094 unique positions with 8568 markers. This consensus map shows strong colinearity (Spearman correlation >0.92) with the bread wheat reference (except for chromosomes 2A (Spearman correlation 0.76) and 4B (Spearman correlation 0.84). This consensus map is thus a highly valuable tool for QTL mapping in durum wheat. The expected publication of the durum wheat genome (Distelfeld 2016) will be an invaluable resource to determine the physical position of the markers presented here.

Our consensus genetic map (3488 cM) is longer than other durum or cultivated emmer maps [2635 cM (Maccaferri et al. 2014)], and SNP-based maps of wild wheat [2258 cM (Avni et al. 2014)]. This kind of difference between genetic map lengths in durum wheat has already been reported (Leflon et al. 2010; Vaissayre et al. 2012). Here, the increased map length could be due to a high per se recombination rate of the Lloyd cultivar, which has already been detected in a previous study (Vaissayre et al. 2012). Variations in recombination rate among genotypes have also be documented and tested in other species (Esch et al. 2007). We observed that the number of crossovers was significantly higher in DL than in DS RILs (68 vs. 50.3 crossovers on average per RIL, *P* < 10^−8^).

The DL map and proposed physical assignments in BWr show the same chromosome assignment discrepancies as those previously documented between the DS map and BWr assignments (Holtz et al. 2016). This confirms that durum wheat has some translocations between chromosomes 4B and 5A, and between chromosomes 4A and 7A, which may differ from those of bread wheat.

### Phenotyping the WSSMV resistance of durum wheat

We observed some slight within-year inconsistencies between the phenotyping methods for the same sample. Several explanations could be proposed: (1) SS evaluation could be biased if WSSMV symptoms are confused with other symptoms, e.g., due to possible rare spots of SBCMV; (2) ELISA tests are based on an extract of 0.4 g from a bundle (ca. 20) of randomly drawn leaves, while an expert could spot one individual plant with symptoms among 30–40 plants to score SS; and (3) symptomless carriers may exist if some plants/genotypes have WSSMV tolerance (Carroll et al. 1997). Although qPCR is described as a very sensitive (and expensive) method (Vaïanopoulos et al. 2006), it could even be more dependent on the sampling procedure than ELISA since only 0.1 g of leaves are sampled to represent the two rows of an experimental unit. Indeed, qPCR yielded lower heritability in our study than SS and ELISA. This confirms that sampling is a crucial point for virus evaluation, especially when the attack is mild, as may have been the case at Pray. An alternative procedure could be envisaged for ELISA and qPCR, e.g., harvesting, pooling, and grinding a large quantity of leaves and then sampling the extracted juice. However, this would be almost untractable and very expensive for such a large number of analyses. Evaluating WSSMV resistance with SS and ELISA appeared to be the best compromise.

Spatial auto-correlation of symptoms confirmed the findings of some previous studies (Cadle-Davidson et al. 2006), indicating that the soil virus concentration may be highly variable at the field level or that some environmental factor may locally modify the virus virulence (such as local immersion of roots). Our model describing spatial heterogeneity enhanced the accuracy in RIL BLUP values, therefore, increasing the LOD scores at peak QTL detection values (up to 1.5 LOD in the best cases, data not shown). This increased accuracy led to smaller confidence intervals around the markers showing the peak LOD values.

Although WSSMV symptom expression could vary depending on the climatic conditions (Cadle-Davidson et al. 2006), our data suggested that the genotype resistance ranking of our lines was very stable, since the between-year genetic correlation was high compared to the trait heritability.

### Role of homeologous group 7 in virus resistance

Two main QTLs for WSSMV resistance were found on chromosomes 7A and 7B. These QTLs were consistently detected through years, phenotyping methods and populations. Joint analysis of the two RIL populations enabled us to achieve LOD scores of over 8 and to reduce the confidence interval to 4.2 and 4.9 cM for QTLs 7A and 7B, respectively. These two QTLs were not in homeologous positions (56 cM on average on 7B and 116 cM on the 7A). Their underlying causal genes may thus not be linked to the same resistance mechanisms. Another QTL, *Qsbm*-*ubo*-*7BS*, has been reported on chromosome 7B in durum wheat for SBCMV resistance, positioned between markers *Xbarc106*8 and *Xgwm400* (Maccaferri et al. 2011) or below *Xgwm400* (Maccaferri et al. 2012; Russo et al. 2012). This region was projected between 0 and 27.6 cM on our high-resolution consensus genetic map, suggesting that *Qssm*-*mtpsa*-*7BS* differs from Qsbm-ubo-7BS, but further investigations are needed to sort out this question. Another minor QTL concerning the WYMV resistance of bread wheat was described by Zhu et al. (2012) on chromosome 7B, alongside marker *Xgwm46*. This marker is located at 54.7 cM on our consensus genetic map, colocalizing near *Qssm*-*mtpsa*-*7BS*. A cluster of expressed resistance genes (R genes) have been also reported on homeologous group 7 (Dilbirligi et al. 2004).

### Role of group 2 chromosomes

In our study, a QTL (*Qssm*-*mtpsa*-*2AL*) was detected in the sub-telomeric region of the long arm of chromosome 2A (227 cM). Other QTLs for mosaic virus resistance have also been reported on homeologous group 2, a distal QTL on chromosome arm 2BS (6–18 cM, *QSbm.ubo*-*2BS*) for SBCMV resistance in durum wheat (Maccaferri et al. 2011; Russo et al. 2012), a distal QTL for WYMV (*Qym1*) on chromosome 2DS (60 cM) in bread wheat (Suzuki et al. 2015), and a QTL for resistance to WSSMV on chromosome arm 2DL in bread wheat (Khan et al. 2000). Although we could not determine the colocalizations for the QTL on chromosomes 7, it is unlikely that the genes underlying the QTLs mentioned above are homeologous.

For A and B genomes, homeologous groups 2 and 7 had the highest number of well annotated R genes on the bread wheat sequence (Bouktila et al. 2015), but much more in-depth investigations are still needed to confirm whether the virus resistance is determined by R genes.

In our study, we did not detect any QTL in other important groups such as the group 5 [Sbm1 for SBCMV resistance in bread wheat on 5DL (Bayles et al. 2007)] or the group 3 [Qym2 for WYMV resistance in bread wheat (Zhu et al. 2012)]. Therefore, multiple sources of resistance exist for these *P. graminis* transmitted viruses (Online Resource 11).

### Epistatic interactions

There was a synergistic effect between the resistance alleles at 7A and 7B QTLs that were first detected upon their additive effect. For instance, if we simply considered R_7A_ versus S_7A_ plants, a significant difference could be noted since R_7A_ consisted of 50% resistant R_7A_R_7B_ and 50% susceptible R_7A_S_7B_ when S_7A_ plants were all susceptible. This mimicked an additive effect at the 7A locus while based on a positive epistatic interaction. When considered separately, each of these two QTLs explained between 5 and 9% of the BLUP variance only, but between 22 and 43% when considered together. On average, plants having the two resistant alleles had much stronger resistance than plants having one favorable allele or none (Fig. 4). Such complex genetic control has already been observed for two other viruses transmitted by *P. graminis* (Walker et al. 1998; Gutiérrez et al. 2010). On WYMV, a bymovirus relatively close to WSSMV (Liu et al. 2016), the Qym1 and Qym2 locus also seemed to have a positive interaction effect, as resistant alleles at the two loci were necessary to get a high level of resistance (Suzuki et al. 2015).

The genetic determinism of virus resistance thus appeared to be quite complex since different chromosomal regions are involved and interact with the WSSMV. If such epistatic interactions were confirmed in a larger number of situations, this could lead to different QTL effect estimates according to the polymorphism between the parents involved in the cross. Epistasis can turn into additivity if the two parents of a cross share a common allele at one locus implied in the resistance (7B or 7A). Hence, as a result of successive bottlenecks reducing diversity since domestication (Haudry et al. 2007), elite durum may be monomorphic at many loci and *durum* × *durum* crosses may exhibit mostly additive effects at QTLs while *dicoccum* × *durum* crosses may yield more complex situations.

### Resistance mechanisms

Two bymoviruses (WSSMV and WYMV) are infectious on wheat and induce rather similar symptoms (Clover and Henry 1999). Although they are not very close from a molecular standpoint [i.e., 70% of identity on their coat protein (Liu et al. 2016)], resistance mechanisms may share some similarities. In bread wheat, WYMV resistance is not based on the prevention of root colonization by *P. graminis*, but rather on the inhibition of viral multiplication in the root cells or viral transmission among them (Liu et al. 2016). As the WSSMV QTLs detected in our study did not colocalize with *Qym1*, *Qym2* nor *Qym3* (Suzuki et al. 2015), the molecular basis of resistance may be different. However, another known mechanism of resistance to bymovirus or furovirus is translocation resistance: the resistant plant can restrict upward root-to-shoot virus translocation (Carroll et al. 2002; Kanyuka et al. 2004; Bass et al. 2006; Lyons et al. 2009). Moreover, the resistance mechanism of Dic2 still has to be explored, notably by testing the different genotypic combinations of the three QTLs for their ability to control virus multiplication in the roots or its transmission to the leaves.

The existence of strong epistatic positive interactions between *Qssm*-*mtpsa*-*7A* and *Qssm*-*mtpsa*-*7BS* suggests that two successive steps are at stake or that two genes are necessary to control one resistance component. As the two major QTLs are located in medium recombining areas, additional work remains to develop markers closer to the causal genes and then to clone the underlying causal genes.

### Plant breeding and WSSMV resistance

The difficulty and cost of acquiring WSSMV phenotypic values emphasize the interest of a molecular breeding approach. Marker-assisted selection (MAS) is all the more promising as the QTLs in this study were consistently detected for different years and populations, and thus seemed to be very good proxies for up to 40% of the WSSMV resistance variation. The sequence information provided in this study will be useful for developing PCR-based SNP markers through Kompetitive Allele Specific PCR (http://www.lgcgroup.com/) or other assays for marker-assisted selection of the QTLs in breeding programs.

The two major QTLs should be selected together, which may transfer a genetic burden to bred lines if the Dic2 emmer carries some unfavorable alleles close to these loci. However, the QTLs are not located close to major agronomic loci such as the *Rht1* locus on chromosome arm 4BS (Gale and Marshall (1976) or the *Vrn* locus (Yan et al. 2004). Furthermore, WSSMV resistance was not found to be correlated with plant height or flowering date. So we hope that these two *QSwm.Mtps* could be mobilized in breeding programs with almost no detrimental effects on agronomic value.

We do not know if the genetic determinism of the WSSMV resistance of Dic2 is different from that of Soldur. If the resistance genes turn out to be different, which is possible since emmer and durum wheat have many specific alleles, the use of both resistance sources would provide an opportunity to better manage resistance sustainability.

#### **Author contribution statement**

YH analyzed the data, wrote most of the paper and coordinated the writing process. MB supervised the field experiment and performed the visual phenotyping. VV performed the ELISA and qPCR analysis. MA produced the DNA libraries and produced the genomic data. GP and VV created and maintained the RILs. VV, VR, NOR, VMJ and PR were involved in discussions concerning data production and analysis, while also participating in the writing process. SS supervised the biomolecular work. DG and JD initiated the project. JD proposed the method, supervised the work and was involved in the writing. All authors read and approved the final version of the paper. We warmly thank Christèle Cornier for administrative help.

## Electronic supplementary material

Below is the link to the electronic supplementary material.

**Online Resource 1: Genotyping by capture protocol.** Description of the protocol used to capture DNA using the myBait technology with specific oligos (DOCX 27 kb)

**Online Resource 2: Protocol used for ELISA and qPCR.** Description of the protocol used to phenotype leaf samples with ELISA and qPCR (DOCX 22 kb)

**Online Resource 3: Statistical analysis details.** Complete description of the statistical analyses performed in this study (DOCX 35 kb)

**Online Resource 4: Data and R scripts for reproducible QTL detection.** Data and R script (.csv and.rmd format) are provided in this tar archive. A scheme aims to explain the content of each file and its role in the QTL detection pipeline. The upstream bioinformatic steps (from raw reads to consensus genetic map) are not included (GZ 72829 kb)

**Online Resource 5: Genetic maps.** This Excel file contains three sheets giving information concerning i) the consensus genetic maps, ii) the DS map and iii) the DL map. The information is organized as an array with three fields in a row per marker: the chromosome assigned to this marker, the marker name, and the marker positions (in cM) within the chromosome. Marker names are composed by the name of the contig containing the SNP, an “@”, and the position in the contig where the SNP is located (XLSX 497 kb)

**Online Resource 6: Visualization of the genetic maps.** For each chromosome, three parallel black lines represent the three genetic maps (DL, consensus and DS) with lengths represented in cM. The consensus map is represented in the middle, with the DS map on its left and the DL map on its right. Each marker is represented by a black point, indicating its position along the chromosome. Blue lines link common markers between two adjacent maps (PDF 139 kb)

**Online Resource 7: Model selection using AICc.** This file gives details concerning the selection of the model used for STL detection. It provides, for each trait (SS, ELISA and qPCR), the AICc and main features of all tested models (XLSX 63 kb)

**Online Resource 8: Observation of the spatial heterogeneity of WSSMV infection.** Experiments of 2012 and 2015 are represented in two distinct sheets. Each cell represents an accession. The cell color reflects the mean symptom severity observed in the direct neighborhood of the corresponding accession (including itself). Red indicates a strong infection (SS = 5) and white indicates no infection (SS = 0) (PDF 3167 kb)

**Online Resource 9: Details of every QTL detected.** Here we report details on every QTL detected with simple interval mapping analysis. Meta-QTLs are first reported, followed by QTLs of the DS and DL RILs. For each QTL, we reported: LOD score, position and name of the marker with the highest LOD, LOD-1.5 confidence interval, additive effects. This latter is defined here as half of the difference between the mean value of the RILs carrying the susceptible allele (Silur and Lloyd) and the RILs with the resistant allele (Dic2). SS denotes symptom severity (XLSX 73 kb)

**Online Resource 10: Visualization of QTLs along chromosome 7A and 7B for DS and DL.** Four graphics are provided that depict LOD scores observed for DS (two graphics on top) or DL (two graphics on bottom) along chromosome 7A (left graphics) or 7B (right). In each graphic, LOD scores for association with WSSMV resistance detected by simple interval mapping with QTL Rel are represented for every marker. The LOD scores of each phenotypic variable (SS, ELISA and qPCR in 2012 and 2015) are represented by a specific color (PDF 26 kb)

**Online Resource 11: QTLs detected for virus resistance in the literature.** Review of published QTL of resistance to viruses transmitted by *P. graminis* in wheat (XLSX 57 kb)
