# Supplementary material for: Epistatic determinism of durum wheat resistance to the wheat spindle streak mosaic virus
Source: Theor Appl Genet. 2017 Apr 27;130(7):1491–505. doi: 10.1007/s00122-017-2904-6 (PMC5487696; doi:10.1007/s00122-017-2904-6)
Supplement: Supplementary file 1 — Online Resource 1: Genotyping by capture protocol. Description of the protocol used to capture DNA using the myBait technology with specific oligos (DOCX 27 kb) [file 122_2017_2904_MOESM1_ESM.docx]

**Epistatic determinism of durum wheat resistance to the Wheat Spindle Streak Mosaic Virus**

### *Yan Holtz^1*^, Michel Bonnefoy^3^, Véronique Viader^2^, Morgane Ardisson^2^, Nicolas O. Rode*^2^*, Gérard Poux^2^, Pierre Roumet^2^, Véronique Marie-Jeanne^2^, Vincent Ranwez^1^, Sylvain Santoni^2^, David Gouache^3^, Jacques L. David^1*^*

**Online Resource 1: GENOTYPING BY CAPTURE PROTOCOL**

**Plant DNA purification**

DNA was extracted from 100 mg of fresh young leaves with the Chemagic DNA Plant Kit (Perkin Elmer Chemagen, Baesweller, DE, Part # CMG-194), according to the manufacturer’s instructions. The protocol is adapted to the use of the KingFisher Flex™ (Thermo Fisher Scientific, Waltham, MA, USA) automated DNA purification workstation.

**Construction of enriched library and sequencing**

Genomic library preparation for multiplexed individuals and enrichment step by capture follow published protocols of Rohland and Reich, 2012 and Mascher et al, 2013 respectively with some modifications.

**A Target preparation, construction of barcoded genomic libraries**

1 : For each individual, 1 µg of total DNA (in 100 µL of water) are sheared using a Bioruptor Pico (Diagenode, Seraing, BE) sonication device in 500 µl microtubes to a targeted 300 bp DNA fragment size using parameters of the 300pb standard protocol for DNA shearing. *(*[*https://www.diagenode.com/files/protocols/Standard_protocols_for_DNAShearing.pdf*](https://www.diagenode.com/files/protocols/Standard_protocols_for_DNAShearing.pdf)*).*

2: 400 ng of fragmented DNA (in 40 µl of water) are blunted and 5’ phosphorylated using the Thermo Scientific Fast DNA End Repair Kit (Thermo Fischer Scientific, Waltham, MA, USA, Part # K0771). A clean-up step is performed with 1 x volume of Agencourt AMPure XP magnetic beads. The elution volume is 20 µL.

3: Fragmented and repaired DNA are individually controlled (sizing and estimation of the concentration) by electrophoresis on a AATI Fragment Analyzer™ (Advanced Analytical Technologies, Ankeny, IA, USA) device with the DNF-474 High Sensitivity Fragment Analysis Kit.

4: 50 ng of fragmented DNA are ligated with 4 pmol of PE-P5 and MPE-P7 adapters. Each PE-P5 and each MPE-P7 adapter carries the same specific hexamer barcode (modified protocol from [Rohland & Reich 2012](#_ENREF_1)). Reactions are conducted in 15 µl final volume with 1 unit of T4 DNA ligase for 1 hour at 22 °C followed by a heat inactivation step at 65°C for 10 minutes.

5: 48 samples (corresponding to 48 hexamer barcodes on the PE-P5 and PE-P7 adapter) are pooled. A clean-up step is performed with 1.8x volume of Agencourt AMPure XP magnetic beads. The elution volume is 94µL.

6: A nick fill-in step is performed using 64 units of Bst DNA polymerase (New England Biolabs, Ipswich, MA, USA, Part # M0275), 1x ThermoPol® reaction buffer, 250 µM dNTP in 120 µl final volume and incubated for 15 minutes at 37°C. A clean-up step is performed with 1.8x volume of Agencourt AMPure XP magnetic beads. The elution volume is 40 µL.

7: For each pool of 48 samples, a pre-hybridization PCR is performed using the Phusion® High-Fidelity PCR Master Mix (Thermo Fischer Scientific, Part # 1040-2678) with 200 nM PreHyb-PE_F (ctttccctacacgacgctcttc) and 200 nM PreHyb-MPE_R (TGACTGGAGTTCAGACGTGTG) primers in a final volume of 100µl.

Thermocycling parameters: 3 minutes at 98°C, followed by 12 cycles of 80 seconds at 98°C; 45 seconds at 55°C and 60 seconds at 68°C, with a final elongation of 10 minutes at 72°C. A clean-up step is performed with 1.8x volume Agencourt AMPure XP magnetic beads. The elution volume is 10 µL.

**B Enrichment, capture by hybridisation**

The protocol used is based on Mascher et al, 2013, the User Manual of the MYBaits Sequence Enrichment for Targeted Sequencing kit (<http://www.mycroarray.com/pdf/MYbaits-manual-v2.pdf>) and on the Roche NimbleGen SeqCap EZ Library SR User’s Guide (<http://sequencing.roche.com/products/nimblegen-seqcap-target-enrichment/seqcap-reagents.html>**)**

**B-1 Hybridization of the barcoded libraries to biotinylated RNA probes**

8: Prior to hybridization, 10 μl of Roche Diagnostics (Indianapolis, IN, USA) proprietary SeqCap EZ Developer Reagent (Roche, Part # 06684335001) were added to a 1.5-ml tube containing 0,5 μg of the 48 barcoded samples genomic library.

Next were added as blocking oligos:

1 µl (100 pmol/µl solution) of the P5 adapter blocking oligo , 5’-AGATCGGAAGAGCGTCGTGTAGGGAAAG and 1 µl (100 pmol/µl solution) of the MP7 adapter blocking oligo, 5’-AGATCGGAAGAGCACACGTCTGAACTCCAGTCA, designed to block the truncated segment of TruSeq DNA library adapters during the sequence capture.

2 µl of the mixed solution (33 pmol/µl each) of Block-GAA, 5’GAAGAAGAAGAAGAAGAAGAA, Block-GGA 5’-GGAGGAGGAGGAGGAGGAGGA and Block-CAA 5’-CAACAACAACAACAACAACAA designed to block repetitive sequences of wheat and to reduce the Daisy-chain reaction previously described.

The mixture was dried down in a SpeedVac at 60°C during 20 to 30 min.

9: 7.5 μl of 2 × Sequence Capture Hybridization Buffer (tube 5, SeqCap EZ Hybridization and Wash Kit, Roche, Part # 05634261001) and 3 μl of Hybridization Component A (tube 6, SeqCap EZ Hybridization and Wash Kit, Roche, Part # 05634261001) were added. The hybridization cocktail was vortexed for 10 sec and collected by centrifugation. Following denaturation in a heat block (95°C, 10 min) the sample was transferred to a 0.2 ml PCR tube containing 80 ng of biotinylated RNA probes (4.5 µL of MYBaits Capture Probe).

10: The hybridization sample (15 μl) was incubated in a thermocycler (lid heated to 57°C) at 47°C for 64–72 h.

**B-2 Washing of the captured library. Biotinylated probes annealed to the target complexes are then immobilized with Streptavidin coupled magnetic beads.**

11: Streptavidin coupled magnetic beads are previously equilibrate as recommended by the Roche-Nimblegen protocol. Invitrogen Dynabeads MyOne™ Streptavidin C1 (Invitrogen, Thermo Fischer Scientific, Part # 65001) at 10 µg/µl were thoroughly vortexed, aliquoted (50 μl per hybridization) into 1.5-ml tubes and prepared for the affinity purification of captured DNA. The tubes were placed in a DynaMag-2 magnet (Invitrogen, Part # 123-21D) for 2 min. The clear liquid was discarded and 100 μl of 1 X Bead Wash Buffer (Tube 7, SeqCap EZ Hybridization and Wash Kit, Roche, Part # 05634261001) were added. Tubes were vortexed, placed back in the magnet, the clear liquid was removed, and the washing was repeated once. Dynabeads were resuspended in 50 μl 1 x Bead Wash Buffer, transferred into PCR plates and collected using a Agencourt SPRIPlate 96R (Agencourt, Part # A32782) . The clear supernatant was discarded.

12: The hybridization sample was added to the wet Dynabeads and mixed thoroughly by pipetting up and down. Using a thermocycler (lid heated to 57°C) at 47°C for 45 min the captured sample was bound to the Dynabeads. The sample was vortexed for 3 sec in 15-min intervals to ensure that the Dynabeads remain in suspension. Dynabeads plus bound DNA (15 μl) were washed by adding 100 μl 1 X Wash Buffer 1 (pre-heated to 47°C for 1 h) (Tube 1, SeqCap EZ Hybridization and Wash Kit, Roche, Part # 05634261001) and vortexing for 10 sec.

13: The suspension was transferred to a 1.5-ml tube and placed in a DynaMag-2 device, and the supernatant was discarded once clear. Washing was continued by adding 200 μl 1 X Stringent Wash Buffer (pre-heated to 47°C for 1 h) (Tube 4, SeqCap EZ Hybridization and Wash Kit, Roche, Part # 05634261001) The sample was mixed by pipetting avoiding a major temperature drop and incubated for 5 min at 47°C. The tube was placed in the DynaMag-2 magnet, the liquid was discarded and the washing at 47°C with 1 X Stringent Wash Buffer was repeated once.

14: 200 μl 1 X Wash Buffer 1 (pre-heated to room temperature) was added to the Dynabeads plus bound DNA. The sample was vortexed for 2 min and the liquid was collected to the tube's bottom. Following magnetic concentration the liquid was discarded, and the sample was washed at room temperature with 200 μl 1 X Wash Buffer 2 (vortexing for 1 min) (Tube 2, SeqCap EZ Hybridization and Wash Kit, Roche, Part # 05634261001), followed by a wash with 200 μl 1 X Wash Buffer 3 (vortexing for 30 sec) (Tube 3, SeqCap EZ Hybridization and Wash Kit, Roche, Part # 05634261001) as described for washing with Wash Buffer 1. The tube was removed from the magnet, the bead-bound captured library was resuspended in 22 μl PCR-grade water and the entire sample (beads + liquid) was transferred to a 0.2 ml PCR tube.

**3° PCR post-capture and sequencing**

15: An on- beads PCR amplification is undertaken to enrich library fragments, extend the adaptor sequence and incorporate an index to the P7 adaptor. The PCR reaction is using the KAPA® HiFi HotStart ReadyMix PCR Kit (KAPABiosystems, Boston, MA, Part # KR0370) with 500 nM SOL-PE-PCR_F (aatgatacggcgaccaccgagatctacactctttccctacacgacgctcttc) (Rohland and Reich, [2012](#_ENREF_1)) primer and 500 nM SOL-MPE-INDX_R (CAAGCAGAAGACGGCATACGAGATXXXXXXGTGACTGGAGTTCAGACGTGT) indexed primers . This primer carries 6 bases of the official Illumina Index. Thermocycling parameters: 2 minutes at 98°C, followed by 18 cycles of 20 seconds at 98°C; 30 seconds at 62°C and 30 seconds at 72°C, with a final elongation of 5 minutes at 72°C. The reaction volume is 50 µL. A clean-up step is performed with 1.8x volume Agencourt AMPure XP magnetic beads. The elution volume is 20 µL.

16: Indexed libraries are individually controlled (sizing and estimation of the concentration) by electrophoresis on a AATI Fragment Analyzer™ device with the DNF-474 High Sensitivity Fragment Analysis Kit.

17: Four indexed libraries, corresponding to 192 captured barcoded DNA samples, are equally mixed. The final pooled library is quantify by qPCR with the KAPA Library Quantification Kit (Part # KK4824) and provided to the Get-PlaGe core facility (GenoToul platform, INRA Toulouse, France http://www.genotoul.fr) for sequencing.

18: The final pooled library is sequenced using the Illumina paired-end protocol on a single lane of a HiSeq3000 sequencer, for 2 x 150 cycles.

References

Rohland N, Reich D (2012) Cost-effective, high-throughput DNA sequencing libraries for multiplexed target capture. *Genome Research* **22**, 939-946.

Mascher, M., Richmond, T. A., Gerhardt, D. J., Himmelbach, A., Clissold, L., Sampath, D., ... & Akhunov, E. D. (2013). Barley whole exome capture: a tool for genomic research in the genus Hordeum and beyond. *The Plant Journal*, *76*(3), 494-505.
