# Supplementary material for: Epistatic determinism of durum wheat resistance to the wheat spindle streak mosaic virus
Source: Theor Appl Genet. 2017 Apr 27;130(7):1491–505. doi: 10.1007/s00122-017-2904-6 (PMC5487696; doi:10.1007/s00122-017-2904-6)
Supplement: Supplementary file 2 — Online Resource 2: Protocol used for ELISA and qPCR. Description of the protocol used to phenotype leaf samples with ELISA and qPCR (DOCX 22 kb) [file 122_2017_2904_MOESM2_ESM.docx]

**Epistatic determinism of durum wheat resistance to the Wheat Spindle Streak Mosaic Virus**

### *Yan Holtz^1*^, Michel Bonnefoy^3^, Véronique Viader^2^, Morgane Ardisson^2^, Nicolas O. Rode*^2^*, Gérard Poux^2^, Pierre Roumet^2^, Véronique Marie-Jeanne^3^, Vincent Ranwez^1^, Sylvain Santoni^2^, David Gouache^4^, Jacques L. David^1*^*

**Online Resource 2: ELISA PROTOCOL**

The virus concentration was determined on 20 diluted extracts from fresh or (depending on the experimental year) -80°C or -20°C frozen leaves collected on various leaf levels along two field lines of 10-20 plants, in parallel with visual evaluation, using DAS (double antibody sandwich) ELISA (enzyme-linked immunosorbent assay). Each 0.4 g sample from a whole leaf sample bundle was ground in 4 mL of grinding buffer (tri-sodium citrate C_6_H_5_Na_3_O_7_, (114 mM); urea, (0.5 M), Tween (0.05 % v/v) at pH: 7.4). Most of the grinding was done using grinding bags (ACC00930, Bioford) and a Homogenizer hand model 40001 (Bioreba). Leaf “juices” were centrifuged for 5 min at 5000 g and at 5°C. Supernatants were diluted twice with grinding buffer and deposited on SEDIAG anti-WSSMV antibody (WSSM-SRA) coated plates (Maxisorb™(442.404 VWR)). Coating buffer: Na_2_CO_3_: 0.015M, NaHCO_3_: 0.034M, miliQ water: qsp 1L, pH 9.6. Coating step, diluted juice deposition step and conjugate deposition step, were performed according to the manufacturer’s (SEDIAG) instructions. At each step, plates were incubated for 3 h at 37°C or overnight at 6°C, with the plates being kept in a wet environment. Between each step, plates were washed three times with PBS-Tween-PVP (PBS + 2% m/v PVP + 0.05% v/v Tween, pH 7.4). Conjugated antibodies were diluted on (PBS-Tween-PVP + 0.2% m/v of chicken albumin (A5503, Sigma)). The enzyme substrate (*p*-nitro-phenyl-phosphate, pNPP (ACC00404, Biofords), was diluted in substrate buffer (diethanol amine ((D8885, Sigma) 97: 1000 v/v (water), pH 9.8), 1 mg/mL. After 1 h of substrate incubation (out of light) at 25°C, DO (optical densities = absorbances) were read at 405 nm. Each sample was duplicated and each plate had eight “white” wells (empty wells), four positive and four negative controls. To minimize inter-plate variation, DO were used when transformed as follows: log((DOs-DOw)/(DOpc-DOw)). DOs = two sample well DO mean; Dow = plate eight white wells DO mean; DOpc = plate positive control well DO mean.

**Online Resource 2: QPCR PROTOCOL**

The virus concentration was determined on 20 miliQ water diluted cDNAs obtained from 10 diluted RNAs extracted from 100 mg of the same leaf bundles as used for the ELISA evaluation. Leaf samples were weighed and then stored at -80°C. RNA was extracted using the Sigma spectrum kit, cDNA was produced using the A3500 Promega kit, both according to the manufacturer’s instructions. For RNA extraction, only 400 mL of lysing solution was transferred on the extraction column to avoid saturating it. Standards for calibration curves were obtained by purifying a bulk of various positive PCR products. The purified bulk was dosed using a nanodrop device and the number of RNA copies was calculated on the basis of the known PCR product length. The purified product evaluated in this way was used at dilutions ranging the copies number from 10^4^ to 10^8^ in triplicate. Each cDNA sample was duplicated. We used an Applied Biosystems StepOne Plus device, using 0 cycles at 95°C, 15 s, 60°C, 30 s, PCR conditions. The primers used were as follows: 5’-3’) WMc1F = 5’-AGCAACCCTTAGCGAAGTCA-3’ et WMVc1R = 5’-AGGGACGTGGAACAAAGAAA-3’ (Vaïanopoulos et al. 2006). The sample mix was: qsp 5µL of 10 diluted cDNA, 0.5 µL of primers at 10 pmol/µL, water: 4 µL, Fast SyberGreen (TF, 5 pack fast SYBR Green master mix (ref10566555, Fischer): 10 µL. Specific consumables were used (TF, optical fast 96-well PLT, ref : 10310855 et FG, optical adhesive covers, ref : 10299204, Fisher). Melting curves were done from 60°C to 95°C by 0.3°C steps.
