# Supplementary material for: Epistatic determinism of durum wheat resistance to the wheat spindle streak mosaic virus
Source: Theor Appl Genet. 2017 Apr 27;130(7):1491–505. doi: 10.1007/s00122-017-2904-6 (PMC5487696; doi:10.1007/s00122-017-2904-6)
Supplement: Supplementary file 3 — Online Resource 3: Statistical analysis details. Complete description of the statistical analyses performed in this study (DOCX 35 kb) [file 122_2017_2904_MOESM3_ESM.docx]

**Epistatic determinism of durum wheat resistance to the Wheat Spindle Streak Mosaic Virus**

### *Yan Holtz^1*^, Michel Bonnefoy^3^, Véronique Viader^2^, Morgane Ardisson^2^, Nicolas O. Rode*^2^*, Gérard Poux^2^, Pierre Roumet^2^, Véronique Marie-Jeanne^2^, Vincent Ranwez^1^, Sylvain Santoni^2^, David Gouache^3^, Jacques L. David^1*^*

**Online Resource 3: Statistical analyses**

The data were analyzed using the following full linear mixed model with a normal error distribution:

| $\boldsymbol{z=Xb+}\boldsymbol{Z}_{\boldsymbol{DL}}\boldsymbol{u}_{\boldsymbol{DL}}\boldsymbol{+}\boldsymbol{Z}_{\boldsymbol{DS}}\boldsymbol{u}_{\boldsymbol{DS}}\boldsymbol{+\varepsilon}$, | (1) |
| --- | --- |

where $\boldsymbol{z}$ is a vector of individual plant observations for a given trait; $\boldsymbol{b}$ is a vector of fixed effects; $\boldsymbol{u}_{\boldsymbol{DL}}$ and $\boldsymbol{u}_{\boldsymbol{DS}}$ are vectors of random additive genetic effects of DL and DS RILs of dimensions equal to the number of DL and DS RILs respectively; $\boldsymbol{\varepsilon}$ is a vector of random errors. $\boldsymbol{X}$, $\boldsymbol{Z}_{\boldsymbol{DL}}$ and $\boldsymbol{Z}_{\boldsymbol{DS}}$ are incidence matrices relating the observations to the fixed and random effects, respectively.

*Fixed effects*

The vector $\boldsymbol{b}$ consisted in either three (qPCR) or four fixed effects (SS and ELISA). Details are provided in the main text.

*Random genetic effects*

For SS and ELISA analyses, genetic effects could potentially differ between 2012 and 2015. We tested three models with different variance-covariance matrices for $\boldsymbol{u}_{\boldsymbol{DL}}$ and $\boldsymbol{u}_{\boldsymbol{DS}}$. Genetic effects could either differ between years with a genetic covariance (model A), or differ between years with no genetic covariance (model B) or be the same across the two years (model C). For qPCR, only a single model (model D) could be fitted since there were no qPCR measurements in 2012.

For model A, $\boldsymbol{u}_{\boldsymbol{DL}}$ and $\boldsymbol{u}_{\boldsymbol{DS}}$ were assumed to follow a normal distribution with zero mean vectors and variance-covariance matrices:

| $V\left[ \boldsymbol{u}_{\boldsymbol{DL}} \right]=\left[ \begin{matrix} \sigma_{{DL}_{2012}}^{2} & \sigma_{{DL}_{2012-2015}} \\ \sigma_{{DL}_{2012-2015}} & \sigma_{{DL}_{2015}}^{2} \end{matrix} \right]\bigotimes\boldsymbol{I}_{\boldsymbol{n}_{\boldsymbol{DL}}}$, | (2) |
| --- | --- |

and

| $V\left[ \boldsymbol{u}_{\boldsymbol{DS}} \right]=\left[ \begin{matrix} \sigma_{{DS}_{2012}}^{2} & \sigma_{{DS}_{2012-2015}} \\ \sigma_{{DS}_{2012-2015}} & \sigma_{{DS}_{2015}}^{2} \end{matrix} \right]\bigotimes\boldsymbol{I}_{\boldsymbol{n}_{\boldsymbol{DS}}}$, | (3) |
| --- | --- |

where $\boldsymbol{I}_{\boldsymbol{n}_{\boldsymbol{DL}}}$ and $\boldsymbol{I}_{\boldsymbol{n}_{\boldsymbol{DS}}}$ represent identity matrices of dimension equal to the number of DL and DS RILs respectively, and $\bigotimes$ represents the Kronecker product.$\sigma_{{DL}_{2012}}^{2}$ and $\sigma_{{DL}_{2015}}^{2}$ (respectively, $\sigma_{{DS}_{2012}}^{2}$ and $\sigma_{{DS}_{2015}}^{2}$) correspond to the genetic variances for DL-2012 and DL-2015 crosses (respectively, DS-2012 and DS-2015 crosses) and where $\sigma_{{DL}_{2012-2015}}$ (respectively, $\sigma_{{DS}_{2012-2015}}$) is the genetic covariance between DL-2012 and DL-2015 RILs (respectively, between DS-2012 and DS-2015 RILs). Note that the susceptible check had random effects set at zero in 2012 and 2015, as we only fitted the average trait value for this genotype.

For model B, $\boldsymbol{u}_{\boldsymbol{DL}}$ and $\boldsymbol{u}_{\boldsymbol{DS}}$ were assumed to follow a normal distribution with zero mean vectors and variance-covariance matrices:

| $V\left[ \boldsymbol{u}_{\boldsymbol{DL}} \right]=\left[ \begin{matrix} \sigma_{{DL}_{2012}}^{2} & 0 \\ 0 & \sigma_{{DL}_{2015}}^{2} \end{matrix} \right]\bigotimes\boldsymbol{I}_{\boldsymbol{n}_{\boldsymbol{DL}}}$,  and | (4) |
| --- | --- |
| $V\left[ \boldsymbol{u}_{\boldsymbol{DS}} \right]=\left[ \begin{matrix} \sigma_{{DS}_{2012}}^{2} & 0 \\ 0 & \sigma_{{DS}_{2015}}^{2} \end{matrix} \right]\bigotimes\boldsymbol{I}_{\boldsymbol{n}_{\boldsymbol{DS}}}$, | (5) |

The definitions of the different variance components are the same as above. For model C, we ignored potential year effects and fitted a reduced model with $V\left[ \boldsymbol{u}_{\boldsymbol{DL}} \right]=\sigma_{DL}^{2}\boldsymbol{I}_{\boldsymbol{n}_{\boldsymbol{DL}}}$ and $V\left[ \boldsymbol{u}_{\boldsymbol{DS}} \right]=\sigma_{DS}^{2}\boldsymbol{I}_{\boldsymbol{n}_{\boldsymbol{DS}}}$, where $\sigma_{DL}^{2}$ and $\sigma_{DS}^{2}$ represent the genetic variance for DL and DS RIL populations, respectively.

*Random environmental effects*

For each of the three traits, we tested for spatial heterogeneity in virus distribution by fitting three different models with a zero mean vector and different variance structures for the errors. We assumed different environmental variances in 2012 and 2015 (with $\boldsymbol{\varepsilon}^{\boldsymbol{'}}$ = ($\boldsymbol{\varepsilon}_{\boldsymbol{2012}}^{\boldsymbol{'}}\boldsymbol{,}\boldsymbol{\varepsilon}_{\boldsymbol{201}\boldsymbol{5}}^{\boldsymbol{'}}$**)**, for data ordered by year). Model 1 corresponds to the traditional model in quantitative genetics with independently and normally distributed errors within each year:

| $Var\left[ \boldsymbol{\varepsilon} \right]=Var\left[ \begin{matrix} \boldsymbol{\varepsilon}_{\boldsymbol{2012}} \\ \boldsymbol{\varepsilon}_{\boldsymbol{2015}} \end{matrix} \right]=\left[ \begin{matrix} \sigma_{uncor2012}^{2} & 0 \\ 0 & \sigma_{uncor2015}^{2} \end{matrix} \right]{\bigotimes\boldsymbol{I}}_{\boldsymbol{n}}$, | (6) |
| --- | --- |

where $\sigma_{uncor2012}^{2}$ and $\sigma_{uncor2015}^{2}$ are variances of the errors and $I_{n}$ is the identity matrix of rank equal to the number of observations.

Model 2 corresponds to a first-order auto-regressive (AR1) model (Gilmour et al. 1997). For each year, we fitted different auto-correlation parameters in the row and column directions, as the space between rows and columns differed. The errors, $\boldsymbol{\varepsilon}_{\boldsymbol{2012}}$ and $\boldsymbol{\varepsilon}_{\boldsymbol{2015}}$ were normally distributed and for data ordered as rows within columns within years:

| $Var\left[ \boldsymbol{\varepsilon}_{\boldsymbol{2012}} \right]= \sigma_{cor2012}^{2}\left( \Sigma_{c2012}\bigotimes\Sigma_{r2012} \right)$, | (7) |
| --- | --- |

and

| \| $Var\left[ \boldsymbol{\varepsilon}_{\boldsymbol{2015}} \right]= \sigma_{cor2015}^{2}\left( \Sigma_{c2015}\bigotimes\Sigma_{r2015} \right)$. \| (8) \| \| --- \| --- \| |  |
| --- | --- | --- | --- |

where $\sigma_{cor2012}^{2}$and $\sigma_{cor2015}^{2}$ are variances of the spatially correlated errors, $\Sigma_{c2012}$, $\Sigma_{c2015}$ and $\Sigma_{r2012}$, $\Sigma_{r2015}$ represent AR1 correlation matrices in the column and row directions, respectively. Briefly, the correlation between the residuals $\varepsilon_{p}$ and $\varepsilon_{p+d}$ of two individuals at positions $p$ and $p+d$ within the same row (respectively, the same column) is: ${\mathrm{cor}(\varepsilon}_{p},\varepsilon_{p+d})= \rho_{r}^{\left| d \right|}$ (respectively, $\rho_{c}^{\left| d \right|}$). Hence, the further away two individuals are, the lower the correlations $\rho_{r}$ and $\rho_{c}$ between their errors.

Model 3 accounts for both spatially correlated and spatially uncorrelated environmental variations by combining models 1 and 2. The vector of random errors $\boldsymbol{\varepsilon}$ was defined as:

| $Var\left[ \boldsymbol{\varepsilon}_{\boldsymbol{2012}} \right]= \sigma_{cor2012}^{2}\left( \Sigma_{c2012}\bigotimes\Sigma_{r2012} \right)+ \sigma_{uncor2012}^{2}\boldsymbol{I}_{\boldsymbol{n}\boldsymbol{2012}}$, | (9) |
| --- | --- |

and

| $Var\left[ \boldsymbol{\varepsilon}_{\boldsymbol{2015}} \right]= \sigma_{cor2015}^{2}\left( \Sigma_{c2015}\bigotimes\Sigma_{r2015} \right)+ \sigma_{uncor2015}^{2}\boldsymbol{I}_{\boldsymbol{n}\boldsymbol{2015}}$. | (9) |
| --- | --- |

where $\boldsymbol{I}_{\boldsymbol{n}\boldsymbol{2012}}$ and $\boldsymbol{I}_{\boldsymbol{n}\boldsymbol{2015}}$ are identity matrices of rank equal to the number of observations in 2012 and 2015 respectively (see Models 1 and 2 for the definitions of the other terms).

For SS and ELISA analyses, by default we fitted different spatially correlated and uncorrelated variances in 2012 and 2015 (heteroscedastic models). We also include a model with the same spatially uncorrelated variance in 2012 and 2015 (homoscedastic models). To our knowledge, building such model with similar spatially correlated environmental variances in 2012 and 2015 was not possible within ASReml-R. Individual heritabilities were computed for each trait based on the estimates from the best model (see Supporting Tables) as:

| $h^{2}=\frac{\sigma_{{cross}_{year}}^{2}}{\sigma_{{cross}_{year}}^{2}+\sigma_{uncor}^{2}}$, | (10) |
| --- | --- |

purposely excluding the variance $\sigma_{cor}^{2}$ due to spatially auto-correlation, so that heritabilities could be compared across sites and studies (Costa E Silva et al. 2013).

**References:**

Costa E Silva J, Potts BM, Bijma P, et al (2013) Genetic control of interactions among individuals: Contrasting outcomes of indirect genetic effects arising from neighbour disease infection and competition in a forest tree. New Phytol 197:631–641. doi: 10.1111/nph.12035

Gilmour AR, Cullis BR, Verbyla AP (1997) Accounting for natural and extraneous variation in the analysis of field experiments. J Agric Biol Environ Stat 269–293.
