# Supplementary material for: Epistatic determinism of durum wheat resistance to the wheat spindle streak mosaic virus
Source: Theor Appl Genet. 2017 Apr 27;130(7):1491–505. doi: 10.1007/s00122-017-2904-6 (PMC5487696; doi:10.1007/s00122-017-2904-6)
Supplement: Supplementary file 4 — Online Resource 4: Data and R scripts for reproducible QTL detection. Data and R script (.csv and.rmd format) are provided in this tar archive. A scheme aims to explain the content of each file and its role in the QTL detection pipeline. The upstream bioinformatic steps (from raw reads to consensus genetic map) are not included (GZ 72829 kb) [file 122_2017_2904_MOESM4_ESM.gz › TMP/SCRIPT/Explanation_Pipeline_Analysis.pptx]

## Slide 1
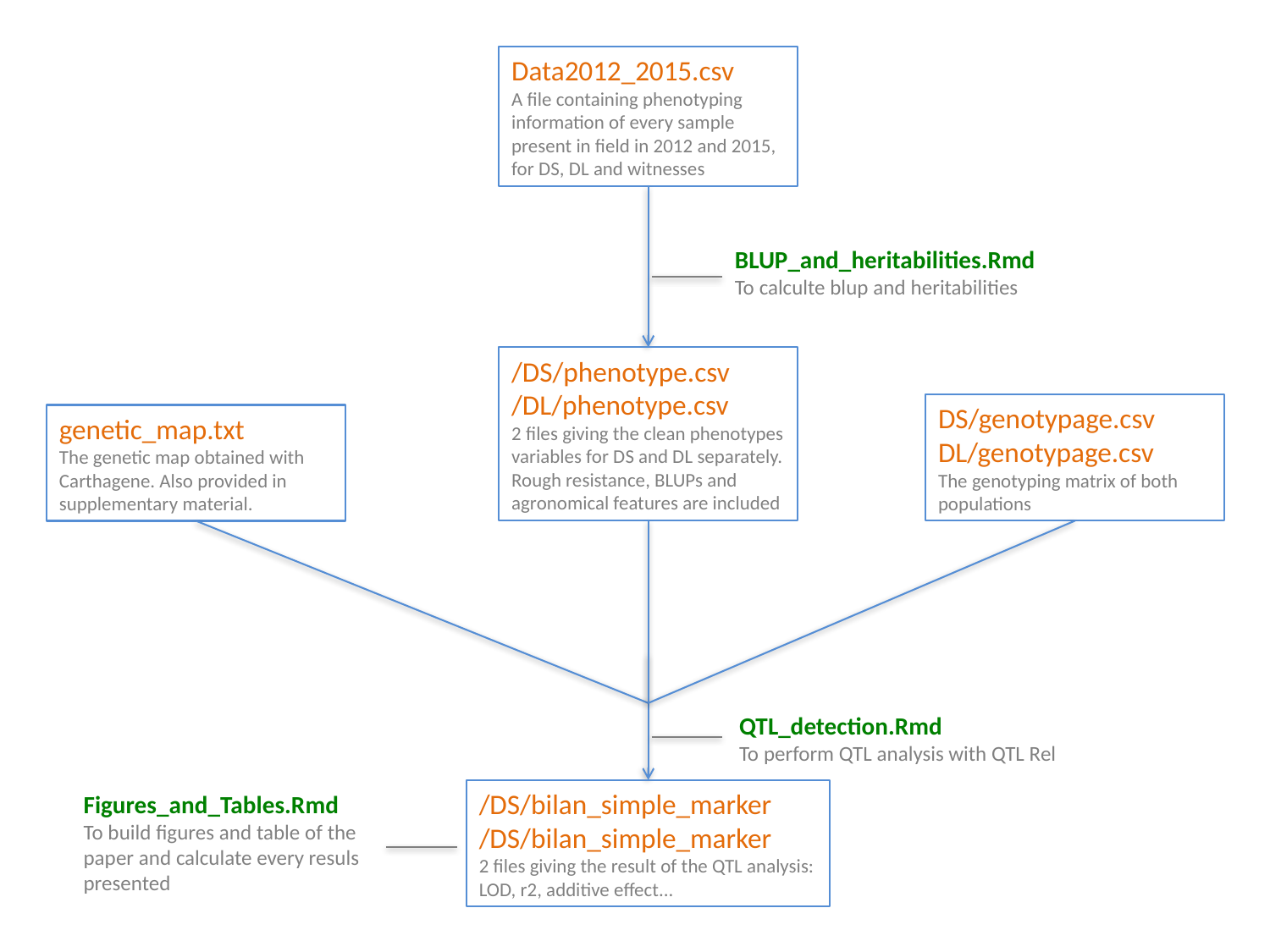

Data2012_2015.csv
A file containing phenotyping information of every sample present in field in 2012 and 2015, for DS, DL and witnesses
BLUP_and_heritabilities.Rmd
To calculte blup and heritabilities
/DS/phenotype.csv
/DL/phenotype.csv
2 files giving the clean phenotypes variables for DS and DL separately. Rough resistance, BLUPs and agronomical features are included
DS/genotypage.csv
DL/genotypage.csv
The genotyping matrix of both populations
genetic_map.txt
The genetic map obtained with Carthagene. Also provided in supplementary material.
QTL_detection.Rmd
To perform QTL analysis with QTL Rel
/DS/bilan_simple_marker
/DS/bilan_simple_marker
2 files giving the result of the QTL analysis: LOD, r2, additive effect...
Figures_and_Tables.Rmd
To build figures and table of the paper and calculate every resuls presented
