# Supplementary material for: Epistatic determinism of durum wheat resistance to the wheat spindle streak mosaic virus
Source: Theor Appl Genet. 2017 Apr 27;130(7):1491–505. doi: 10.1007/s00122-017-2904-6 (PMC5487696; doi:10.1007/s00122-017-2904-6)
Supplement: Supplementary file 4 — Online Resource 4: Data and R scripts for reproducible QTL detection. Data and R script (.csv and.rmd format) are provided in this tar archive. A scheme aims to explain the content of each file and its role in the QTL detection pipeline. The upstream bioinformatic steps (from raw reads to consensus genetic map) are not included (GZ 72829 kb) [file 122_2017_2904_MOESM4_ESM.gz › TMP/SCRIPT/4_Answer_Review/Interaction_QTL_Year/Interaction_QTL_Year_LM.html]

Interaction QTL x Year


# Interaction QTL x Year

## Interaction QTL x Year

- Introduction
  - Load data
  - Prepare data
- test1: DS - 2012
- test2: DS - 2015
- test3: DL - 2012
- test4: DL - 2015
- test5: DS AND DL - 2012
- test6: DS AND DL - 2015
- test7: DS - 2012 AND 2015
- test8: DL - 2012 AND 2015
- test9: DS AND DL - 2012 AND 2015
- test10: DS AND DL - 2012 AND 2015 - INTERACTION
- RECAP
  - Detection by year and pop separately
  - Grouping population
  - Grouping Years AND pops

This file aims to provide more details concerning the QTL x Year interaction of the resistance of Durum Wheat to WSSMV. We are going to study ELISA

```
library(QTLRel)
```

```
## R/QTLRel is loaded
```

```
library(xtable)
```

# Introduction

Can we do exactly the same thing with lm instead of QTL-Rel? The only difference is that we do not take the population structure into account.

## Load data

I load the genotyping matrix first.

```
genotype<-read.table("/Users/holtz/Dropbox/Publi_Mosaique/DATA/DATA/GROUPED/genotypage.csv", sep = ";" , header = F, na.strings = "-")
genotype=as.matrix(genotype)
colnames(genotype)=genotype[1,]
genotype=as.data.frame(genotype[-1 , ])
names(genotype)[1]<-"geno"
print("--- Your genotyping matrix looks correct. Dimension of the matrix are :")
```

```
## [1] "--- Your genotyping matrix looks correct. Dimension of the matrix are :"
```

```
print(dim(genotype))
```

```
## [1]  348 7342
```

```
# I copy this matrix 2 times, since I read 2012 and 2015 together.
rownames(genotype)=genotype[,1]
genotype=genotype[,-1]
a=genotype ; rownames(a)=paste(rownames(a),"2012",sep="_")
b=genotype ; rownames(b)=paste(rownames(b),"2015",sep="_")
genotype=rbind(a,b)
```

Then the genetic map:

```
map <- read.table("/Users/holtz/Dropbox/Publi_Mosaique/DATA/DATA/genetic_map.txt" , header=T , dec = ".", na.strings = "-" , check.names=F)
colnames(map) <- c("LG", "marqueur", "Distance","group_physique","Posi_physique")
rownames(map) <- map$marqueur
map$LG <- as.factor(map$LG)
print("--- Your genetic map looks correct. Dimension of the map are :")
```

```
## [1] "--- Your genetic map looks correct. Dimension of the map are :"
```

```
print(dim(map))
```

```
## [1] 8568    5
```

```
map=map[    , c(2,1,3,5)]
colnames(map)=c("snp","chr", "dist", "phyPos")
```

And finally the phenotyping matrix

```
BLUP<-read.table("/Users/holtz/Dropbox/Publi_Mosaique/DATA/DATA/GROUPED/phenotypage.csv", header = TRUE, sep=";")
colnames(BLUP)[1]="geno"
print("--- Your Phenotyping matrix looks correct. Dimension of the matrix are :")
```

```
## [1] "--- Your Phenotyping matrix looks correct. Dimension of the matrix are :"
```

```
print(dim(BLUP))
```

```
## [1] 396   6
```

```
# Fichier de phénotypage modifié, il va falloir mettre la Elisa de 2012 et 2015 ensemble, avec une colonne année.
a=BLUP[, c(1,3)] ; a$year="2012" ; colnames(a)=c("geno", "Elisa_blup_AR1","year") 
#a[,2]=a[,2]/sqrt( mean(c(0.66,0.83)) )
#a[,2]=ifelse(substr(a$geno, 1,2)=="TT", a[,2]/sqrt(0.66) , a[,2]/sqrt(0.83))
b=BLUP[, c(1,5)] ; b$year="2015" ; colnames(b)=c("geno", "Elisa_blup_AR1", "year") 
#b[,2]=b[,2]/sqrt( mean(c(1.2,1.21)) )
#b[,2]=ifelse(substr(b$geno, 1,2)=="TT", b[,2]/sqrt(1.2) , a[,2]/sqrt(1.21))
BLUP=rbind(a,b)
rownames(BLUP)=paste( BLUP[,1], BLUP$year,sep="_")
BLUP=BLUP[,-1]
# Note: On peut garder les blups tels quels / ou les pondéré par la variance génet de chaque année moyennée sur les 2 pops / ou par la variance génet de chaque année et chaque pop.
```

## Prepare data

We need to have genotype and phenotype in the same order.  
And I add a “pop” column in the phenotyping matrix:

```
Y=na.omit(BLUP)
Y=Y[which(rownames(Y)%in%rownames(genotype)) , ]
Y$pop=substr(rownames(Y),1,2)
genotype=genotype[which(rownames(genotype)%in%rownames(Y)) , ]
genotype=genotype[ match(rownames(Y),rownames(genotype)) , ]
```

# test1: DS - 2012

QTL detection without QTL rel for DS only in 2012 only

```
# Initialize the result table
result_lm_2012_DS=data.frame(matrix(0,0,3))
colnames(result_lm_2012_DS)=c("marker","pval-marker","R2")
num=0

# Run a loop on every markers
for(i in colnames(genotype)){
  
  # build data frame
  num=num+1
  don=cbind(Y, genotype[,which(colnames(genotype)%in%i)])
  colnames(don)[4]="allele"
  don=don[which(don$year=="2012") , ]
  don=don[which(don$pop=="TT") , ]
  don$allele=droplevels(don$allele)
  
  # Run the linear model
  if(nlevels(don$allele)==2){
    model=lm(don$Elisa_blup_AR1 ~ don$allele)
    res=anova(model)
  
  # Add result to the 'result' file
    result_lm_2012_DS[num,1:3]=c(i, res$`Pr(>F)`[1],summary(model)$r.squared)
  }
}

# wrong class
result_lm_2012_DS[,2:3]=apply(result_lm_2012_DS[,2:3] , 2 , as.numeric)
dim(result_lm_2012_DS)
```

```
## [1] 3544    3
```

We are supposed to find more or less the same result than with QTL-Rel. Is it true? The only difference is that we use lm and not QTL-Rel, thus we do not take into account the kinship matrix. Let’s check the manathan plot.

```
# Merge LODs with the genetic map
result_lm_2012_DS=merge(map,result_lm_2012_DS, by.x=1 , by.y=1, all.y=T)
result_lm_2012_DS=result_lm_2012_DS[order(result_lm_2012_DS$chr, result_lm_2012_DS$dist) , ]

# And plot it
plot(-log10(result_lm_2012_DS$`pval-marker`) , pch=20 , col=as.numeric(result_lm_2012_DS$chr) , cex=1.3, xaxt="n", ylab="LOD - scores" )
abline(h=3.6, col="grey", lwd=1.5)
num=seq(1,nrow(result_lm_2012_DS))
num=aggregate(num, by=list(result_lm_2012_DS$chr), mean , na.rm=T)
axis(1, at=num[,2], labels=num[,1])
```

//: =========================================================================================================================

# test2: DS - 2015

QTL detection without QTL rel for DS only in 2012 only

```
# Initialize the result table
result_lm_2015_DS=data.frame(matrix(0,0,3))
colnames(result_lm_2015_DS)=c("marker","pval-marker","R2")
num=0

# Run a loop on every markers
for(i in colnames(genotype)){
  
  # build data frame
  num=num+1
  don=cbind(Y, genotype[,which(colnames(genotype)%in%i)])
  colnames(don)[4]="allele"
  don=don[which(don$year=="2015") , ]
  don=don[which(don$pop=="TT") , ]
  don$allele=droplevels(don$allele)
  
  # Run the linear model
  if(nlevels(don$allele)==2){
    model=lm(don$Elisa_blup_AR1 ~ don$allele)
    res=anova(model)
  
  # Add result to the 'result' file
    result_lm_2015_DS[num,1:3]=c(i, res$`Pr(>F)`[1],summary(model)$r.squared)
  }
}

# wrong class
result_lm_2015_DS[,2:3]=apply(result_lm_2015_DS[,2:3] , 2 , as.numeric)
dim(result_lm_2015_DS)
```

```
## [1] 3544    3
```

We are supposed to find more or less the same result than with QTL-Rel. Is it true? The only difference is that we use lm and not QTL-Rel, thus we do not take into account the kinship matrix. Let’s check the manathan plot.

```
# Merge LODs with the genetic map
result_lm_2015_DS=merge(map,result_lm_2015_DS, by.x=1 , by.y=1, all.y=T)
result_lm_2015_DS=result_lm_2015_DS[order(result_lm_2015_DS$chr, result_lm_2015_DS$dist) , ]

# And plot it
plot(-log10(result_lm_2015_DS$`pval-marker`) , pch=20 , col=as.numeric(result_lm_2015_DS$chr) , cex=1.3, xaxt="n", ylab="LOD - scores" )
abline(h=3.6, col="grey", lwd=1.5)
num=seq(1,nrow(result_lm_2015_DS))
num=aggregate(num, by=list(result_lm_2015_DS$chr), mean , na.rm=T)
axis(1, at=num[,2], labels=num[,1])
```

//: =========================================================================================================================

# test3: DL - 2012

QTL detection without QTL rel for DL only in 2012 only

```
# Initialize the result table
result_lm_2012_DL=data.frame(matrix(0,0,3))
colnames(result_lm_2012_DL)=c("marker","pval-marker","R2")
num=0

# Run a loop on every markers
for(i in colnames(genotype)){
  
  # build data frame
  num=num+1
  don=cbind(Y, genotype[,which(colnames(genotype)%in%i)])
  colnames(don)[4]="allele"
  don=don[which(don$year=="2012") , ]
  don=don[which(don$pop=="BX") , ]
  don$allele=droplevels(don$allele)
  
  # Run the linear model
  if(nlevels(don$allele)==2){
    model=lm(don$Elisa_blup_AR1 ~ don$allele)
    res=anova(model)
  
  # Add result to the 'result' file
    result_lm_2012_DL[num,1:3]=c(i, res$`Pr(>F)`[1],summary(model)$r.squared)
  }
}

# wrong class
result_lm_2012_DL[,2:3]=apply(result_lm_2012_DL[,2:3] , 2 , as.numeric)
result_lm_2012_DL=na.omit(result_lm_2012_DL)
dim(result_lm_2012_DL)
```

```
## [1] 5851    3
```

We are supposed to find more or less the same result than with QTL-Rel. Is it true? The only difference is that we use lm and not QTL-Rel, thus we do not take into account the kinship matrix. Let’s check the manathan plot.

```
# Merge LOD with the genetic map
result_lm_2012_DL=merge(map,result_lm_2012_DL, by.x=1 , by.y=1, all.y=T)
result_lm_2012_DL=result_lm_2012_DL[order(result_lm_2012_DL$chr, result_lm_2012_DL$dist) , ]

# And plot it
plot(-log10(result_lm_2012_DL$`pval-marker`) , pch=20 , col=as.numeric(result_lm_2012_DL$chr) , cex=1.3, xaxt="n", ylab="LOD - scores" )
abline(h=3.6, col="grey", lwd=1.5)
num=seq(1,nrow(result_lm_2012_DL))
num=aggregate(num, by=list(result_lm_2012_DL$chr), mean , na.rm=T)
axis(1, at=num[,2], labels=num[,1])
```

//: =========================================================================================================================

# test4: DL - 2015

QTL detection without QTL rel for DL only in 2015 only

```
# Initialize the result table
result_lm_2015_DL=data.frame(matrix(0,0,3))
colnames(result_lm_2015_DL)=c("marker","pval-marker","R2")
num=0

# Run a loop on every markers
for(i in colnames(genotype)){
  
  # build data frame
  num=num+1
  don=cbind(Y, genotype[,which(colnames(genotype)%in%i)])
  colnames(don)[4]="allele"
  don=don[which(don$year=="2015") , ]
  don=don[which(don$pop=="BX") , ]
  don$allele=droplevels(don$allele)
  
  # Run the linear model
  if(nlevels(don$allele)==2){
    model=lm(don$Elisa_blup_AR1 ~ don$allele)
    res=anova(model)
  
  # Add result to the 'result' file
    result_lm_2015_DL[num,1:3]=c(i, res$`Pr(>F)`[1],summary(model)$r.squared)
  }
}

# wrong class
result_lm_2015_DL[,2:3]=apply(result_lm_2015_DL[,2:3] , 2 , as.numeric)
result_lm_2015_DL=na.omit(result_lm_2015_DL)
dim(result_lm_2015_DL)
```

```
## [1] 5851    3
```

We are supposed to find more or less the same result than with QTL-Rel. Is it true? The only difference is that we use lm and not QTL-Rel, thus we do not take into account the kinship matrix. Let’s check the manathan plot.

```
# Merge LOD with the genetic map
result_lm_2015_DL=merge(map,result_lm_2015_DL, by.x=1 , by.y=1, all.y=T)
result_lm_2015_DL=result_lm_2015_DL[order(result_lm_2015_DL$chr, result_lm_2015_DL$dist) , ]

# And plot it
plot(-log10(result_lm_2015_DL$`pval-marker`) , pch=20 , col=as.numeric(result_lm_2015_DL$chr) , cex=1.3, xaxt="n", ylab="LOD - scores" )
abline(h=3.6, col="grey", lwd=1.5)
num=seq(1,nrow(result_lm_2015_DL))
num=aggregate(num, by=list(result_lm_2015_DL$chr), mean , na.rm=T)
axis(1, at=num[,2], labels=num[,1])
```

# test5: DS AND DL - 2012

QTL detection without QTL rel for DS *and* DL in 2012 only

```
# Initialize the result table
result_lm_2012_DSDL=data.frame(matrix(0,0,4))
colnames(result_lm_2012_DSDL)=c("marker","pval-marker","pval-pop","R2")
num=0

# Run a loop on every markers
for(i in colnames(genotype)){
  
  # build data frame
  num=num+1
  don=cbind(Y, genotype[,which(colnames(genotype)%in%i)])
  colnames(don)[4]="allele"
  don=don[which(don$year=="2012") , ]
  don$allele=droplevels(don$allele)
  
  # Run the linear model
  if(nlevels(don$allele)==2){
    model=lm(don$Elisa_blup_AR1 ~ don$allele + don$pop)
    res=anova(model)
  
  # Add result to the 'result' file
    result_lm_2012_DSDL[num,1:4]=c(i, res$`Pr(>F)`[1:2],summary(model)$r.squared)
  }
}

# wrong class
result_lm_2012_DSDL[,2:4]=apply(result_lm_2012_DSDL[,2:4] , 2 , as.numeric)
dim(result_lm_2012_DSDL)
```

```
## [1] 7341    4
```

We are supposed to find more or less the same result than with QTL-Rel. Is it true? The only difference is that we use lm and not QTL-Rel, thus we do not take into account the kinship matrix. Let’s check the manathan plot.

```
# Merge LODs with the genetic map
result_lm_2012_DSDL=merge(map,result_lm_2012_DSDL, by.x=1 , by.y=1, all.y=T)
result_lm_2012_DSDL=result_lm_2012_DSDL[order(result_lm_2012_DSDL$chr, result_lm_2012_DSDL$dist) , ]

# And plot it
plot(-log10(result_lm_2012_DSDL$`pval-marker`) , pch=20 , col=as.numeric(result_lm_2012_DSDL$chr) , cex=1.3, xaxt="n", ylab="LOD - scores" )
abline(h=3.6, col="grey", lwd=1.5)
num=seq(1,nrow(result_lm_2012_DSDL))
num=aggregate(num, by=list(result_lm_2012_DSDL$chr), mean , na.rm=T)
axis(1, at=num[,2], labels=num[,1])
```

//: =========================================================================================================================

# test6: DS AND DL - 2015

QTL detection without QTL rel for DS *and* DL in 2015 only

```
# Initialize the result table
result_lm_2015_DSDL=data.frame(matrix(0,0,4))
colnames(result_lm_2015_DSDL)=c("marker","pval-marker","pval-pop","R2")
num=0

# Run a loop on every markers
for(i in colnames(genotype)){
  
  # build data frame
  num=num+1
  don=cbind(Y, genotype[,which(colnames(genotype)%in%i)])
  colnames(don)[4]="allele"
  don=don[which(don$year=="2015") , ]
  don$allele=droplevels(don$allele)
  
  # Run the linear model
  if(nlevels(don$allele)==2){
    model=lm(don$Elisa_blup_AR1 ~ don$allele + don$pop)
    res=anova(model)
  
  # Add result to the 'result' file
    result_lm_2015_DSDL[num,1:4]=c(i, res$`Pr(>F)`[1:2],summary(model)$r.squared)
  }
}

# wrong class
result_lm_2015_DSDL[,2:4]=apply(result_lm_2015_DSDL[,2:4] , 2 , as.numeric)
dim(result_lm_2015_DSDL)
```

```
## [1] 7341    4
```

We are supposed to find more or less the same result than with QTL-Rel. Is it true? The only difference is that we use lm and not QTL-Rel, thus we do not take into account the kinship matrix. Let’s check the manathan plot.

```
# Merge LODs with the genetic map
result_lm_2015_DSDL=merge(map,result_lm_2015_DSDL, by.x=1 , by.y=1, all.y=T)
result_lm_2015_DSDL=result_lm_2015_DSDL[order(result_lm_2015_DSDL$chr, result_lm_2015_DSDL$dist) , ]

# And plot it
plot(-log10(result_lm_2015_DSDL$`pval-marker`) , pch=20 , col=as.numeric(result_lm_2015_DSDL$chr) , cex=1.3, xaxt="n", ylab="LOD - scores" )
abline(h=3.6, col="grey", lwd=1.5)
num=seq(1,nrow(result_lm_2015_DSDL))
num=aggregate(num, by=list(result_lm_2015_DSDL$chr), mean , na.rm=T)
axis(1, at=num[,2], labels=num[,1])
```

//: =========================================================================================================================

# test7: DS - 2012 AND 2015

QTL detection without QTL rel for DS only in 2012 only

```
# Initialize the result table
result_lm_2012_2015_DS=data.frame(matrix(0,0,4))
colnames(result_lm_2012_2015_DS)=c("marker","pval-marker","pval-year","R2")
num=0

# Run a loop on every markers
for(i in colnames(genotype)){
  
  # build data frame
  num=num+1
  don=cbind(Y, genotype[,which(colnames(genotype)%in%i)])
  colnames(don)[4]="allele"
  don=don[which(don$pop=="TT") , ]
  don$allele=droplevels(don$allele)
  
  # Run the linear model
  if(nlevels(don$allele)==2){
    model=lm(don$Elisa_blup_AR1 ~ don$allele + don$year)
    res=anova(model)
  
  # Add result to the 'result' file
    result_lm_2012_2015_DS[num,1:4]=c(i, res$`Pr(>F)`[1:2],summary(model)$r.squared)
  }
}

# wrong class
result_lm_2012_2015_DS[,2:4]=apply(result_lm_2012_2015_DS[,2:4] , 2 , as.numeric)
dim(result_lm_2012_2015_DS)
```

```
## [1] 3544    4
```

We are supposed to find more or less the same result than with QTL-Rel. Is it true? The only difference is that we use lm and not QTL-Rel, thus we do not take into account the kinship matrix. Let’s check the manathan plot.

```
# Merge LODs with the genetic map
result_lm_2012_2015_DS=merge(map,result_lm_2012_2015_DS, by.x=1 , by.y=1, all.y=T)
result_lm_2012_2015_DS=result_lm_2012_2015_DS[order(result_lm_2012_2015_DS$chr, result_lm_2012_2015_DS$dist) , ]

# And plot it
plot(-log10(result_lm_2012_2015_DS$`pval-marker`) , pch=20 , col=as.numeric(result_lm_2012_2015_DS$chr) , cex=1.3, xaxt="n", ylab="LOD - scores" )
abline(h=3.6, col="grey", lwd=1.5)
num=seq(1,nrow(result_lm_2012_2015_DS))
num=aggregate(num, by=list(result_lm_2012_2015_DS$chr), mean , na.rm=T)
axis(1, at=num[,2], labels=num[,1])
```

//: =========================================================================================================================

# test8: DL - 2012 AND 2015

QTL detection without QTL rel for DL only in 2012 only

```
# Initialize the result table
result_lm_2012_2015_DL=data.frame(matrix(0,0,4))
colnames(result_lm_2012_2015_DL)=c("marker","pval-marker","pval-year","R2")
num=0

# Run a loop on every markers
for(i in colnames(genotype)){
  
  # build data frame
  num=num+1
  don=cbind(Y, genotype[,which(colnames(genotype)%in%i)])
  colnames(don)[4]="allele"
  don=don[which(don$pop=="BX") , ]
  don$allele=droplevels(don$allele)
  
  # Run the linear model
  if(nlevels(don$allele)==2){
    model=lm(don$Elisa_blup_AR1 ~ don$allele + don$year)
    res=anova(model)
  
  # Add result to the 'result' file
    result_lm_2012_2015_DL[num,1:4]=c(i, res$`Pr(>F)`[1:2],summary(model)$r.squared)
  }
}

# wrong class
result_lm_2012_2015_DL[,2:4]=apply(result_lm_2012_2015_DL[,2:4] , 2 , as.numeric)
result_lm_2012_2015_DL=na.omit(result_lm_2012_2015_DL)
dim(result_lm_2012_2015_DL)
```

```
## [1] 5851    4
```

We are supposed to find more or less the same result than with QTL-Rel. Is it true? The only difference is that we use lm and not QTL-Rel, thus we do not take into account the kinship matrix. Let’s check the manathan plot.

```
# Merge LODL with the genetic map
result_lm_2012_2015_DL=merge(map,result_lm_2012_2015_DL, by.x=1 , by.y=1, all.y=T)
result_lm_2012_2015_DL=result_lm_2012_2015_DL[order(result_lm_2012_2015_DL$chr, result_lm_2012_2015_DL$dist) , ]

# And plot it
plot(-log10(result_lm_2012_2015_DL$`pval-marker`) , pch=20 , col=as.numeric(result_lm_2012_2015_DL$chr) , cex=1.3, xaxt="n", ylab="LOD - scores" )
abline(h=3.6, col="grey", lwd=1.5)
num=seq(1,nrow(result_lm_2012_2015_DL))
num=aggregate(num, by=list(result_lm_2012_2015_DL$chr), mean , na.rm=T)
axis(1, at=num[,2], labels=num[,1])
```

//: =========================================================================================================================

# test9: DS AND DL - 2012 AND 2015

QTL detection without QTL rel for *DS and DL* in *2012 and 2015*

```
# Initialize the result table
result_lm_2012_2015_DSDL=data.frame(matrix(0,0,5))
colnames(result_lm_2012_2015_DSDL)=c("marker","pval-marker","pval-year","pval-pop","R2")
num=0

# Run a loop on every markers
for(i in colnames(genotype)){
  
  # build data frame
  num=num+1
  don=cbind(Y, genotype[,which(colnames(genotype)%in%i)])
  colnames(don)[4]="allele"
  don$allele=droplevels(don$allele)
  
  # Run the linear model
  if(nlevels(don$allele)==2){
    model=lm(don$Elisa_blup_AR1 ~ don$allele + don$year +don$pop)
    res=anova(model)
  
  # Add result to the 'result' file
    result_lm_2012_2015_DSDL[num,1:5]=c(i, res$`Pr(>F)`[1:3],summary(model)$r.squared)
  }
}

# wrong class
result_lm_2012_2015_DSDL[,2:5]=apply(result_lm_2012_2015_DSDL[,2:5] , 2 , as.numeric)
result_lm_2012_2015_DSDL=na.omit(result_lm_2012_2015_DSDL)
dim(result_lm_2012_2015_DSDL)
```

```
## [1] 7341    5
```

We are supposed to find more or less the same result than with QTL-Rel. Is it true? The only difference is that we use lm and not QTL-Rel, thus we do not take into account the kinship matrix. Let’s check the manathan plot.

```
# Merge LODL with the genetic map
result_lm_2012_2015_DSDL=merge(map,result_lm_2012_2015_DSDL, by.x=1 , by.y=1, all.y=T)
result_lm_2012_2015_DSDL=result_lm_2012_2015_DSDL[order(result_lm_2012_2015_DSDL$chr, result_lm_2012_2015_DSDL$dist) , ]

# And plot it
plot(-log10(result_lm_2012_2015_DSDL$`pval-marker`) , pch=20 , col=as.numeric(result_lm_2012_2015_DSDL$chr) , cex=1.3, xaxt="n", ylab="LOD - scores" )
abline(h=3.6, col="grey", lwd=1.5)
num=seq(1,nrow(result_lm_2012_2015_DSDL))
num=aggregate(num, by=list(result_lm_2012_2015_DSDL$chr), mean , na.rm=T)
axis(1, at=num[,2], labels=num[,1])
```

//: =========================================================================================================================

# test10: DS AND DL - 2012 AND 2015 - INTERACTION

QTL detection without QTL rel for *DS and DL* in *2012 and 2015*. And I add interaction between marker and year

```
# Initialize the result table
result_lm_2012_2015_DSDL_inter=data.frame(matrix(0,0,6))
colnames(result_lm_2012_2015_DSDL_inter)=c("marker","pval-marker","pval-year","pval-pop","pval-inter","R2")
num=0

# Run a loop on every markers
for(i in colnames(genotype)){
  
  # build data frame
  num=num+1
  don=cbind(Y, genotype[,which(colnames(genotype)%in%i)])
  colnames(don)[4]="allele"
  don$allele=droplevels(don$allele)
  
  # Run the linear model
  if(nlevels(don$allele)==2){
    model=lm(don$Elisa_blup_AR1 ~ don$allele + don$year +don$pop + don$allele*don$year)
    res=anova(model)
  
  # Add result to the 'result' file
    result_lm_2012_2015_DSDL_inter[num,1:6]=c(i, res$`Pr(>F)`[1:4],summary(model)$r.squared)
  }
}

# wrong class
result_lm_2012_2015_DSDL_inter[,2:6]=apply(result_lm_2012_2015_DSDL_inter[,2:6] , 2 , as.numeric)
result_lm_2012_2015_DSDL_inter=na.omit(result_lm_2012_2015_DSDL_inter)
dim(result_lm_2012_2015_DSDL_inter)
```

```
## [1] 7341    6
```

We are supposed to find more or less the same result than with QTL-Rel. Is it true? The only difference is that we use lm and not QTL-Rel, thus we do not take into account the kinship matrix. Let’s check the manathan plot.

```
# Merge LODL with the genetic map
result_lm_2012_2015_DSDL_inter=merge(map,result_lm_2012_2015_DSDL_inter, by.x=1 , by.y=1, all.y=T)
result_lm_2012_2015_DSDL_inter=result_lm_2012_2015_DSDL_inter[order(result_lm_2012_2015_DSDL_inter$chr, result_lm_2012_2015_DSDL_inter$dist) , ]

# And plot it
plot(-log10(result_lm_2012_2015_DSDL_inter$`pval-marker`) , pch=20 , col=as.numeric(result_lm_2012_2015_DSDL_inter$chr) , cex=1.3, xaxt="n", ylab="LOD - scores" )
abline(h=3.6, col="grey", lwd=1.5)
num=seq(1,nrow(result_lm_2012_2015_DSDL_inter))
num=aggregate(num, by=list(result_lm_2012_2015_DSDL_inter$chr), mean , na.rm=T)
axis(1, at=num[,2], labels=num[,1])
```

//: =========================================================================================================================

# RECAP

```
# Function to plot
manat_plot=function(data, my_xlab){
  plot(-log10(data$`pval-marker`) , pch=20 , col=as.numeric(data$chr) , cex=1.3, xaxt="n", ylab="LOD - scores" , xlab=my_xlab)
  abline(h=3.6, col="grey", lwd=1.5)
  num=seq(1,nrow(data))
  num=aggregate(num, by=list(data$chr), mean , na.rm=T)
  axis(1, at=num[,2], labels=num[,1])
}
```

## Detection by year and pop separately

```
par(mfrow=c(2,2))
manat_plot(result_lm_2012_DS, "DS 2012")
manat_plot(result_lm_2012_DL, "DL 2012")
manat_plot(result_lm_2015_DS, "DS 2015")
manat_plot(result_lm_2015_DL, "DL 2015")
```

## Grouping population

```
par(mfrow=c(2,1))
manat_plot(result_lm_2012_DSDL, "DS & DL in 2012")
manat_plot(result_lm_2015_DSDL, "DS & DL in 2015")
```

## Grouping Years

```
par(mfrow=c(1,2))
manat_plot(result_lm_2012_2015_DS, "DS in 2012 + 2015")
manat_plot(result_lm_2012_2015_DL, "DL in 2012 + 2015")
```

## Grouping Years AND pops

Without interaction

```
par(mfrow=c(1,1))
manat_plot(result_lm_2012_2015_DSDL, "DS +DL in 2012 + 2015")
```

With interaction

```
par(mfrow=c(1,1))
manat_plot(result_lm_2012_2015_DSDL_inter, "DS +DL in 2012 + 2015 AND Interaction")
```

Yan Holtz

December 2016
