# Supplementary material for: Epistatic determinism of durum wheat resistance to the wheat spindle streak mosaic virus
Source: Theor Appl Genet. 2017 Apr 27;130(7):1491–505. doi: 10.1007/s00122-017-2904-6 (PMC5487696; doi:10.1007/s00122-017-2904-6)
Supplement: Supplementary file 4 — Online Resource 4: Data and R scripts for reproducible QTL detection. Data and R script (.csv and.rmd format) are provided in this tar archive. A scheme aims to explain the content of each file and its role in the QTL detection pipeline. The upstream bioinformatic steps (from raw reads to consensus genetic map) are not included (GZ 72829 kb) [file 122_2017_2904_MOESM4_ESM.gz › TMP/SCRIPT/4_Answer_Review/Interaction_QTL_Year/Interaction_QTL_Year_QTLRel_Elisa.html]

Interaction QTL x Year with QTLRel | Elisa


# Interaction QTL x Year with QTLRel | Elisa

## Interaction QTL x Year with QTLRel | Elisa

- Introduction
  - Load data
  - Prepare data
  - Impute missing data
- test1: DS - 2012
- test2: DS - 2015
- test3: DL - 2012
- test4: DL - 2015
- test5: DS AND DL - 2012
- test6: DS AND DL - 2015
- test7: DS - 2012 & 2015
- test8: DL - 2012 & 2015
- test9: DS AND DL - 2012 & 2015
- test10: DS AND DL - 2012 & 2015 - interaction effect

This file aims to provide more details concerning the QTL x Year interaction of the resistance of Durum Wheat to WSSMV. We are going to study ELISA. We will use QTLRel

```
library(QTLRel)
```

```
## R/QTLRel is loaded
```

```
library(xtable)
```

# Introduction

We are going to study the effects of markers trough years and pop progressively.

## Load data

I load the genotyping matrix first.

```
genotype<-read.table("/Users/holtz/Dropbox/Publi_Mosaique/DATA/DATA/GROUPED/genotypage.csv", sep = ";" , header = F, na.strings = "-")
genotype=as.matrix(genotype)
colnames(genotype)=genotype[1,]
genotype=as.data.frame(genotype[-1 , ])
names(genotype)[1]<-"geno"
print("--- Your genotyping matrix looks correct. Dimension of the matrix are :")
```

```
## [1] "--- Your genotyping matrix looks correct. Dimension of the matrix are :"
```

```
print(dim(genotype))
```

```
## [1]  348 7342
```

```
# I copy this matrix 2 times, since I read 2012 and 2015 together.
rownames(genotype)=genotype[,1]
genotype=genotype[,-1]
a=genotype ; rownames(a)=paste(rownames(a),"2012",sep="_")
b=genotype ; rownames(b)=paste(rownames(b),"2015",sep="_")
genotype=rbind(a,b)
```

Then the genetic map:

```
map <- read.table("/Users/holtz/Dropbox/Publi_Mosaique/DATA/DATA/genetic_map.txt" , header=T , dec = ".", na.strings = "-" , check.names=F)
colnames(map) <- c("LG", "marqueur", "Distance","group_physique","Posi_physique")
rownames(map) <- map$marqueur
map$LG <- as.factor(map$LG)
print("--- Your genetic map looks correct. Dimension of the map are :")
```

```
## [1] "--- Your genetic map looks correct. Dimension of the map are :"
```

```
print(dim(map))
```

```
## [1] 8568    5
```

```
map=map[    , c(2,1,3,5)]
colnames(map)=c("snp","chr", "dist", "phyPos")
```

And finally the phenotyping matrix

```
BLUP<-read.table("/Users/holtz/Dropbox/Publi_Mosaique/DATA/DATA/GROUPED/phenotypage.csv", header = TRUE, sep=";")
colnames(BLUP)[1]="geno"
print("--- Your Phenotyping matrix looks correct. Dimension of the matrix are :")
```

```
## [1] "--- Your Phenotyping matrix looks correct. Dimension of the matrix are :"
```

```
print(dim(BLUP))
```

```
## [1] 396   6
```

```
# Fichier de phénotypage modifié, il va falloir mettre la Elisa de 2012 et 2015 ensemble, avec une colonne année.
a=BLUP[, c(1,3)] ; a$year="2012" ; colnames(a)=c("geno", "Elisa_blup_AR1","year") 
#a[,2]=a[,2]/sqrt( mean(c(0.66,0.83)) )
#a[,2]=ifelse(substr(a$geno, 1,2)=="TT", a[,2]/sqrt(0.66) , a[,2]/sqrt(0.83))
b=BLUP[, c(1,5)] ; b$year="2015" ; colnames(b)=c("geno", "Elisa_blup_AR1", "year") 
#b[,2]=b[,2]/sqrt( mean(c(1.2,1.21)) )
#b[,2]=ifelse(substr(b$geno, 1,2)=="TT", b[,2]/sqrt(1.2) , a[,2]/sqrt(1.21))
BLUP=rbind(a,b)
rownames(BLUP)=paste( BLUP[,1], BLUP$year,sep="_")
BLUP=BLUP[,-1]
# Note: On peut garder les blups tels quels / ou les pondéré par la variance génet de chaque année moyennée sur les 2 pops / ou par la variance génet de chaque année et chaque pop.
```

## Prepare data

We need to have genotype and phenotype in the same order.  
And I add a “pop” column in the phenotyping matrix:

```
Y=na.omit(BLUP)
Y=Y[which(rownames(Y)%in%rownames(genotype)) , ]
Y$pop=substr(rownames(Y),1,2)
genotype=genotype[which(rownames(genotype)%in%rownames(Y)) , ]
genotype=genotype[ match(rownames(Y),rownames(genotype)) , ]
```

## Impute missing data

```
# missing data
set.seed(123)
XNNA=genotype
my_fun=function(x){length(x[x=="A" & !is.na(x) ])/length(!is.na(x)) }
prop=apply(XNNA , 2 , my_fun)
for(i in c(1:ncol(XNNA))){
        aa=XNNA[,i][is.na(XNNA[,i])]
        bb=rbinom(length(aa),1,prob=prop[i])
        XNNA[,i][is.na(XNNA[,i])]=c("B","A")[bb+1]
        }
        
# Change "A" to "AA"
XNNA=as.matrix(XNNA)
XNNA[which(XNNA=="A")]<-"AA"
XNNA[which(XNNA=="B")]<-"BB"
```

# test1: DS - 2012

QTL detection with QTL rel for DS only in 2012 only

```
# Select the corresponding phenotype data
Y_tmp=Y[which(Y$pop=="TT" & Y$year=="2012") , ]

# Select the corresponding genotyping data?
XNNA_tmp=XNNA[ which (substr(rownames(XNNA),1,2)=="TT" & grepl("2012" , rownames(XNNA))) , ]
XNNA_tmp=XNNA_tmp [ , which(apply(XNNA_tmp , 2 , function(x){length(unique(x))} )==2) ]
dim(XNNA_tmp)
```

```
## [1]  161 3544
```

```
# Kinship matrix
K<-genMatrix(XNNA_tmp)
        
# I = identity matrix
I<-diag(nrow(Y_tmp))

# Variance components
mod1<-estVC(y=Y_tmp$Elisa_blup_AR1, v=list(AA=K$AA,DD=NULL,HH=NULL,AD=NULL,MH=NULL,EE=I))

# Test marker per marker
llk.hk1 <- scanOne(y=Y_tmp$Elisa_blup_AR1, vc=mod1, gdat=XNNA_tmp ,test="Chisq")
```

Let’s observe results.

```
# Merge LODs with the genetic map
AA=merge(map,data.frame( names(llk.hk1$p) , llk.hk1$p), by.x=1 , by.y=1, all.x=T)
AA=AA[order(AA$chr, AA$dist) , ]
AA=AA[!is.na(AA$llk.hk1.p) , ]
# And plot it
plot(-log10(AA$llk.hk1.p) , pch=20 , col=as.numeric(AA$chr) , cex=1.3, xaxt="n" )
abline(h=3.6, col="grey", lwd=1.5)
num=seq(1,nrow(AA))
num=aggregate(num, by=list(AA$chr), mean , na.rm=T)
axis(1, at=num[,2], labels=num[,1])
```

```
# Save as the object
res_DS_2012=AA
```

# test2: DS - 2015

QTL detection with QTL rel for DS only in 2012 only

```
# Select the corresponding phenotype data
Y_tmp=Y[which(Y$pop=="TT" & Y$year=="2015") , ]

# Select the corresponding genotyping data?
XNNA_tmp=XNNA[ which (substr(rownames(XNNA),1,2)=="TT" & grepl("2015" , rownames(XNNA))) , ]
XNNA_tmp=XNNA_tmp [ , which(apply(XNNA_tmp , 2 , function(x){length(unique(x))} )==2) ]
dim(XNNA_tmp)
```

```
## [1]  161 3544
```

```
# Kinship matrix
K<-genMatrix(XNNA_tmp)
        
# I = identity matrix
I<-diag(nrow(Y_tmp))

# Variance components
mod1<-estVC(y=Y_tmp$Elisa_blup_AR1, v=list(AA=K$AA,DD=NULL,HH=NULL,AD=NULL,MH=NULL,EE=I))

# Test marker per marker
llk.hk1 <- scanOne(y=Y_tmp$Elisa_blup_AR1, vc=mod1, gdat=XNNA_tmp ,test="Chisq")
```

Let’s observe results.

```
# Merge LODs with the genetic map
AA=merge(map,data.frame( names(llk.hk1$p) , llk.hk1$p), by.x=1 , by.y=1, all.x=T)
AA=AA[order(AA$chr, AA$dist) , ]
AA=AA[!is.na(AA$llk.hk1.p) , ]
# And plot it
plot(-log10(AA$llk.hk1.p) , pch=20 , col=as.numeric(AA$chr) , cex=1.3, xaxt="n" )
abline(h=3.6, col="grey", lwd=1.5)
num=seq(1,nrow(AA))
num=aggregate(num, by=list(AA$chr), mean , na.rm=T)
axis(1, at=num[,2], labels=num[,1])
```

```
# Save as the object
res_DS_2015=AA
```

# test3: DL - 2012

QTL detection with QTL rel for DS only in 2012 only

```
# Select the corresponding phenotype data
Y_tmp=Y[which(Y$pop=="BX" & Y$year=="2012") , ]

# Select the corresponding genotyping data?
XNNA_tmp=XNNA[ which (substr(rownames(XNNA),1,2)=="BX" & grepl("2012" , rownames(XNNA))) , ]
XNNA_tmp=XNNA_tmp [ , which(apply(XNNA_tmp , 2 , function(x){length(unique(x))} )==2) ]
dim(XNNA_tmp)
```

```
## [1]  181 5851
```

```
# Kinship matrix
K<-genMatrix(XNNA_tmp)
        
# I = identity matrix
I<-diag(nrow(Y_tmp))

# Variance components
mod1<-estVC(y=Y_tmp$Elisa_blup_AR1, v=list(AA=K$AA,DD=NULL,HH=NULL,AD=NULL,MH=NULL,EE=I))

# Test marker per marker
llk.hk1 <- scanOne(y=Y_tmp$Elisa_blup_AR1, vc=mod1, gdat=XNNA_tmp ,test="Chisq")
```

Let’s observe results.

```
# Merge LODs with the genetic map
AA=merge(map,data.frame( names(llk.hk1$p) , llk.hk1$p), by.x=1 , by.y=1, all.x=T)
AA=AA[order(AA$chr, AA$dist) , ]
AA=AA[!is.na(AA$llk.hk1.p) , ]
# And plot it
plot(-log10(AA$llk.hk1.p) , pch=20 , col=as.numeric(AA$chr) , cex=1.3, xaxt="n" )
abline(h=3.6, col="grey", lwd=1.5)
num=seq(1,nrow(AA))
num=aggregate(num, by=list(AA$chr), mean , na.rm=T)
axis(1, at=num[,2], labels=num[,1])
```

```
# Save as the object
res_DL_2012=AA
```

# test4: DL - 2015

QTL detection with QTL rel for DS only in 2012 only

```
# Select the corresponding phenotype data
Y_tmp=Y[which(Y$pop=="BX" & Y$year=="2015") , ]

# Select the corresponding genotyping data?
XNNA_tmp=XNNA[ which (substr(rownames(XNNA),1,2)=="BX" & grepl("2015" , rownames(XNNA))) , ]
XNNA_tmp=XNNA_tmp [ , which(apply(XNNA_tmp , 2 , function(x){length(unique(x))} )==2) ]
dim(XNNA_tmp)
```

```
## [1]  185 5851
```

```
# Kinship matrix
K<-genMatrix(XNNA_tmp)
        
# I = identity matrix
I<-diag(nrow(Y_tmp))

# Variance components
mod1<-estVC(y=Y_tmp$Elisa_blup_AR1, v=list(AA=K$AA,DD=NULL,HH=NULL,AD=NULL,MH=NULL,EE=I))

# Test marker per marker
llk.hk1 <- scanOne(y=Y_tmp$Elisa_blup_AR1, vc=mod1, gdat=XNNA_tmp ,test="Chisq")
```

Let’s observe results.

```
# Merge LODs with the genetic map
AA=merge(map,data.frame( names(llk.hk1$p) , llk.hk1$p), by.x=1 , by.y=1, all.x=T)
AA=AA[order(AA$chr, AA$dist) , ]
AA=AA[!is.na(AA$llk.hk1.p) , ]
# And plot it
plot(-log10(AA$llk.hk1.p) , pch=20 , col=as.numeric(AA$chr) , cex=1.3, xaxt="n" )
abline(h=3.6, col="grey", lwd=1.5)
num=seq(1,nrow(AA))
num=aggregate(num, by=list(AA$chr), mean , na.rm=T)
axis(1, at=num[,2], labels=num[,1])
```

```
# Save as the object
res_DL_2015=AA
```

# test5: DS AND DL - 2012

QTL detection with QTL rel for DS only in 2012 only

```
# Select the corresponding phenotype data
Y_tmp=Y[which( Y$year=="2012") , ]

# Select the corresponding genotyping data?
XNNA_tmp=XNNA[ which ( grepl("2012" , rownames(XNNA))) , ]
XNNA_tmp=XNNA_tmp [ , which(apply(XNNA_tmp , 2 , function(x){length(unique(x))} )==2) ]
dim(XNNA_tmp)
```

```
## [1]  342 7341
```

```
# Kinship matrix
K<-genMatrix(XNNA_tmp)
        
# I = identity matrix
I<-diag(nrow(Y_tmp))

# Variance components
mod1<-estVC(y=Y_tmp$Elisa_blup_AR1,  x=Y_tmp$pop, v=list(AA=K$AA,DD=NULL,HH=NULL,AD=NULL,MH=NULL,EE=I))

# Test marker per marker
llk.hk1 <- scanOne(y=Y_tmp$Elisa_blup_AR1, x=Y_tmp$pop, vc=mod1, gdat=XNNA_tmp ,test="Chisq")
```

Let’s observe results.

```
# Merge LODs with the genetic map
AA=merge(map,data.frame( names(llk.hk1$p) , llk.hk1$p), by.x=1 , by.y=1, all.x=T)
AA=AA[order(AA$chr, AA$dist) , ]
AA=AA[!is.na(AA$llk.hk1.p) , ]
# And plot it
plot(-log10(AA$llk.hk1.p) , pch=20 , col=as.numeric(AA$chr) , cex=1.3, xaxt="n" )
abline(h=3.6, col="grey", lwd=1.5)
num=seq(1,nrow(AA))
num=aggregate(num, by=list(AA$chr), mean , na.rm=T)
axis(1, at=num[,2], labels=num[,1])
```

```
# Save as the object
res_DSDL_2012=AA
```

# test6: DS AND DL - 2015

QTL detection with QTL rel for DS only in 2012 only

```
# Select the corresponding phenotype data
Y_tmp=Y[which( Y$year=="2015") , ]

# Select the corresponding genotyping data?
XNNA_tmp=XNNA[ which ( grepl("2015" , rownames(XNNA))) , ]
XNNA_tmp=XNNA_tmp [ , which(apply(XNNA_tmp , 2 , function(x){length(unique(x))} )==2) ]
dim(XNNA_tmp)
```

```
## [1]  346 7341
```

```
# Kinship matrix
K<-genMatrix(XNNA_tmp)
        
# I = identity matrix
I<-diag(nrow(Y_tmp))

# Variance components
mod1<-estVC(y=Y_tmp$Elisa_blup_AR1, x=Y_tmp$pop, v=list(AA=K$AA,DD=NULL,HH=NULL,AD=NULL,MH=NULL,EE=I))

# Test marker per marker
llk.hk1 <- scanOne(y=Y_tmp$Elisa_blup_AR1, x=Y_tmp$pop,  vc=mod1, gdat=XNNA_tmp ,test="Chisq")
```

Let’s observe results.

```
# Merge LODs with the genetic map
AA=merge(map,data.frame( names(llk.hk1$p) , llk.hk1$p), by.x=1 , by.y=1, all.x=T)
AA=AA[order(AA$chr, AA$dist) , ]
AA=AA[!is.na(AA$llk.hk1.p) , ]
# And plot it
plot(-log10(AA$llk.hk1.p) , pch=20 , col=as.numeric(AA$chr) , cex=1.3, xaxt="n" )
abline(h=3.6, col="grey", lwd=1.5)
num=seq(1,nrow(AA))
num=aggregate(num, by=list(AA$chr), mean , na.rm=T)
axis(1, at=num[,2], labels=num[,1])
```

```
# Save as the object
res_DSDL_2012=AA
```

# test7: DS - 2012 & 2015

QTL detection with QTL rel for DS only in 2012 only

```
# Select the corresponding phenotype data
Y_tmp=Y[which(Y$pop=="TT" ) , ]

# Select the corresponding genotyping data?
XNNA_tmp=XNNA[ which (substr(rownames(XNNA),1,2)=="TT" ) , ]
XNNA_tmp=XNNA_tmp [ , which(apply(XNNA_tmp , 2 , function(x){length(unique(x))} )==2) ]
dim(XNNA_tmp)
```

```
## [1]  322 3544
```

```
# Kinship matrix
K<-genMatrix(XNNA_tmp)
        
# I = identity matrix
I<-diag(nrow(Y_tmp))

# Variance components
mod1<-estVC(y=Y_tmp$Elisa_blup_AR1, x=Y_tmp$year, v=list(AA=K$AA,DD=NULL,HH=NULL,AD=NULL,MH=NULL,EE=I))

# Test marker per marker
llk.hk1 <- scanOne(y=Y_tmp$Elisa_blup_AR1, x=Y_tmp$year, vc=mod1, gdat=XNNA_tmp ,test="Chisq")
```

Let’s observe results.

```
# Merge LODs with the genetic map
AA=merge(map,data.frame( names(llk.hk1$p) , llk.hk1$p), by.x=1 , by.y=1, all.x=T)
AA=AA[order(AA$chr, AA$dist) , ]
AA=AA[!is.na(AA$llk.hk1.p) , ]
# And plot it
plot(-log10(AA$llk.hk1.p) , pch=20 , col=as.numeric(AA$chr) , cex=1.3, xaxt="n" )
abline(h=3.6, col="grey", lwd=1.5)
num=seq(1,nrow(AA))
num=aggregate(num, by=list(AA$chr), mean , na.rm=T)
axis(1, at=num[,2], labels=num[,1])
```

```
# Save as the object
res_DS_2012_2015=AA
```

# test8: DL - 2012 & 2015

QTL detection with QTL rel for DS only in 2012 only

```
# Select the corresponding phenotype data
Y_tmp=Y[which(Y$pop=="BX" ) , ]

# Select the corresponding genotyping data?
XNNA_tmp=XNNA[ which (substr(rownames(XNNA),1,2)=="BX" ) , ]
XNNA_tmp=XNNA_tmp [ , which(apply(XNNA_tmp , 2 , function(x){length(unique(x))} )==2) ]
dim(XNNA_tmp)
```

```
## [1]  366 5851
```

```
# Kinship matrix
K<-genMatrix(XNNA_tmp)
        
# I = identity matrix
I<-diag(nrow(Y_tmp))

# Variance components
mod1<-estVC(y=Y_tmp$Elisa_blup_AR1, x=Y_tmp$year, v=list(AA=K$AA,DD=NULL,HH=NULL,AD=NULL,MH=NULL,EE=I))

# Test marker per marker
llk.hk1 <- scanOne(y=Y_tmp$Elisa_blup_AR1, x=Y_tmp$year, vc=mod1, gdat=XNNA_tmp ,test="Chisq")
```

Let’s observe results.

```
# Merge LODs with the genetic map
AA=merge(map,data.frame( names(llk.hk1$p) , llk.hk1$p), by.x=1 , by.y=1, all.x=T)
AA=AA[order(AA$chr, AA$dist) , ]
AA=AA[!is.na(AA$llk.hk1.p) , ]
# And plot it
plot(-log10(AA$llk.hk1.p) , pch=20 , col=as.numeric(AA$chr) , cex=1.3, xaxt="n" )
abline(h=3.6, col="grey", lwd=1.5)
num=seq(1,nrow(AA))
num=aggregate(num, by=list(AA$chr), mean , na.rm=T)
axis(1, at=num[,2], labels=num[,1])
```

```
# Save as the object
res_DL_2012_2015=AA
```

# test9: DS AND DL - 2012 & 2015

QTL detection with QTL rel for DS only in 2012 only

```
# Select the corresponding phenotype data
Y_tmp=Y

# Select the corresponding genotyping data?
XNNA_tmp=XNNA
XNNA_tmp=XNNA_tmp [ , which(apply(XNNA_tmp , 2 , function(x){length(unique(x))} )==2) ]
dim(XNNA_tmp)
```

```
## [1]  688 7341
```

```
# Kinship matrix
K<-genMatrix(XNNA_tmp)
        
# I = identity matrix
I<-diag(nrow(Y_tmp))

# Variance components
mod1<-estVC(y=Y_tmp$Elisa_blup_AR1, x=Y_tmp$year, v=list(AA=K$AA,DD=NULL,HH=NULL,AD=NULL,MH=NULL,EE=I))

# Test marker per marker
llk.hk1 <- scanOne(y=Y_tmp$Elisa_blup_AR1, x=Y_tmp$year, vc=mod1, gdat=XNNA_tmp ,test="Chisq")
```

Let’s observe results.

```
# Merge LODs with the genetic map
AA=merge(map,data.frame( names(llk.hk1$p) , llk.hk1$p), by.x=1 , by.y=1, all.x=T)
AA=AA[order(AA$chr, AA$dist) , ]
AA=AA[!is.na(AA$llk.hk1.p) , ]
# And plot it
plot(-log10(AA$llk.hk1.p) , pch=20 , col=as.numeric(AA$chr) , cex=1.3, xaxt="n" )
abline(h=3.6, col="grey", lwd=1.5)
num=seq(1,nrow(AA))
num=aggregate(num, by=list(AA$chr), mean , na.rm=T)
axis(1, at=num[,2], labels=num[,1])
```

```
# Save as the object
res_DSDL_2012_2015=AA
```

More info on the 2 weird significant QTLs

```
bilan<-data.frame(marqueurs=names(llk.hk1$p),LOD=-log10(llk.hk1$p), r2=llk.hk1$v)
bilan=merge(map,bilan, by.x=1 , by.y=1, all.y=T)
bilan=bilan[order(bilan$chr, bilan$dist) , ]

a=bilan[which(bilan$chr=="4B") , ]
a=a[which(a$LOD==max(a$LOD, na.rm=T)) , ]
b=bilan[which(bilan$chr=="3A") , ]
b=b[which(b$LOD==max(b$LOD, na.rm=T)) , ]
bilan=rbind(a,b)
head(bilan)
```

```
##                                    snp chr  dist    phyPos      LOD
## 1573 Cluster_156|Contig1|original@1139  4B 125.9 286159851 5.736851
## 2363 Cluster_2107|Contig1|original@272  4B 125.9 265310050 5.736851
## 4985  Cluster_619|Contig1|original@832  3A 201.2 172452668 4.099960
##            r2
## 1573 3.216066
## 2363 3.216066
## 4985 2.222644
```

Distortion de ségrégation?

```
tmp=data.frame(all=genotype[ , "Cluster_2107|Contig1|original@272"], pop=substr(rownames(genotype), 1 , 2))
table( tmp )
```

```
##    pop
## all  BX  TT
##   A 366 194
##   B   0 126
```

```
tmp=data.frame(all=genotype[ , "Cluster_619|Contig1|original@832"], pop=substr(rownames(genotype), 1 , 2))
table( tmp )
```

```
##    pop
## all  BX  TT
##   A 185 322
##   B 172   0
```

Donc les 2 marqueurs qui créent ces QTLs innatendus sont en fait présent chez une seule des 2 pops! Donc c’est juste un artéfact! //: =========================================================================================================================

# test10: DS AND DL - 2012 & 2015 - interaction effect

QTL detection with QTL rel for DS only in 2012 only

```
# Beginning is the same as test 9

# Test marker per marker with and without interaction
llk.hk1 <- scanOne(y=Y_tmp$Elisa_blup_AR1, x=Y_tmp$year, vc=mod1, gdat=XNNA_tmp ,test="None")
llk.hk2 <- scanOne(y=Y_tmp$Elisa_blup_AR1, x=Y_tmp$year, intcovar=Y$year, vc=mod1, gdat=XNNA_tmp ,test="None")

# Find pvalue of each marker for interaction significance
diff_deviance <- llk.hk2$p - llk.hk1$p
pvaluesexpected=1-pchisq(diff_deviance, 1)
```

Let’s observe results.

```
# Merge LODs with the genetic map
AA=merge(map,data.frame( names(pvaluesexpected) , pvaluesexpected), by.x=1 , by.y=1, all.x=T)
AA=AA[order(AA$chr, AA$dist) , ]
AA=AA[!is.na(AA$pvaluesexpected) , ]
# And plot it
plot(-log10(AA$pvaluesexpected) , pch=20 , col=as.numeric(AA$chr) , cex=1.3, xaxt="n" , ylim=c(0,4) )
abline(h=-log10(0.05), col="grey", lwd=1.5)
abline(h=3.61, col="grey", lwd=1.5)
num=seq(1,nrow(AA))
num=aggregate(num, by=list(AA$chr), mean , na.rm=T)
axis(1, at=num[,2], labels=num[,1])
```

```
# Save as the object
res_DSDL_2012_2015_inter=AA
```

Show the 3 interaction effects (marker details)

```
a=res_DSDL_2012_2015_inter[which(res_DSDL_2012_2015_inter$chr=="2A") , ]
a=a[a$pvaluesexpected==min(a$pvaluesexpected) , ]
b=res_DSDL_2012_2015_inter[which(res_DSDL_2012_2015_inter$chr=="7A") , ]
b=b[b$pvaluesexpected==min(b$pvaluesexpected) , ]
c=res_DSDL_2012_2015_inter[which(res_DSDL_2012_2015_inter$chr=="7B") , ]
c=c[c$pvaluesexpected==min(c$pvaluesexpected) , ]
signif_inter=rbind(a,b,c)
```

|  | snp | chr | dist | phyPos | pvaluesexpected |
| --- | --- | --- | --- | --- | --- |
| 1526 | Cluster\_14091|Contig2|original@276 | 2A | 218.80 | 240000045 | 0.00 |
| 3478 | Cluster\_2740|Contig1|complementarySeq@2743 | 7A | 120.00 | 51363712 | 0.00 |
| 7586 | Cluster\_8668|Contig2|original@192 | 7B | 54.40 | 45672329 | 0.00 |

Yan Holtz

December 2016
