# Supplementary material for: Epistatic determinism of durum wheat resistance to the wheat spindle streak mosaic virus
Source: Theor Appl Genet. 2017 Apr 27;130(7):1491–505. doi: 10.1007/s00122-017-2904-6 (PMC5487696; doi:10.1007/s00122-017-2904-6)
Supplement: Supplementary file 4 — Online Resource 4: Data and R scripts for reproducible QTL detection. Data and R script (.csv and.rmd format) are provided in this tar archive. A scheme aims to explain the content of each file and its role in the QTL detection pipeline. The upstream bioinformatic steps (from raw reads to consensus genetic map) are not included (GZ 72829 kb) [file 122_2017_2904_MOESM4_ESM.gz › TMP/SCRIPT/4_Answer_Review/Interaction_Between_Markers/Interaction_between_markers.html]

Interaction between markers


# Interaction between markers

## Interaction between markers

- Calculate interactions
- Analysing the interaction
  - NV 2012
  - Elisa 2012
  - NV 2015
  - Elisa 2015
  - QPCR 2015

This file is part of the publication concerning the genetic determinism of the resistance of Durum Wheat to Durum Wheat. To main QTLs on chromosome 7A and 7B have been detected in the study and the epistatic relationship between these 2 QTLs has been described. However, a reviewer rightly pointed out that the epistatic relationship between every pairs of markers should be checked. The idea of this script is thus to calculate the interaction term of every pair of markers. To avoid huge computation time, we considered only one marker every slice of 10 cM along the genetic map. Then, the script describes the results of these interactions.

Libraries:

```
library(xtable)
```

# Calculate interactions

The script *Interaction\_calculation\_for\_review.R* does all the calculation. It cut the genetic map by slices of 5 cM. For each pair of slice, it randomly takes 2 markers and compute a linear model: phenotyp = u + marker1 + marker2 + marker1 x marker2. The R2 and the interaction term p-values are saved. We used it on a calculation cluster.  
We realized it for the 5 traits of the study: NV2012, NV2015, Elisa2012, Elisa2015, qPCR2015.

```
# Not run
cd ~/work/MOSAIQUE_REVIEW_EPISTASIE
for i in $(echo 1A 1B 2A 2B 3A 3B 4A 4B 5A 5B 6A 6B 7A 7B); do qsub -q normal.q -b yes -cwd -N tmp_epistasie "Rscript Interaction_calculation_for_review.R genotypage.csv phenotypage.csv genetic_map.txt $i NV_blup_AR1_2012" ; done
for i in $(echo 1A 1B 2A 2B 3A 3B 4A 4B 5A 5B 6A 6B 7A 7B); do qsub -q normal.q -b yes -cwd -N tmp_epistasie "Rscript Interaction_calculation_for_review.R genotypage.csv phenotypage.csv genetic_map.txt $i ELISA_blup_AR1_2012" ; done
for i in $(echo 1A 1B 2A 2B 3A 3B 4A 4B 5A 5B 6A 6B 7A 7B); do qsub -q normal.q -b yes -cwd -N tmp_epistasie "Rscript Interaction_calculation_for_review.R genotypage.csv phenotypage.csv genetic_map.txt $i NV_blup_AR1_2015" ; done
for i in $(echo 1A 1B 2A 2B 3A 3B 4A 4B 5A 5B 6A 6B 7A 7B); do qsub -q normal.q -b yes -cwd -N tmp_epistasie "Rscript Interaction_calculation_for_review.R genotypage.csv phenotypage.csv genetic_map.txt $i ELISA_blup_AR1_2015" ; done
for i in $(echo 1A 1B 2A 2B 3A 3B 4A 4B 5A 5B 6A 6B 7A 7B); do qsub -q normal.q -b yes -cwd -N tmp_epistasie "Rscript Interaction_calculation_for_review.R genotypage.csv phenotypage.csv genetic_map.txt $i QPCR_blup_AR1_2015" ; done
```

Then, we concatenated the results of each chromosome in a summar file:

```
# Concatenate results (--> ~287000 raws)
cd ~/work/MOSAIQUE_REVIEW_EPISTASIE
more result_epistasie_K_1A_NV_blup_AR1_2012.txt | head -1 > bilan_NV_2012
cat result_epi*NV_blup_AR1_2012* | grep -v "pheno" | cut -f2- -d" " >>  bilan_NV_2012
more result_epistasie_K_1A_ELISA_blup_AR1_2012.txt | head -1 > bilan_Elisa_2012
cat result_epi*ELISA_blup_AR1_2012* | grep -v "pheno" | cut -f2- -d" "  >>  bilan_Elisa_2012
more result_epistasie_K_1A_NV_blup_AR1_2015.txt | head -1 > bilan_NV_2015
cat result_epi*NV_blup_AR1_2015* | grep -v "pheno" | cut -f2- -d" "  >>  bilan_NV_2015
more result_epistasie_K_1A_ELISA_blup_AR1_2015.txt | head -1 > bilan_Elisa_2015
cat result_epi*ELISA_blup_AR1_2015* | grep -v "pheno" | cut -f2- -d" "  >>  bilan_Elisa_2015
more result_epistasie_K_1A_QPCR_blup_AR1_2015.txt | head -1 > bilan_qPCR_2015
cat result_epi*QPCR_blup_AR1_2015* | grep -v "pheno" | cut -f2- -d" "  >>  bilan_qPCR_2015

#Transfert
gzip bilan*
scp holtz@CC2-login.cirad.fr://homedir/holtz/work/MOSAIQUE_REVIEW_EPISTASIE/bila* .
```

# Analysing the interaction

Load files:

```
# Analyse des fichiers dans R:
NV2012=read.table("bilan_NV_2012.gz", header=T) ; NV2012=NV2012[!is.na(NV2012$pval_inter) , ]
Elisa2012=read.table("bilan_Elisa_2012.gz", header=T) ; Elisa2012=Elisa2012[!is.na(Elisa2012$pval_inter) , ]
NV2015=read.table("bilan_NV_2015.gz", header=T) ; NV2015=NV2015[!is.na(NV2015$pval_inter) , ]
Elisa2015=read.table("bilan_Elisa_2015.gz", header=T) ; Elisa2015=Elisa2015[!is.na(Elisa2015$pval_inter) , ]
qPCR2015=read.table("bilan_qPCR_2015.gz", header=T) ; qPCR2015=qPCR2015[!is.na(qPCR2015$pval_inter) , ]
```

How many comparison did we do?

```
nrow(NV2012)
```

```
## [1] 174060
```

Histogram of p-values of interaction for each variable:

```
par(mfrow=c(2,3))
hist(NV2012$pval_inter , col=rgb(0.1,0.3,0.4,0.6) , border=F, xlab="pvalue interaction between markers", breaks=100, main="NV2012")
hist(Elisa2012$pval_inter , col=rgb(0.1,0.3,0.4,0.6) , border=F, xlab="pvalue interaction between markers", breaks=100, main="Elisa2012")
hist(NV2015$pval_inter , col=rgb(0.1,0.3,0.4,0.6) , border=F, xlab="pvalue interaction between markers", breaks=100, main="NV2015")
hist(Elisa2015$pval_inter , col=rgb(0.1,0.3,0.4,0.6) , border=F, xlab="pvalue interaction between markers", breaks=100, main="Elisa2015")
hist(qPCR2015$pval_inter , col=rgb(0.1,0.3,0.4,0.6) , border=F, xlab="pvalue interaction between markers", breaks=100, main="qPCR2015")
```

Since the number of test we do si very huge, we will use a false discovery rate (FDR) of 5% as a threshold of significativity. For that we use the qvalue library.

```
# qvalue library for FDR
library(qvalue)
```

## NV 2012

Calculate qvalue from pvalues

```
qv = qvalue(p=NV2012$pval_inter, fdr.level=0.05, robust=TRUE)
NV2012$qvalues=qv$qvalues
```

transforme pvalues of markers in LOD:

```
NV2012$pval_mark1=-log10(NV2012$pval_mark1+0.0000000000000000001)
NV2012$pval_mark2=-log10(NV2012$pval_mark2+0.0000000000000000001)
```

How many significant interactions do we have?

```
a=NV2012[which(NV2012$qvalues<0.05) , ]
nrow(a)
```

```
## [1] 1
```

|  | pheno | chromo\_ref | min\_Kref | max\_Kref | mark\_ref | chromo\_other | min\_Kother | max\_Kother | mark\_Kother | R2 | pval\_mark1 | pval\_mark2 | pval\_inter | qvalues |
| --- | --- | --- | --- | --- | --- | --- | --- | --- | --- | --- | --- | --- | --- | --- |
| 9708 | NV\_blup\_AR1\_2012 | 1A | 90 | 95 | Cluster\_9208|Contig1|original@1858 | 6B | 150 | 155 | Cluster\_14323|Contig2|original@958 | 0.09 | 3.71 | 4.74 | 0.00 | 0.00 |

Let’s represent the qvalues we have:

```
plot(sort(NV2012$qvalues) , ylab="q-values", ylim=c(0,1) )
```

What is the lowest q-value we have?

```
min(NV2012$qvalues)
```

```
## [1] 0
```

What interests us is the relationship between the q-value of interaction, and the R2 of the model. Let’s represent both, and check if we find out a pair of marker with a high R2 and a high interaction term? Every pair of markers will be blue, except the interactions between chromosome 7A and chromosome 7B. I will use dots only, except for representing the interaction between the QTL 7A and the QTL 7B described in the study.

```
my_colors=ifelse( (NV2012$chromo_ref=="7B" & NV2012$chromo_other=="7A") | (NV2012$chromo_ref=="7A" & NV2012$chromo_other=="7B") , "red" , "blue")
my_pch=ifelse( NV2012$chromo_ref=="7A" & NV2012$chromo_other=="7B" & NV2012$min_Kref>=115 & NV2012$max_Kref<=120 & NV2012$min_Kother>=45 & NV2012$max_Kother<=50 , 15, 20)
plot(NV2012$R2~NV2012$qvalues, col=my_colors, pch=my_pch, xlab="qvalue", ylab="R2" )
```

It is quite clear that the only relevant QTL is the one described in the study. A few other pairs of markers presents an interaction, but always with a small R2 and are thus probably due to hazard.

We can have a look to the 10 biggest R2:

```
tmp1=head(NV2012[order(NV2012$R2, decreasing=T) , ], 10)
tmp2=head(NV2012[order(NV2012$qvalues) , ] , 10)
```

| pheno | chromo\_ref | min\_Kref | max\_Kref | mark\_ref | chromo\_other | min\_Kother | max\_Kother | mark\_Kother | R2 | pval\_mark1 | pval\_mark2 | pval\_inter | qvalues |
| --- | --- | --- | --- | --- | --- | --- | --- | --- | --- | --- | --- | --- | --- |
| NV\_blup\_AR1\_2012 | 7A | 115 | 120 | Cluster\_17879|Contig1|likelySeq@409 | 7B | 55 | 60 | Cluster\_932|Contig1|original@517 | 0.20 | 19.00 | 19.00 | 0.00 | 0.19 |
| NV\_blup\_AR1\_2012 | 7A | 115 | 120 | Cluster\_17879|Contig1|likelySeq@409 | 7B | 45 | 50 | Cluster\_2114|Contig3|original@2227 | 0.18 | 19.00 | 19.00 | 0.00 | 0.05 |
| NV\_blup\_AR1\_2012 | 7A | 125 | 130 | Cluster\_8137|Contig3|original@2003 | 7B | 55 | 60 | Cluster\_3623|Contig2|original@924 | 0.17 | 4.96 | 19.00 | 0.07 | 0.59 |
| NV\_blup\_AR1\_2012 | 4B | 95 | 100 | Cluster\_1185|Contig2|original@96 | 7B | 55 | 60 | Cluster\_3623|Contig2|original@924 | 0.17 | 2.42 | 4.38 | 0.55 | 0.80 |
| NV\_blup\_AR1\_2012 | 4A | 55 | 60 | Cluster\_12204|Contig1|likelySeq@2065 | 7B | 55 | 60 | Cluster\_3623|Contig2|original@924 | 0.16 | 2.48 | 4.17 | 0.66 | 0.82 |
| NV\_blup\_AR1\_2012 | 4B | 100 | 105 | Cluster\_11589|Contig1|original@203 | 7B | 55 | 60 | Cluster\_932|Contig1|original@517 | 0.15 | 2.98 | 4.70 | 0.77 | 0.84 |
| NV\_blup\_AR1\_2012 | 4A | 65 | 70 | Cluster\_18343|Contig1|complementarySeq@1368 | 7B | 55 | 60 | Cluster\_932|Contig1|original@517 | 0.15 | 1.37 | 4.07 | 0.16 | 0.66 |
| NV\_blup\_AR1\_2012 | 7B | 55 | 60 | Cluster\_3623|Contig2|original@924 | 7B | 190 | 195 | Cluster\_2843|Contig3|original@1198 | 0.15 | 5.52 | 0.75 | 0.15 | 0.65 |
| NV\_blup\_AR1\_2012 | 7A | 100 | 105 | Cluster\_6826|Contig3|complementarySeq@624 | 7B | 55 | 60 | Cluster\_3623|Contig2|original@924 | 0.15 | 3.17 | 19.00 | 0.40 | 0.77 |
| NV\_blup\_AR1\_2012 | 7A | 115 | 120 | Cluster\_17879|Contig1|likelySeq@409 | 7B | 85 | 90 | Cluster\_8184|Contig2|original@531 | 0.15 | 19.00 | 19.00 | 0.00 | 0.31 |

As presented in this table, the highest R2 always involved QTL 7A or QTL 7B, or both.

Let’s look the 10 lowest qvalues:

| pheno | chromo\_ref | min\_Kref | max\_Kref | mark\_ref | chromo\_other | min\_Kother | max\_Kother | mark\_Kother | R2 | pval\_mark1 | pval\_mark2 | pval\_inter | qvalues |
| --- | --- | --- | --- | --- | --- | --- | --- | --- | --- | --- | --- | --- | --- |
| NV\_blup\_AR1\_2012 | 1A | 90 | 95 | Cluster\_9208|Contig1|original@1858 | 6B | 150 | 155 | Cluster\_14323|Contig2|original@958 | 0.09 | 3.71 | 4.74 | 0.00 | 0.00 |
| NV\_blup\_AR1\_2012 | 4A | 160 | 165 | Cluster\_5029|Contig6|likelySeq@165 | 4A | 145 | 150 | Cluster\_11872|Contig8|original@205 | 0.08 | 2.73 | 5.00 | 0.00 | 0.05 |
| NV\_blup\_AR1\_2012 | 7A | 115 | 120 | Cluster\_17879|Contig1|likelySeq@409 | 7B | 45 | 50 | Cluster\_2114|Contig3|original@2227 | 0.18 | 19.00 | 19.00 | 0.00 | 0.05 |
| NV\_blup\_AR1\_2012 | 1A | 65 | 70 | Cluster\_73|Contig5|likelySeq@1874 | 4A | 110 | 115 | Cluster\_1986|Contig1|original@236 | 0.07 | 3.13 | 5.30 | 0.00 | 0.19 |
| NV\_blup\_AR1\_2012 | 1A | 85 | 90 | Cluster\_3611|Contig2|likelySeq@143 | 7A | 90 | 95 | Cluster\_21715|Contig3|original@469 | 0.07 | 1.24 | 0.33 | 0.00 | 0.19 |
| NV\_blup\_AR1\_2012 | 1A | 90 | 95 | Cluster\_9208|Contig1|original@1858 | 6B | 145 | 150 | Cluster\_14088|Contig1|complementarySeq@359 | 0.07 | 3.33 | 4.06 | 0.00 | 0.19 |
| NV\_blup\_AR1\_2012 | 1A | 95 | 100 | Cluster\_5844|Contig3|original@293 | 6B | 150 | 155 | Cluster\_11335|Contig3|original@831 | 0.07 | 2.44 | 5.05 | 0.00 | 0.19 |
| NV\_blup\_AR1\_2012 | 3A | 0 | 5 | Cluster\_3261|Contig1|original@112 | 5B | 230 | 235 | Cluster\_2698|Contig2|original@1306 | 0.07 | 5.10 | 2.34 | 0.00 | 0.19 |
| NV\_blup\_AR1\_2012 | 3A | 0 | 5 | Cluster\_3261|Contig1|original@112 | 5B | 240 | 245 | Cluster\_10467|Contig1|original@1549 | 0.08 | 4.89 | 3.08 | 0.00 | 0.19 |
| NV\_blup\_AR1\_2012 | 3A | 155 | 160 | Cluster\_1292|Contig2|original@87 | 5B | 180 | 185 | Cluster\_11865|Contig3|original@318 | 0.06 | 3.05 | 2.57 | 0.00 | 0.19 |

## Elisa 2012

Calculate qvalue from pvalues

```
qv = qvalue(p=Elisa2012$pval_inter, fdr.level=0.05, robust=TRUE)
Elisa2012$qvalues=qv$qvalues
```

transforme pvalues of markers in LOD:

```
Elisa2012$pval_mark1=-log10(Elisa2012$pval_mark1+0.0000000000000000001)
Elisa2012$pval_mark2=-log10(Elisa2012$pval_mark2+0.0000000000000000001)
```

How many significant interactions do we have?

```
my_signif=Elisa2012[which(Elisa2012$qvalues<0.05) , ]
nrow(my_signif)
```

```
## [1] 6
```

Let’s show the details of these significant interactions:

| pheno | chromo\_ref | min\_Kref | max\_Kref | mark\_ref | chromo\_other | min\_Kother | max\_Kother | mark\_Kother | R2 | pval\_mark1 | pval\_mark2 | pval\_inter | qvalues |
| --- | --- | --- | --- | --- | --- | --- | --- | --- | --- | --- | --- | --- | --- |
| ELISA\_blup\_AR1\_2012 | 4A | 20 | 25 | Cluster\_622|Contig5|original@894 | 5B | 35 | 40 | Cluster\_3003|Contig2|original@468 | 0.08 | 4.82 | 0.92 | 0.00 | 0.00 |
| ELISA\_blup\_AR1\_2012 | 7A | 100 | 105 | Cluster\_811|Contig2|original@731 | 7B | 55 | 60 | Cluster\_932|Contig1|original@517 | 0.26 | 19.00 | 19.00 | 0.00 | 0.03 |
| ELISA\_blup\_AR1\_2012 | 7A | 100 | 105 | Cluster\_811|Contig2|original@731 | 7B | 80 | 85 | Cluster\_3627|Contig1|complementarySeq@440 | 0.18 | 19.00 | 19.00 | 0.00 | 0.00 |
| ELISA\_blup\_AR1\_2012 | 7A | 100 | 105 | Cluster\_811|Contig2|original@731 | 7B | 85 | 90 | Cluster\_3743|Contig5|original@2638 | 0.20 | 19.00 | 19.00 | 0.00 | 0.03 |
| ELISA\_blup\_AR1\_2012 | 7A | 115 | 120 | Cluster\_17879|Contig1|likelySeq@409 | 7B | 80 | 85 | Cluster\_3627|Contig1|complementarySeq@440 | 0.28 | 19.00 | 19.00 | 0.00 | 0.00 |
| ELISA\_blup\_AR1\_2012 | 7A | 115 | 120 | Cluster\_17879|Contig1|likelySeq@409 | 7B | 90 | 95 | Cluster\_7636|Contig1|original@1632 | 0.28 | 19.00 | 19.00 | 0.00 | 0.03 |

All the significant interactions are related to the interactions between 7A and 7B QTL. The highest interaction is found between the 2 zones containing the QTLs IC. Other significant interaction are found for the slices surrounding the QTLs, due to linkage desequilibrium.

The same representation as above, looking for relationship between R2 and qvalue, highlighting the QTL and 7A x 7B interactions:

```
my_colors=ifelse( (Elisa2012$chromo_ref=="7B" & Elisa2012$chromo_other=="7A") | (Elisa2012$chromo_ref=="7A" & Elisa2012$chromo_other=="7B") , "red" , "blue")
my_pch=ifelse( Elisa2012$chromo_ref=="7A" & Elisa2012$chromo_other=="7B" & Elisa2012$min_Kref>=100 & Elisa2012$max_Kref<=105 & Elisa2012$min_Kother>=55 & Elisa2012$max_Kother<=60 , 15, 20)
plot(Elisa2012$R2~Elisa2012$qvalues, col=my_colors, pch=my_pch, xlab="qvalue", ylab="R2" )
abline(v=0.05, col="grey")
```

//: =========================================================================================================================

## NV 2015

Calculate qvalue from pvalues

```
qv = qvalue(p=NV2015$pval_inter, fdr.level=0.05, robust=TRUE)
NV2015$qvalues=qv$qvalues
```

transforme pvalues of markers in LOD:

```
NV2015$pval_mark1=-log10(NV2015$pval_mark1+0.0000000000000000001)
NV2015$pval_mark2=-log10(NV2015$pval_mark2+0.0000000000000000001)
```

How many significant interactions do we have?

```
my_signif=NV2015[which(NV2015$qvalues<0.05) , ]
nrow(my_signif)
```

```
## [1] 4
```

Let’s show the details of these significant interactions:

| pheno | chromo\_ref | min\_Kref | max\_Kref | mark\_ref | chromo\_other | min\_Kother | max\_Kother | mark\_Kother | R2 | pval\_mark1 | pval\_mark2 | pval\_inter | qvalues |
| --- | --- | --- | --- | --- | --- | --- | --- | --- | --- | --- | --- | --- | --- |
| NV\_blup\_AR1\_2015 | 2A | 170 | 175 | Cluster\_6201|Contig2|original@351 | 4B | 155 | 160 | Cluster\_1288|Contig2|original@142 | 0.08 | 1.04 | 4.07 | 0.00 | 0.00 |
| NV\_blup\_AR1\_2015 | 4A | 145 | 150 | Cluster\_11872|Contig8|original@205 | 4A | 160 | 165 | Cluster\_5029|Contig6|likelySeq@165 | 0.08 | 5.10 | 2.89 | 0.00 | 0.04 |
| NV\_blup\_AR1\_2015 | 4A | 160 | 165 | Cluster\_8384|Contig2|likelySeq@315 | 4A | 145 | 150 | Cluster\_11872|Contig8|original@205 | 0.09 | 2.89 | 5.52 | 0.00 | 0.00 |
| NV\_blup\_AR1\_2015 | 4A | 160 | 165 | Cluster\_8384|Contig2|likelySeq@315 | 4A | 150 | 155 | Cluster\_9132|Contig1|likelySeq@42 | 0.08 | 3.02 | 5.10 | 0.00 | 0.04 |

Let’s represent the qvalues we have:

```
plot(sort(NV2015$qvalues) , ylab="q-values", ylim=c(0,1) )
```

Representation of R2 and qvalues

```
my_colors=ifelse( (NV2015$chromo_ref=="7B" & NV2015$chromo_other=="7A") | (NV2015$chromo_ref=="7A" & NV2015$chromo_other=="7B") , "red" , "blue")
my_pch=ifelse( NV2015$chromo_ref=="7A" & NV2015$chromo_other=="7B" & NV2015$min_Kref>=115 & NV2015$max_Kref<=120 & NV2015$min_Kother>=55 & NV2015$max_Kother<=60 , 15, 20)
plot(NV2015$R2~NV2015$qvalues, col=my_colors, pch=my_pch, xlab="qvalue", ylab="R2" )
abline(v=0.05, col="grey")
```

It is quite clear that the only relevant QTL is the one described in the study. A few other pairs of markers presents an interaction, but always with a small R2 and are thus probably due to hazard.

We can have a look to the 10 biggest R2:

```
tmp1=head(NV2015[order(NV2015$R2, decreasing=T) , ], 10)
tmp2=head(NV2015[order(NV2015$qvalues) , ] , 10)
```

| pheno | chromo\_ref | min\_Kref | max\_Kref | mark\_ref | chromo\_other | min\_Kother | max\_Kother | mark\_Kother | R2 | pval\_mark1 | pval\_mark2 | pval\_inter | qvalues |
| --- | --- | --- | --- | --- | --- | --- | --- | --- | --- | --- | --- | --- | --- |
| NV\_blup\_AR1\_2015 | 7A | 115 | 120 | Cluster\_932|Contig2|original@602 | 7B | 55 | 60 | Cluster\_932|Contig1|original@517 | 0.22 | 19.00 | 19.00 | 0.00 | 0.10 |
| NV\_blup\_AR1\_2015 | 7A | 115 | 120 | Cluster\_932|Contig2|original@602 | 7B | 50 | 55 | Cluster\_8668|Contig2|original@192 | 0.20 | 19.00 | 19.00 | 0.00 | 0.16 |
| NV\_blup\_AR1\_2015 | 2A | 215 | 220 | Cluster\_14091|Contig1|original@109 | 4B | 100 | 105 | Cluster\_6287|Contig3|original@107 | 0.18 | 19.00 | 6.00 | 0.45 | 0.74 |
| NV\_blup\_AR1\_2015 | 2A | 215 | 220 | Cluster\_14091|Contig1|original@109 | 7B | 55 | 60 | Cluster\_932|Contig1|original@517 | 0.18 | 1.85 | 2.60 | 0.08 | 0.53 |
| NV\_blup\_AR1\_2015 | 7B | 190 | 195 | Cluster\_1989|Contig7|original@189 | 7B | 55 | 60 | Cluster\_3623|Contig2|original@924 | 0.17 | 1.33 | 19.00 | 0.15 | 0.60 |
| NV\_blup\_AR1\_2015 | 2A | 185 | 190 | Cluster\_6910|Contig1|original@443 | 7B | 55 | 60 | Cluster\_3623|Contig2|original@924 | 0.17 | 1.08 | 3.81 | 0.05 | 0.49 |
| NV\_blup\_AR1\_2015 | 7A | 125 | 130 | Cluster\_9668|Contig1|complementarySeq@303 | 7B | 55 | 60 | Cluster\_932|Contig3|original@346 | 0.17 | 4.89 | 19.00 | 0.07 | 0.51 |
| NV\_blup\_AR1\_2015 | 2A | 215 | 220 | Cluster\_14091|Contig1|original@109 | 7A | 115 | 120 | Cluster\_2439|Contig7|original@810 | 0.17 | 19.00 | 5.70 | 0.14 | 0.59 |
| NV\_blup\_AR1\_2015 | 2A | 205 | 210 | Cluster\_14710|Contig1|original@326 | 7B | 55 | 60 | Cluster\_3623|Contig2|original@924 | 0.17 | 1.29 | 3.47 | 0.20 | 0.63 |
| NV\_blup\_AR1\_2015 | 2A | 215 | 220 | Cluster\_14091|Contig1|original@109 | 7B | 50 | 55 | Cluster\_8668|Contig2|original@192 | 0.17 | 1.77 | 1.92 | 0.05 | 0.48 |

As presented in this table, the highest R2 always involved QTL 7A or QTL 7B, or both.

Let’s look the 10 lowest qvalues:

| pheno | chromo\_ref | min\_Kref | max\_Kref | mark\_ref | chromo\_other | min\_Kother | max\_Kother | mark\_Kother | R2 | pval\_mark1 | pval\_mark2 | pval\_inter | qvalues |
| --- | --- | --- | --- | --- | --- | --- | --- | --- | --- | --- | --- | --- | --- |
| NV\_blup\_AR1\_2015 | 2A | 170 | 175 | Cluster\_6201|Contig2|original@351 | 4B | 155 | 160 | Cluster\_1288|Contig2|original@142 | 0.08 | 1.04 | 4.07 | 0.00 | 0.00 |
| NV\_blup\_AR1\_2015 | 4A | 160 | 165 | Cluster\_8384|Contig2|likelySeq@315 | 4A | 145 | 150 | Cluster\_11872|Contig8|original@205 | 0.09 | 2.89 | 5.52 | 0.00 | 0.00 |
| NV\_blup\_AR1\_2015 | 4A | 145 | 150 | Cluster\_11872|Contig8|original@205 | 4A | 160 | 165 | Cluster\_5029|Contig6|likelySeq@165 | 0.08 | 5.10 | 2.89 | 0.00 | 0.04 |
| NV\_blup\_AR1\_2015 | 4A | 160 | 165 | Cluster\_8384|Contig2|likelySeq@315 | 4A | 150 | 155 | Cluster\_9132|Contig1|likelySeq@42 | 0.08 | 3.02 | 5.10 | 0.00 | 0.04 |
| NV\_blup\_AR1\_2015 | 3A | 0 | 5 | Cluster\_6008|Contig1|original@625 | 5B | 245 | 250 | Cluster\_8784|Contig1|original@431 | 0.08 | 4.44 | 3.68 | 0.00 | 0.09 |
| NV\_blup\_AR1\_2015 | 2B | 105 | 110 | Cluster\_1202|Contig5|original@10551 | 5B | 210 | 215 | Cluster\_1322|Contig2|original@1062 | 0.07 | 2.25 | 3.77 | 0.00 | 0.10 |
| NV\_blup\_AR1\_2015 | 2A | 170 | 175 | Cluster\_6201|Contig2|original@351 | 4B | 145 | 150 | Cluster\_5054|Contig2|original@179 | 0.07 | 0.64 | 3.19 | 0.00 | 0.10 |
| NV\_blup\_AR1\_2015 | 2B | 105 | 110 | Cluster\_1202|Contig5|original@10551 | 5B | 200 | 205 | Cluster\_4194|Contig2|complementarySeq@406 | 0.06 | 2.61 | 2.57 | 0.00 | 0.10 |
| NV\_blup\_AR1\_2015 | 7A | 115 | 120 | Cluster\_932|Contig2|original@602 | 7B | 55 | 60 | Cluster\_932|Contig1|original@517 | 0.22 | 19.00 | 19.00 | 0.00 | 0.10 |
| NV\_blup\_AR1\_2015 | 1B | 225 | 230 | Cluster\_9819|Contig1|original@723 | 6A | 95 | 100 | Cluster\_1195|Contig1|complementarySeq@2217 | 0.07 | 4.09 | 1.80 | 0.00 | 0.14 |

## Elisa 2015

Calculate qvalue from pvalues

```
qv = qvalue(p=Elisa2015$pval_inter, fdr.level=0.05, robust=TRUE)
Elisa2015$qvalues=qv$qvalues
```

transforme pvalues of markers in LOD:

```
Elisa2015$pval_mark1=-log10(Elisa2015$pval_mark1+0.0000000000000000001)
Elisa2015$pval_mark2=-log10(Elisa2015$pval_mark2+0.0000000000000000001)
```

How many significant interactions do we have?

```
my_signif=Elisa2015[which(Elisa2015$qvalues<0.05) , ]
nrow(my_signif)
```

```
## [1] 2
```

Let’s show the details of these significant interactions:

| pheno | chromo\_ref | min\_Kref | max\_Kref | mark\_ref | chromo\_other | min\_Kother | max\_Kother | mark\_Kother | R2 | pval\_mark1 | pval\_mark2 | pval\_inter | qvalues |
| --- | --- | --- | --- | --- | --- | --- | --- | --- | --- | --- | --- | --- | --- |
| ELISA\_blup\_AR1\_2015 | 3A | 10 | 15 | Cluster\_10171|Contig1|original@3288 | 5A | 230 | 235 | Cluster\_11126|Contig1|original@548 | 0.08 | 1.95 | 2.68 | 0.00 | 0.00 |
| ELISA\_blup\_AR1\_2015 | 7A | 115 | 120 | Cluster\_10461|Contig1|likelySeq@159 | 7B | 80 | 85 | Cluster\_6554|Contig2|original@852 | 0.28 | 19.00 | 19.00 | 0.00 | 0.00 |

All the significant interactions are related to the interactions between 7A and 7B QTL. The highest interaction is found between the 2 zones containing the QTLs IC. Other significant interaction are found for the slices surrounding the QTLs, due to linkage desequilibrium.

The same representation as above, looking for relationship between R2 and qvalue, highlighting the QTL and 7A x 7B interactions:

```
my_colors=ifelse( (Elisa2015$chromo_ref=="7B" & Elisa2015$chromo_other=="7A") | (Elisa2015$chromo_ref=="7A" & Elisa2015$chromo_other=="7B") , "red" , "blue")
my_pch=ifelse( Elisa2015$chromo_ref=="7A" & Elisa2015$chromo_other=="7B" & Elisa2015$min_Kref>=115 & Elisa2015$max_Kref<=120 & Elisa2015$min_Kother>=80 & Elisa2015$max_Kother<=85 , 15, 20)
plot(Elisa2015$R2~Elisa2015$qvalues, col=my_colors, pch=my_pch, xlab="qvalue", ylab="R2" )
abline(v=0.05, col="grey")
```

//: =========================================================================================================================

## QPCR 2015

Calculate qvalue from pvalues

```
qv = qvalue(p=qPCR2015$pval_inter, fdr.level=0.05, robust=TRUE)
qPCR2015$qvalues=qv$qvalues
```

transforme pvalues of markers in LOD:

```
qPCR2015$pval_mark1=-log10(qPCR2015$pval_mark1+0.0000000000000000001)
qPCR2015$pval_mark2=-log10(qPCR2015$pval_mark2+0.0000000000000000001)
```

How many significant interactions do we have?

```
a=qPCR2015[which(qPCR2015$qvalues<0.05) , ]
nrow(a)
```

```
## [1] 4
```

|  | pheno | chromo\_ref | min\_Kref | max\_Kref | mark\_ref | chromo\_other | min\_Kother | max\_Kother | mark\_Kother | R2 | pval\_mark1 | pval\_mark2 | pval\_inter | qvalues |
| --- | --- | --- | --- | --- | --- | --- | --- | --- | --- | --- | --- | --- | --- | --- |
| 225071 | QPCR\_blup\_AR1\_2015 | 7A | 115 | 120 | Cluster\_16523|Contig1|complementarySeq@373 | 7B | 50 | 55 | Cluster\_8668|Contig2|original@192 | 0.25 | 19.00 | 19.00 | 0.00 | 0.00 |
| 225077 | QPCR\_blup\_AR1\_2015 | 7A | 115 | 120 | Cluster\_16523|Contig1|complementarySeq@373 | 7B | 80 | 85 | Cluster\_9012|Contig1|original@267 | 0.15 | 19.00 | 19.00 | 0.00 | 0.04 |
| 225255 | QPCR\_blup\_AR1\_2015 | 7A | 125 | 130 | Cluster\_1722|Contig1|original@583 | 7B | 50 | 55 | Cluster\_8668|Contig2|original@192 | 0.19 | 19.00 | 19.00 | 0.00 | 0.04 |
| 225261 | QPCR\_blup\_AR1\_2015 | 7A | 125 | 130 | Cluster\_1722|Contig1|original@583 | 7B | 80 | 85 | Cluster\_8884|Contig1|complementarySeq@1336 | 0.11 | 19.00 | 5.05 | 0.00 | 0.04 |

Let’s represent the qvalues we have:

```
plot(sort(qPCR2015$qvalues) , ylab="q-values", ylim=c(0,1) )
```

What is the lowest q-value we have?

```
min(qPCR2015$qvalues)
```

```
## [1] 0
```

What interests us is the relationship between the q-value of interaction, and the R2 of the model. Let’s represent both, and check if we find out a pair of marker with a high R2 and a high interaction term? Every pair of markers will be blue, except the interactions between chromosome 7A and chromosome 7B. I will use dots only, except for representing the interaction between the QTL 7A and the QTL 7B described in the study.

```
my_colors=ifelse( (qPCR2015$chromo_ref=="7B" & qPCR2015$chromo_other=="7A") | (qPCR2015$chromo_ref=="7A" & qPCR2015$chromo_other=="7B") , "red" , "blue")
my_pch=ifelse( qPCR2015$chromo_ref=="7A" & qPCR2015$chromo_other=="7B" & qPCR2015$min_Kref>=115 & qPCR2015$max_Kref<=120 & qPCR2015$min_Kother>=50 & qPCR2015$max_Kother<=55 , 15, 20)
plot(qPCR2015$R2~qPCR2015$qvalues, col=my_colors, pch=my_pch, xlab="qvalue", ylab="R2" )
```

It is quite clear that the only relevant QTL is the one described in the study. A few other pairs of markers presents an interaction, but always with a small R2 and are thus probably due to hazard.

We can have a look to the 10 biggest R2:

```
tmp1=head(qPCR2015[order(qPCR2015$R2, decreasing=T) , ], 10)
tmp2=head(qPCR2015[order(qPCR2015$qvalues) , ] , 10)
```

| pheno | chromo\_ref | min\_Kref | max\_Kref | mark\_ref | chromo\_other | min\_Kother | max\_Kother | mark\_Kother | R2 | pval\_mark1 | pval\_mark2 | pval\_inter | qvalues |
| --- | --- | --- | --- | --- | --- | --- | --- | --- | --- | --- | --- | --- | --- |
| QPCR\_blup\_AR1\_2015 | 7A | 115 | 120 | Cluster\_16523|Contig1|complementarySeq@373 | 7B | 50 | 55 | Cluster\_8668|Contig2|original@192 | 0.25 | 19.00 | 19.00 | 0.00 | 0.00 |
| QPCR\_blup\_AR1\_2015 | 7A | 125 | 130 | Cluster\_1722|Contig1|original@583 | 7B | 50 | 55 | Cluster\_8668|Contig2|original@192 | 0.19 | 19.00 | 19.00 | 0.00 | 0.04 |
| QPCR\_blup\_AR1\_2015 | 7A | 105 | 110 | Cluster\_9397|Contig1|likelySeq@1055 | 7B | 50 | 55 | Cluster\_8668|Contig2|original@192 | 0.16 | 5.52 | 19.00 | 0.00 | 0.40 |
| QPCR\_blup\_AR1\_2015 | 7A | 130 | 135 | Cluster\_10762|Contig2|original@86 | 7B | 50 | 55 | Cluster\_8668|Contig2|original@192 | 0.15 | 4.85 | 19.00 | 0.03 | 0.61 |
| QPCR\_blup\_AR1\_2015 | 7A | 115 | 120 | Cluster\_16523|Contig1|complementarySeq@373 | 7B | 80 | 85 | Cluster\_9012|Contig1|original@267 | 0.15 | 19.00 | 19.00 | 0.00 | 0.04 |
| QPCR\_blup\_AR1\_2015 | 7A | 95 | 100 | Cluster\_10130|Contig1|complementarySeq@473 | 7B | 55 | 60 | Cluster\_3623|Contig2|original@924 | 0.15 | 4.04 | 19.00 | 0.01 | 0.57 |
| QPCR\_blup\_AR1\_2015 | 7A | 100 | 105 | Cluster\_10107|Contig2|likelySeq@262 | 7B | 55 | 60 | Cluster\_3623|Contig2|original@924 | 0.15 | 4.36 | 19.00 | 0.02 | 0.59 |
| QPCR\_blup\_AR1\_2015 | 5A | 0 | 5 | Cluster\_3397|Contig1|complementarySeq@200 | 7B | 50 | 55 | Cluster\_8668|Contig2|original@192 | 0.14 | 1.05 | 2.80 | 0.05 | 0.65 |
| QPCR\_blup\_AR1\_2015 | 7A | 135 | 140 | Cluster\_730|Contig2|complementarySeq@3177 | 7B | 55 | 60 | Cluster\_932|Contig3|original@346 | 0.14 | 4.72 | 19.00 | 0.02 | 0.61 |
| QPCR\_blup\_AR1\_2015 | 2B | 10 | 15 | Cluster\_600|Contig4|original@1198 | 7B | 55 | 60 | Cluster\_3623|Contig2|original@924 | 0.14 | 1.99 | 2.91 | 0.07 | 0.68 |

As presented in this table, the highest R2 always involved QTL 7A or QTL 7B, or both.

Let’s look the 10 lowest qvalues:

| pheno | chromo\_ref | min\_Kref | max\_Kref | mark\_ref | chromo\_other | min\_Kother | max\_Kother | mark\_Kother | R2 | pval\_mark1 | pval\_mark2 | pval\_inter | qvalues |
| --- | --- | --- | --- | --- | --- | --- | --- | --- | --- | --- | --- | --- | --- |
| QPCR\_blup\_AR1\_2015 | 7A | 115 | 120 | Cluster\_16523|Contig1|complementarySeq@373 | 7B | 50 | 55 | Cluster\_8668|Contig2|original@192 | 0.25 | 19.00 | 19.00 | 0.00 | 0.00 |
| QPCR\_blup\_AR1\_2015 | 7A | 115 | 120 | Cluster\_16523|Contig1|complementarySeq@373 | 7B | 80 | 85 | Cluster\_9012|Contig1|original@267 | 0.15 | 19.00 | 19.00 | 0.00 | 0.04 |
| QPCR\_blup\_AR1\_2015 | 7A | 125 | 130 | Cluster\_1722|Contig1|original@583 | 7B | 50 | 55 | Cluster\_8668|Contig2|original@192 | 0.19 | 19.00 | 19.00 | 0.00 | 0.04 |
| QPCR\_blup\_AR1\_2015 | 7A | 125 | 130 | Cluster\_1722|Contig1|original@583 | 7B | 80 | 85 | Cluster\_8884|Contig1|complementarySeq@1336 | 0.11 | 19.00 | 5.05 | 0.00 | 0.04 |
| QPCR\_blup\_AR1\_2015 | 3A | 10 | 15 | Cluster\_10171|Contig1|original@3288 | 5A | 250 | 255 | Cluster\_10187|Contig1|complementarySeq@502 | 0.07 | 1.99 | 1.54 | 0.00 | 0.06 |
| QPCR\_blup\_AR1\_2015 | 7A | 125 | 130 | Cluster\_1722|Contig1|original@583 | 7B | 90 | 95 | Cluster\_6745|Contig2|original@111 | 0.12 | 19.00 | 19.00 | 0.00 | 0.08 |
| QPCR\_blup\_AR1\_2015 | 1A | 110 | 115 | Cluster\_10416|Contig1|original@242 | 7A | 225 | 230 | Cluster\_5352|Contig3|original@291 | 0.06 | 1.97 | 2.31 | 0.00 | 0.12 |
| QPCR\_blup\_AR1\_2015 | 1B | 170 | 175 | Cluster\_9241|Contig2|complementarySeq@284 | 7A | 55 | 60 | Cluster\_6112|Contig1|original@1269 | 0.06 | 1.70 | 1.98 | 0.00 | 0.12 |
| QPCR\_blup\_AR1\_2015 | 3B | 160 | 165 | Cluster\_798|Contig5|original@1881 | 4B | 115 | 120 | Cluster\_7998|Contig2|original@291 | 0.08 | 4.04 | 4.80 | 0.00 | 0.12 |
| QPCR\_blup\_AR1\_2015 | 4B | 110 | 115 | Cluster\_7008|Contig2|original@125 | 6A | 110 | 115 | Cluster\_11896|Contig2|original@393 | 0.07 | 0.45 | 4.03 | 0.00 | 0.21 |

Yan Holtz

December 2016
