# Supplementary material for: Epistatic determinism of durum wheat resistance to the wheat spindle streak mosaic virus
Source: Theor Appl Genet. 2017 Apr 27;130(7):1491–505. doi: 10.1007/s00122-017-2904-6 (PMC5487696; doi:10.1007/s00122-017-2904-6)
Supplement: Supplementary file 4 — Online Resource 4: Data and R scripts for reproducible QTL detection. Data and R script (.csv and.rmd format) are provided in this tar archive. A scheme aims to explain the content of each file and its role in the QTL detection pipeline. The upstream bioinformatic steps (from raw reads to consensus genetic map) are not included (GZ 72829 kb) [file 122_2017_2904_MOESM4_ESM.gz › TMP/SCRIPT/3_Figures_and_Tables/Figures_and_Tables.html]

Epistatic determinism of durum wheat resistance to the Wheat Spindle Streak Mosaic Virus


# Epistatic determinism of durum wheat resistance to the Wheat Spindle Streak Mosaic Virus

## Epistatic determinism of durum wheat resistance to the Wheat Spindle Streak Mosaic Virus

- 1/ Upload data
- 2/ Basic statistics concerning the experiments
  - Counts of plants
  - Counts of genotypes
  - Basic features
- 3/ Relationship between phenotyping methods
- 4/ QTL representation
  - Summary table
  - Visualize LOD-scores
  - Visualize IC
- 5/ Epistatic effect between QTLs

This file is a supplementary data attached with the publication. It aims to describe most of the analysis done in this study. The bioinformatics part is not included (reads preparation, mapping, SNP calling, imputation and genetic map building)

# 1/ Upload data

Let’s upload every files, packages and objects we will need in the rest of the script.

Path:

```
my_path="~/Dropbox/Publi_Mosaique/DATA/DATA"
```

Let’s upload 6 files: - all\_pheno is a file with every phenotypes available (all pops and controls , all years, all phenotyping methods) - map is the genetic consensus map - Y\_DS is the summary of phenotypes for Dic2 x Silur (DS), including BLUPs - Y\_DL is the summary of phenotypes for Dic2 x Lloyd (DL), including BLUPs - DS is the QTL analysis summary for DS: it gives the LOD and other informations for each markers - DL is the QTL analysis summary for DL: it gives the LOD and other informations for each markers

```
# Raw phenotypuc data, all pop & all years:
all_pheno <- read.table(paste(my_path,"Data2012_2015.csv",sep="/") , header=T , sep="," , na.strings="NA")
# Consensus genetic map:
map  <- read.table(paste(my_path,"genetic_map.txt",sep="/") , header=T , sep="," , na.strings="NA")
# blups & LOD scores for DS
Y_DS <- read.table(paste(my_path,"DS/phenotypage.csv",sep="/") , header=T , sep=";" , na.strings="NA")
DS   <- read.table(paste(my_path,"DS/bilan_simple_marker",sep="/") , header=T , sep="," , na.strings="NA")
# blups & LOD scores for DL
Y_DL <- read.table(paste(my_path,"DL/phenotypage.csv",sep="/") , header=T , sep=";" , na.strings="NA")
DL   <- read.table(paste(my_path,"DL/bilan_simple_marker",sep="/") , header=T , sep="," , na.strings="NA")
# blups & LOD scores for both pops groupes together
Y_BOTH <- read.table(paste(my_path,"GROUPED/phenotypage.csv",sep="/") , header=T , sep=";" , na.strings="NA")
BOTH   <- read.table(paste(my_path,"GROUPED/bilan_simple_marker",sep="/") , header=T , sep="," , na.strings="NA")
```

Charge some libraries:

```
library(RColorBrewer)
library(xtable)
```

# 2/ Basic statistics concerning the experiments

## Counts of plants

How many plants were present in 2012 for each pop and parents:

```
#For 2012:
table(all_pheno$pop[all_pheno$Year=="2012"])
```

```
## 
##          Dic2  Dic2 x Lloyd  Dic2 x Silur Dic2 x Soldur          dicA 
##             1           187           246           100             2 
##          dicB          dicC          dicE          dicF          dicG 
##             2             2             2             2             2 
##          dicH          dicI          dicJ         Lloyd      pescadou 
##             2             2             2             5           149 
##         Silur        Soldur 
##             4            10
```

How many plants were present in 2015 for each pop and parents:

```
#For 2015:
table(all_pheno$pop[all_pheno$Year=="2015"])
```

```
## 
##          Dic2  Dic2 x Lloyd  Dic2 x Silur Dic2 x Soldur          dicA 
##             2           243           218            30             0 
##          dicB          dicC          dicE          dicF          dicG 
##             0             0             0             0             0 
##          dicH          dicI          dicJ         Lloyd      pescadou 
##             0             0             0             3           100 
##         Silur        Soldur 
##             2             2
```

## Counts of genotypes

How many genotypes were present in 2012 and 2015 for each pop?:

```
#For DS in 2012:
length( unique( all_pheno$genotype[ which(all_pheno$pop=="Dic2 x Silur" & all_pheno$Year=="2012") ]))
```

```
## [1] 165
```

```
#For DS in 2015:
length( unique( all_pheno$genotype[ which(all_pheno$pop=="Dic2 x Silur" & all_pheno$Year=="2015") ]))
```

```
## [1] 164
```

```
#For DL in 2012:
length( unique( all_pheno$genotype[ which(all_pheno$pop=="Dic2 x Lloyd" & all_pheno$Year=="2012") ]))
```

```
## [1] 187
```

```
#For DL in 2015:
length( unique( all_pheno$genotype[ which(all_pheno$pop=="Dic2 x Lloyd" & all_pheno$Year=="2015") ]))
```

```
## [1] 189
```

How genotypes are repeated in 2012 AND in 2015 for DS?

```
aa=table( all_pheno[which(all_pheno$Year=="2012" & all_pheno$pop=="Dic2 x Silur" ) , "genotype"] )
aa=names(aa[aa>=2])
bb=table( all_pheno[which(all_pheno$Year=="2015" & all_pheno$pop=="Dic2 x Silur" ) , "genotype"] )
bb=names(bb[bb>=2])
length(which(aa%in%bb))
```

```
## [1] 26
```

## Basic features

### Features of controls and parents in 2012 and 2015.

```
AA=all_pheno[ (all_pheno$pop=="Silur" | all_pheno$pop=="Lloyd" | all_pheno$pop=="Dic2" | all_pheno$pop=="pescadou" )  & !is.na(all_pheno$pop) , ]
a=aggregate(AA$NV , by=list(pop=AA$pop , année=AA$Year) , mean , na.rm=T)
b=aggregate(AA$Elisa , by=list(pop=AA$pop , année=AA$Year) , mean , na.rm=T)
c=aggregate(AA$QPCR , by=list(pop=AA$pop , année=AA$Year) , mean , na.rm=T)
d=aggregate(AA$NV , by=list(pop=AA$pop , année=AA$Year) , length)
FINAL=cbind(a , b[,3] , c[,3] , d[,3])
colnames(FINAL)=c("ind","year","SS","Elisa","qPCR","#sample")
```

```
print(xtable(FINAL), type = "html", include.rownames = F , comment=FALSE)
```

| ind | year | SS | Elisa | qPCR | #sample |
| --- | --- | --- | --- | --- | --- |
| Dic2 | 2012 | 3.00 | 1.01 |  | 1 |
| Lloyd | 2012 | 4.50 | 0.92 |  | 5 |
| pescadou | 2012 | 3.45 | 1.28 |  | 149 |
| Silur | 2012 | 4.12 | 0.97 |  | 4 |
| Dic2 | 2015 | 1.50 | 0.21 | 3.47 | 2 |
| Lloyd | 2015 | 4.67 | 1.08 | 13.87 | 3 |
| pescadou | 2015 | 4.03 | 1.02 | 14.47 | 100 |
| Silur | 2015 | 4.00 | 1.00 | 15.42 | 2 |

### Features of DS and DL populations in 2012 and 2015.

calculation of Mean, Min, Max and CV for RILS in 2012 and 2015, for SS, Elisa and qPCR

#### SS

```
# Symptome severity
AA=all_pheno[  (all_pheno$pop=="Dic2 x Lloyd" | all_pheno$pop=="Dic2 x Silur"  )  & !is.na(all_pheno$pop) , ]
a=aggregate(AA$NV , by=list(pop=AA$pop , année=AA$Year) , mean , na.rm=T)
b=aggregate(AA$NV , by=list(pop=AA$pop , année=AA$Year) , min , na.rm=T)
c=aggregate(AA$NV , by=list(pop=AA$pop , année=AA$Year) , max , na.rm=T)
d=aggregate(AA$NV , by=list(pop=AA$pop , année=AA$Year) , function(x){sd(x , na.rm=T) / mean(x , na.rm=T) *100 } )
FINAL=cbind(a , b[,3] , c[,3], d[,3])
colnames(FINAL)=c("ind","year","mean","min","max","CV" )
```

```
print(xtable(FINAL), type = "html", include.rownames = F , comment=FALSE)
```

| ind | year | mean | min | max | CV |
| --- | --- | --- | --- | --- | --- |
| Dic2 x Lloyd | 2012 | 2.28 | 0.00 | 4.50 | 47.57 |
| Dic2 x Silur | 2012 | 2.08 | 0.00 | 5.00 | 51.15 |
| Dic2 x Lloyd | 2015 | 2.48 | 0.00 | 5.00 | 55.70 |
| Dic2 x Silur | 2015 | 2.07 | 0.00 | 5.00 | 66.28 |

#### ELISA

```
# Elisa
AA=all_pheno[  (all_pheno$pop=="Dic2 x Lloyd" | all_pheno$pop=="Dic2 x Silur"  )  & !is.na(all_pheno$pop) , ]
a=aggregate(AA$Elisa , by=list(pop=AA$pop , année=AA$Year) , mean , na.rm=T)
b=aggregate(AA$Elisa , by=list(pop=AA$pop , année=AA$Year) , min , na.rm=T)
c=aggregate(AA$Elisa , by=list(pop=AA$pop , année=AA$Year) , max , na.rm=T)
d=aggregate(AA$Elisa , by=list(pop=AA$pop , année=AA$Year) , function(x){sd(x , na.rm=T) / mean(x , na.rm=T) *100 } )
FINAL=cbind(a , b[,3] , c[,3], d[,3])
colnames(FINAL)=c("ind","year","mean","min","max","CV" )
```

```
print(xtable(FINAL), type = "html", include.rownames = F , comment=FALSE)
```

| ind | year | mean | min | max | CV |
| --- | --- | --- | --- | --- | --- |
| Dic2 x Lloyd | 2012 | 0.87 | 0.03 | 2.39 | 53.91 |
| Dic2 x Silur | 2012 | 0.87 | 0.04 | 2.76 | 56.19 |
| Dic2 x Lloyd | 2015 | 0.72 | 0.01 | 1.39 | 59.31 |
| Dic2 x Silur | 2015 | 0.62 | 0.01 | 1.35 | 71.30 |

#### qPCR

```
# qPCR
AA=all_pheno[  (all_pheno$pop=="Dic2 x Lloyd" | all_pheno$pop=="Dic2 x Silur"  )  & !is.na(all_pheno$pop) & all_pheno$Year=="2015" , ]
a=aggregate(AA$QPCR , by=list(pop=AA$pop , année=AA$Year) , mean , na.rm=T)
b=aggregate(AA$QPCR , by=list(pop=AA$pop , année=AA$Year) , min , na.rm=T)
c=aggregate(AA$QPCR , by=list(pop=AA$pop , année=AA$Year) , max , na.rm=T)
d=aggregate(AA$QPCR , by=list(pop=AA$pop , année=AA$Year) , function(x){sd(x , na.rm=T) / mean(x , na.rm=T) *100 } )
FINAL=cbind(a , b[,3] , c[,3], d[,3])
colnames(FINAL)=c("ind","year","mean","min","max","CV" )
```

```
print(xtable(FINAL), type = "html", include.rownames = F , comment=FALSE)
```

| ind | year | mean | min | max | CV |
| --- | --- | --- | --- | --- | --- |
| Dic2 x Lloyd | 2015 | 10.06 | -1.98 | 18.45 | 49.87 |
| Dic2 x Silur | 2015 | 8.83 | -4.57 | 17.55 | 56.90 |

# 3/ Relationship between phenotyping methods

Pearson correlation between SS, Elisa and qPCR in 2012 and 2015?

```
#Compute only between SS and Elisa
AA=all_pheno[  !is.na(all_pheno$Year) & all_pheno$Year=="2012" , ]
cor(AA$NV , AA$Elisa, use="complete.obs")
```

```
## [1] 0.526052
```

```
#I can also do a graphical representation:
boxplot(AA$Elisa  ~ AA$NV)
```

For 2015:

```
# I have 3 comparisons to do:
AA=all_pheno[  !is.na(all_pheno$Year) & all_pheno$Year=="2015" , ]
cor(AA$NV , AA$Elisa, use="complete.obs")
```

```
## [1] 0.6561641
```

```
cor(AA$NV , AA$QPCR, use="complete.obs")
```

```
## [1] 0.5261549
```

```
cor(AA$Elisa , AA$QPCR, use="complete.obs")
```

```
## [1] 0.7236596
```

Correlation between agronomical traits and resistance (SS)

```
#For DS & precocity
for(i in list(Y_DS$NV_blup_AR1_2012,Y_DS$ELISA_blup_AR1_2012 , Y_DS$NV_blup_AR1_2015 , Y_DS$ELISA_blup_AR1_2015 , Y_DS$QPCR_blup_AR1_2015 ) ){
  print(cor( i , Y_DS$epi2013 , use="complete.obs"))
}
```

```
## [1] 0.01490834
## [1] 0.1293095
## [1] 0.1384685
## [1] 0.130189
## [1] 0.05490634
```

```
#For DS & height
for(i in list(Y_DS$NV_blup_AR1_2012,Y_DS$ELISA_blup_AR1_2012 , Y_DS$NV_blup_AR1_2015 , Y_DS$ELISA_blup_AR1_2015 , Y_DS$QPCR_blup_AR1_2015 ) ){
  print(cor( i , Y_DS$Ht2013 , use="complete.obs"))
}
```

```
## [1] -0.1504971
## [1] -0.1537651
## [1] -0.2348963
## [1] -0.1691582
## [1] -0.1890344
```

```
#For DL & precocity
for(i in list(Y_DL$NV_blup_AR1_2012,Y_DL$ELISA_blup_AR1_2012 , Y_DL$NV_blup_AR1_2015 , Y_DL$ELISA_blup_AR1_2015 , Y_DL$QPCR_blup_AR1_2015 ) ){
  print(cor( i , Y_DL$epi , use="complete.obs"))
}
```

```
## [1] 0.006831426
## [1] -0.1099837
## [1] 0.0264017
## [1] -0.1004984
## [1] 0.02329141
```

```
#For DS & height
for(i in list(Y_DL$NV_blup_AR1_2012, Y_DL$ELISA_blup_AR1_2012 , Y_DL$NV_blup_AR1_2015 , Y_DL$ELISA_blup_AR1_2015 , Y_DL$QPCR_blup_AR1_2015 ) ){
  print(cor( i , Y_DL$Ht , use="complete.obs"))
}
```

```
## [1] -0.07930496
## [1] 0.0757165
## [1] -0.1276028
## [1] 0.08256291
## [1] 0.03032634
```

# 4/ QTL representation

The LOD threshold is 3.61:

```
my_lod_seuil=3.61
```

## Summary table

### Both populations together

Let’s build a table that summarizes all significant QTLs:

```
#data
variables=c("NV_blup_AR1_2012", "NV_blup_AR1_2015", "ELISA_blup_AR1_2012",  "ELISA_blup_AR1_2015", "QPCR_blup_AR1_2015")
BOTH=BOTH[ order(BOTH$LG,BOTH$Distance) , ]
BOTH=BOTH[which(BOTH$variable%in%variables) , ]

# Préparation des paramètres
xchro=c(seq(5,15,5) )
chromosomes=c("2A","7A","7B")
size_IC=1.5

#Création d'un tableau bilan
bil=data.frame(matrix(0,30,9))
colnames(bil)=c("pop","carac","chromo","LOD_max","position","marker","IC","R2","a")
    
#Boucle pour les 5 variable d'intéret + les 3 chromosomes d'intéret
data=BOTH
num_line_bilan=num_line_bilan=0
num_chromo=0
for(chrom in chromosomes){
    num_variable=0
    num_col=0
    num_chromo=num_chromo+1

    for(var in variables){
        num_col=num_col+1
        current_data=data[ data$variable==var & data$LG==chrom  , ]
        current_data=current_data[!is.na(current_data$LOD) , ]
        signif_data=current_data[current_data$LOD>my_lod_seuil  , ]
        
        #Si j'ai des marqueurs significatifs
        if(nrow(signif_data)>0){
            
            #Je récupère les infos de ce QTL
            LOD_max=max(signif_data$LOD)
            mark_max=signif_data$marqueur[signif_data$LOD==LOD_max]
            pos_max=signif_data$Distance[signif_data$LOD==LOD_max]
            r2_max=signif_data$R2[signif_data$LOD==LOD_max]
            a_max=signif_data$a[signif_data$LOD==LOD_max]

            #Détermination IC --> je bloque la zone max a 30 cM
            in_IC=current_data[ current_data$LOD > (LOD_max-size_IC) & current_data$Distance > (pos_max-30) & current_data$Distance < (pos_max+30)  , ]
            IC_min=min(in_IC$Distance)
            IC_max=max(in_IC$Distance)
            
            #Je remplie le tableau bilan
            num_line_bilan=num_line_bilan+1
            bil[num_line_bilan , 1]="BOTH"
            bil[num_line_bilan , 2]=var
            bil[num_line_bilan , 3]=chrom
            bil[num_line_bilan , 4]=LOD_max
            bil[num_line_bilan , 5]=pos_max[1]
            bil[num_line_bilan , 6]=as.character(mark_max)[1]
            bil[num_line_bilan , 7]=paste(IC_min, IC_max, sep="-")
            bil[num_line_bilan , 8]=r2_max[1]
            bil[num_line_bilan , 9]=a_max[1]
            
            }
}}
    

# Clean and print the Table
bil=bil[bil$pop!=0 , ]
bil=bil[ order(bil$pop , bil$chromo , bil$carac) , ]
```

```
print(xtable(bil), type = "html", include.rownames = F , comment=FALSE)
```

| pop | carac | chromo | LOD\_max | position | marker | IC | R2 | a |
| --- | --- | --- | --- | --- | --- | --- | --- | --- |
| BOTH | ELISA\_blup\_AR1\_2015 | 2A | 4.46 | 227.10 | Cluster\_268|Contig4|original@2519 | 218.8-227.1 | 4.75 | 0.07 |
| BOTH | NV\_blup\_AR1\_2012 | 2A | 3.61 | 214.70 | Cluster\_8997|Contig2|original@993 | 214.7-218.8 | 3.80 | 0.17 |
| BOTH | NV\_blup\_AR1\_2015 | 2A | 5.82 | 214.70 | Cluster\_8997|Contig2|original@993 | 214.7-218.8 | 6.29 | 0.29 |
| BOTH | ELISA\_blup\_AR1\_2012 | 7A | 9.50 | 115.50 | Cluster\_11075|Contig2|original@652 | 115.5-119.4 | 10.42 | 0.09 |
| BOTH | ELISA\_blup\_AR1\_2015 | 7A | 7.42 | 116.90 | Cluster\_5672|Contig1|likelySeq@976 | 115.5-120 | 8.08 | 0.13 |
| BOTH | NV\_blup\_AR1\_2012 | 7A | 5.00 | 116.90 | Cluster\_5672|Contig1|likelySeq@976 | 115.5-117.7 | 5.42 | 0.22 |
| BOTH | NV\_blup\_AR1\_2015 | 7A | 5.71 | 116.90 | Cluster\_5672|Contig1|likelySeq@976 | 116.9-117.7 | 6.17 | 0.28 |
| BOTH | ELISA\_blup\_AR1\_2012 | 7B | 8.69 | 54.40 | Cluster\_8668|Contig2|original@192 | 54.4-57.9 | 9.56 | 0.08 |
| BOTH | ELISA\_blup\_AR1\_2015 | 7B | 8.19 | 54.40 | Cluster\_8668|Contig2|original@192 | 54.4-57.9 | 8.92 | 0.12 |
| BOTH | NV\_blup\_AR1\_2012 | 7B | 5.32 | 57.90 | Cluster\_3623|Contig2|original@924 | 57.9-59.3 | 5.79 | 0.24 |
| BOTH | NV\_blup\_AR1\_2015 | 7B | 5.85 | 57.90 | Cluster\_3623|Contig2|original@924 | 57.9-59.3 | 6.32 | 0.32 |
| BOTH | QPCR\_blup\_AR1\_2015 | 7B | 6.93 | 57.90 | Cluster\_3623|Contig2|original@924 | 54.4-59.3 | 7.73 | 1.00 |

### Pop by pop

```
#data
variables=c("NV_blup_AR1_2012", "NV_blup_AR1_2015", "ELISA_blup_AR1_2012",  "ELISA_blup_AR1_2015", "QPCR_blup_AR1_2015")
DS=DS[ order(DS$LG,DS$Distance) , ]
DS=DS[which(DS$variable%in%variables) , ]
DL=DL[ order(DL$LG,DL$Distance) , ]
DL=DL[which(DL$variable%in%variables) , ]

# Préparation des paramètres
xchro=c(seq(5,15,5) )
chromosomes=c("2A","7A","7B")
size_IC=1.5

#Création d'un tableau bilan
bil=data.frame(matrix(0,30,9))
colnames(bil)=c("pop","carac","chromo","LOD_max","position","marker","IC","R2","a")

# loop
num_line_bilan=0
for(pop in list(DS,DL)){
    data=pop
    num_chromo=0

    for(chrom in chromosomes){
        num_variable=0
        num_col=0
        num_chromo=num_chromo+1

        for(var in variables){
            num_col=num_col+1
            current_data=data[ data$variable==var & data$LG==chrom  , ]
            current_data=current_data[!is.na(current_data$LOD) , ]
            signif_data=current_data[current_data$LOD>my_lod_seuil  , ]
            
            #Si j'ai des marqueurs significatifs
            if(nrow(signif_data)>0){
                
                #Je récupère les infos de ce QTL
                LOD_max=max(signif_data$LOD)
                mark_max=signif_data$marqueur[signif_data$LOD==LOD_max]
                pos_max=signif_data$Distance[signif_data$LOD==LOD_max]
                r2_max=signif_data$R2[signif_data$LOD==LOD_max]
                a_max=signif_data$a[signif_data$LOD==LOD_max]
    
                #Détermination IC --> je bloque la zone max a 30 cM
                in_IC=current_data[ current_data$LOD > (LOD_max-size_IC) & current_data$Distance > (pos_max-30) & current_data$Distance < (pos_max+30)  , ]
                IC_min=min(in_IC$Distance)
                IC_max=max(in_IC$Distance)
                
                #Je remplie le tableau bilan
                num_line_bilan=num_line_bilan+1
                bil[num_line_bilan , 1]=ifelse(identical(pop,DS) , "DS" , "DL")
                bil[num_line_bilan , 2]=var
                bil[num_line_bilan , 3]=chrom
                bil[num_line_bilan , 4]=LOD_max
                bil[num_line_bilan , 5]=pos_max[1]
                bil[num_line_bilan , 6]=as.character(mark_max)[1]
                bil[num_line_bilan , 7]=paste(IC_min, IC_max, sep="-")
                bil[num_line_bilan , 8]=r2_max[1]
                bil[num_line_bilan , 9]=a_max[1]
                
                }
}}}
```

```
## Warning in current_data$Distance > (pos_max - 30): la taille d'un objet
## plus long n'est pas multiple de la taille d'un objet plus court
```

```
## Warning in current_data$Distance < (pos_max + 30): la taille d'un objet
## plus long n'est pas multiple de la taille d'un objet plus court
```

```
## Warning in current_data$Distance > (pos_max - 30): la taille d'un objet
## plus long n'est pas multiple de la taille d'un objet plus court
```

```
## Warning in current_data$Distance < (pos_max + 30): la taille d'un objet
## plus long n'est pas multiple de la taille d'un objet plus court
```

```
# Clean the Table
bil=bil[bil$pop!=0 , ]
bil=bil[ order(bil$pop , bil$chromo , bil$carac) , ]
```

```
print(xtable(bil), type = "html", include.rownames = F , comment=FALSE)
```

| pop | carac | chromo | LOD\_max | position | marker | IC | R2 | a |
| --- | --- | --- | --- | --- | --- | --- | --- | --- |
| DL | NV\_blup\_AR1\_2015 | 2A | 3.95 | 215.20 | Cluster\_15067|Contig1|likelySeq@499 | 214.7-218.8 | 7.54 | 0.25 |
| DL | ELISA\_blup\_AR1\_2012 | 7A | 6.30 | 119.00 | Cluster\_926|Contig2|original@498 | 112.8-126 | 12.37 | 0.09 |
| DL | ELISA\_blup\_AR1\_2015 | 7A | 6.20 | 116.90 | Cluster\_5672|Contig1|likelySeq@976 | 116-127.4 | 11.95 | 0.15 |
| DL | NV\_blup\_AR1\_2012 | 7A | 3.69 | 117.20 | Cluster\_13379|Contig1|likelySeq@256 | 115.5-140.1 | 7.15 | 0.25 |
| DL | NV\_blup\_AR1\_2015 | 7A | 4.79 | 116.90 | Cluster\_5672|Contig1|likelySeq@976 | 116.9-132.1 | 9.23 | 0.30 |
| DL | QPCR\_blup\_AR1\_2015 | 7A | 4.20 | 123.00 | Cluster\_10741|Contig3|complementarySeq@307 | 115.5-140.6 | 8.22 | 1.04 |
| DL | ELISA\_blup\_AR1\_2012 | 7B | 4.09 | 57.90 | Cluster\_3623|Contig2|original@924 | 54.1-60 | 7.97 | 0.07 |
| DL | ELISA\_blup\_AR1\_2015 | 7B | 5.51 | 54.40 | Cluster\_8668|Contig1|original@1027 | 54.4-54.4 | 10.62 | 0.12 |
| DL | NV\_blup\_AR1\_2012 | 7B | 3.82 | 59.00 | Traes\_7BS\_1101C1585@760 | 57.9-60 | 7.41 | 0.27 |
| DL | NV\_blup\_AR1\_2015 | 7B | 4.06 | 57.90 | Cluster\_3623|Contig2|original@924 | 31.7-59.3 | 7.75 | 0.35 |
| DL | QPCR\_blup\_AR1\_2015 | 7B | 4.71 | 57.90 | Cluster\_3623|Contig2|original@924 | 54.1-60 | 9.24 | 1.00 |
| DS | NV\_blup\_AR1\_2015 | 2A | 4.29 | 193.00 | Cluster\_12235|Contig1|likelySeq@801 | 187.7-218.8 | 9.35 | 0.39 |
| DS | ELISA\_blup\_AR1\_2012 | 7A | 5.16 | 119.00 | Cluster\_926|Contig2|original@498 | 115.1-120.5 | 11.28 | 0.08 |
| DS | ELISA\_blup\_AR1\_2015 | 7A | 4.70 | 119.00 | Cluster\_926|Contig2|original@498 | 115.5-120.5 | 10.28 | 0.11 |
| DS | ELISA\_blup\_AR1\_2012 | 7B | 6.64 | 54.40 | Cluster\_8668|Contig2|original@192 | 46.8-54.4 | 14.42 | 0.09 |
| DS | ELISA\_blup\_AR1\_2015 | 7B | 5.26 | 57.90 | Cluster\_3623|Contig2|original@924 | 54.4-59.3 | 11.49 | 0.13 |
| DS | NV\_blup\_AR1\_2012 | 7B | 6.85 | 19.60 | Cluster\_6229|Contig1|original@215 | 19-19.6 | 14.84 | 0.31 |
| DS | NV\_blup\_AR1\_2015 | 7B | 3.73 | 46.80 | Cluster\_2114|Contig3|original@2227 | 19-68 | 8.07 | 0.29 |
| DS | QPCR\_blup\_AR1\_2015 | 7B | 4.16 | 54.40 | Cluster\_8668|Contig2|original@192 | 46.8-78.5 | 9.33 | 1.11 |

## Visualize LOD-scores

### Both populations together

This is the figure 3 of the paper:

```
#Charge data
BOTH=BOTH[ order(BOTH$LG,BOTH$Distance) , ]

#Output
par(mfrow=c(1,2))
my_colors=brewer.pal(6,"Paired")

#BOTH 7A
par(mar=c(5,5,2,0))
a=BOTH[BOTH$variable=="NV_blup_AR1_2012" & BOTH$LG=="7A" & !is.na(BOTH$LOD) , ] ; plot(a$LOD ~ a$Distance , type="l" , ylim=c(0,10) , ylab="LOD score"  , col=my_colors[1] , lwd=1.6, xlab="" )
abline(h=3.61 , col="grey")
a=BOTH[BOTH$variable=="ELISA_blup_AR1_2012"  & BOTH$LG=="7A" & !is.na(BOTH$LOD) , ] ; points(a$LOD ~ a$Distance , type="l" , ylim=c(0,10) , ylab="LOD", col=my_colors[3], lwd=1.6)
a=BOTH[BOTH$variable=="NV_blup_AR1_2015" & BOTH$LG=="7A" & !is.na(BOTH$LOD) , ] ; points(a$LOD ~ a$Distance , type="l" , ylim=c(0,10) , ylab="LOD" , col=my_colors[2], lwd=1.6)
a=BOTH[BOTH$variable=="ELISA_blup_AR1_2015" & BOTH$LG=="7A" & !is.na(BOTH$LOD) , ] ; points(a$LOD ~ a$Distance , type="l" , ylim=c(0,10) , ylab="LOD" , col=my_colors[4], lwd=1.6)
a=BOTH[BOTH$variable=="QPCR_blup_AR1_2015" & BOTH$LG=="7A" & !is.na(BOTH$LOD) , ] ; points(a$LOD ~ a$Distance , type="l" , ylim=c(0,10) , ylab="LOD" , col=my_colors[6], lwd=1.6)
mtext("position on chromosome 7A (cM)" , side=1 , line=3 , col="black" , cex=1)
#Ajout légende
legend("topright" , horiz=F , col=my_colors[c(1,3,2,4,6)] , legend=c("SS 2012" , "Elisa 2012" , "SS 2015", "Elisa 2015" , "qPCR 2015") , bty="n" , lty=1 , pt.cex=2, lwd=1.6)

#BOTH 7B
par(mar=c(5,1,2,4))
a=BOTH[BOTH$variable=="NV_blup_AR1_2012" & BOTH$LG=="7B" & !is.na(BOTH$LOD) , ] ; plot(a$LOD ~ a$Distance , type="l" , ylim=c(0,10) , ylab="" , col=my_colors[1] , yaxt="n", lwd=1.6 , xlab="")
abline(h=3.61 , col="grey")
a=BOTH[BOTH$variable=="ELISA_blup_AR1_2012"  & BOTH$LG=="7B" & !is.na(BOTH$LOD) , ] ; points(a$LOD ~ a$Distance , type="l" , ylim=c(0,10) , ylab="LOD", col=my_colors[3], lwd=1.6)
a=BOTH[BOTH$variable=="NV_blup_AR1_2015" & BOTH$LG=="7B" & !is.na(BOTH$LOD) , ] ; points(a$LOD ~ a$Distance , type="l" , ylim=c(0,10) , ylab="LOD" , col=my_colors[2], lwd=1.6)
a=BOTH[BOTH$variable=="ELISA_blup_AR1_2015" & BOTH$LG=="7B" & !is.na(BOTH$LOD) , ] ; points(a$LOD ~ a$Distance , type="l" , ylim=c(0,10) , ylab="LOD" , col=my_colors[4], lwd=1.6)
a=BOTH[BOTH$variable=="QPCR_blup_AR1_2015" & BOTH$LG=="7B" & !is.na(BOTH$LOD) , ] ; points(a$LOD ~ a$Distance , type="l" , ylim=c(0,10) , ylab="LOD" , col=my_colors[6], lwd=1.6)
mtext("position on chromosome 7B (cM)" , side=1 , line=3 , col="black" , cex=1)
```

### Populations one by one

There is the representation of QTLs found on chromosome 7A and 7B for populations DS and DL separately (Fig3 in the paper.)

```
# Order data:
DS=DS[ order(DS$LG,DS$Distance) , ]
DL=DL[ order(DL$LG,DL$Distance) , ]

#Make the plot
par(mfrow=c(2,2))
my_colors=brewer.pal(6,"Paired")

#DS 7A
par(mar=c(0,5,2,0))
a=DS[DS$variable=="NV_blup_AR1_2012" & DS$LG=="7A" & !is.na(DS$LOD) , ] ; plot(a$LOD ~ a$Distance , type="l" , ylim=c(0,7) , ylab="LOD score" , xaxt="n" , col=my_colors[1] , lwd=1.6 )
abline(h=3 , col="grey")
a=DS[DS$variable=="ELISA_blup_AR1_2012"  & DS$LG=="7A" & !is.na(DS$LOD) , ] ; points(a$LOD ~ a$Distance , type="l" , ylim=c(0,10) , ylab="LOD", col=my_colors[3], lwd=1.6)
a=DS[DS$variable=="NV_blup_AR1_2015" & DS$LG=="7A" & !is.na(DS$LOD) , ] ; points(a$LOD ~ a$Distance , type="l" , ylim=c(0,10) , ylab="LOD" , col=my_colors[2], lwd=1.6)
a=DS[DS$variable=="ELISA_blup_AR1_2015" & DS$LG=="7A" & !is.na(DS$LOD) , ] ; points(a$LOD ~ a$Distance , type="l" , ylim=c(0,10) , ylab="LOD" , col=my_colors[4], lwd=1.6)
a=DS[DS$variable=="QPCR_blup_AR1_2015" & DS$LG=="7A" & !is.na(DS$LOD) , ] ; points(a$LOD ~ a$Distance , type="l" , ylim=c(0,10) , ylab="LOD" , col=my_colors[6], lwd=1.6)
legend("topleft", "Dic2 x Silur" , col="black" , cex=1.2 , bty="n")
#Ajout légende
legend("topright" , horiz=F , col=my_colors[c(1,3,2,4,6)] , legend=c("SS 2012" , "Elisa 2012" , "SS 2015", "Elisa 2015" , "qPCR 2015") , bty="n" , lty=1 , pt.cex=2, lwd=1.6)

#DS 7B
par(mar=c(0,1,2,4))
a=DS[DS$variable=="NV_blup_AR1_2012" & DS$LG=="7B" & !is.na(DS$LOD) , ] ; plot(a$LOD ~ a$Distance , type="l" , ylim=c(0,7) , ylab="" , xaxt="n" , col=my_colors[1] , yaxt="n", lwd=1.6)
abline(h=3 , col="grey")
a=DS[DS$variable=="ELISA_blup_AR1_2012"  & DS$LG=="7B" & !is.na(DS$LOD) , ] ; points(a$LOD ~ a$Distance , type="l" , ylim=c(0,10) , ylab="LOD", col=my_colors[3], lwd=1.6)
a=DS[DS$variable=="NV_blup_AR1_2015" & DS$LG=="7B" & !is.na(DS$LOD) , ] ; points(a$LOD ~ a$Distance , type="l" , ylim=c(0,10) , ylab="LOD" , col=my_colors[2], lwd=1.6)
a=DS[DS$variable=="ELISA_blup_AR1_2015" & DS$LG=="7B" & !is.na(DS$LOD) , ] ; points(a$LOD ~ a$Distance , type="l" , ylim=c(0,10) , ylab="LOD" , col=my_colors[4], lwd=1.6)
a=DS[DS$variable=="QPCR_blup_AR1_2015" & DS$LG=="7B" & !is.na(DS$LOD) , ] ; points(a$LOD ~ a$Distance , type="l" , ylim=c(0,10) , ylab="LOD" , col=my_colors[6], lwd=1.6)
legend("topleft", "Dic2 x Lloyd" , col="black" , cex=1.2 , bty="n")

#DL 7A
par(mar=c(5,5,1,0))
a=DL[DL$variable=="NV_blup_AR1_2012" & DL$LG=="7A" & !is.na(DL$LOD) , ] ; plot(a$LOD ~ a$Distance , type="l" , ylim=c(0,7) , ylab="LOD score", xlab="" , col=my_colors[1], lwd=1.6 )
abline(h=3 , col="grey")
a=DL[DL$variable=="ELISA_blup_AR1_2012"  & DL$LG=="7A" & !is.na(DL$LOD) , ] ; points(a$LOD ~ a$Distance , type="l" , ylim=c(0,10) , ylab="LOD", col=my_colors[3], lwd=1.6)
a=DL[DL$variable=="NV_blup_AR1_2015" & DL$LG=="7A" & !is.na(DL$LOD) , ] ; points(a$LOD ~ a$Distance , type="l" , ylim=c(0,10) , ylab="LOD" , col=my_colors[2], lwd=1.6)
a=DL[DL$variable=="ELISA_blup_AR1_2015" & DL$LG=="7A" & !is.na(DL$LOD) , ] ; points(a$LOD ~ a$Distance , type="l" , ylim=c(0,10) , ylab="LOD" , col=my_colors[4], lwd=1.6)
a=DL[DL$variable=="QPCR_blup_AR1_2015" & DL$LG=="7A" & !is.na(DL$LOD) , ] ; points(a$LOD ~ a$Distance , type="l" , ylim=c(0,10) , ylab="LOD" , col=my_colors[6], lwd=1.6)
mtext("position on chromosome 7A (cM)" , side=1 , line=3 , col="black" , cex=1)
legend("topleft", "Dic2 x Silur" , col="black" , cex=1.2 , bty="n")

#DL 7B
par(mar=c(5,1,1,4))
a=DL[DL$variable=="NV_blup_AR1_2012" & DL$LG=="7B" & !is.na(DL$LOD) , ] ; plot(a$LOD ~ a$Distance , type="l" , ylim=c(0,7) , ylab="" , xlab=""  , col=my_colors[1] , yaxt="n", lwd=1.6)
abline(h=3 , col="grey")
a=DL[DL$variable=="ELISA_blup_AR1_2012"  & DL$LG=="7B" & !is.na(DL$LOD) , ] ; points(a$LOD ~ a$Distance , type="l" , ylim=c(0,10) , ylab="LOD", col=my_colors[3], lwd=1.6)
a=DL[DL$variable=="NV_blup_AR1_2015" & DL$LG=="7B" & !is.na(DL$LOD) , ] ; points(a$LOD ~ a$Distance , type="l" , ylim=c(0,10) , ylab="LOD" , col=my_colors[2], lwd=1.6)
a=DL[DL$variable=="ELISA_blup_AR1_2015" & DL$LG=="7B" & !is.na(DL$LOD) , ] ; points(a$LOD ~ a$Distance , type="l" , ylim=c(0,10) , ylab="LOD" , col=my_colors[4], lwd=1.6)
a=DL[DL$variable=="QPCR_blup_AR1_2015" & DL$LG=="7B" & !is.na(DL$LOD) , ] ; points(a$LOD ~ a$Distance , type="l" , ylim=c(0,10) , ylab="LOD" , col=my_colors[6], lwd=1.6)
mtext("position on chromosome 7B (cM)" , side=1 , line=3 , col="black" , cex=1)
legend("topleft", "Dic2 x Lloyd" , col="black" , cex=1.2 , bty="n")
```

## Visualize IC

There is the representation of every significant QTLs found (chromosome 7A and 7B and 2A) for DS and DL (Fig2 in the paper.). In this part we determine and show the IC of each QTL. We show it for the 2 pops separately only:

```
# Variable I am going to represent:
variables=c("NV_blup_AR1_2012", "NV_blup_AR1_2015", "ELISA_blup_AR1_2012",  "ELISA_blup_AR1_2015", "QPCR_blup_AR1_2015")
DS=DS[which(DS$variable%in%variables) , ]
DL=DL[which(DL$variable%in%variables) , ]

# Set parameters of graphic:
my_colors=brewer.pal(5,"Paired")
xchro=c(seq(5,15,5) )
chromosomes=c("2A","7A","7B")
size_IC=1.5

# Initiate a summary file (Supplementary data)
bil=data.frame(matrix(0,30,9))
colnames(bil)=c("pop","carac","chromo","LOD_max","position","marker","IC","R2","a")

#Mise en place du fond de carte:
par(mar=c(0,5,2,1))
plot(1,1,col="transparent",bty="n", xaxt="n", yaxt="l" , xlab="", ylab="position in cM" , xlim=c(3,max(xchro)+3) , ylim=c(320,-5) )
num=0
for(chrom in chromosomes){
    num=num+1
    A=DS[DS$variable=="NV_blup_AR1_2012" & DS$LG==chrom, ]
    points(rep(xchro[num],nrow(A)) , A$Distance , pch=20 , cex=0.8 , col="grey")
    }
    
#Boucle pour les 5 variable d'intéret + les 3 chromosomes d'intéret + Les 2 pops
num_line_bilan=0
for(pop in list(DS,DL)){
    data=pop
    num_chromo=0

    for(chrom in chromosomes){
        num_variable=0
        num_col=0
        num_chromo=num_chromo+1

        for(var in variables){
            num_col=num_col+1
            current_data=data[ data$variable==var & data$LG==chrom  , ]
            current_data=current_data[!is.na(current_data$LOD) , ]
            signif_data=current_data[current_data$LOD>my_lod_seuil  , ]
            
            #Si j'ai des marqueurs significatifs
            if(nrow(signif_data)>0){
                
                #Je récupère les infos de ce QTL
                LOD_max=max(signif_data$LOD)
                mark_max=signif_data$marqueur[signif_data$LOD==LOD_max]
                pos_max=signif_data$Distance[signif_data$LOD==LOD_max]
                r2_max=signif_data$R2[signif_data$LOD==LOD_max]
                a_max=signif_data$a[signif_data$LOD==LOD_max]
    
                #Détermination IC --> je bloque la zone max a 30 cM
                in_IC=current_data[ current_data$LOD > (LOD_max-size_IC) & current_data$Distance > (pos_max-30) & current_data$Distance < (pos_max+30)  , ]
                IC_min=min(in_IC$Distance)
                IC_max=max(in_IC$Distance)
                
                #Je remplie le tableau bilan
                num_line_bilan=num_line_bilan+1
                bil[num_line_bilan , 1]=ifelse(identical(pop,DS) , "DS" , "DL")
                bil[num_line_bilan , 2]=var
                bil[num_line_bilan , 3]=chrom
                bil[num_line_bilan , 4]=LOD_max
                bil[num_line_bilan , 5]=pos_max[1]
                bil[num_line_bilan , 6]=as.character(mark_max)[1]
                bil[num_line_bilan , 7]=paste(IC_min, IC_max, sep="-")
                bil[num_line_bilan , 8]=r2_max[1]
                bil[num_line_bilan , 9]=a_max[1]
                
                #J'ajoute le trait corresponant a ma variable
                num_variable=num_variable+0.5
                if(identical(pop,DL)){ num_variable=-abs(num_variable) }
                lines(c(xchro[num_chromo]+num_variable,xchro[num_chromo]+num_variable) , c(IC_min,IC_max) , col=my_colors[num_col], lwd=6)
                num_variable=abs(num_variable)
                }
}}}
```

```
## Warning in current_data$Distance > (pos_max - 30): la taille d'un objet
## plus long n'est pas multiple de la taille d'un objet plus court
```

```
## Warning in current_data$Distance < (pos_max + 30): la taille d'un objet
## plus long n'est pas multiple de la taille d'un objet plus court
```

```
## Warning in current_data$Distance > (pos_max - 30): la taille d'un objet
## plus long n'est pas multiple de la taille d'un objet plus court
```

```
## Warning in current_data$Distance < (pos_max + 30): la taille d'un objet
## plus long n'est pas multiple de la taille d'un objet plus court
```

```
#Ajout légende et nom de chromosome?
text(xchro , rep(-10,3) , chromosomes , col="orange")
legend(14 , 240 , horiz=F , col=my_colors , legend=c("SS 2012" , "SS 2015", "Elisa 2012" , "Elisa 2015" , "qPCR 2015") , bty="n" , lty=1 , lwd=6 )
```

```
# Clean and print the Table
bil=bil[bil$pop!=0 , ]
bil=bil[ order(bil$pop , bil$chromo , bil$carac) , ]

# Show the supplementary data
print(head(bil))
```

```
##    pop               carac chromo  LOD_max position
## 9   DL    NV_blup_AR1_2015     2A 3.951210    215.2
## 12  DL ELISA_blup_AR1_2012     7A 6.303916    119.0
## 13  DL ELISA_blup_AR1_2015     7A 6.204936    116.9
## 10  DL    NV_blup_AR1_2012     7A 3.689640    117.2
## 11  DL    NV_blup_AR1_2015     7A 4.792257    116.9
## 14  DL  QPCR_blup_AR1_2015     7A 4.204108    123.0
##                                        marker          IC        R2
## 9         Cluster_15067|Contig1|likelySeq@499 214.7-218.8  7.540279
## 12           Cluster_926|Contig2|original@498   112.8-126 12.373244
## 13         Cluster_5672|Contig1|likelySeq@976   116-127.4 11.950204
## 10        Cluster_13379|Contig1|likelySeq@256 115.5-140.1  7.152917
## 11         Cluster_5672|Contig1|likelySeq@976 116.9-132.1  9.225176
## 14 Cluster_10741|Contig3|complementarySeq@307 115.5-140.6  8.216540
##             a
## 9  0.25209804
## 12 0.09178861
## 13 0.15469894
## 10 0.25251817
## 11 0.30334781
## 14 1.04368778
```

# 5/ Epistatic effect between QTLs

```
my_fun=function(path, markers, col){
    setwd(path)
    Y=read.table(file = "phenotypage.csv" , header = TRUE, sep = ";", dec = ".", na.strings = "NA")
    colnames(Y)[1]="geno"
    geno <- read.table(file="genotypage.csv" , sep = ";" , header = F, na.strings = "-")
    geno=as.matrix(geno)
    colnames(geno)=geno[1,]
    geno=as.data.frame(geno[-1 , ])
    geno=geno[ , c(1,which(colnames(geno)%in%markers))]
    data=merge(geno , Y , by.x=1 , by.y=1 )
    data=data[ , c(1:3, col)]
    return(data)
    }

# Function to calculate interaction effect between QTLs:
analyse_inter=function(data, res, name){
    
    # Prepare
    data=data[ !is.na(data[,2]) & !is.na(data[,3]) , ]
    my_mark=paste(data[,2],data[,3],sep="-")
    means <- round(tapply(data[,4],my_mark,mean,na.rm=T) ,2)
    
    # Calculate interaction
    model=lm(data[,4] ~ data[,2] * data[,3])
    sum=summary(model)
    tot_r2=round(sum$r.squared,3)
    inter_pval=round(sum$coefficients[4,4],10)
    
    # Complete summary files
    res[num , c(4:9)]=c(means, tot_r2, inter_pval)
    return(res)
}


# ========================
# Préparation d'un tableau bilan:
res=data.frame(matrix(0,4,9))
colnames(res)=c("phenotype","marker_7A_QTL","marker_7B_QTL","A-A","A-B","B-A","B-B","R2_tot","pval_inter")
num=0

# SS 2012
num=num+1
markers=c("Cluster_10461|Contig1|likelySeq@159" ,  "Cluster_932|Contig1|original@517"  )
data=my_fun("~/Dropbox/Publi_Mosaique/DATA/DATA/GROUPED" ,  markers , 4)
res[num , c(1:3)]=c(colnames(data)[4] , markers)
res=analyse_inter(data, res, "SS-2012" )

# SS 2015
num=num+1
markers=c("Cluster_4000|Contig1|likelySeq@264" ,  "Cluster_3623|Contig2|original@924"  )
data=my_fun("~/Dropbox/Publi_Mosaique/DATA/DATA/GROUPED" ,  markers , 6)
res[num , c(1:3)]=c(colnames(data)[4] , markers)
res=analyse_inter(data, res, "SS-2015")

# Elisa 2012
num=num+1
markers=c("Cluster_5672|Contig1|likelySeq@976" ,  "Cluster_3623|Contig2|original@924"  )
data=my_fun("~/Dropbox/Publi_Mosaique/DATA/DATA/GROUPED" ,  markers , 5)
res[num , c(1:3)]=c(colnames(data)[4] , markers)
res=analyse_inter(data, res, "Elisa-2012" )

# Elisa 2015
num=num+1
markers=c("Cluster_4000|Contig1|likelySeq@264" ,  "Cluster_3623|Contig2|original@924"  )
#markers=c("Cluster_4000|Contig1|likelySeq@264" ,  "Cluster_268|Contig4|original@2519"  )
#markers=c("Cluster_268|Contig4|original@2519" ,  "Cluster_3623|Contig2|original@924"  )
data=my_fun("~/Dropbox/Publi_Mosaique/DATA/DATA/GROUPED" ,  markers , 7)
res[num , c(1:3)]=c(colnames(data)[4] , markers)
res=analyse_inter(data, res, "Elisa-2015" )

# Pour Elisa 2015, on va aussi faire la figure 4
data=my_fun("~/Dropbox/Publi_Mosaique/DATA/DATA/GROUPED" ,  markers , 7)
data=data[ !is.na(data[,2]) & !is.na(data[,3]) , ]
my_mark=paste(data[,2],data[,3],sep="-")
means <- round(tapply(data[,4],my_mark,mean,na.rm=T) ,2)
par(mfrow=c(1,1) , mar=c(5,5,2,2))
boxplot(data[,4] ~ my_mark , medlwd=0, cex.axis=0.6, cex.col="grey", las=1, main="" ,ylab="Elisa 2015 BLUP value",  xlab="Bi-locus genotype" , col=rgb(0.3,0.1,0.4,0.2) , xaxt="n", boxwex=0.4) ; 
my_labels=c( expression(paste('R'["7A"],"-",'R'["7B"])) , expression(paste('R'["7A"],"-",'S'["7B"])) ,  expression(paste('S'["7A"],"-",'R'["7B"])) , expression(paste('S'["7A"],"-",'S'["7B"])) )
axis(labels=my_labels , at=c(1,2,3,4) , side=T)
#table(my_mark) if I want to add number of obs
points(seq(1,4), c(-0.25, -0.01 , 0.01 , 0.25  ), pch=17 , col="orange", cex=2)
segments(c(0,0,0,0),c(-0.25, -0.01 , 0.01 , 0.25  ),c(1,2,3,4),c(-0.25, -0.01 , 0.01 , 0.25  ) , lty=3, col="grey")
points(seq(1,4), means, pch=4 , lwd=4 , col="red" , cex=2)
```

```
print(xtable(res), type = "html", include.rownames = F , comment=FALSE)
```

| phenotype | marker\_7A\_QTL | marker\_7B\_QTL | A-A | A-B | B-A | B-B | R2\_tot | pval\_inter |
| --- | --- | --- | --- | --- | --- | --- | --- | --- |
| NV\_blup\_AR1\_2012 | Cluster\_10461|Contig1|likelySeq@159 | Cluster\_932|Contig1|original@517 | -0.58 | 0.19 | 0.14 | 0.28 | 0.21 | 0.00 |
| NV\_blup\_AR1\_2015 | Cluster\_4000|Contig1|likelySeq@264 | Cluster\_3623|Contig2|original@924 | -0.82 | 0.25 | 0.14 | 0.38 | 0.25 | 0.00 |
| ELISA\_blup\_AR1\_2012 | Cluster\_5672|Contig1|likelySeq@976 | Cluster\_3623|Contig2|original@924 | -0.26 | 0.07 | 0.08 | 0.09 | 0.43 | 0.00 |
| ELISA\_blup\_AR1\_2015 | Cluster\_4000|Contig1|likelySeq@264 | Cluster\_3623|Contig2|original@924 | -0.38 | 0.10 | 0.12 | 0.15 | 0.42 | 0.00 |

Yan Holtz, Michel Bonnefoy, Véronique Viader, Morgane Ardisson, Nicolas O. Rode, Gérard Poux, Pierre Roumet, Véronique Marie-Jeanne, Vincent Ranwez, Sylvain Santoni, David Gouache, Jacques L. David

30th May 2016
