# Supplementary material for: Epistatic determinism of durum wheat resistance to the wheat spindle streak mosaic virus
Source: Theor Appl Genet. 2017 Apr 27;130(7):1491–505. doi: 10.1007/s00122-017-2904-6 (PMC5487696; doi:10.1007/s00122-017-2904-6)
Supplement: Supplementary file 4 — Online Resource 4: Data and R scripts for reproducible QTL detection. Data and R script (.csv and.rmd format) are provided in this tar archive. A scheme aims to explain the content of each file and its role in the QTL detection pipeline. The upstream bioinformatic steps (from raw reads to consensus genetic map) are not included (GZ 72829 kb) [file 122_2017_2904_MOESM4_ESM.gz › TMP/SCRIPT/1_Blup_and_Heritability/AnalysisWheatResistance2012-2015_18Aug2016.html]

WheatResistance2012-2015


# WheatResistance2012-2015

#### *Nicolas O. Rode*

#### *Aug, 18th 2016*

# 1 Set working directory

```
setwd("/homedir/rode/Rstudio/Yan/2012-2015/18082016")
list.files()
```

```
##  [1] "AICcTable_ASReml-R_Analyses_2016-08-19Elisa.csv" 
##  [2] "AICcTable_ASReml-R_Analyses_2016-08-19NV.csv"    
##  [3] "AICcTable_ASReml-R_Analyses_2016-08-19qPCR.csv"  
##  [4] "AnalysisWheatResistance2012-2015_18Aug2016_files"
##  [5] "AnalysisWheatResistance2012-2015_18Aug2016.html" 
##  [6] "AnalysisWheatResistance2012-2015_18Aug2016.Rmd"  
##  [7] "BLUPsElisa2012_2015.csv"                         
##  [8] "BLUPsNV2012_2015.csv"                            
##  [9] "BLUPsqPCR2015.csv"                               
## [10] "Data2012_2015.csv"                               
## [11] "Data2012.csv"                                    
## [12] "Data2015.csv"                                    
## [13] "PrepDataWheatResistance2012-2015.html"           
## [14] "PrepDataWheatResistance2012-2015.Rmd"
```

```
## Load Packages
##see: http://stats.stackexchange.com/questions/18709/lme4-or-other-open-source-r-package-code-equivalent-to-asreml-r
library(asreml)
```

```
## Loading required package: lattice
```

```
## Checking for license in  /homedir/rode 
## Licensed to: CIRAD
## Serial Number: 402587648 Expires: 31-dec-2016 (134 days)
```

```
library(asremlPlus)
library(lattice)
source("~/Rstudio/Rsrc/pin.R")
source("~/Rstudio/Rsrc/AIC_table_function.R")
```

```
## Loading required package: svGUI
```

```
data <- read.table("~/Rstudio/Yan/2012-2015/18082016/Data2012_2015.csv" , header=T , sep="," , na.strings="NA")
head(data)
```

```
##   plante          pop     genotype NV     Elisa QPCR X Y neigh1 neigh2
## 1     p1         <NA>       Soldur NA        NA   NA 1 1  side1  side2
## 2   p144 Dic2 x Silur TT06DC 44.28  3 0.7173187   NA 2 1  side1  side2
## 3   p145         <NA>        Silur NA        NA   NA 3 1  side1  side2
## 4   p284 Dic2 x Silur TT06DC 44.39  0 1.0147956   NA 4 1  side1  side2
## 5   p285 Dic2 x Silur TT06DC 38.27  3 1.0133161   NA 5 1  side1  side2
## 6   p429 Dic2 x Silur TT06DC 42.15  3 0.2261215   NA 6 1  side1  side2
##   neigh3       neigh4        neigh5        neigh6        neigh7
## 1  side3 TT06DC 44.28         lloyd  TT06DC 39.35         side7
## 2  side3        Silur   TT06DC 40.4         lloyd  TT06DC 39.35
## 3  side3 TT06DC 44.39 BX07.2.1f2.71   TT06DC 40.4         lloyd
## 4  side3 TT06DC 38.27  TT06DC 38.12 BX07.2.1f2.71   TT06DC 40.4
## 5  side3 TT06DC 42.15 BX07.2.1f2.53  TT06DC 38.12 BX07.2.1f2.71
## 6  side3 BX07.3.2f1.7 BX07.2.1f2.91 BX07.2.1f2.53  TT06DC 38.12
##         neigh8 X_Y Year  cross
## 1        side8 1_1 2012 Soldur
## 2       Soldur 2_1 2012  cross
## 3 TT06DC 44.28 3_1 2012  Silur
## 4        Silur 4_1 2012  cross
## 5 TT06DC 44.39 5_1 2012  cross
## 6 TT06DC 38.27 6_1 2012  cross
```

# 2 Format variables

```
## 1529 rows
nrow(data)
```

```
## [1] 1529
```

```
## 423 rows with missing data
nrow(data[is.na(data$NV)|is.na(data$Elisa)|is.na(data$genotype),])
```

```
## [1] 423
```

```
## 209 missing genotypes
sum(is.na(data$genotype))
```

```
## [1] 209
```

```
## 380 missing visual scores
sum(is.na(data$NV))
```

```
## [1] 397
```

```
## 416 missing Elisa
sum(is.na(data$Elisa))
```

```
## [1] 416
```

```
## 361 missing Elisa
sum(is.na(data$QPCR[data$Year=="2015"]))
```

```
## [1] 361
```

```
data <- data[order(data$Year,data$Y,data$X),]

## Convert position and year as factors
data$X <- as.factor(data$X)
data$Y <- as.factor(data$Y)
data$Year <- as.factor(data$Year)
data$Obs <- as.factor(1:nrow(data))
data$genotype_year <- paste(data$genotype,data$Year,sep="_")
data$pop2015 <- data$pop
data$pop2015 <- ifelse(data$Year=="2012",NA,as.character(data$pop2015))
data$pop2015 <- as.factor(data$pop2015)
data$pop2015mean <- as.factor(ifelse(data$pop2015=="pescadou","pescadou","DLDS"))

data$cross2015 <- data$cross
data$cross2015 <- ifelse(data$Year=="2012",NA,as.character(data$cross2015))
data$cross2015 <- as.factor(data$cross2015)
data$popmean <- as.factor(ifelse(data$pop=="pescadou","pescadou","DLDS"))
levels(data$popmean)
```

```
## [1] "DLDS"     "pescadou"
```

```
## Factors to test for indirect genetic effects
levY <- sort(unique(c(levels(data$neigh2),levels(data$neigh6))))
data$neigh2<-factor(data$neigh2,levels=levY)
data$neigh6<-factor(data$neigh6,levels=levY)
data$Year2012 <- ifelse(data$Year=="2012",1,0)
data$Year2015 <- ifelse(data$Year=="2015",1,0)
## Grid arrangement on the field plot
Neighbour <- data.frame(matrix(c("neigh1","neigh2","neigh3","neigh8","focal_genotype","neigh4","neigh7","neigh6","neigh5"),byrow=F,ncol=3))
colnames(Neighbour) <- c("Y1","Y2","Y3")
rownames(Neighbour) <- c("X1","X2","X3")
Neighbour
```

```
##        Y1             Y2     Y3
## X1 neigh1         neigh8 neigh7
## X2 neigh2 focal_genotype neigh6
## X3 neigh3         neigh4 neigh5
```

```
## Dataset for model AR1 on X
data2 <- data[order(data$Year,data$X,data$Y),]

head(data)
```

```
##   plante          pop     genotype NV     Elisa QPCR X Y neigh1 neigh2
## 1     p1         <NA>       Soldur NA        NA   NA 1 1  side1  side2
## 2   p144 Dic2 x Silur TT06DC 44.28  3 0.7173187   NA 2 1  side1  side2
## 3   p145         <NA>        Silur NA        NA   NA 3 1  side1  side2
## 4   p284 Dic2 x Silur TT06DC 44.39  0 1.0147956   NA 4 1  side1  side2
## 5   p285 Dic2 x Silur TT06DC 38.27  3 1.0133161   NA 5 1  side1  side2
## 6   p429 Dic2 x Silur TT06DC 42.15  3 0.2261215   NA 6 1  side1  side2
##   neigh3       neigh4        neigh5        neigh6        neigh7
## 1  side3 TT06DC 44.28         lloyd  TT06DC 39.35         side7
## 2  side3        Silur   TT06DC 40.4         lloyd  TT06DC 39.35
## 3  side3 TT06DC 44.39 BX07.2.1f2.71   TT06DC 40.4         lloyd
## 4  side3 TT06DC 38.27  TT06DC 38.12 BX07.2.1f2.71   TT06DC 40.4
## 5  side3 TT06DC 42.15 BX07.2.1f2.53  TT06DC 38.12 BX07.2.1f2.71
## 6  side3 BX07.3.2f1.7 BX07.2.1f2.91 BX07.2.1f2.53  TT06DC 38.12
##         neigh8 X_Y Year  cross Obs     genotype_year pop2015 pop2015mean
## 1        side8 1_1 2012 Soldur   1       Soldur_2012    <NA>        <NA>
## 2       Soldur 2_1 2012  cross   2 TT06DC 44.28_2012    <NA>        <NA>
## 3 TT06DC 44.28 3_1 2012  Silur   3        Silur_2012    <NA>        <NA>
## 4        Silur 4_1 2012  cross   4 TT06DC 44.39_2012    <NA>        <NA>
## 5 TT06DC 44.39 5_1 2012  cross   5 TT06DC 38.27_2012    <NA>        <NA>
## 6 TT06DC 38.27 6_1 2012  cross   6 TT06DC 42.15_2012    <NA>        <NA>
##   cross2015 popmean Year2012 Year2015
## 1      <NA>    <NA>        1        0
## 2      <NA>    DLDS        1        0
## 3      <NA>    <NA>        1        0
## 4      <NA>    DLDS        1        0
## 5      <NA>    DLDS        1        0
## 6      <NA>    DLDS        1        0
```

```
tail(data,n=7)
```

```
##         plante          pop        genotype NV Elisa     QPCR  X  Y
## 1523 2399-2400 Dic2 x Lloyd BX07.2.1f1.34.2  2  0.72 7.240005 13 41
## 1524 2397-2398 Dic2 x Silur    TT06DC 42.53  2  0.05 4.290934 14 41
## 1525 2395-2396     pescadou        pescadou  4  1.00       NA 15 41
## 1526    espace         <NA>            <NA> NA    NA       NA 16 41
## 1527     NA-NA         <NA>            <NA> NA    NA       NA 17 41
## 1528     NA-NA         <NA>            <NA> NA    NA       NA 18 41
## 1529     NA-NA         <NA>            <NA> NA    NA       NA 19 41
##               neigh1          neigh2 neigh3 neigh4 neigh5       neigh6
## 1523   BX07.2.1f2.99        pescadou  side3  side4  side5 TT06DC 42.53
## 1524    TT06DC 39.35 BX07.2.1f1.34.2  side3  side4  side5     pescadou
## 1525 BX07.2.1f1.50.2    TT06DC 42.53  side3  side4  side5         <NA>
## 1526   BX07.2.1f2.34        pescadou  side3  side4  side5         <NA>
## 1527            <NA>            <NA>  side3  side4  side5         <NA>
## 1528            <NA>            <NA>  side3  side4  side5         <NA>
## 1529            <NA>            <NA>  side3  side4  side5        side6
##               neigh7          neigh8   X_Y Year    cross  Obs
## 1523 BX07.2.1f1.50.2    TT06DC 39.35 13_41 2015    cross 1523
## 1524   BX07.2.1f2.34 BX07.2.1f1.50.2 14_41 2015    cross 1524
## 1525            <NA>   BX07.2.1f2.34 15_41 2015 pescadou 1525
## 1526            <NA>            <NA> 16_41 2015     <NA> 1526
## 1527            <NA>            <NA> 17_41 2015     <NA> 1527
## 1528            <NA>            <NA> 18_41 2015     <NA> 1528
## 1529           side7            <NA> 19_41 2015     <NA> 1529
##             genotype_year      pop2015 pop2015mean cross2015  popmean
## 1523 BX07.2.1f1.34.2_2015 Dic2 x Lloyd        DLDS     cross     DLDS
## 1524    TT06DC 42.53_2015 Dic2 x Silur        DLDS     cross     DLDS
## 1525        pescadou_2015     pescadou    pescadou  pescadou pescadou
## 1526              NA_2015         <NA>        <NA>      <NA>     <NA>
## 1527              NA_2015         <NA>        <NA>      <NA>     <NA>
## 1528              NA_2015         <NA>        <NA>      <NA>     <NA>
## 1529              NA_2015         <NA>        <NA>      <NA>     <NA>
##      Year2012 Year2015
## 1523        0        1
## 1524        0        1
## 1525        0        1
## 1526        0        1
## 1527        0        1
## 1528        0        1
## 1529        0        1
```

# 3 Phenotypic data

```
histogram(~NV|pop*Year,data=data[data$pop=="Dic2 x Lloyd"|data$pop=="Dic2 x Silur",])
```

```
histogram(~Elisa|pop*Year,data=data[data$pop=="Dic2 x Lloyd"|data$pop=="Dic2 x Silur",])
```

```
histogram(~QPCR|pop,data=data[data$pop=="Dic2 x Lloyd"|data$pop=="Dic2 x Silur",])
```

# 4 Severity Score (NV)

## 4.1 Model selection

```
## Spatially uncorrelated and correlated (AR1) environmental variance on row (X) and column (Y) , genotype and no linear X effect (model A for direct genetic effects and model 3 for residuals)
## By default, only 13 iterations, so increase this number with maxiter
m0 <- asreml(NV ~  popmean+Year+popmean:Year+pol(X,-1)+pol(Y,-1),rcov=~at(Year):ar1(Y):ar1(X),random=~corgh(Year):at(pop,"Dic2 x Lloyd"):genotype+corgh(Year):at(pop,"Dic2 x Silur"):genotype+at(Year):X_Y,na.method.X="include",data=data,maxiter=40,trace=F)
```

```
## LogLikelihood not converged
```

```
m0 <-update(m0)

hist(m0$coefficients$random[grepl("_pop_Dic2 x Lloyd",names(m0$coefficients$random))&m0$coefficient$random!=0],main="Dic2 x Lloyd",xlab=NA)
```

```
hist(m0$coefficients$random[grepl("_pop_Dic2 x Silur",names(m0$coefficients$random))&m0$coefficient$random!=0],main="Dic2 x Silur",xlab=NA)
```

```
m0$coefficients$fixed
```

```
##     popmean_DLDS:Year_2012     popmean_DLDS:Year_2015 
##                  0.0000000                  0.0000000 
## popmean_pescadou:Year_2012 popmean_pescadou:Year_2015 
##                  0.0000000                  0.4342085 
##          pol(Y, -1)_order1          pol(X, -1)_order1 
##                 -0.1528678                  0.1728209 
##                  Year_2012                  Year_2015 
##                  0.0000000                 -0.1806650 
##               popmean_DLDS           popmean_pescadou 
##                  0.0000000                  1.2788824 
##                (Intercept) 
##                  2.3689796
```

```
summary(m0)$varcomp
```

```
##                                                                  gamma
## Year:at(pop, Dic2 x Lloyd):genotype!Year.2015:!Year.2012.cor 0.7673173
## Year:at(pop, Dic2 x Lloyd):genotype!Year.2012                0.8277440
## Year:at(pop, Dic2 x Lloyd):genotype!Year.2015                1.2129981
## Year:at(pop, Dic2 x Silur):genotype!Year.2015:!Year.2012.cor 0.6403170
## Year:at(pop, Dic2 x Silur):genotype!Year.2012                0.6638288
## Year:at(pop, Dic2 x Silur):genotype!Year.2015                1.1990636
## at(Year, 2012):X_Y!X_Y.var                                   0.3260470
## at(Year, 2015):X_Y!X_Y.var                                   0.6320306
## Year_2012!variance                                           0.2640736
## Year_2012!Y.cor                                              0.9930346
## Year_2012!X.cor                                              0.4908568
## Year_2015!variance                                           0.1877372
## Year_2015!Y.cor                                              0.9607315
## Year_2015!X.cor                                              0.8372698
##                                                              component
## Year:at(pop, Dic2 x Lloyd):genotype!Year.2015:!Year.2012.cor 0.7673173
## Year:at(pop, Dic2 x Lloyd):genotype!Year.2012                0.8277440
## Year:at(pop, Dic2 x Lloyd):genotype!Year.2015                1.2129981
## Year:at(pop, Dic2 x Silur):genotype!Year.2015:!Year.2012.cor 0.6403170
## Year:at(pop, Dic2 x Silur):genotype!Year.2012                0.6638288
## Year:at(pop, Dic2 x Silur):genotype!Year.2015                1.1990636
## at(Year, 2012):X_Y!X_Y.var                                   0.3260470
## at(Year, 2015):X_Y!X_Y.var                                   0.6320306
## Year_2012!variance                                           0.2640736
## Year_2012!Y.cor                                              0.9930346
## Year_2012!X.cor                                              0.4908568
## Year_2015!variance                                           0.1877372
## Year_2015!Y.cor                                              0.9607315
## Year_2015!X.cor                                              0.8372698
##                                                                std.error
## Year:at(pop, Dic2 x Lloyd):genotype!Year.2015:!Year.2012.cor 0.072076484
## Year:at(pop, Dic2 x Lloyd):genotype!Year.2012                0.127474831
## Year:at(pop, Dic2 x Lloyd):genotype!Year.2015                0.193134079
## Year:at(pop, Dic2 x Silur):genotype!Year.2015:!Year.2012.cor 0.082728995
## Year:at(pop, Dic2 x Silur):genotype!Year.2012                0.104837669
## Year:at(pop, Dic2 x Silur):genotype!Year.2015                0.198184648
## at(Year, 2012):X_Y!X_Y.var                                   0.034287304
## at(Year, 2015):X_Y!X_Y.var                                   0.069731011
## Year_2012!variance                                           0.139384365
## Year_2012!Y.cor                                              0.005049568
## Year_2012!X.cor                                              0.253252004
## Year_2015!variance                                           0.165726929
## Year_2015!Y.cor                                              0.052385581
## Year_2015!X.cor                                              0.166254394
##                                                                 z.ratio
## Year:at(pop, Dic2 x Lloyd):genotype!Year.2015:!Year.2012.cor  10.645876
## Year:at(pop, Dic2 x Lloyd):genotype!Year.2012                  6.493392
## Year:at(pop, Dic2 x Lloyd):genotype!Year.2015                  6.280601
## Year:at(pop, Dic2 x Silur):genotype!Year.2015:!Year.2012.cor   7.739934
## Year:at(pop, Dic2 x Silur):genotype!Year.2012                  6.331969
## Year:at(pop, Dic2 x Silur):genotype!Year.2015                  6.050234
## at(Year, 2012):X_Y!X_Y.var                                     9.509263
## at(Year, 2015):X_Y!X_Y.var                                     9.063838
## Year_2012!variance                                             1.894571
## Year_2012!Y.cor                                              196.657332
## Year_2012!X.cor                                                1.938215
## Year_2015!variance                                             1.132811
## Year_2015!Y.cor                                               18.339618
## Year_2015!X.cor                                                5.036076
##                                                                 constraint
## Year:at(pop, Dic2 x Lloyd):genotype!Year.2015:!Year.2012.cor Unconstrained
## Year:at(pop, Dic2 x Lloyd):genotype!Year.2012                     Positive
## Year:at(pop, Dic2 x Lloyd):genotype!Year.2015                     Positive
## Year:at(pop, Dic2 x Silur):genotype!Year.2015:!Year.2012.cor Unconstrained
## Year:at(pop, Dic2 x Silur):genotype!Year.2012                     Positive
## Year:at(pop, Dic2 x Silur):genotype!Year.2015                     Positive
## at(Year, 2012):X_Y!X_Y.var                                        Positive
## at(Year, 2015):X_Y!X_Y.var                                        Positive
## Year_2012!variance                                                Positive
## Year_2012!Y.cor                                              Unconstrained
## Year_2012!X.cor                                              Unconstrained
## Year_2015!variance                                                Positive
## Year_2015!Y.cor                                              Unconstrained
## Year_2015!X.cor                                              Unconstrained
```

```
## No genetic correlation between years (model B for direct genetic effects)
##- corgh(Year)->diag(Year)
m1 <- asreml(NV ~  popmean+Year+popmean:Year+pol(X,-1)+pol(Y,-1),rcov=~at(Year):ar1(Y):ar1(X),random=~diag(Year):at(pop,"Dic2 x Lloyd"):genotype+diag(Year):at(pop,"Dic2 x Silur"):genotype+at(Year):X_Y,na.method.X="include",data=data,maxiter=50,trace=F)
```

```
## LogLikelihood not converged
```

```
m1 <-update(m1)

## No difference in genetic effects between years (model C for direct genetic effects)
##- corgh(Year):
m2 <- asreml(NV ~  popmean+Year+popmean:Year+pol(X,-1)+pol(Y,-1),rcov=~at(Year):ar1(Y):ar1(X),random=~at(pop,"Dic2 x Lloyd"):genotype+at(pop,"Dic2 x Silur"):genotype+at(Year):X_Y,na.method.X="include",data=data,maxiter=40,trace=F)
```

```
## LogLikelihood not converged
```

```
m2 <-update(m2)

summary(m2)$varcomp
```

```
##                                                 gamma component
## at(pop, Dic2 x Lloyd):genotype!genotype.var 0.7923168 0.7923168
## at(pop, Dic2 x Silur):genotype!genotype.var 0.6430747 0.6430747
## at(Year, 2012):X_Y!X_Y.var                  0.3618297 0.3618297
## at(Year, 2015):X_Y!X_Y.var                  1.0160778 1.0160778
## Year_2012!variance                          0.2519044 0.2519044
## Year_2012!Y.cor                             0.9933488 0.9933488
## Year_2012!X.cor                             0.4726873 0.4726873
## Year_2015!variance                          0.1695163 0.1695163
## Year_2015!Y.cor                             0.9708398 0.9708398
## Year_2015!X.cor                             0.9394825 0.9394825
##                                               std.error    z.ratio
## at(pop, Dic2 x Lloyd):genotype!genotype.var 0.111162863   7.127531
## at(pop, Dic2 x Silur):genotype!genotype.var 0.095057817   6.765090
## at(Year, 2012):X_Y!X_Y.var                  0.033936401  10.661993
## at(Year, 2015):X_Y!X_Y.var                  0.075852936  13.395366
## Year_2012!variance                          0.131785957   1.911466
## Year_2012!Y.cor                             0.004948237 200.748028
## Year_2012!X.cor                             0.266274605   1.775187
## Year_2015!variance                          0.115207610   1.471399
## Year_2015!Y.cor                                      NA         NA
## Year_2015!X.cor                                      NA         NA
##                                                constraint
## at(pop, Dic2 x Lloyd):genotype!genotype.var      Positive
## at(pop, Dic2 x Silur):genotype!genotype.var      Positive
## at(Year, 2012):X_Y!X_Y.var                       Positive
## at(Year, 2015):X_Y!X_Y.var                       Positive
## Year_2012!variance                               Positive
## Year_2012!Y.cor                             Unconstrained
## Year_2012!X.cor                             Unconstrained
## Year_2015!variance                               Positive
## Year_2015!Y.cor                                  Boundary
## Year_2015!X.cor                                  Boundary
```

```
## Same spatially uncorrelated environmental variances, σ_uncor^2, in 2012 and 2015
## - at(Year):X_Y + Obs
m3 <- asreml(NV ~  popmean+Year+popmean:Year+pol(X,-1)+pol(Y,-1),rcov=~at(Year):ar1(Y):ar1(X),random=~corgh(Year):at(pop,"Dic2 x Lloyd"):genotype+corgh(Year):at(pop,"Dic2 x Silur"):genotype+Obs,na.method.X="include",data=data,maxiter=40,trace=F)
summary(m3)$varcomp
```

```
##                                                                   gamma
## Year:at(pop, Dic2 x Lloyd):genotype!Year.2015:!Year.2012.cor 0.77833439
## Year:at(pop, Dic2 x Lloyd):genotype!Year.2012                0.82430352
## Year:at(pop, Dic2 x Lloyd):genotype!Year.2015                1.20559252
## Year:at(pop, Dic2 x Silur):genotype!Year.2015:!Year.2012.cor 0.65930777
## Year:at(pop, Dic2 x Silur):genotype!Year.2012                0.66341323
## Year:at(pop, Dic2 x Silur):genotype!Year.2015                1.14329622
## Obs!Obs.var                                                  0.32845727
## Year_2012!variance                                           0.26851935
## Year_2012!Y.cor                                              0.99324382
## Year_2012!X.cor                                              0.50022702
## Year_2015!variance                                           0.41872070
## Year_2015!Y.cor                                              0.32090200
## Year_2015!X.cor                                              0.09898766
##                                                               component
## Year:at(pop, Dic2 x Lloyd):genotype!Year.2015:!Year.2012.cor 0.77833439
## Year:at(pop, Dic2 x Lloyd):genotype!Year.2012                0.82430352
## Year:at(pop, Dic2 x Lloyd):genotype!Year.2015                1.20559252
## Year:at(pop, Dic2 x Silur):genotype!Year.2015:!Year.2012.cor 0.65930777
## Year:at(pop, Dic2 x Silur):genotype!Year.2012                0.66341323
## Year:at(pop, Dic2 x Silur):genotype!Year.2015                1.14329622
## Obs!Obs.var                                                  0.32845727
## Year_2012!variance                                           0.26851935
## Year_2012!Y.cor                                              0.99324382
## Year_2012!X.cor                                              0.50022702
## Year_2015!variance                                           0.41872070
## Year_2015!Y.cor                                              0.32090200
## Year_2015!X.cor                                              0.09898766
##                                                                std.error
## Year:at(pop, Dic2 x Lloyd):genotype!Year.2015:!Year.2012.cor 0.072788270
## Year:at(pop, Dic2 x Lloyd):genotype!Year.2012                0.127341224
## Year:at(pop, Dic2 x Lloyd):genotype!Year.2015                0.196500141
## Year:at(pop, Dic2 x Silur):genotype!Year.2015:!Year.2012.cor 0.083439906
## Year:at(pop, Dic2 x Silur):genotype!Year.2012                0.104965746
## Year:at(pop, Dic2 x Silur):genotype!Year.2015                0.194882338
## Obs!Obs.var                                                  0.034473818
## Year_2012!variance                                           0.136050044
## Year_2012!Y.cor                                              0.004846643
## Year_2012!X.cor                                              0.240323655
## Year_2015!variance                                           0.079932699
## Year_2015!Y.cor                                              0.117571882
## Year_2015!X.cor                                              0.125872965
##                                                                  z.ratio
## Year:at(pop, Dic2 x Lloyd):genotype!Year.2015:!Year.2012.cor  10.6931293
## Year:at(pop, Dic2 x Lloyd):genotype!Year.2012                  6.4731867
## Year:at(pop, Dic2 x Lloyd):genotype!Year.2015                  6.1353265
## Year:at(pop, Dic2 x Silur):genotype!Year.2015:!Year.2012.cor   7.9015882
## Year:at(pop, Dic2 x Silur):genotype!Year.2012                  6.3202831
## Year:at(pop, Dic2 x Silur):genotype!Year.2015                  5.8665974
## Obs!Obs.var                                                    9.5277312
## Year_2012!variance                                             1.9736808
## Year_2012!Y.cor                                              204.9343863
## Year_2012!X.cor                                                2.0814723
## Year_2015!variance                                             5.2384157
## Year_2015!Y.cor                                                2.7294111
## Year_2015!X.cor                                                0.7864092
##                                                                 constraint
## Year:at(pop, Dic2 x Lloyd):genotype!Year.2015:!Year.2012.cor Unconstrained
## Year:at(pop, Dic2 x Lloyd):genotype!Year.2012                     Positive
## Year:at(pop, Dic2 x Lloyd):genotype!Year.2015                     Positive
## Year:at(pop, Dic2 x Silur):genotype!Year.2015:!Year.2012.cor Unconstrained
## Year:at(pop, Dic2 x Silur):genotype!Year.2012                     Positive
## Year:at(pop, Dic2 x Silur):genotype!Year.2015                     Positive
## Obs!Obs.var                                                       Positive
## Year_2012!variance                                                Positive
## Year_2012!Y.cor                                              Unconstrained
## Year_2012!X.cor                                              Unconstrained
## Year_2015!variance                                                Positive
## Year_2015!Y.cor                                              Unconstrained
## Year_2015!X.cor                                              Unconstrained
```

```
## AR1 on X only
m4 <- asreml(NV ~  popmean+Year+popmean:Year+pol(X,-1)+pol(Y,-1),rcov=~at(Year):id(Y):ar1(X),random=~corgh(Year):at(pop,"Dic2 x Lloyd"):genotype+corgh(Year):at(pop,"Dic2 x Silur"):genotype+at(Year):X_Y,na.method.X="include",data=data,maxiter=40,trace=F)
summary(m4)$varcomp
```

```
##                                                                     gamma
## Year:at(pop, Dic2 x Lloyd):genotype!Year.2015:!Year.2012.cor 8.712477e-01
## Year:at(pop, Dic2 x Lloyd):genotype!Year.2012                6.086689e-01
## Year:at(pop, Dic2 x Lloyd):genotype!Year.2015                1.180099e+00
## Year:at(pop, Dic2 x Silur):genotype!Year.2015:!Year.2012.cor 6.969421e-01
## Year:at(pop, Dic2 x Silur):genotype!Year.2012                6.036458e-01
## Year:at(pop, Dic2 x Silur):genotype!Year.2015                1.136004e+00
## at(Year, 2012):X_Y!X_Y.var                                   1.396584e-06
## at(Year, 2015):X_Y!X_Y.var                                   7.000926e-01
## Year_2012!variance                                           5.720022e-01
## Year_2012!X.cor                                              1.732769e-01
## Year_2015!variance                                           7.065313e-02
## Year_2015!X.cor                                              8.920204e-01
##                                                                 component
## Year:at(pop, Dic2 x Lloyd):genotype!Year.2015:!Year.2012.cor 8.712477e-01
## Year:at(pop, Dic2 x Lloyd):genotype!Year.2012                6.086689e-01
## Year:at(pop, Dic2 x Lloyd):genotype!Year.2015                1.180099e+00
## Year:at(pop, Dic2 x Silur):genotype!Year.2015:!Year.2012.cor 6.969421e-01
## Year:at(pop, Dic2 x Silur):genotype!Year.2012                6.036458e-01
## Year:at(pop, Dic2 x Silur):genotype!Year.2015                1.136004e+00
## at(Year, 2012):X_Y!X_Y.var                                   1.396584e-06
## at(Year, 2015):X_Y!X_Y.var                                   7.000926e-01
## Year_2012!variance                                           5.720022e-01
## Year_2012!X.cor                                              1.732769e-01
## Year_2015!variance                                           7.065313e-02
## Year_2015!X.cor                                              8.920204e-01
##                                                               std.error
## Year:at(pop, Dic2 x Lloyd):genotype!Year.2015:!Year.2012.cor 0.09434143
## Year:at(pop, Dic2 x Lloyd):genotype!Year.2012                0.13187687
## Year:at(pop, Dic2 x Lloyd):genotype!Year.2015                0.19675335
## Year:at(pop, Dic2 x Silur):genotype!Year.2015:!Year.2012.cor 0.09446132
## Year:at(pop, Dic2 x Silur):genotype!Year.2012                0.11688412
## Year:at(pop, Dic2 x Silur):genotype!Year.2015                0.19841799
## at(Year, 2012):X_Y!X_Y.var                                           NA
## at(Year, 2015):X_Y!X_Y.var                                   0.09001491
## Year_2012!variance                                           0.05364189
## Year_2012!X.cor                                              0.07191618
## Year_2015!variance                                           0.06213481
## Year_2015!X.cor                                              0.19208714
##                                                                z.ratio
## Year:at(pop, Dic2 x Lloyd):genotype!Year.2015:!Year.2012.cor  9.235048
## Year:at(pop, Dic2 x Lloyd):genotype!Year.2012                 4.615433
## Year:at(pop, Dic2 x Lloyd):genotype!Year.2015                 5.997859
## Year:at(pop, Dic2 x Silur):genotype!Year.2015:!Year.2012.cor  7.378069
## Year:at(pop, Dic2 x Silur):genotype!Year.2012                 5.164481
## Year:at(pop, Dic2 x Silur):genotype!Year.2015                 5.725307
## at(Year, 2012):X_Y!X_Y.var                                          NA
## at(Year, 2015):X_Y!X_Y.var                                    7.777518
## Year_2012!variance                                           10.663349
## Year_2012!X.cor                                               2.409429
## Year_2015!variance                                            1.137094
## Year_2015!X.cor                                               4.643832
##                                                                 constraint
## Year:at(pop, Dic2 x Lloyd):genotype!Year.2015:!Year.2012.cor Unconstrained
## Year:at(pop, Dic2 x Lloyd):genotype!Year.2012                     Positive
## Year:at(pop, Dic2 x Lloyd):genotype!Year.2015                     Positive
## Year:at(pop, Dic2 x Silur):genotype!Year.2015:!Year.2012.cor Unconstrained
## Year:at(pop, Dic2 x Silur):genotype!Year.2012                     Positive
## Year:at(pop, Dic2 x Silur):genotype!Year.2015                     Positive
## at(Year, 2012):X_Y!X_Y.var                                        Boundary
## at(Year, 2015):X_Y!X_Y.var                                        Positive
## Year_2012!variance                                                Positive
## Year_2012!X.cor                                              Unconstrained
## Year_2015!variance                                                Positive
## Year_2015!X.cor                                              Unconstrained
```

```
## AR1 on Y only
m5 <- asreml(NV ~ popmean+Year+popmean:Year+pol(X,-1)+pol(Y,-1),rcov=~at(Year):id(X):ar1(Y),random=~corgh(Year):at(pop,"Dic2 x Lloyd"):genotype+corgh(Year):at(pop,"Dic2 x Silur"):genotype+at(Year):X_Y,na.method.X="include",data=data2,maxiter=40,trace=F)
summary(m5)$varcomp
```

```
##                                                                  gamma
## Year:at(pop, Dic2 x Lloyd):genotype!Year.2015:!Year.2012.cor 0.7656302
## Year:at(pop, Dic2 x Lloyd):genotype!Year.2012                0.8358303
## Year:at(pop, Dic2 x Lloyd):genotype!Year.2015                1.2143915
## Year:at(pop, Dic2 x Silur):genotype!Year.2015:!Year.2012.cor 0.6476197
## Year:at(pop, Dic2 x Silur):genotype!Year.2012                0.6618250
## Year:at(pop, Dic2 x Silur):genotype!Year.2015                1.1971866
## at(Year, 2012):X_Y!X_Y.var                                   0.3274891
## at(Year, 2015):X_Y!X_Y.var                                   0.6259011
## Year_2012!variance                                           0.2500466
## Year_2012!Y.cor                                              0.9941297
## Year_2015!variance                                           0.1116337
## Year_2015!Y.cor                                              0.9055349
##                                                              component
## Year:at(pop, Dic2 x Lloyd):genotype!Year.2015:!Year.2012.cor 0.7656302
## Year:at(pop, Dic2 x Lloyd):genotype!Year.2012                0.8358303
## Year:at(pop, Dic2 x Lloyd):genotype!Year.2015                1.2143915
## Year:at(pop, Dic2 x Silur):genotype!Year.2015:!Year.2012.cor 0.6476197
## Year:at(pop, Dic2 x Silur):genotype!Year.2012                0.6618250
## Year:at(pop, Dic2 x Silur):genotype!Year.2015                1.1971866
## at(Year, 2012):X_Y!X_Y.var                                   0.3274891
## at(Year, 2015):X_Y!X_Y.var                                   0.6259011
## Year_2012!variance                                           0.2500466
## Year_2012!Y.cor                                              0.9941297
## Year_2015!variance                                           0.1116337
## Year_2015!Y.cor                                              0.9055349
##                                                               std.error
## Year:at(pop, Dic2 x Lloyd):genotype!Year.2015:!Year.2012.cor 0.07278232
## Year:at(pop, Dic2 x Lloyd):genotype!Year.2012                0.12831575
## Year:at(pop, Dic2 x Lloyd):genotype!Year.2015                0.19508744
## Year:at(pop, Dic2 x Silur):genotype!Year.2015:!Year.2012.cor 0.08281810
## Year:at(pop, Dic2 x Silur):genotype!Year.2012                0.10468993
## Year:at(pop, Dic2 x Silur):genotype!Year.2015                0.19953740
## at(Year, 2012):X_Y!X_Y.var                                   0.03428314
## at(Year, 2015):X_Y!X_Y.var                                   0.07887500
## Year_2012!variance                                           0.10940186
## Year_2012!Y.cor                                              0.00432848
## Year_2015!variance                                           0.05667364
## Year_2015!Y.cor                                              0.08371478
##                                                                 z.ratio
## Year:at(pop, Dic2 x Lloyd):genotype!Year.2015:!Year.2012.cor  10.519453
## Year:at(pop, Dic2 x Lloyd):genotype!Year.2012                  6.513856
## Year:at(pop, Dic2 x Lloyd):genotype!Year.2015                  6.224858
## Year:at(pop, Dic2 x Silur):genotype!Year.2015:!Year.2012.cor   7.819785
## Year:at(pop, Dic2 x Silur):genotype!Year.2012                  6.321763
## Year:at(pop, Dic2 x Silur):genotype!Year.2015                  5.999811
## at(Year, 2012):X_Y!X_Y.var                                     9.552481
## at(Year, 2015):X_Y!X_Y.var                                     7.935354
## Year_2012!variance                                             2.285579
## Year_2012!Y.cor                                              229.671752
## Year_2015!variance                                             1.969765
## Year_2015!Y.cor                                               10.816906
##                                                                 constraint
## Year:at(pop, Dic2 x Lloyd):genotype!Year.2015:!Year.2012.cor Unconstrained
## Year:at(pop, Dic2 x Lloyd):genotype!Year.2012                     Positive
## Year:at(pop, Dic2 x Lloyd):genotype!Year.2015                     Positive
## Year:at(pop, Dic2 x Silur):genotype!Year.2015:!Year.2012.cor Unconstrained
## Year:at(pop, Dic2 x Silur):genotype!Year.2012                     Positive
## Year:at(pop, Dic2 x Silur):genotype!Year.2015                     Positive
## at(Year, 2012):X_Y!X_Y.var                                        Positive
## at(Year, 2015):X_Y!X_Y.var                                        Positive
## Year_2012!variance                                                Positive
## Year_2012!Y.cor                                              Unconstrained
## Year_2015!variance                                                Positive
## Year_2015!Y.cor                                              Unconstrained
```

```
## No spatially correlated environmental variance (model 1 for residuals)
m6 <- asreml(NV ~  popmean+Year+popmean:Year+pol(X,-1)+pol(Y,-1),rcov=~at(Year):id(Y):id(X),random=~corgh(Year):at(pop,"Dic2 x Lloyd"):genotype+corgh(Year):at(pop,"Dic2 x Silur"):genotype,na.method.X="include",data=data,maxiter=40,trace=F)

summary(m6)$varcomp
```

```
##                                                                  gamma
## Year:at(pop, Dic2 x Lloyd):genotype!Year.2015:!Year.2012.cor 0.9236535
## Year:at(pop, Dic2 x Lloyd):genotype!Year.2012                0.5985778
## Year:at(pop, Dic2 x Lloyd):genotype!Year.2015                1.1604287
## Year:at(pop, Dic2 x Silur):genotype!Year.2015:!Year.2012.cor 0.7095442
## Year:at(pop, Dic2 x Silur):genotype!Year.2012                0.5420766
## Year:at(pop, Dic2 x Silur):genotype!Year.2015                1.1343733
## Year_2012!variance                                           0.5923947
## Year_2015!variance                                           0.7776540
##                                                              component
## Year:at(pop, Dic2 x Lloyd):genotype!Year.2015:!Year.2012.cor 0.9236535
## Year:at(pop, Dic2 x Lloyd):genotype!Year.2012                0.5985778
## Year:at(pop, Dic2 x Lloyd):genotype!Year.2015                1.1604287
## Year:at(pop, Dic2 x Silur):genotype!Year.2015:!Year.2012.cor 0.7095442
## Year:at(pop, Dic2 x Silur):genotype!Year.2012                0.5420766
## Year:at(pop, Dic2 x Silur):genotype!Year.2015                1.1343733
## Year_2012!variance                                           0.5923947
## Year_2015!variance                                           0.7776540
##                                                               std.error
## Year:at(pop, Dic2 x Lloyd):genotype!Year.2015:!Year.2012.cor 0.09834647
## Year:at(pop, Dic2 x Lloyd):genotype!Year.2012                0.13572715
## Year:at(pop, Dic2 x Lloyd):genotype!Year.2015                0.19675282
## Year:at(pop, Dic2 x Silur):genotype!Year.2015:!Year.2012.cor 0.09974610
## Year:at(pop, Dic2 x Silur):genotype!Year.2012                0.11341132
## Year:at(pop, Dic2 x Silur):genotype!Year.2015                0.20112813
## Year_2012!variance                                           0.05618670
## Year_2015!variance                                           0.07550024
##                                                                z.ratio
## Year:at(pop, Dic2 x Lloyd):genotype!Year.2015:!Year.2012.cor  9.391832
## Year:at(pop, Dic2 x Lloyd):genotype!Year.2012                 4.410155
## Year:at(pop, Dic2 x Lloyd):genotype!Year.2015                 5.897901
## Year:at(pop, Dic2 x Silur):genotype!Year.2015:!Year.2012.cor  7.113503
## Year:at(pop, Dic2 x Silur):genotype!Year.2012                 4.779739
## Year:at(pop, Dic2 x Silur):genotype!Year.2015                 5.640053
## Year_2012!variance                                           10.543327
## Year_2015!variance                                           10.300021
##                                                                 constraint
## Year:at(pop, Dic2 x Lloyd):genotype!Year.2015:!Year.2012.cor Unconstrained
## Year:at(pop, Dic2 x Lloyd):genotype!Year.2012                     Positive
## Year:at(pop, Dic2 x Lloyd):genotype!Year.2015                     Positive
## Year:at(pop, Dic2 x Silur):genotype!Year.2015:!Year.2012.cor Unconstrained
## Year:at(pop, Dic2 x Silur):genotype!Year.2012                     Positive
## Year:at(pop, Dic2 x Silur):genotype!Year.2015                     Positive
## Year_2012!variance                                                Positive
## Year_2015!variance                                                Positive
```

```
## No spatially uncorrelated environmental variance (model 2 for residuals)
m7 <- asreml(NV ~ popmean+Year+popmean:Year+pol(X,-1)+pol(Y,-1),rcov=~at(Year):ar1(Y):ar1(X),random=~corgh(Year):at(pop,"Dic2 x Lloyd"):genotype+corgh(Year):at(pop,"Dic2 x Silur"):genotype,na.method.X="include",data=data,maxiter=40,trace=F)


AICcASRemlRmd(paste("m",0:6,sep=""))
```

```
##   modnames ParNum       LL      AIC     AICc DeltaAICc      EvRatio weight
## 1       m0     25 -564.085 1178.170 1179.340     0.000 1.000000e+00  0.924
## 2       m5     23 -569.077 1184.154 1185.146     5.806 1.822900e+01  0.051
## 3       m3     24 -568.734 1185.468 1186.547     7.207 3.672700e+01  0.025
## 4       m2     21 -580.171 1202.342 1203.171    23.831 1.495670e+05  0.000
## 5       m1     23 -613.137 1272.274 1273.266    93.926 2.487550e+20  0.000
## 6       m4     23 -625.301 1296.602 1297.594   118.254 4.770126e+25  0.000
## 7       m6     19 -630.794 1299.588 1300.268   120.928 1.816275e+26  0.000
##                                                     fixed
## 1 popmean + Year + popmean:Year + pol(X, -1) + pol(Y, -1)
## 2 popmean + Year + popmean:Year + pol(X, -1) + pol(Y, -1)
## 3 popmean + Year + popmean:Year + pol(X, -1) + pol(Y, -1)
## 4 popmean + Year + popmean:Year + pol(X, -1) + pol(Y, -1)
## 5 popmean + Year + popmean:Year + pol(X, -1) + pol(Y, -1)
## 6 popmean + Year + popmean:Year + pol(X, -1) + pol(Y, -1)
## 7 popmean + Year + popmean:Year + pol(X, -1) + pol(Y, -1)
##                                                                                                        Gside
## 1 corgh(Year):at(pop, "Dic2 x Lloyd"):genotype + corgh(Year):at(pop, "Dic2 x Silur"):genotype + at(Year):X_Y
## 2 corgh(Year):at(pop, "Dic2 x Lloyd"):genotype + corgh(Year):at(pop, "Dic2 x Silur"):genotype + at(Year):X_Y
## 3          corgh(Year):at(pop, "Dic2 x Lloyd"):genotype + corgh(Year):at(pop, "Dic2 x Silur"):genotype + Obs
## 4                         at(pop, "Dic2 x Lloyd"):genotype + at(pop, "Dic2 x Silur"):genotype + at(Year):X_Y
## 5   diag(Year):at(pop, "Dic2 x Lloyd"):genotype + diag(Year):at(pop, "Dic2 x Silur"):genotype + at(Year):X_Y
## 6 corgh(Year):at(pop, "Dic2 x Lloyd"):genotype + corgh(Year):at(pop, "Dic2 x Silur"):genotype + at(Year):X_Y
## 7                corgh(Year):at(pop, "Dic2 x Lloyd"):genotype + corgh(Year):at(pop, "Dic2 x Silur"):genotype
##                     Rside
## 1 ~at(Year):ar1(Y):ar1(X)
## 2  ~at(Year):id(X):ar1(Y)
## 3 ~at(Year):ar1(Y):ar1(X)
## 4 ~at(Year):ar1(Y):ar1(X)
## 5 ~at(Year):ar1(Y):ar1(X)
## 6  ~at(Year):id(Y):ar1(X)
## 7   ~at(Year):id(Y):id(X)
```

```
filename<-paste('AICcTable_ASReml-R_Analyses_', Sys.Date(),".csv",sep="")
file.rename(from=filename, to=paste('AICcTable_ASReml-R_Analyses_', Sys.Date(),"NV",".csv",sep=""))
```

```
## [1] TRUE
```

```
summary(m0)$varcomp
```

```
##                                                                  gamma
## Year:at(pop, Dic2 x Lloyd):genotype!Year.2015:!Year.2012.cor 0.7673173
## Year:at(pop, Dic2 x Lloyd):genotype!Year.2012                0.8277440
## Year:at(pop, Dic2 x Lloyd):genotype!Year.2015                1.2129981
## Year:at(pop, Dic2 x Silur):genotype!Year.2015:!Year.2012.cor 0.6403170
## Year:at(pop, Dic2 x Silur):genotype!Year.2012                0.6638288
## Year:at(pop, Dic2 x Silur):genotype!Year.2015                1.1990636
## at(Year, 2012):X_Y!X_Y.var                                   0.3260470
## at(Year, 2015):X_Y!X_Y.var                                   0.6320306
## Year_2012!variance                                           0.2640736
## Year_2012!Y.cor                                              0.9930346
## Year_2012!X.cor                                              0.4908568
## Year_2015!variance                                           0.1877372
## Year_2015!Y.cor                                              0.9607315
## Year_2015!X.cor                                              0.8372698
##                                                              component
## Year:at(pop, Dic2 x Lloyd):genotype!Year.2015:!Year.2012.cor 0.7673173
## Year:at(pop, Dic2 x Lloyd):genotype!Year.2012                0.8277440
## Year:at(pop, Dic2 x Lloyd):genotype!Year.2015                1.2129981
## Year:at(pop, Dic2 x Silur):genotype!Year.2015:!Year.2012.cor 0.6403170
## Year:at(pop, Dic2 x Silur):genotype!Year.2012                0.6638288
## Year:at(pop, Dic2 x Silur):genotype!Year.2015                1.1990636
## at(Year, 2012):X_Y!X_Y.var                                   0.3260470
## at(Year, 2015):X_Y!X_Y.var                                   0.6320306
## Year_2012!variance                                           0.2640736
## Year_2012!Y.cor                                              0.9930346
## Year_2012!X.cor                                              0.4908568
## Year_2015!variance                                           0.1877372
## Year_2015!Y.cor                                              0.9607315
## Year_2015!X.cor                                              0.8372698
##                                                                std.error
## Year:at(pop, Dic2 x Lloyd):genotype!Year.2015:!Year.2012.cor 0.072076484
## Year:at(pop, Dic2 x Lloyd):genotype!Year.2012                0.127474831
## Year:at(pop, Dic2 x Lloyd):genotype!Year.2015                0.193134079
## Year:at(pop, Dic2 x Silur):genotype!Year.2015:!Year.2012.cor 0.082728995
## Year:at(pop, Dic2 x Silur):genotype!Year.2012                0.104837669
## Year:at(pop, Dic2 x Silur):genotype!Year.2015                0.198184648
## at(Year, 2012):X_Y!X_Y.var                                   0.034287304
## at(Year, 2015):X_Y!X_Y.var                                   0.069731011
## Year_2012!variance                                           0.139384365
## Year_2012!Y.cor                                              0.005049568
## Year_2012!X.cor                                              0.253252004
## Year_2015!variance                                           0.165726929
## Year_2015!Y.cor                                              0.052385581
## Year_2015!X.cor                                              0.166254394
##                                                                 z.ratio
## Year:at(pop, Dic2 x Lloyd):genotype!Year.2015:!Year.2012.cor  10.645876
## Year:at(pop, Dic2 x Lloyd):genotype!Year.2012                  6.493392
## Year:at(pop, Dic2 x Lloyd):genotype!Year.2015                  6.280601
## Year:at(pop, Dic2 x Silur):genotype!Year.2015:!Year.2012.cor   7.739934
## Year:at(pop, Dic2 x Silur):genotype!Year.2012                  6.331969
## Year:at(pop, Dic2 x Silur):genotype!Year.2015                  6.050234
## at(Year, 2012):X_Y!X_Y.var                                     9.509263
## at(Year, 2015):X_Y!X_Y.var                                     9.063838
## Year_2012!variance                                             1.894571
## Year_2012!Y.cor                                              196.657332
## Year_2012!X.cor                                                1.938215
## Year_2015!variance                                             1.132811
## Year_2015!Y.cor                                               18.339618
## Year_2015!X.cor                                                5.036076
##                                                                 constraint
## Year:at(pop, Dic2 x Lloyd):genotype!Year.2015:!Year.2012.cor Unconstrained
## Year:at(pop, Dic2 x Lloyd):genotype!Year.2012                     Positive
## Year:at(pop, Dic2 x Lloyd):genotype!Year.2015                     Positive
## Year:at(pop, Dic2 x Silur):genotype!Year.2015:!Year.2012.cor Unconstrained
## Year:at(pop, Dic2 x Silur):genotype!Year.2012                     Positive
## Year:at(pop, Dic2 x Silur):genotype!Year.2015                     Positive
## at(Year, 2012):X_Y!X_Y.var                                        Positive
## at(Year, 2015):X_Y!X_Y.var                                        Positive
## Year_2012!variance                                                Positive
## Year_2012!Y.cor                                              Unconstrained
## Year_2012!X.cor                                              Unconstrained
## Year_2015!variance                                                Positive
## Year_2015!Y.cor                                              Unconstrained
## Year_2015!X.cor                                              Unconstrained
```

```
## Goodness of fit
plot(m0)
```

```
plot.asrVariogram(variogram(m0))
```

```
## Spatially correlated residuals
hist(m0$residuals,main="Autocorrelated residual distribution")
```

```
## Spatially uncorrelated residuals
hist(m0$coefficient$random[grepl("X_Y",names(m0$coefficient$random))&m0$coefficient$random!=0],main="Non-autocorrelated residual  distribution")
```

```
## Check spatial auto-correlation

## 2012
y <- -100:100*0.1
plot(y,summary(m0)$varcomp[10,2]^abs(y))
```

```
x <- -10:10*2
plot(x,summary(m0)$varcomp[11,2]^abs(x))
```

```
## 2015
y <- -100:100*0.1
plot(y,summary(m0)$varcomp[13,2]^abs(y))
```

```
x <- -10:10*2
plot(x,summary(m0)$varcomp[14,2]^abs(x))
```

## 4.2 Fixed effects for severity score (NV)

```
## Fixed effects
m0 <- asreml(NV ~  popmean+Year+popmean:Year+pol(X,-1)+pol(Y,-1),rcov=~at(Year):ar1(Y):ar1(X),random=~corgh(Year):at(pop,"Dic2 x Lloyd"):genotype+corgh(Year):at(pop,"Dic2 x Silur"):genotype+at(Year):X_Y,na.method.X="include",data=data,maxiter=40,trace=F)
```

```
## LogLikelihood not converged
```

```
m0$coefficients$fixed
```

```
##     popmean_DLDS:Year_2012     popmean_DLDS:Year_2015 
##                  0.0000000                  0.0000000 
## popmean_pescadou:Year_2012 popmean_pescadou:Year_2015 
##                  0.0000000                  0.4342605 
##          pol(Y, -1)_order1          pol(X, -1)_order1 
##                 -0.1528942                  0.1739964 
##                  Year_2012                  Year_2015 
##                  0.0000000                 -0.1809834 
##               popmean_DLDS           popmean_pescadou 
##                  0.0000000                  1.2788829 
##                (Intercept) 
##                  2.3696012
```

```
##Conditional Wald F-test to test factors and their interaction
wald.asreml(m0, ssType="conditional", denDF="numeric")
```

```
## $Wald
##              Df denDF    F.inc     F.con Margin           Pr
## (Intercept)   1   2.0 381.5000 329.70000        3.297367e-03
## popmean       1 641.1 439.0000 440.00000      A 8.781404e-75
## Year          1   2.3   0.1212   0.01636      A 9.085282e-01
## pol(X, -1)    1   2.4   0.6285   0.49240      B 5.441858e-01
## pol(Y, -1)    1   5.0   1.5290   1.38200      B 2.924094e-01
## popmean:Year  1 527.2  14.1700  14.17000      B 1.853035e-04
## 
## $stratumVariances
## NULL
```

```
##Predictions for the interaction
pred <- predict(m0,classify="popmean:Year",sed=list("popmean:Year"=T))
```

```
## Predict terminating with 2 errors
## Error flags: 7 9
```

```
## Warning: Abnormal termination
## LogLikelihood not converged
## Results may be erroneous
```

```
pred$predictions
```

```
## $pvals
## 
## Notes:
## 
##    popmean Year predicted.value standard.error est.status
## 1     DLDS 2012               0              0         NA
## 2     DLDS 2015               0              0         NA
## 3 pescadou 2012               0              0         NA
## 4 pescadou 2015               0              0         NA
## 
## $sed
##      [,1] [,2] [,3] [,4]
## [1,]    0    0    0    0
## [2,]    0    0    0    0
## [3,]    0    0    0    0
## [4,]    0    0    0    0
## 
## $avsed
##  min mean  max 
##    0    0    0
```

```
## Covariance between estimates
svc(m0)
```

```
##                                                                    df
## Year:at(pop, Dic2 x Lloyd):genotype!Year.2015:!Year.2012.cor   283.99
## Year:at(pop, Dic2 x Lloyd):genotype!Year.2012                  143.84
## Year:at(pop, Dic2 x Lloyd):genotype!Year.2015                  158.59
## Year:at(pop, Dic2 x Silur):genotype!Year.2015:!Year.2012.cor   368.96
## Year:at(pop, Dic2 x Silur):genotype!Year.2012                   12.94
## Year:at(pop, Dic2 x Silur):genotype!Year.2015                   85.01
## at(Year, 2012):X_Y!X_Y.var                                    4478.59
## at(Year, 2015):X_Y!X_Y.var                                       9.46
## Year_2012!variance                                           24283.01
## Year_2012!Y.cor                                              80292.78
## Year_2012!X.cor                                                 14.49
## Year_2015!variance                                             640.04
## Year_2015!Y.cor                                                932.99
## Year_2015!X.cor                                                 50.28
##                                                              variance
## Year:at(pop, Dic2 x Lloyd):genotype!Year.2015:!Year.2012.cor    58.46
## Year:at(pop, Dic2 x Lloyd):genotype!Year.2012                 -151.00
## Year:at(pop, Dic2 x Lloyd):genotype!Year.2015                  -44.29
## Year:at(pop, Dic2 x Silur):genotype!Year.2015:!Year.2012.cor  1002.85
## Year:at(pop, Dic2 x Silur):genotype!Year.2012                  -52.04
## Year:at(pop, Dic2 x Silur):genotype!Year.2015                  -34.85
## at(Year, 2012):X_Y!X_Y.var                                    2728.99
## at(Year, 2015):X_Y!X_Y.var                                      -1.14
## Year_2012!variance                                           -1676.67
## Year_2012!Y.cor                                              -3634.38
## Year_2012!X.cor                                                 67.82
## Year_2015!variance                                               3.77
## Year_2015!Y.cor                                                 -3.22
## Year_2015!X.cor                                                  0.84
##                                                              Year:at(pop, Dic2 x Lloyd):genotype!Year.2015:!Year.2012.cor
## Year:at(pop, Dic2 x Lloyd):genotype!Year.2015:!Year.2012.cor                                                        72.49
## Year:at(pop, Dic2 x Lloyd):genotype!Year.2012                                                                        0.00
## Year:at(pop, Dic2 x Lloyd):genotype!Year.2015                                                                        0.00
## Year:at(pop, Dic2 x Silur):genotype!Year.2015:!Year.2012.cor                                                         0.00
## Year:at(pop, Dic2 x Silur):genotype!Year.2012                                                                        0.00
## Year:at(pop, Dic2 x Silur):genotype!Year.2015                                                                        0.00
## at(Year, 2012):X_Y!X_Y.var                                                                                           0.00
## at(Year, 2015):X_Y!X_Y.var                                                                                           0.00
## Year_2012!variance                                                                                                   0.00
## Year_2012!Y.cor                                                                                                      0.00
## Year_2012!X.cor                                                                                                      0.00
## Year_2015!variance                                                                                                   0.00
## Year_2015!Y.cor                                                                                                      0.00
## Year_2015!X.cor                                                                                                      0.00
##                                                              Year:at(pop, Dic2 x Lloyd):genotype!Year.2012
## Year:at(pop, Dic2 x Lloyd):genotype!Year.2015:!Year.2012.cor                                         -3.32
## Year:at(pop, Dic2 x Lloyd):genotype!Year.2012                                                       151.02
## Year:at(pop, Dic2 x Lloyd):genotype!Year.2015                                                         0.00
## Year:at(pop, Dic2 x Silur):genotype!Year.2015:!Year.2012.cor                                          0.00
## Year:at(pop, Dic2 x Silur):genotype!Year.2012                                                         0.00
## Year:at(pop, Dic2 x Silur):genotype!Year.2015                                                         0.00
## at(Year, 2012):X_Y!X_Y.var                                                                            0.00
## at(Year, 2015):X_Y!X_Y.var                                                                            0.00
## Year_2012!variance                                                                                    0.00
## Year_2012!Y.cor                                                                                       0.00
## Year_2012!X.cor                                                                                       0.00
## Year_2015!variance                                                                                    0.00
## Year_2015!Y.cor                                                                                       0.00
## Year_2015!X.cor                                                                                       0.00
##                                                              Year:at(pop, Dic2 x Lloyd):genotype!Year.2015
## Year:at(pop, Dic2 x Lloyd):genotype!Year.2015:!Year.2012.cor                                         -1.71
## Year:at(pop, Dic2 x Lloyd):genotype!Year.2012                                                       -27.66
## Year:at(pop, Dic2 x Lloyd):genotype!Year.2015                                                       -26.95
## Year:at(pop, Dic2 x Silur):genotype!Year.2015:!Year.2012.cor                                          0.00
## Year:at(pop, Dic2 x Silur):genotype!Year.2012                                                         0.00
## Year:at(pop, Dic2 x Silur):genotype!Year.2015                                                         0.00
## at(Year, 2012):X_Y!X_Y.var                                                                            0.00
## at(Year, 2015):X_Y!X_Y.var                                                                            0.00
## Year_2012!variance                                                                                    0.00
## Year_2012!Y.cor                                                                                       0.00
## Year_2012!X.cor                                                                                       0.00
## Year_2015!variance                                                                                    0.00
## Year_2015!Y.cor                                                                                       0.00
## Year_2015!X.cor                                                                                       0.00
##                                                              Year:at(pop, Dic2 x Silur):genotype!Year.2015:!Year.2012.cor
## Year:at(pop, Dic2 x Lloyd):genotype!Year.2015:!Year.2012.cor                                                         0.26
## Year:at(pop, Dic2 x Lloyd):genotype!Year.2012                                                                       -0.14
## Year:at(pop, Dic2 x Lloyd):genotype!Year.2015                                                                       -0.17
## Year:at(pop, Dic2 x Silur):genotype!Year.2015:!Year.2012.cor                                                       929.48
## Year:at(pop, Dic2 x Silur):genotype!Year.2012                                                                        0.00
## Year:at(pop, Dic2 x Silur):genotype!Year.2015                                                                        0.00
## at(Year, 2012):X_Y!X_Y.var                                                                                           0.00
## at(Year, 2015):X_Y!X_Y.var                                                                                           0.00
## Year_2012!variance                                                                                                   0.00
## Year_2012!Y.cor                                                                                                      0.00
## Year_2012!X.cor                                                                                                      0.00
## Year_2015!variance                                                                                                   0.00
## Year_2015!Y.cor                                                                                                      0.00
## Year_2015!X.cor                                                                                                      0.00
##                                                              Year:at(pop, Dic2 x Silur):genotype!Year.2012
## Year:at(pop, Dic2 x Lloyd):genotype!Year.2015:!Year.2012.cor                                         -0.09
## Year:at(pop, Dic2 x Lloyd):genotype!Year.2012                                                         1.23
## Year:at(pop, Dic2 x Lloyd):genotype!Year.2015                                                        -0.02
## Year:at(pop, Dic2 x Silur):genotype!Year.2015:!Year.2012.cor                                        -97.07
## Year:at(pop, Dic2 x Silur):genotype!Year.2012                                                       205.27
## Year:at(pop, Dic2 x Silur):genotype!Year.2015                                                         0.00
## at(Year, 2012):X_Y!X_Y.var                                                                            0.00
## at(Year, 2015):X_Y!X_Y.var                                                                            0.00
## Year_2012!variance                                                                                    0.00
## Year_2012!Y.cor                                                                                       0.00
## Year_2012!X.cor                                                                                       0.00
## Year_2015!variance                                                                                    0.00
## Year_2015!Y.cor                                                                                       0.00
## Year_2015!X.cor                                                                                       0.00
##                                                              Year:at(pop, Dic2 x Silur):genotype!Year.2015
## Year:at(pop, Dic2 x Lloyd):genotype!Year.2015:!Year.2012.cor                                         -0.02
## Year:at(pop, Dic2 x Lloyd):genotype!Year.2012                                                        -0.22
## Year:at(pop, Dic2 x Lloyd):genotype!Year.2015                                                        -0.42
## Year:at(pop, Dic2 x Silur):genotype!Year.2015:!Year.2012.cor                                        -36.64
## Year:at(pop, Dic2 x Silur):genotype!Year.2012                                                       -22.58
## Year:at(pop, Dic2 x Silur):genotype!Year.2015                                                       -27.91
## at(Year, 2012):X_Y!X_Y.var                                                                            0.00
## at(Year, 2015):X_Y!X_Y.var                                                                            0.00
## Year_2012!variance                                                                                    0.00
## Year_2012!Y.cor                                                                                       0.00
## Year_2012!X.cor                                                                                       0.00
## Year_2015!variance                                                                                    0.00
## Year_2015!Y.cor                                                                                       0.00
## Year_2015!X.cor                                                                                       0.00
##                                                              at(Year, 2012):X_Y!X_Y.var
## Year:at(pop, Dic2 x Lloyd):genotype!Year.2015:!Year.2012.cor                     -39.20
## Year:at(pop, Dic2 x Lloyd):genotype!Year.2012                                    158.25
## Year:at(pop, Dic2 x Lloyd):genotype!Year.2015                                      0.19
## Year:at(pop, Dic2 x Silur):genotype!Year.2015:!Year.2012.cor                    -367.26
## Year:at(pop, Dic2 x Silur):genotype!Year.2012                                    152.90
## Year:at(pop, Dic2 x Silur):genotype!Year.2015                                      0.14
## at(Year, 2012):X_Y!X_Y.var                                                     -1738.93
## at(Year, 2015):X_Y!X_Y.var                                                         0.00
## Year_2012!variance                                                                 0.00
## Year_2012!Y.cor                                                                    0.00
## Year_2012!X.cor                                                                    0.00
## Year_2015!variance                                                                 0.00
## Year_2015!Y.cor                                                                    0.00
## Year_2015!X.cor                                                                    0.00
##                                                              at(Year, 2015):X_Y!X_Y.var
## Year:at(pop, Dic2 x Lloyd):genotype!Year.2015:!Year.2012.cor                     -19.48
## Year:at(pop, Dic2 x Lloyd):genotype!Year.2012                                    -23.21
## Year:at(pop, Dic2 x Lloyd):genotype!Year.2015                                    -22.72
## Year:at(pop, Dic2 x Silur):genotype!Year.2015:!Year.2012.cor                    -230.21
## Year:at(pop, Dic2 x Silur):genotype!Year.2012                                    -17.56
## Year:at(pop, Dic2 x Silur):genotype!Year.2015                                    -21.08
## at(Year, 2012):X_Y!X_Y.var                                                        -2.41
## at(Year, 2015):X_Y!X_Y.var                                                        -7.91
## Year_2012!variance                                                                 0.00
## Year_2012!Y.cor                                                                    0.00
## Year_2012!X.cor                                                                    0.00
## Year_2015!variance                                                                 0.00
## Year_2015!Y.cor                                                                    0.00
## Year_2015!X.cor                                                                    0.00
##                                                              Year_2012!variance
## Year:at(pop, Dic2 x Lloyd):genotype!Year.2015:!Year.2012.cor              -0.37
## Year:at(pop, Dic2 x Lloyd):genotype!Year.2012                              5.51
## Year:at(pop, Dic2 x Lloyd):genotype!Year.2015                             -0.07
## Year:at(pop, Dic2 x Silur):genotype!Year.2015:!Year.2012.cor             -19.52
## Year:at(pop, Dic2 x Silur):genotype!Year.2012                              6.05
## Year:at(pop, Dic2 x Silur):genotype!Year.2015                              0.02
## at(Year, 2012):X_Y!X_Y.var                                              -107.74
## at(Year, 2015):X_Y!X_Y.var                                                 0.01
## Year_2012!variance                                                       149.63
## Year_2012!Y.cor                                                            0.00
## Year_2012!X.cor                                                            0.00
## Year_2015!variance                                                         0.00
## Year_2015!Y.cor                                                            0.00
## Year_2015!X.cor                                                            0.00
##                                                              Year_2012!Y.cor
## Year:at(pop, Dic2 x Lloyd):genotype!Year.2015:!Year.2012.cor           26.91
## Year:at(pop, Dic2 x Lloyd):genotype!Year.2012                        -285.38
## Year:at(pop, Dic2 x Lloyd):genotype!Year.2015                           0.56
## Year:at(pop, Dic2 x Silur):genotype!Year.2015:!Year.2012.cor          684.49
## Year:at(pop, Dic2 x Silur):genotype!Year.2012                        -212.22
## Year:at(pop, Dic2 x Silur):genotype!Year.2015                          -0.70
## at(Year, 2012):X_Y!X_Y.var                                           3339.63
## at(Year, 2015):X_Y!X_Y.var                                              0.04
## Year_2012!variance                                                  -1716.19
## Year_2012!Y.cor                                                     -3662.74
## Year_2012!X.cor                                                         0.00
## Year_2015!variance                                                      0.00
## Year_2015!Y.cor                                                         0.00
## Year_2015!X.cor                                                         0.00
##                                                              Year_2012!X.cor
## Year:at(pop, Dic2 x Lloyd):genotype!Year.2015:!Year.2012.cor           -0.12
## Year:at(pop, Dic2 x Lloyd):genotype!Year.2012                           0.47
## Year:at(pop, Dic2 x Lloyd):genotype!Year.2015                           0.02
## Year:at(pop, Dic2 x Silur):genotype!Year.2015:!Year.2012.cor            7.07
## Year:at(pop, Dic2 x Silur):genotype!Year.2012                          -1.67
## Year:at(pop, Dic2 x Silur):genotype!Year.2015                           0.03
## at(Year, 2012):X_Y!X_Y.var                                             22.44
## at(Year, 2015):X_Y!X_Y.var                                              0.00
## Year_2012!variance                                                    -38.38
## Year_2012!Y.cor                                                        13.82
## Year_2012!X.cor                                                        99.82
## Year_2015!variance                                                      0.00
## Year_2015!Y.cor                                                         0.00
## Year_2015!X.cor                                                         0.00
##                                                              Year_2015!variance
## Year:at(pop, Dic2 x Lloyd):genotype!Year.2015:!Year.2012.cor              -2.29
## Year:at(pop, Dic2 x Lloyd):genotype!Year.2012                             -1.24
## Year:at(pop, Dic2 x Lloyd):genotype!Year.2015                             -1.34
## Year:at(pop, Dic2 x Silur):genotype!Year.2015:!Year.2012.cor             -13.57
## Year:at(pop, Dic2 x Silur):genotype!Year.2012                             -1.90
## Year:at(pop, Dic2 x Silur):genotype!Year.2015                             -2.35
## at(Year, 2012):X_Y!X_Y.var                                                -0.37
## at(Year, 2015):X_Y!X_Y.var                                                -1.59
## Year_2012!variance                                                         1.10
## Year_2012!Y.cor                                                           -2.98
## Year_2012!X.cor                                                            5.09
## Year_2015!variance                                                        -2.40
## Year_2015!Y.cor                                                            0.00
## Year_2015!X.cor                                                            0.00
##                                                              Year_2015!Y.cor
## Year:at(pop, Dic2 x Lloyd):genotype!Year.2015:!Year.2012.cor            5.95
## Year:at(pop, Dic2 x Lloyd):genotype!Year.2012                           1.31
## Year:at(pop, Dic2 x Lloyd):genotype!Year.2015                           2.27
## Year:at(pop, Dic2 x Silur):genotype!Year.2015:!Year.2012.cor          109.27
## Year:at(pop, Dic2 x Silur):genotype!Year.2012                           9.89
## Year:at(pop, Dic2 x Silur):genotype!Year.2015                          12.67
## at(Year, 2012):X_Y!X_Y.var                                             -2.31
## at(Year, 2015):X_Y!X_Y.var                                              3.42
## Year_2012!variance                                                      6.08
## Year_2012!Y.cor                                                        -4.38
## Year_2012!X.cor                                                        17.72
## Year_2015!variance                                                      3.52
## Year_2015!Y.cor                                                        -4.22
## Year_2015!X.cor                                                         0.00
##                                                              Year_2015!X.cor
## Year:at(pop, Dic2 x Lloyd):genotype!Year.2015:!Year.2012.cor               1
## Year:at(pop, Dic2 x Lloyd):genotype!Year.2012                              1
## Year:at(pop, Dic2 x Lloyd):genotype!Year.2015                              1
## Year:at(pop, Dic2 x Silur):genotype!Year.2015:!Year.2012.cor               1
## Year:at(pop, Dic2 x Silur):genotype!Year.2012                              1
## Year:at(pop, Dic2 x Silur):genotype!Year.2015                              1
## at(Year, 2012):X_Y!X_Y.var                                                 1
## at(Year, 2015):X_Y!X_Y.var                                                 1
## Year_2012!variance                                                         1
## Year_2012!Y.cor                                                            1
## Year_2012!X.cor                                                            1
## Year_2015!variance                                                         1
## Year_2015!Y.cor                                                            1
## Year_2015!X.cor                                                            1
```

## 4.3 Compute genetic correlation for severity score (NV)

```
m0 <- asreml(NV ~ -1+popmean:Year,rcov=~at(Year):ar1(Y):ar1(X),random=~corgh(Year):at(pop,"Dic2 x Lloyd"):genotype+corgh(Year):at(pop,"Dic2 x Silur"):genotype+at(Year):X_Y,na.method.X="include",data=data,maxiter=40,trace=F)

summary(m0)$varcomp[c(1,4),2:3]
```

```
##                                                              component
## Year:at(pop, Dic2 x Lloyd):genotype!Year.2015:!Year.2012.cor 0.7664767
## Year:at(pop, Dic2 x Silur):genotype!Year.2015:!Year.2012.cor 0.6391386
##                                                               std.error
## Year:at(pop, Dic2 x Lloyd):genotype!Year.2015:!Year.2012.cor 0.07208418
## Year:at(pop, Dic2 x Silur):genotype!Year.2015:!Year.2012.cor 0.08281162
```

## 4.4 Compute heritabilities for NV

```
## Heritability Dic2 x Lloyd 2012
summary(m0)$varcomp[2,2]/sum(summary(m0)$varcomp[c(2,7),2])
```

```
## [1] 0.7176507
```

```
Heritability <- as.numeric(pin(m0,prop~(V2)/(V2+V7)))
CI<-c(Heritability[1]-qnorm(0.975)* Heritability[2], Heritability[1]+qnorm(0.975)* Heritability[2])
names(CI) <- c("Lower95%CI","Upper95%CI")
CI
```

```
## Lower95%CI Upper95%CI 
##  0.6350124  0.8002889
```

```
## Heritability Dic2 x Lloyd 2015
summary(m0)$varcomp[3,2]/sum(summary(m0)$varcomp[c(3,8),2])
```

```
## [1] 0.6574093
```

```
Heritability <- as.numeric(pin(m0,prop~(V3)/(V3+V8)))
CI<-c(Heritability[1]-qnorm(0.975)* Heritability[2], Heritability[1]+qnorm(0.975)* Heritability[2])
names(CI) <- c("Lower95%CI","Upper95%CI")
CI
```

```
## Lower95%CI Upper95%CI 
##  0.5611796  0.7536391
```

```
## Heritability Dic2 x Silur 2012
summary(m0)$varcomp[5,2]/sum(summary(m0)$varcomp[c(5,7),2])
```

```
## [1] 0.670006
```

```
Heritability <- as.numeric(pin(m0,prop~(V5)/(V5+V7)))
CI<-c(Heritability[1]-qnorm(0.975)* Heritability[2], Heritability[1]+qnorm(0.975)* Heritability[2])
names(CI) <- c("Lower95%CI","Upper95%CI")
CI
```

```
## Lower95%CI Upper95%CI 
##  0.5793475  0.7606644
```

```
## Heritability Dic2 x Silur 2015
summary(m0)$varcomp[6,2]/sum(summary(m0)$varcomp[c(6,8),2])
```

```
## [1] 0.6550398
```

```
Heritability <- as.numeric(pin(m0,prop~(V6)/(V6+V8)))
CI<-c(Heritability[1]-qnorm(0.975)* Heritability[2], Heritability[1]+qnorm(0.975)* Heritability[2])
names(CI) <- c("Lower95%CI","Upper95%CI")
CI
```

```
## Lower95%CI Upper95%CI 
##  0.5578999  0.7521797
```

## 4.5 Proportion of total variance explained by spatial autocorrelation for NV

```
## Spatially correlated variance Dic2 x Lloyd 2012
summary(m0)$varcomp[9,2]/sum(summary(m0)$varcomp[c(2,7,9),2])
```

```
## [1] 0.1678668
```

```
## Spatial autocorrelation Dic2 x Silur 2012
summary(m0)$varcomp[9,2]/sum(summary(m0)$varcomp[c(5,7,9),2])
```

```
## [1] 0.190789
```

```
## Spatially correlated variance Dic2 x Lloyd 2015
summary(m0)$varcomp[12,2]/sum(summary(m0)$varcomp[c(3,8,12),2])
```

```
## [1] 0.1010808
```

```
## Spatial autocorrelation  Dic2 x Silur 2015
summary(m0)$varcomp[12,2]/sum(summary(m0)$varcomp[c(6,8,12),2])
```

```
## [1] 0.1017089
```

## 4.6 Extract BLUPs for NV

```
hist(m0$coefficient$random[grepl("genotype",names(m0$coefficient$random))&m0$coefficient$random!=0],main="BLUP distribution")
```

```
## 357 genotypes in total
length(unique(data$genotype[data$cross=="cross"&!is.na(data$cross)&!is.na(data$NV)]))
```

```
## [1] 357
```

```
## 10 genotypes without replication
tab <- table(data$genotype[data$cross=="cross"&!is.na(data$cross)&!is.na(data$NV)])
length(tab[tab==1])
```

```
## [1] 10
```

```
## Extract BLUPS
coefNV <- m0$coefficient$random[grepl("genotype",names(m0$coefficient$random))&m0$coefficient$random!=0]
## Extract genotype, pop, year
coefNVname <- strsplit(names(coefNV),":")
coefNVname <- lapply(coefNVname,function(x){strsplit(x,"_")})

year = do.call("rbind", lapply(coefNVname, "[[", 1))
pop = do.call("rbind", lapply(coefNVname, "[[", 2))
geno = do.call("rbind", lapply(coefNVname, "[[", 3))

coefNV <- data.frame(pop[,3],geno[,2],year[,2],coefNV)

names(coefNV) <- c("pop","geno","year","BLUP")
head(coefNV)
```

```
##                                                                                    pop
## Year_2012:at(pop, Dic2 x Lloyd)_pop_Dic2 x Lloyd:genotype_BX07.2.1f1.1.1  Dic2 x Lloyd
## Year_2012:at(pop, Dic2 x Lloyd)_pop_Dic2 x Lloyd:genotype_BX07.2.1f1.1.2  Dic2 x Lloyd
## Year_2012:at(pop, Dic2 x Lloyd)_pop_Dic2 x Lloyd:genotype_BX07.2.1f1.10.1 Dic2 x Lloyd
## Year_2012:at(pop, Dic2 x Lloyd)_pop_Dic2 x Lloyd:genotype_BX07.2.1f1.10.2 Dic2 x Lloyd
## Year_2012:at(pop, Dic2 x Lloyd)_pop_Dic2 x Lloyd:genotype_BX07.2.1f1.11.1 Dic2 x Lloyd
## Year_2012:at(pop, Dic2 x Lloyd)_pop_Dic2 x Lloyd:genotype_BX07.2.1f1.11.2 Dic2 x Lloyd
##                                                                                      geno
## Year_2012:at(pop, Dic2 x Lloyd)_pop_Dic2 x Lloyd:genotype_BX07.2.1f1.1.1   BX07.2.1f1.1.1
## Year_2012:at(pop, Dic2 x Lloyd)_pop_Dic2 x Lloyd:genotype_BX07.2.1f1.1.2   BX07.2.1f1.1.2
## Year_2012:at(pop, Dic2 x Lloyd)_pop_Dic2 x Lloyd:genotype_BX07.2.1f1.10.1 BX07.2.1f1.10.1
## Year_2012:at(pop, Dic2 x Lloyd)_pop_Dic2 x Lloyd:genotype_BX07.2.1f1.10.2 BX07.2.1f1.10.2
## Year_2012:at(pop, Dic2 x Lloyd)_pop_Dic2 x Lloyd:genotype_BX07.2.1f1.11.1 BX07.2.1f1.11.1
## Year_2012:at(pop, Dic2 x Lloyd)_pop_Dic2 x Lloyd:genotype_BX07.2.1f1.11.2 BX07.2.1f1.11.2
##                                                                           year
## Year_2012:at(pop, Dic2 x Lloyd)_pop_Dic2 x Lloyd:genotype_BX07.2.1f1.1.1  2012
## Year_2012:at(pop, Dic2 x Lloyd)_pop_Dic2 x Lloyd:genotype_BX07.2.1f1.1.2  2012
## Year_2012:at(pop, Dic2 x Lloyd)_pop_Dic2 x Lloyd:genotype_BX07.2.1f1.10.1 2012
## Year_2012:at(pop, Dic2 x Lloyd)_pop_Dic2 x Lloyd:genotype_BX07.2.1f1.10.2 2012
## Year_2012:at(pop, Dic2 x Lloyd)_pop_Dic2 x Lloyd:genotype_BX07.2.1f1.11.1 2012
## Year_2012:at(pop, Dic2 x Lloyd)_pop_Dic2 x Lloyd:genotype_BX07.2.1f1.11.2 2012
##                                                                                 BLUP
## Year_2012:at(pop, Dic2 x Lloyd)_pop_Dic2 x Lloyd:genotype_BX07.2.1f1.1.1  -0.4017219
## Year_2012:at(pop, Dic2 x Lloyd)_pop_Dic2 x Lloyd:genotype_BX07.2.1f1.1.2   1.6763282
## Year_2012:at(pop, Dic2 x Lloyd)_pop_Dic2 x Lloyd:genotype_BX07.2.1f1.10.1 -0.3965409
## Year_2012:at(pop, Dic2 x Lloyd)_pop_Dic2 x Lloyd:genotype_BX07.2.1f1.10.2  0.2253626
## Year_2012:at(pop, Dic2 x Lloyd)_pop_Dic2 x Lloyd:genotype_BX07.2.1f1.11.1 -0.2857047
## Year_2012:at(pop, Dic2 x Lloyd)_pop_Dic2 x Lloyd:genotype_BX07.2.1f1.11.2 -0.4051561
```

```
## 357 genotypes
nrow(coefNV[coefNV$year=="2012",])
```

```
## [1] 357
```

```
nrow(coefNV[coefNV$year=="2015",])
```

```
## [1] 357
```

```
coefNV$genotype_year <- paste(coefNV$geno,coefNV$year,sep="_")

coefNV$real <- coefNV$genotype_year%in%data$genotype_year
head(coefNV[coefNV$real==FALSE,])
```

```
##                                                                                    pop
## Year_2012:at(pop, Dic2 x Lloyd)_pop_Dic2 x Lloyd:genotype_BX07.2.1f1.17.1 Dic2 x Lloyd
## Year_2012:at(pop, Dic2 x Lloyd)_pop_Dic2 x Lloyd:genotype_BX07.2.1f1.46.1 Dic2 x Lloyd
## Year_2012:at(pop, Dic2 x Lloyd)_pop_Dic2 x Lloyd:genotype_BX07.2.1f2.26   Dic2 x Lloyd
## Year_2012:at(pop, Dic2 x Lloyd)_pop_Dic2 x Lloyd:genotype_BX07.2.1f2.81   Dic2 x Lloyd
## Year_2012:at(pop, Dic2 x Lloyd)_pop_Dic2 x Lloyd:genotype_BX07.2.1f2.95   Dic2 x Lloyd
## Year_2015:at(pop, Dic2 x Lloyd)_pop_Dic2 x Lloyd:genotype_BX07.2.1f1.16.1 Dic2 x Lloyd
##                                                                                      geno
## Year_2012:at(pop, Dic2 x Lloyd)_pop_Dic2 x Lloyd:genotype_BX07.2.1f1.17.1 BX07.2.1f1.17.1
## Year_2012:at(pop, Dic2 x Lloyd)_pop_Dic2 x Lloyd:genotype_BX07.2.1f1.46.1 BX07.2.1f1.46.1
## Year_2012:at(pop, Dic2 x Lloyd)_pop_Dic2 x Lloyd:genotype_BX07.2.1f2.26     BX07.2.1f2.26
## Year_2012:at(pop, Dic2 x Lloyd)_pop_Dic2 x Lloyd:genotype_BX07.2.1f2.81     BX07.2.1f2.81
## Year_2012:at(pop, Dic2 x Lloyd)_pop_Dic2 x Lloyd:genotype_BX07.2.1f2.95     BX07.2.1f2.95
## Year_2015:at(pop, Dic2 x Lloyd)_pop_Dic2 x Lloyd:genotype_BX07.2.1f1.16.1 BX07.2.1f1.16.1
##                                                                           year
## Year_2012:at(pop, Dic2 x Lloyd)_pop_Dic2 x Lloyd:genotype_BX07.2.1f1.17.1 2012
## Year_2012:at(pop, Dic2 x Lloyd)_pop_Dic2 x Lloyd:genotype_BX07.2.1f1.46.1 2012
## Year_2012:at(pop, Dic2 x Lloyd)_pop_Dic2 x Lloyd:genotype_BX07.2.1f2.26   2012
## Year_2012:at(pop, Dic2 x Lloyd)_pop_Dic2 x Lloyd:genotype_BX07.2.1f2.81   2012
## Year_2012:at(pop, Dic2 x Lloyd)_pop_Dic2 x Lloyd:genotype_BX07.2.1f2.95   2012
## Year_2015:at(pop, Dic2 x Lloyd)_pop_Dic2 x Lloyd:genotype_BX07.2.1f1.16.1 2015
##                                                                                  BLUP
## Year_2012:at(pop, Dic2 x Lloyd)_pop_Dic2 x Lloyd:genotype_BX07.2.1f1.17.1 -1.11941479
## Year_2012:at(pop, Dic2 x Lloyd)_pop_Dic2 x Lloyd:genotype_BX07.2.1f1.46.1 -0.37006183
## Year_2012:at(pop, Dic2 x Lloyd)_pop_Dic2 x Lloyd:genotype_BX07.2.1f2.26    0.41069507
## Year_2012:at(pop, Dic2 x Lloyd)_pop_Dic2 x Lloyd:genotype_BX07.2.1f2.81   -0.71891793
## Year_2012:at(pop, Dic2 x Lloyd)_pop_Dic2 x Lloyd:genotype_BX07.2.1f2.95   -0.08590703
## Year_2015:at(pop, Dic2 x Lloyd)_pop_Dic2 x Lloyd:genotype_BX07.2.1f1.16.1  0.33385106
##                                                                                  genotype_year
## Year_2012:at(pop, Dic2 x Lloyd)_pop_Dic2 x Lloyd:genotype_BX07.2.1f1.17.1 BX07.2.1f1.17.1_2012
## Year_2012:at(pop, Dic2 x Lloyd)_pop_Dic2 x Lloyd:genotype_BX07.2.1f1.46.1 BX07.2.1f1.46.1_2012
## Year_2012:at(pop, Dic2 x Lloyd)_pop_Dic2 x Lloyd:genotype_BX07.2.1f2.26     BX07.2.1f2.26_2012
## Year_2012:at(pop, Dic2 x Lloyd)_pop_Dic2 x Lloyd:genotype_BX07.2.1f2.81     BX07.2.1f2.81_2012
## Year_2012:at(pop, Dic2 x Lloyd)_pop_Dic2 x Lloyd:genotype_BX07.2.1f2.95     BX07.2.1f2.95_2012
## Year_2015:at(pop, Dic2 x Lloyd)_pop_Dic2 x Lloyd:genotype_BX07.2.1f1.16.1 BX07.2.1f1.16.1_2015
##                                                                            real
## Year_2012:at(pop, Dic2 x Lloyd)_pop_Dic2 x Lloyd:genotype_BX07.2.1f1.17.1 FALSE
## Year_2012:at(pop, Dic2 x Lloyd)_pop_Dic2 x Lloyd:genotype_BX07.2.1f1.46.1 FALSE
## Year_2012:at(pop, Dic2 x Lloyd)_pop_Dic2 x Lloyd:genotype_BX07.2.1f2.26   FALSE
## Year_2012:at(pop, Dic2 x Lloyd)_pop_Dic2 x Lloyd:genotype_BX07.2.1f2.81   FALSE
## Year_2012:at(pop, Dic2 x Lloyd)_pop_Dic2 x Lloyd:genotype_BX07.2.1f2.95   FALSE
## Year_2015:at(pop, Dic2 x Lloyd)_pop_Dic2 x Lloyd:genotype_BX07.2.1f1.16.1 FALSE
```

```
## Get rid of genotypes with infered BLUPS (i.e. that were not observed that year)
coefNV <- coefNV[coefNV$real==T,]
write.csv(coefNV,file="BLUPsNV2012_2015.csv",row.names=F,quote=F)
```

# 5 ELISA

## 5.1 Model selection

```
## Spatially uncorrelated and correlated (AR1) environmental variance on row (X) and column (Y) , genotype and no linear X effect (model A for direct genetic effects and model 3 for residuals) (not converged)
## By default, only 13 iterations, so increase this number with maxiter (not converged)
m0 <- asreml(Elisa ~ popmean+Year+popmean:Year+pol(X,-1)+pol(Y,-1),rcov=~at(Year):ar1(Y):ar1(X),random=~corgh(Year):at(pop,"Dic2 x Silur"):genotype+corgh(Year):at(pop,"Dic2 x Lloyd"):genotype+at(Year):X_Y,na.method.X="include",data=data,maxiter=100,trace=F)

hist(m0$coefficients$random[grepl("_pop_Dic2 x Lloyd",names(m0$coefficients$random))&m0$coefficient$random!=0],main="Dic2 x Lloyd",xlab=NA)
```

```
hist(m0$coefficients$random[grepl("_pop_Dic2 x Silur",names(m0$coefficients$random))&m0$coefficient$random!=0],main="Dic2 x Silur",xlab=NA)
```

```
summary(m0)$varcomp
```

```
##                                                                   gamma
## Year:at(pop, Dic2 x Silur):genotype!Year.2015:!Year.2012.cor 0.76726707
## Year:at(pop, Dic2 x Silur):genotype!Year.2012                0.08373045
## Year:at(pop, Dic2 x Silur):genotype!Year.2015                0.14488006
## Year:at(pop, Dic2 x Lloyd):genotype!Year.2015:!Year.2012.cor 0.79259441
## Year:at(pop, Dic2 x Lloyd):genotype!Year.2012                0.07145543
## Year:at(pop, Dic2 x Lloyd):genotype!Year.2015                0.13135315
## at(Year, 2012):X_Y!X_Y.var                                   0.12157232
## at(Year, 2015):X_Y!X_Y.var                                   0.03906296
## Year_2012!variance                                           0.07981287
## Year_2012!Y.cor                                              0.99337414
## Year_2012!X.cor                                              0.40213251
## Year_2015!variance                                           0.01898157
## Year_2015!Y.cor                                              0.91994742
## Year_2015!X.cor                                              0.85880638
##                                                               component
## Year:at(pop, Dic2 x Silur):genotype!Year.2015:!Year.2012.cor 0.76726707
## Year:at(pop, Dic2 x Silur):genotype!Year.2012                0.08373045
## Year:at(pop, Dic2 x Silur):genotype!Year.2015                0.14488006
## Year:at(pop, Dic2 x Lloyd):genotype!Year.2015:!Year.2012.cor 0.79259441
## Year:at(pop, Dic2 x Lloyd):genotype!Year.2012                0.07145543
## Year:at(pop, Dic2 x Lloyd):genotype!Year.2015                0.13135315
## at(Year, 2012):X_Y!X_Y.var                                   0.12157232
## at(Year, 2015):X_Y!X_Y.var                                   0.03906296
## Year_2012!variance                                           0.07981287
## Year_2012!Y.cor                                              0.99337414
## Year_2012!X.cor                                              0.40213251
## Year_2015!variance                                           0.01898157
## Year_2015!Y.cor                                              0.91994742
## Year_2015!X.cor                                              0.85880638
##                                                                std.error
## Year:at(pop, Dic2 x Silur):genotype!Year.2015:!Year.2012.cor 0.103382458
## Year:at(pop, Dic2 x Silur):genotype!Year.2012                0.021219200
## Year:at(pop, Dic2 x Silur):genotype!Year.2015                0.020464606
## Year:at(pop, Dic2 x Lloyd):genotype!Year.2015:!Year.2012.cor 0.140231764
## Year:at(pop, Dic2 x Lloyd):genotype!Year.2012                0.025018224
## Year:at(pop, Dic2 x Lloyd):genotype!Year.2015                0.017918229
## at(Year, 2012):X_Y!X_Y.var                                   0.012858728
## at(Year, 2015):X_Y!X_Y.var                                   0.004844235
## Year_2012!variance                                           0.041840428
## Year_2012!Y.cor                                              0.005010018
## Year_2012!X.cor                                              0.301631899
## Year_2015!variance                                           0.010080950
## Year_2015!Y.cor                                              0.067463505
## Year_2015!X.cor                                              0.105596953
##                                                                 z.ratio
## Year:at(pop, Dic2 x Silur):genotype!Year.2015:!Year.2012.cor   7.421637
## Year:at(pop, Dic2 x Silur):genotype!Year.2012                  3.945976
## Year:at(pop, Dic2 x Silur):genotype!Year.2015                  7.079543
## Year:at(pop, Dic2 x Lloyd):genotype!Year.2015:!Year.2012.cor   5.652032
## Year:at(pop, Dic2 x Lloyd):genotype!Year.2012                  2.856135
## Year:at(pop, Dic2 x Lloyd):genotype!Year.2015                  7.330699
## at(Year, 2012):X_Y!X_Y.var                                     9.454460
## at(Year, 2015):X_Y!X_Y.var                                     8.063803
## Year_2012!variance                                             1.907554
## Year_2012!Y.cor                                              198.277573
## Year_2012!X.cor                                                1.333190
## Year_2015!variance                                             1.882914
## Year_2015!Y.cor                                               13.636223
## Year_2015!X.cor                                                8.132871
##                                                                 constraint
## Year:at(pop, Dic2 x Silur):genotype!Year.2015:!Year.2012.cor Unconstrained
## Year:at(pop, Dic2 x Silur):genotype!Year.2012                     Positive
## Year:at(pop, Dic2 x Silur):genotype!Year.2015                     Positive
## Year:at(pop, Dic2 x Lloyd):genotype!Year.2015:!Year.2012.cor Unconstrained
## Year:at(pop, Dic2 x Lloyd):genotype!Year.2012                     Positive
## Year:at(pop, Dic2 x Lloyd):genotype!Year.2015                     Positive
## at(Year, 2012):X_Y!X_Y.var                                        Positive
## at(Year, 2015):X_Y!X_Y.var                                        Positive
## Year_2012!variance                                                Positive
## Year_2012!Y.cor                                              Unconstrained
## Year_2012!X.cor                                              Unconstrained
## Year_2015!variance                                                Positive
## Year_2015!Y.cor                                              Unconstrained
## Year_2015!X.cor                                              Unconstrained
```

```
## No genetic correlation between years (model B for direct genetic effects) (not converged)
##- corgh(Year)->diag(Year)
m1 <- asreml(Elisa ~ popmean+Year+popmean:Year+pol(X,-1)+pol(Y,-1),rcov=~at(Year):ar1(Y):ar1(X),random=~diag(Year):at(pop,"Dic2 x Lloyd"):genotype+diag(Year):at(pop,"Dic2 x Silur"):genotype+at(Year):X_Y,na.method.X="include",data=data,maxiter=50,trace=F)

## No difference in genetic effects between years (model C for direct genetic effects)
##- corgh(Year):
m2 <- asreml(Elisa ~ popmean+Year+popmean:Year+pol(X,-1)+pol(Y,-1),rcov=~at(Year):ar1(Y):ar1(X),random=~at(pop,"Dic2 x Lloyd"):genotype+at(pop,"Dic2 x Silur"):genotype+at(Year):X_Y,na.method.X="include",data=data,maxiter=40,trace=F)
summary(m2)$varcomp
```

```
##                                                  gamma  component
## at(pop, Dic2 x Lloyd):genotype!genotype.var 0.09853361 0.09853361
## at(pop, Dic2 x Silur):genotype!genotype.var 0.10735021 0.10735021
## at(Year, 2012):X_Y!X_Y.var                  0.13103087 0.13103087
## at(Year, 2015):X_Y!X_Y.var                  0.05416998 0.05416998
## Year_2012!variance                          0.08202406 0.08202406
## Year_2012!Y.cor                             0.99472771 0.99472771
## Year_2012!X.cor                             0.43149549 0.43149549
## Year_2015!variance                          0.01793122 0.01793122
## Year_2015!Y.cor                             0.89274037 0.89274037
## Year_2015!X.cor                             0.83618495 0.83618495
##                                               std.error    z.ratio
## at(pop, Dic2 x Lloyd):genotype!genotype.var 0.014337732   6.872329
## at(pop, Dic2 x Silur):genotype!genotype.var 0.015692189   6.840996
## at(Year, 2012):X_Y!X_Y.var                  0.009758943  13.426749
## at(Year, 2015):X_Y!X_Y.var                  0.006071193   8.922460
## Year_2012!variance                          0.047896206   1.712538
## Year_2012!Y.cor                             0.004423111 224.893225
## Year_2012!X.cor                             0.323433102   1.334110
## Year_2015!variance                          0.008003633   2.240385
## Year_2015!Y.cor                             0.079047594  11.293707
## Year_2015!X.cor                             0.110041935   7.598785
##                                                constraint
## at(pop, Dic2 x Lloyd):genotype!genotype.var      Positive
## at(pop, Dic2 x Silur):genotype!genotype.var      Positive
## at(Year, 2012):X_Y!X_Y.var                       Positive
## at(Year, 2015):X_Y!X_Y.var                       Positive
## Year_2012!variance                               Positive
## Year_2012!Y.cor                             Unconstrained
## Year_2012!X.cor                             Unconstrained
## Year_2015!variance                               Positive
## Year_2015!Y.cor                             Unconstrained
## Year_2015!X.cor                             Unconstrained
```

```
## Same spatially uncorrelated environmental variances, σ_uncor^2, in 2012 and 2015
## - at(Year):X_Y + Obs
m3 <- asreml(Elisa ~ popmean+Year+popmean:Year+pol(X,-1)+pol(Y,-1),rcov=~at(Year):ar1(Y):ar1(X),random=~corgh(Year):at(pop,"Dic2 x Lloyd"):genotype+corgh(Year):at(pop,"Dic2 x Silur"):genotype+Obs,na.method.X="include",data=data,maxiter=40,trace=F)
summary(m3)$varcomp
```

```
##                                                                   gamma
## Year:at(pop, Dic2 x Lloyd):genotype!Year.2015:!Year.2012.cor 0.65708941
## Year:at(pop, Dic2 x Lloyd):genotype!Year.2012                0.12550609
## Year:at(pop, Dic2 x Lloyd):genotype!Year.2015                0.10468441
## Year:at(pop, Dic2 x Silur):genotype!Year.2015:!Year.2012.cor 0.68443539
## Year:at(pop, Dic2 x Silur):genotype!Year.2012                0.11886597
## Year:at(pop, Dic2 x Silur):genotype!Year.2015                0.12068702
## Obs!Obs.var                                                  0.07376984
## Year_2012!variance                                           0.08698773
## Year_2012!Y.cor                                              0.98574413
## Year_2012!X.cor                                              0.17073246
## Year_2015!variance                                           0.01887765
## Year_2015!Y.cor                                              0.96092778
## Year_2015!X.cor                                              0.93356180
##                                                               component
## Year:at(pop, Dic2 x Lloyd):genotype!Year.2015:!Year.2012.cor 0.65708941
## Year:at(pop, Dic2 x Lloyd):genotype!Year.2012                0.12550609
## Year:at(pop, Dic2 x Lloyd):genotype!Year.2015                0.10468441
## Year:at(pop, Dic2 x Silur):genotype!Year.2015:!Year.2012.cor 0.68443539
## Year:at(pop, Dic2 x Silur):genotype!Year.2012                0.11886597
## Year:at(pop, Dic2 x Silur):genotype!Year.2015                0.12068702
## Obs!Obs.var                                                  0.07376984
## Year_2012!variance                                           0.08698773
## Year_2012!Y.cor                                              0.98574413
## Year_2012!X.cor                                              0.17073246
## Year_2015!variance                                           0.01887765
## Year_2015!Y.cor                                              0.96092778
## Year_2015!X.cor                                              0.93356180
##                                                                std.error
## Year:at(pop, Dic2 x Lloyd):genotype!Year.2015:!Year.2012.cor 0.101156209
## Year:at(pop, Dic2 x Lloyd):genotype!Year.2012                0.023109251
## Year:at(pop, Dic2 x Lloyd):genotype!Year.2015                0.018157811
## Year:at(pop, Dic2 x Silur):genotype!Year.2015:!Year.2012.cor 0.093143419
## Year:at(pop, Dic2 x Silur):genotype!Year.2012                0.020658972
## Year:at(pop, Dic2 x Silur):genotype!Year.2015                0.020654395
## Obs!Obs.var                                                  0.005947288
## Year_2012!variance                                           0.031929517
## Year_2012!Y.cor                                              0.007802159
## Year_2012!X.cor                                              0.227641226
## Year_2015!variance                                           0.021630153
## Year_2015!Y.cor                                              0.061569911
## Year_2015!X.cor                                              0.092051835
##                                                                  z.ratio
## Year:at(pop, Dic2 x Lloyd):genotype!Year.2015:!Year.2012.cor   6.4957893
## Year:at(pop, Dic2 x Lloyd):genotype!Year.2012                  5.4309890
## Year:at(pop, Dic2 x Lloyd):genotype!Year.2015                  5.7652551
## Year:at(pop, Dic2 x Silur):genotype!Year.2015:!Year.2012.cor   7.3481884
## Year:at(pop, Dic2 x Silur):genotype!Year.2012                  5.7537212
## Year:at(pop, Dic2 x Silur):genotype!Year.2015                  5.8431642
## Obs!Obs.var                                                   12.4039461
## Year_2012!variance                                             2.7243673
## Year_2012!Y.cor                                              126.3424901
## Year_2012!X.cor                                                0.7500068
## Year_2015!variance                                             0.8727469
## Year_2015!Y.cor                                               15.6071003
## Year_2015!X.cor                                               10.1416968
##                                                                 constraint
## Year:at(pop, Dic2 x Lloyd):genotype!Year.2015:!Year.2012.cor Unconstrained
## Year:at(pop, Dic2 x Lloyd):genotype!Year.2012                     Positive
## Year:at(pop, Dic2 x Lloyd):genotype!Year.2015                     Positive
## Year:at(pop, Dic2 x Silur):genotype!Year.2015:!Year.2012.cor Unconstrained
## Year:at(pop, Dic2 x Silur):genotype!Year.2012                     Positive
## Year:at(pop, Dic2 x Silur):genotype!Year.2015                     Positive
## Obs!Obs.var                                                       Positive
## Year_2012!variance                                                Positive
## Year_2012!Y.cor                                              Unconstrained
## Year_2012!X.cor                                              Unconstrained
## Year_2015!variance                                                Positive
## Year_2015!Y.cor                                              Unconstrained
## Year_2015!X.cor                                              Unconstrained
```

```
##AR1 on X only
m4 <- asreml(Elisa ~ popmean+Year+popmean:Year+pol(X,-1)+pol(Y,-1),rcov=~at(Year):id(Y):ar1(X),random=~corgh(Year):at(pop,"Dic2 x Lloyd"):genotype+corgh(Year):at(pop,"Dic2 x Silur"):genotype+at(Year):X_Y,na.method.X="include",data=data,maxiter=40,trace=F)
```

```
## LogLikelihood not converged
```

```
m4 <- update(m4)
summary(m4)$varcomp
```

```
##                                                                     gamma
## Year:at(pop, Dic2 x Lloyd):genotype!Year.2015:!Year.2012.cor 9.999848e-01
## Year:at(pop, Dic2 x Lloyd):genotype!Year.2012                4.381302e-02
## Year:at(pop, Dic2 x Lloyd):genotype!Year.2015                1.301623e-01
## Year:at(pop, Dic2 x Silur):genotype!Year.2015:!Year.2012.cor 8.634476e-01
## Year:at(pop, Dic2 x Silur):genotype!Year.2012                6.922141e-02
## Year:at(pop, Dic2 x Silur):genotype!Year.2015                1.506585e-01
## at(Year, 2012):X_Y!X_Y.var                                   1.869132e-01
## at(Year, 2015):X_Y!X_Y.var                                   3.942712e-02
## Year_2012!variance                                           5.391076e-08
## Year_2012!X.cor                                              1.551295e-01
## Year_2015!variance                                           1.181654e-02
## Year_2015!X.cor                                              7.987236e-01
##                                                                 component
## Year:at(pop, Dic2 x Lloyd):genotype!Year.2015:!Year.2012.cor 9.999848e-01
## Year:at(pop, Dic2 x Lloyd):genotype!Year.2012                4.381302e-02
## Year:at(pop, Dic2 x Lloyd):genotype!Year.2015                1.301623e-01
## Year:at(pop, Dic2 x Silur):genotype!Year.2015:!Year.2012.cor 8.634476e-01
## Year:at(pop, Dic2 x Silur):genotype!Year.2012                6.922141e-02
## Year:at(pop, Dic2 x Silur):genotype!Year.2015                1.506585e-01
## at(Year, 2012):X_Y!X_Y.var                                   1.869132e-01
## at(Year, 2015):X_Y!X_Y.var                                   3.942712e-02
## Year_2012!variance                                           5.391076e-08
## Year_2012!X.cor                                              1.551295e-01
## Year_2015!variance                                           1.181654e-02
## Year_2015!X.cor                                              7.987236e-01
##                                                                std.error
## Year:at(pop, Dic2 x Lloyd):genotype!Year.2015:!Year.2012.cor          NA
## Year:at(pop, Dic2 x Lloyd):genotype!Year.2012                0.016332973
## Year:at(pop, Dic2 x Lloyd):genotype!Year.2015                0.018206359
## Year:at(pop, Dic2 x Silur):genotype!Year.2015:!Year.2012.cor 0.133419746
## Year:at(pop, Dic2 x Silur):genotype!Year.2012                0.023200832
## Year:at(pop, Dic2 x Silur):genotype!Year.2015                0.021546315
## at(Year, 2012):X_Y!X_Y.var                                   0.013505602
## at(Year, 2015):X_Y!X_Y.var                                   0.007011088
## Year_2012!variance                                                    NA
## Year_2012!X.cor                                                       NA
## Year_2015!variance                                           0.006444086
## Year_2015!X.cor                                              0.169641957
##                                                                z.ratio
## Year:at(pop, Dic2 x Lloyd):genotype!Year.2015:!Year.2012.cor        NA
## Year:at(pop, Dic2 x Lloyd):genotype!Year.2012                 2.682489
## Year:at(pop, Dic2 x Lloyd):genotype!Year.2015                 7.149278
## Year:at(pop, Dic2 x Silur):genotype!Year.2015:!Year.2012.cor  6.471663
## Year:at(pop, Dic2 x Silur):genotype!Year.2012                 2.983574
## Year:at(pop, Dic2 x Silur):genotype!Year.2015                 6.992309
## at(Year, 2012):X_Y!X_Y.var                                   13.839682
## at(Year, 2015):X_Y!X_Y.var                                    5.623538
## Year_2012!variance                                                  NA
## Year_2012!X.cor                                                     NA
## Year_2015!variance                                            1.833703
## Year_2015!X.cor                                               4.708291
##                                                                 constraint
## Year:at(pop, Dic2 x Lloyd):genotype!Year.2015:!Year.2012.cor      Boundary
## Year:at(pop, Dic2 x Lloyd):genotype!Year.2012                     Positive
## Year:at(pop, Dic2 x Lloyd):genotype!Year.2015                     Positive
## Year:at(pop, Dic2 x Silur):genotype!Year.2015:!Year.2012.cor Unconstrained
## Year:at(pop, Dic2 x Silur):genotype!Year.2012                     Positive
## Year:at(pop, Dic2 x Silur):genotype!Year.2015                     Positive
## at(Year, 2012):X_Y!X_Y.var                                        Positive
## at(Year, 2015):X_Y!X_Y.var                                        Positive
## Year_2012!variance                                                Boundary
## Year_2012!X.cor                                                   Boundary
## Year_2015!variance                                                Positive
## Year_2015!X.cor                                              Unconstrained
```

```
##AR1 on Y only
m5 <- asreml(Elisa ~ popmean+Year+popmean:Year+pol(X,-1)+pol(Y,-1),rcov=~at(Year):id(X):ar1(Y),random=~corgh(Year):at(pop,"Dic2 x Lloyd"):genotype+corgh(Year):at(pop,"Dic2 x Silur"):genotype+at(Year):X_Y,na.method.X="include",data=data2,maxiter=40,trace=F)
summary(m5)$varcomp
```

```
##                                                                   gamma
## Year:at(pop, Dic2 x Lloyd):genotype!Year.2015:!Year.2012.cor 0.78775303
## Year:at(pop, Dic2 x Lloyd):genotype!Year.2012                0.07337118
## Year:at(pop, Dic2 x Lloyd):genotype!Year.2015                0.13447276
## Year:at(pop, Dic2 x Silur):genotype!Year.2015:!Year.2012.cor 0.75718032
## Year:at(pop, Dic2 x Silur):genotype!Year.2012                0.08401861
## Year:at(pop, Dic2 x Silur):genotype!Year.2015                0.14848747
## at(Year, 2012):X_Y!X_Y.var                                   0.11962719
## at(Year, 2015):X_Y!X_Y.var                                   0.03448171
## Year_2012!variance                                           0.06894418
## Year_2012!Y.cor                                              0.98981897
## Year_2015!variance                                           0.01575784
## Year_2015!Y.cor                                              0.76664205
##                                                               component
## Year:at(pop, Dic2 x Lloyd):genotype!Year.2015:!Year.2012.cor 0.78775303
## Year:at(pop, Dic2 x Lloyd):genotype!Year.2012                0.07337118
## Year:at(pop, Dic2 x Lloyd):genotype!Year.2015                0.13447276
## Year:at(pop, Dic2 x Silur):genotype!Year.2015:!Year.2012.cor 0.75718032
## Year:at(pop, Dic2 x Silur):genotype!Year.2012                0.08401861
## Year:at(pop, Dic2 x Silur):genotype!Year.2015                0.14848747
## at(Year, 2012):X_Y!X_Y.var                                   0.11962719
## at(Year, 2015):X_Y!X_Y.var                                   0.03448171
## Year_2012!variance                                           0.06894418
## Year_2012!Y.cor                                              0.98981897
## Year_2015!variance                                           0.01575784
## Year_2015!Y.cor                                              0.76664205
##                                                                std.error
## Year:at(pop, Dic2 x Lloyd):genotype!Year.2015:!Year.2012.cor 0.137220429
## Year:at(pop, Dic2 x Lloyd):genotype!Year.2012                0.025109989
## Year:at(pop, Dic2 x Lloyd):genotype!Year.2015                0.018397062
## Year:at(pop, Dic2 x Silur):genotype!Year.2015:!Year.2012.cor 0.103766912
## Year:at(pop, Dic2 x Silur):genotype!Year.2012                0.021242639
## Year:at(pop, Dic2 x Silur):genotype!Year.2015                0.020999800
## at(Year, 2012):X_Y!X_Y.var                                   0.012887853
## at(Year, 2015):X_Y!X_Y.var                                   0.007148562
## Year_2012!variance                                           0.028260176
## Year_2012!Y.cor                                              0.006629087
## Year_2015!variance                                           0.006853698
## Year_2015!Y.cor                                              0.140436036
##                                                                 z.ratio
## Year:at(pop, Dic2 x Lloyd):genotype!Year.2015:!Year.2012.cor   5.740785
## Year:at(pop, Dic2 x Lloyd):genotype!Year.2012                  2.921992
## Year:at(pop, Dic2 x Lloyd):genotype!Year.2015                  7.309470
## Year:at(pop, Dic2 x Silur):genotype!Year.2015:!Year.2012.cor   7.296934
## Year:at(pop, Dic2 x Silur):genotype!Year.2012                  3.955187
## Year:at(pop, Dic2 x Silur):genotype!Year.2015                  7.070899
## at(Year, 2012):X_Y!X_Y.var                                     9.282166
## at(Year, 2015):X_Y!X_Y.var                                     4.823587
## Year_2012!variance                                             2.439623
## Year_2012!Y.cor                                              149.314532
## Year_2015!variance                                             2.299174
## Year_2015!Y.cor                                                5.459012
##                                                                 constraint
## Year:at(pop, Dic2 x Lloyd):genotype!Year.2015:!Year.2012.cor Unconstrained
## Year:at(pop, Dic2 x Lloyd):genotype!Year.2012                     Positive
## Year:at(pop, Dic2 x Lloyd):genotype!Year.2015                     Positive
## Year:at(pop, Dic2 x Silur):genotype!Year.2015:!Year.2012.cor Unconstrained
## Year:at(pop, Dic2 x Silur):genotype!Year.2012                     Positive
## Year:at(pop, Dic2 x Silur):genotype!Year.2015                     Positive
## at(Year, 2012):X_Y!X_Y.var                                        Positive
## at(Year, 2015):X_Y!X_Y.var                                        Positive
## Year_2012!variance                                                Positive
## Year_2012!Y.cor                                              Unconstrained
## Year_2015!variance                                                Positive
## Year_2015!Y.cor                                              Unconstrained
```

```
## No spatially correlated environmental variance (model 1 for residuals)
m6 <- asreml(Elisa ~ popmean+Year+popmean:Year+pol(X,-1)+pol(Y,-1),rcov=~at(Year):id(Y):id(X),random=~corgh(Year):at(pop,"Dic2 x Lloyd"):genotype+corgh(Year):at(pop,"Dic2 x Silur"):genotype,na.method.X="include",data=data,maxiter=40,trace=F)

summary(m6)$varcomp
```

```
##                                                                   gamma
## Year:at(pop, Dic2 x Lloyd):genotype!Year.2015:!Year.2012.cor 0.99998476
## Year:at(pop, Dic2 x Lloyd):genotype!Year.2012                0.04571806
## Year:at(pop, Dic2 x Lloyd):genotype!Year.2015                0.13402822
## Year:at(pop, Dic2 x Silur):genotype!Year.2015:!Year.2012.cor 0.84942726
## Year:at(pop, Dic2 x Silur):genotype!Year.2012                0.06980710
## Year:at(pop, Dic2 x Silur):genotype!Year.2015                0.15015785
## Year_2012!variance                                           0.18655486
## Year_2015!variance                                           0.05013338
##                                                               component
## Year:at(pop, Dic2 x Lloyd):genotype!Year.2015:!Year.2012.cor 0.99998476
## Year:at(pop, Dic2 x Lloyd):genotype!Year.2012                0.04571806
## Year:at(pop, Dic2 x Lloyd):genotype!Year.2015                0.13402822
## Year:at(pop, Dic2 x Silur):genotype!Year.2015:!Year.2012.cor 0.84942726
## Year:at(pop, Dic2 x Silur):genotype!Year.2012                0.06980710
## Year:at(pop, Dic2 x Silur):genotype!Year.2015                0.15015785
## Year_2012!variance                                           0.18655486
## Year_2015!variance                                           0.05013338
##                                                                std.error
## Year:at(pop, Dic2 x Lloyd):genotype!Year.2015:!Year.2012.cor          NA
## Year:at(pop, Dic2 x Lloyd):genotype!Year.2012                0.016725624
## Year:at(pop, Dic2 x Lloyd):genotype!Year.2015                0.018762279
## Year:at(pop, Dic2 x Silur):genotype!Year.2015:!Year.2012.cor 0.133201251
## Year:at(pop, Dic2 x Silur):genotype!Year.2012                0.023281797
## Year:at(pop, Dic2 x Silur):genotype!Year.2015                0.021610097
## Year_2012!variance                                           0.013504218
## Year_2015!variance                                           0.005002001
##                                                                z.ratio
## Year:at(pop, Dic2 x Lloyd):genotype!Year.2015:!Year.2012.cor        NA
## Year:at(pop, Dic2 x Lloyd):genotype!Year.2012                 2.733414
## Year:at(pop, Dic2 x Lloyd):genotype!Year.2015                 7.143493
## Year:at(pop, Dic2 x Silur):genotype!Year.2015:!Year.2012.cor  6.377022
## Year:at(pop, Dic2 x Silur):genotype!Year.2012                 2.998355
## Year:at(pop, Dic2 x Silur):genotype!Year.2015                 6.948504
## Year_2012!variance                                           13.814563
## Year_2015!variance                                           10.022666
##                                                                 constraint
## Year:at(pop, Dic2 x Lloyd):genotype!Year.2015:!Year.2012.cor      Boundary
## Year:at(pop, Dic2 x Lloyd):genotype!Year.2012                     Positive
## Year:at(pop, Dic2 x Lloyd):genotype!Year.2015                     Positive
## Year:at(pop, Dic2 x Silur):genotype!Year.2015:!Year.2012.cor Unconstrained
## Year:at(pop, Dic2 x Silur):genotype!Year.2012                     Positive
## Year:at(pop, Dic2 x Silur):genotype!Year.2015                     Positive
## Year_2012!variance                                                Positive
## Year_2015!variance                                                Positive
```

```
## No spatially uncorrelated environmental variance (model 2 for residuals)
m7 <- asreml(Elisa ~ popmean+Year+popmean:Year+pol(X,-1)+pol(Y,-1),rcov=~at(Year):ar1(Y):ar1(X),random=~corgh(Year):at(pop,"Dic2 x Lloyd"):genotype+corgh(Year):at(pop,"Dic2 x Silur"):genotype,na.method.X="include",data=data,maxiter=40,trace=F)

AICcASRemlRmd(paste("m",0:7,sep=""))
```

```
##   modnames ParNum      LL      AIC     AICc DeltaAICc      EvRatio weight
## 1       m0     25 505.955 -961.910 -960.720     0.000 1.000000e+00  0.999
## 2       m5     23 497.084 -948.168 -947.159    13.561 8.805090e+02  0.001
## 3       m2     21 491.395 -940.790 -939.947    20.773 3.241900e+04  0.000
## 4       m3     24 482.299 -916.598 -915.500    45.220 6.597786e+09  0.000
## 5       m1     23 470.671 -895.342 -894.333    66.387 2.604677e+14  0.000
## 6       m7     23 453.873 -861.746 -860.737    99.983 5.140822e+21  0.000
## 7       m6     19 434.776 -831.552 -830.860   129.860 1.580304e+28  0.000
## 8       m4     23 438.635 -831.270 -830.261   130.459 2.132121e+28  0.000
##                                                     fixed
## 1 popmean + Year + popmean:Year + pol(X, -1) + pol(Y, -1)
## 2 popmean + Year + popmean:Year + pol(X, -1) + pol(Y, -1)
## 3 popmean + Year + popmean:Year + pol(X, -1) + pol(Y, -1)
## 4 popmean + Year + popmean:Year + pol(X, -1) + pol(Y, -1)
## 5 popmean + Year + popmean:Year + pol(X, -1) + pol(Y, -1)
## 6 popmean + Year + popmean:Year + pol(X, -1) + pol(Y, -1)
## 7 popmean + Year + popmean:Year + pol(X, -1) + pol(Y, -1)
## 8 popmean + Year + popmean:Year + pol(X, -1) + pol(Y, -1)
##                                                                                                        Gside
## 1 corgh(Year):at(pop, "Dic2 x Silur"):genotype + corgh(Year):at(pop, "Dic2 x Lloyd"):genotype + at(Year):X_Y
## 2 corgh(Year):at(pop, "Dic2 x Lloyd"):genotype + corgh(Year):at(pop, "Dic2 x Silur"):genotype + at(Year):X_Y
## 3                         at(pop, "Dic2 x Lloyd"):genotype + at(pop, "Dic2 x Silur"):genotype + at(Year):X_Y
## 4          corgh(Year):at(pop, "Dic2 x Lloyd"):genotype + corgh(Year):at(pop, "Dic2 x Silur"):genotype + Obs
## 5   diag(Year):at(pop, "Dic2 x Lloyd"):genotype + diag(Year):at(pop, "Dic2 x Silur"):genotype + at(Year):X_Y
## 6                corgh(Year):at(pop, "Dic2 x Lloyd"):genotype + corgh(Year):at(pop, "Dic2 x Silur"):genotype
## 7                corgh(Year):at(pop, "Dic2 x Lloyd"):genotype + corgh(Year):at(pop, "Dic2 x Silur"):genotype
## 8 corgh(Year):at(pop, "Dic2 x Lloyd"):genotype + corgh(Year):at(pop, "Dic2 x Silur"):genotype + at(Year):X_Y
##                     Rside
## 1 ~at(Year):ar1(Y):ar1(X)
## 2  ~at(Year):id(X):ar1(Y)
## 3 ~at(Year):ar1(Y):ar1(X)
## 4 ~at(Year):ar1(Y):ar1(X)
## 5 ~at(Year):ar1(Y):ar1(X)
## 6 ~at(Year):ar1(Y):ar1(X)
## 7   ~at(Year):id(Y):id(X)
## 8  ~at(Year):id(Y):ar1(X)
```

```
filename<-paste('AICcTable_ASReml-R_Analyses_', Sys.Date(),".csv",sep="")
file.rename(from=filename, to=paste('AICcTable_ASReml-R_Analyses_', Sys.Date(),"Elisa",".csv",sep=""))
```

```
## [1] TRUE
```

```
summary(m0)$varcomp
```

```
##                                                                   gamma
## Year:at(pop, Dic2 x Silur):genotype!Year.2015:!Year.2012.cor 0.76726707
## Year:at(pop, Dic2 x Silur):genotype!Year.2012                0.08373045
## Year:at(pop, Dic2 x Silur):genotype!Year.2015                0.14488006
## Year:at(pop, Dic2 x Lloyd):genotype!Year.2015:!Year.2012.cor 0.79259441
## Year:at(pop, Dic2 x Lloyd):genotype!Year.2012                0.07145543
## Year:at(pop, Dic2 x Lloyd):genotype!Year.2015                0.13135315
## at(Year, 2012):X_Y!X_Y.var                                   0.12157232
## at(Year, 2015):X_Y!X_Y.var                                   0.03906296
## Year_2012!variance                                           0.07981287
## Year_2012!Y.cor                                              0.99337414
## Year_2012!X.cor                                              0.40213251
## Year_2015!variance                                           0.01898157
## Year_2015!Y.cor                                              0.91994742
## Year_2015!X.cor                                              0.85880638
##                                                               component
## Year:at(pop, Dic2 x Silur):genotype!Year.2015:!Year.2012.cor 0.76726707
## Year:at(pop, Dic2 x Silur):genotype!Year.2012                0.08373045
## Year:at(pop, Dic2 x Silur):genotype!Year.2015                0.14488006
## Year:at(pop, Dic2 x Lloyd):genotype!Year.2015:!Year.2012.cor 0.79259441
## Year:at(pop, Dic2 x Lloyd):genotype!Year.2012                0.07145543
## Year:at(pop, Dic2 x Lloyd):genotype!Year.2015                0.13135315
## at(Year, 2012):X_Y!X_Y.var                                   0.12157232
## at(Year, 2015):X_Y!X_Y.var                                   0.03906296
## Year_2012!variance                                           0.07981287
## Year_2012!Y.cor                                              0.99337414
## Year_2012!X.cor                                              0.40213251
## Year_2015!variance                                           0.01898157
## Year_2015!Y.cor                                              0.91994742
## Year_2015!X.cor                                              0.85880638
##                                                                std.error
## Year:at(pop, Dic2 x Silur):genotype!Year.2015:!Year.2012.cor 0.103382458
## Year:at(pop, Dic2 x Silur):genotype!Year.2012                0.021219200
## Year:at(pop, Dic2 x Silur):genotype!Year.2015                0.020464606
## Year:at(pop, Dic2 x Lloyd):genotype!Year.2015:!Year.2012.cor 0.140231764
## Year:at(pop, Dic2 x Lloyd):genotype!Year.2012                0.025018224
## Year:at(pop, Dic2 x Lloyd):genotype!Year.2015                0.017918229
## at(Year, 2012):X_Y!X_Y.var                                   0.012858728
## at(Year, 2015):X_Y!X_Y.var                                   0.004844235
## Year_2012!variance                                           0.041840428
## Year_2012!Y.cor                                              0.005010018
## Year_2012!X.cor                                              0.301631899
## Year_2015!variance                                           0.010080950
## Year_2015!Y.cor                                              0.067463505
## Year_2015!X.cor                                              0.105596953
##                                                                 z.ratio
## Year:at(pop, Dic2 x Silur):genotype!Year.2015:!Year.2012.cor   7.421637
## Year:at(pop, Dic2 x Silur):genotype!Year.2012                  3.945976
## Year:at(pop, Dic2 x Silur):genotype!Year.2015                  7.079543
## Year:at(pop, Dic2 x Lloyd):genotype!Year.2015:!Year.2012.cor   5.652032
## Year:at(pop, Dic2 x Lloyd):genotype!Year.2012                  2.856135
## Year:at(pop, Dic2 x Lloyd):genotype!Year.2015                  7.330699
## at(Year, 2012):X_Y!X_Y.var                                     9.454460
## at(Year, 2015):X_Y!X_Y.var                                     8.063803
## Year_2012!variance                                             1.907554
## Year_2012!Y.cor                                              198.277573
## Year_2012!X.cor                                                1.333190
## Year_2015!variance                                             1.882914
## Year_2015!Y.cor                                               13.636223
## Year_2015!X.cor                                                8.132871
##                                                                 constraint
## Year:at(pop, Dic2 x Silur):genotype!Year.2015:!Year.2012.cor Unconstrained
## Year:at(pop, Dic2 x Silur):genotype!Year.2012                     Positive
## Year:at(pop, Dic2 x Silur):genotype!Year.2015                     Positive
## Year:at(pop, Dic2 x Lloyd):genotype!Year.2015:!Year.2012.cor Unconstrained
## Year:at(pop, Dic2 x Lloyd):genotype!Year.2012                     Positive
## Year:at(pop, Dic2 x Lloyd):genotype!Year.2015                     Positive
## at(Year, 2012):X_Y!X_Y.var                                        Positive
## at(Year, 2015):X_Y!X_Y.var                                        Positive
## Year_2012!variance                                                Positive
## Year_2012!Y.cor                                              Unconstrained
## Year_2012!X.cor                                              Unconstrained
## Year_2015!variance                                                Positive
## Year_2015!Y.cor                                              Unconstrained
## Year_2015!X.cor                                              Unconstrained
```

```
## Goodness of fit
plot(m0)
```

```
plot.asrVariogram(variogram(m0))
```

```
hist(m0$residuals,main="Autocorrelated residual distribution")
```

```
hist(m0$coefficient$random[grepl("X_Y",names(m0$coefficient$random))&m0$coefficient$random!=0],main="Non-autocorrelated residual  distribution")
```

```
## Check spatial auto-correlation
y <- -100:100*0.1
plot(y,summary(m0)$varcomp[10,2]^abs(y))
```

```
x <- -10:10*2
plot(x,summary(m0)$varcomp[11,2]^abs(x))
```

```
y <- -100:100*0.1
plot(y,summary(m0)$varcomp[13,2]^abs(y))
```

```
x <- -10:10*2
plot(x,summary(m0)$varcomp[14,2]^abs(x))
```

## 5.2 Fixed effects for Elisa

```
## Fixed effects
m0 <- asreml(Elisa ~ popmean+Year+popmean:Year+pol(X,-1)+pol(Y,-1),rcov=~at(Year):ar1(Y):ar1(X),random=~corgh(Year):at(pop,"Dic2 x Silur"):genotype+corgh(Year):at(pop,"Dic2 x Lloyd"):genotype+at(Year):X_Y,na.method.X="include",data=data,maxiter=100,trace=F)


m0$coefficients$fixed
```

```
##     popmean_DLDS:Year_2012     popmean_DLDS:Year_2015 
##                 0.00000000                 0.00000000 
## popmean_pescadou:Year_2012 popmean_pescadou:Year_2015 
##                 0.00000000                -0.05913928 
##          pol(Y, -1)_order1          pol(X, -1)_order1 
##                 0.01767357                -0.01150250 
##                  Year_2012                  Year_2015 
##                 0.00000000                -0.23085203 
##               popmean_DLDS           popmean_pescadou 
##                 0.00000000                 0.40149127 
##                (Intercept) 
##                 0.90902859
```

```
##Conditional Wald F-test to test factors and their interaction
wald.asreml(m0, ssType="conditional", denDF="numeric")
```

```
## $Wald
##              Df denDF     F.inc     F.con Margin           Pr
## (Intercept)   1   2.2 290.90000 248.60000        2.731397e-03
## popmean       1 678.6 196.70000 197.40000      A 1.540918e-39
## Year          1   3.2   3.88800   3.18200      A 1.676837e-01
## pol(X, -1)    1   4.2   0.03951   0.02688      B 8.773377e-01
## pol(Y, -1)    1   7.1   0.09758   0.08228      B 7.824047e-01
## popmean:Year  1 575.7   1.83300   1.83300      B 1.763176e-01
## 
## $stratumVariances
## NULL
```

```
##Predictions for the interaction
pred <- predict(m0,classify="popmean:Year",sed=list("popmean:Year"=T))
```

```
## Predict terminating with 2 errors
## Error flags: 7 9
```

```
## Warning: Abnormal termination
## LogLikelihood not converged
## Results may be erroneous
```

```
pred$predictions
```

```
## $pvals
## 
## Notes:
## 
##    popmean Year predicted.value standard.error est.status
## 1     DLDS 2012               0              0         NA
## 2     DLDS 2015               0              0         NA
## 3 pescadou 2012               0              0         NA
## 4 pescadou 2015               0              0         NA
## 
## $sed
##      [,1] [,2] [,3] [,4]
## [1,]    0    0    0    0
## [2,]    0    0    0    0
## [3,]    0    0    0    0
## [4,]    0    0    0    0
## 
## $avsed
##  min mean  max 
##    0    0    0
```

```
## Covariance between estimates
svc(m0)
```

```
##                                                                    df
## Year:at(pop, Dic2 x Silur):genotype!Year.2015:!Year.2012.cor    80.45
## Year:at(pop, Dic2 x Silur):genotype!Year.2012                  189.86
## Year:at(pop, Dic2 x Silur):genotype!Year.2015                  136.84
## Year:at(pop, Dic2 x Lloyd):genotype!Year.2015:!Year.2012.cor   436.77
## Year:at(pop, Dic2 x Lloyd):genotype!Year.2012                  555.05
## Year:at(pop, Dic2 x Lloyd):genotype!Year.2015                  123.97
## at(Year, 2012):X_Y!X_Y.var                                    2835.11
## at(Year, 2015):X_Y!X_Y.var                                       4.58
## Year_2012!variance                                           41478.77
## Year_2012!Y.cor                                              90064.04
## Year_2012!X.cor                                                  4.03
## Year_2015!variance                                             139.31
## Year_2015!Y.cor                                                239.64
## Year_2015!X.cor                                                132.29
##                                                              variance
## Year:at(pop, Dic2 x Silur):genotype!Year.2015:!Year.2012.cor    27.83
## Year:at(pop, Dic2 x Silur):genotype!Year.2012                 -436.41
## Year:at(pop, Dic2 x Silur):genotype!Year.2015                  -22.46
## Year:at(pop, Dic2 x Lloyd):genotype!Year.2015:!Year.2012.cor    20.73
## Year:at(pop, Dic2 x Lloyd):genotype!Year.2012                 -183.38
## Year:at(pop, Dic2 x Lloyd):genotype!Year.2015                  -11.45
## at(Year, 2012):X_Y!X_Y.var                                    -404.07
## at(Year, 2015):X_Y!X_Y.var                                      -0.50
## Year_2012!variance                                            1619.52
## Year_2012!Y.cor                                              -1731.99
## Year_2012!X.cor                                                 13.80
## Year_2015!variance                                               2.21
## Year_2015!Y.cor                                                 -1.59
## Year_2015!X.cor                                                  0.86
##                                                              Year:at(pop, Dic2 x Silur):genotype!Year.2015:!Year.2012.cor
## Year:at(pop, Dic2 x Silur):genotype!Year.2015:!Year.2012.cor                                                        48.02
## Year:at(pop, Dic2 x Silur):genotype!Year.2012                                                                        0.00
## Year:at(pop, Dic2 x Silur):genotype!Year.2015                                                                        0.00
## Year:at(pop, Dic2 x Lloyd):genotype!Year.2015:!Year.2012.cor                                                         0.00
## Year:at(pop, Dic2 x Lloyd):genotype!Year.2012                                                                        0.00
## Year:at(pop, Dic2 x Lloyd):genotype!Year.2015                                                                        0.00
## at(Year, 2012):X_Y!X_Y.var                                                                                           0.00
## at(Year, 2015):X_Y!X_Y.var                                                                                           0.00
## Year_2012!variance                                                                                                   0.00
## Year_2012!Y.cor                                                                                                      0.00
## Year_2012!X.cor                                                                                                      0.00
## Year_2015!variance                                                                                                   0.00
## Year_2015!Y.cor                                                                                                      0.00
## Year_2015!X.cor                                                                                                      0.00
##                                                              Year:at(pop, Dic2 x Silur):genotype!Year.2012
## Year:at(pop, Dic2 x Silur):genotype!Year.2015:!Year.2012.cor                                         45.88
## Year:at(pop, Dic2 x Silur):genotype!Year.2012                                                      2376.67
## Year:at(pop, Dic2 x Silur):genotype!Year.2015                                                         0.00
## Year:at(pop, Dic2 x Lloyd):genotype!Year.2015:!Year.2012.cor                                          0.00
## Year:at(pop, Dic2 x Lloyd):genotype!Year.2012                                                         0.00
## Year:at(pop, Dic2 x Lloyd):genotype!Year.2015                                                         0.00
## at(Year, 2012):X_Y!X_Y.var                                                                            0.00
## at(Year, 2015):X_Y!X_Y.var                                                                            0.00
## Year_2012!variance                                                                                    0.00
## Year_2012!Y.cor                                                                                       0.00
## Year_2012!X.cor                                                                                       0.00
## Year_2015!variance                                                                                    0.00
## Year_2015!Y.cor                                                                                       0.00
## Year_2015!X.cor                                                                                       0.00
##                                                              Year:at(pop, Dic2 x Silur):genotype!Year.2015
## Year:at(pop, Dic2 x Silur):genotype!Year.2015:!Year.2012.cor                                        -36.89
## Year:at(pop, Dic2 x Silur):genotype!Year.2012                                                      -510.90
## Year:at(pop, Dic2 x Silur):genotype!Year.2015                                                      -134.84
## Year:at(pop, Dic2 x Lloyd):genotype!Year.2015:!Year.2012.cor                                          0.00
## Year:at(pop, Dic2 x Lloyd):genotype!Year.2012                                                         0.00
## Year:at(pop, Dic2 x Lloyd):genotype!Year.2015                                                         0.00
## at(Year, 2012):X_Y!X_Y.var                                                                            0.00
## at(Year, 2015):X_Y!X_Y.var                                                                            0.00
## Year_2012!variance                                                                                    0.00
## Year_2012!Y.cor                                                                                       0.00
## Year_2012!X.cor                                                                                       0.00
## Year_2015!variance                                                                                    0.00
## Year_2015!Y.cor                                                                                       0.00
## Year_2015!X.cor                                                                                       0.00
##                                                              Year:at(pop, Dic2 x Lloyd):genotype!Year.2015:!Year.2012.cor
## Year:at(pop, Dic2 x Silur):genotype!Year.2015:!Year.2012.cor                                                         0.32
## Year:at(pop, Dic2 x Silur):genotype!Year.2012                                                                       -2.46
## Year:at(pop, Dic2 x Silur):genotype!Year.2015                                                                        0.11
## Year:at(pop, Dic2 x Lloyd):genotype!Year.2015:!Year.2012.cor                                                        12.90
## Year:at(pop, Dic2 x Lloyd):genotype!Year.2012                                                                        0.00
## Year:at(pop, Dic2 x Lloyd):genotype!Year.2015                                                                        0.00
## at(Year, 2012):X_Y!X_Y.var                                                                                           0.00
## at(Year, 2015):X_Y!X_Y.var                                                                                           0.00
## Year_2012!variance                                                                                                   0.00
## Year_2012!Y.cor                                                                                                      0.00
## Year_2012!X.cor                                                                                                      0.00
## Year_2015!variance                                                                                                   0.00
## Year_2015!Y.cor                                                                                                      0.00
## Year_2015!X.cor                                                                                                      0.00
##                                                              Year:at(pop, Dic2 x Lloyd):genotype!Year.2012
## Year:at(pop, Dic2 x Silur):genotype!Year.2015:!Year.2012.cor                                         -2.06
## Year:at(pop, Dic2 x Silur):genotype!Year.2012                                                         1.29
## Year:at(pop, Dic2 x Silur):genotype!Year.2015                                                         0.54
## Year:at(pop, Dic2 x Lloyd):genotype!Year.2015:!Year.2012.cor                                         28.21
## Year:at(pop, Dic2 x Lloyd):genotype!Year.2012                                                       527.74
## Year:at(pop, Dic2 x Lloyd):genotype!Year.2015                                                         0.00
## at(Year, 2012):X_Y!X_Y.var                                                                            0.00
## at(Year, 2015):X_Y!X_Y.var                                                                            0.00
## Year_2012!variance                                                                                    0.00
## Year_2012!Y.cor                                                                                       0.00
## Year_2012!X.cor                                                                                       0.00
## Year_2015!variance                                                                                    0.00
## Year_2015!Y.cor                                                                                       0.00
## Year_2015!X.cor                                                                                       0.00
##                                                              Year:at(pop, Dic2 x Lloyd):genotype!Year.2015
## Year:at(pop, Dic2 x Silur):genotype!Year.2015:!Year.2012.cor                                         -1.46
## Year:at(pop, Dic2 x Silur):genotype!Year.2012                                                        -5.19
## Year:at(pop, Dic2 x Silur):genotype!Year.2015                                                        -1.30
## Year:at(pop, Dic2 x Lloyd):genotype!Year.2015:!Year.2012.cor                                        -11.94
## Year:at(pop, Dic2 x Lloyd):genotype!Year.2012                                                      -105.12
## Year:at(pop, Dic2 x Lloyd):genotype!Year.2015                                                       -83.13
## at(Year, 2012):X_Y!X_Y.var                                                                            0.00
## at(Year, 2015):X_Y!X_Y.var                                                                            0.00
## Year_2012!variance                                                                                    0.00
## Year_2012!Y.cor                                                                                       0.00
## Year_2012!X.cor                                                                                       0.00
## Year_2015!variance                                                                                    0.00
## Year_2015!Y.cor                                                                                       0.00
## Year_2015!X.cor                                                                                       0.00
##                                                              at(Year, 2012):X_Y!X_Y.var
## Year:at(pop, Dic2 x Silur):genotype!Year.2015:!Year.2012.cor                    -131.31
## Year:at(pop, Dic2 x Silur):genotype!Year.2012                                   1658.78
## Year:at(pop, Dic2 x Silur):genotype!Year.2015                                     -0.45
## Year:at(pop, Dic2 x Lloyd):genotype!Year.2015:!Year.2012.cor                     -45.92
## Year:at(pop, Dic2 x Lloyd):genotype!Year.2012                                    556.76
## Year:at(pop, Dic2 x Lloyd):genotype!Year.2015                                     -0.02
## at(Year, 2012):X_Y!X_Y.var                                                       851.65
## at(Year, 2015):X_Y!X_Y.var                                                         0.00
## Year_2012!variance                                                                 0.00
## Year_2012!Y.cor                                                                    0.00
## Year_2012!X.cor                                                                    0.00
## Year_2015!variance                                                                 0.00
## Year_2015!Y.cor                                                                    0.00
## Year_2015!X.cor                                                                    0.00
##                                                              at(Year, 2015):X_Y!X_Y.var
## Year:at(pop, Dic2 x Silur):genotype!Year.2015:!Year.2012.cor                    -142.38
## Year:at(pop, Dic2 x Silur):genotype!Year.2012                                   -421.55
## Year:at(pop, Dic2 x Silur):genotype!Year.2015                                   -113.53
## Year:at(pop, Dic2 x Lloyd):genotype!Year.2015:!Year.2012.cor                     -44.44
## Year:at(pop, Dic2 x Lloyd):genotype!Year.2012                                    -93.91
## Year:at(pop, Dic2 x Lloyd):genotype!Year.2015                                    -72.90
## at(Year, 2012):X_Y!X_Y.var                                                        -2.79
## at(Year, 2015):X_Y!X_Y.var                                                       -75.23
## Year_2012!variance                                                                 0.00
## Year_2012!Y.cor                                                                    0.00
## Year_2012!X.cor                                                                    0.00
## Year_2015!variance                                                                 0.00
## Year_2015!Y.cor                                                                    0.00
## Year_2015!X.cor                                                                    0.00
##                                                              Year_2012!variance
## Year:at(pop, Dic2 x Silur):genotype!Year.2015:!Year.2012.cor              -1.85
## Year:at(pop, Dic2 x Silur):genotype!Year.2012                             69.78
## Year:at(pop, Dic2 x Silur):genotype!Year.2015                              0.02
## Year:at(pop, Dic2 x Lloyd):genotype!Year.2015:!Year.2012.cor              -0.81
## Year:at(pop, Dic2 x Lloyd):genotype!Year.2012                             15.35
## Year:at(pop, Dic2 x Lloyd):genotype!Year.2015                              0.04
## at(Year, 2012):X_Y!X_Y.var                                                55.75
## at(Year, 2015):X_Y!X_Y.var                                                -0.02
## Year_2012!variance                                                      -388.54
## Year_2012!Y.cor                                                            0.00
## Year_2012!X.cor                                                            0.00
## Year_2015!variance                                                         0.00
## Year_2015!Y.cor                                                            0.00
## Year_2015!X.cor                                                            0.00
##                                                              Year_2012!Y.cor
## Year:at(pop, Dic2 x Silur):genotype!Year.2015:!Year.2012.cor            8.60
## Year:at(pop, Dic2 x Silur):genotype!Year.2012                        -762.17
## Year:at(pop, Dic2 x Silur):genotype!Year.2015                          -1.41
## Year:at(pop, Dic2 x Lloyd):genotype!Year.2015:!Year.2012.cor           15.93
## Year:at(pop, Dic2 x Lloyd):genotype!Year.2012                        -277.71
## Year:at(pop, Dic2 x Lloyd):genotype!Year.2015                          -0.21
## at(Year, 2012):X_Y!X_Y.var                                           -514.59
## at(Year, 2015):X_Y!X_Y.var                                              0.17
## Year_2012!variance                                                   1652.64
## Year_2012!Y.cor                                                     -1748.93
## Year_2012!X.cor                                                         0.00
## Year_2015!variance                                                      0.00
## Year_2015!Y.cor                                                         0.00
## Year_2015!X.cor                                                         0.00
##                                                              Year_2012!X.cor
## Year:at(pop, Dic2 x Silur):genotype!Year.2015:!Year.2012.cor            0.28
## Year:at(pop, Dic2 x Silur):genotype!Year.2012                          -3.91
## Year:at(pop, Dic2 x Silur):genotype!Year.2015                          -0.02
## Year:at(pop, Dic2 x Lloyd):genotype!Year.2015:!Year.2012.cor            0.02
## Year:at(pop, Dic2 x Lloyd):genotype!Year.2012                           0.26
## Year:at(pop, Dic2 x Lloyd):genotype!Year.2015                           0.00
## at(Year, 2012):X_Y!X_Y.var                                             -3.36
## at(Year, 2015):X_Y!X_Y.var                                              0.01
## Year_2012!variance                                                     19.36
## Year_2012!Y.cor                                                        10.57
## Year_2012!X.cor                                                        32.23
## Year_2015!variance                                                      0.00
## Year_2015!Y.cor                                                         0.00
## Year_2015!X.cor                                                         0.00
##                                                              Year_2015!variance
## Year:at(pop, Dic2 x Silur):genotype!Year.2015:!Year.2012.cor             -22.57
## Year:at(pop, Dic2 x Silur):genotype!Year.2012                            -51.51
## Year:at(pop, Dic2 x Silur):genotype!Year.2015                            -17.29
## Year:at(pop, Dic2 x Lloyd):genotype!Year.2015:!Year.2012.cor              -8.26
## Year:at(pop, Dic2 x Lloyd):genotype!Year.2012                            -18.82
## Year:at(pop, Dic2 x Lloyd):genotype!Year.2015                            -15.36
## at(Year, 2012):X_Y!X_Y.var                                                -4.55
## at(Year, 2015):X_Y!X_Y.var                                               -13.93
## Year_2012!variance                                                       -10.46
## Year_2012!Y.cor                                                            5.88
## Year_2012!X.cor                                                           19.61
## Year_2015!variance                                                       -34.59
## Year_2015!Y.cor                                                            0.00
## Year_2015!X.cor                                                            0.00
##                                                              Year_2015!Y.cor
## Year:at(pop, Dic2 x Silur):genotype!Year.2015:!Year.2012.cor            5.61
## Year:at(pop, Dic2 x Silur):genotype!Year.2012                           9.99
## Year:at(pop, Dic2 x Silur):genotype!Year.2015                           2.71
## Year:at(pop, Dic2 x Lloyd):genotype!Year.2015:!Year.2012.cor            0.99
## Year:at(pop, Dic2 x Lloyd):genotype!Year.2012                           2.97
## Year:at(pop, Dic2 x Lloyd):genotype!Year.2015                           2.12
## at(Year, 2012):X_Y!X_Y.var                                             -0.20
## at(Year, 2015):X_Y!X_Y.var                                              1.82
## Year_2012!variance                                                      0.43
## Year_2012!Y.cor                                                         0.14
## Year_2012!X.cor                                                        -0.43
## Year_2015!variance                                                      2.19
## Year_2015!Y.cor                                                        -2.66
## Year_2015!X.cor                                                         0.00
##                                                              Year_2015!X.cor
## Year:at(pop, Dic2 x Silur):genotype!Year.2015:!Year.2012.cor               1
## Year:at(pop, Dic2 x Silur):genotype!Year.2012                              1
## Year:at(pop, Dic2 x Silur):genotype!Year.2015                              1
## Year:at(pop, Dic2 x Lloyd):genotype!Year.2015:!Year.2012.cor               1
## Year:at(pop, Dic2 x Lloyd):genotype!Year.2012                              1
## Year:at(pop, Dic2 x Lloyd):genotype!Year.2015                              1
## at(Year, 2012):X_Y!X_Y.var                                                 1
## at(Year, 2015):X_Y!X_Y.var                                                 1
## Year_2012!variance                                                         1
## Year_2012!Y.cor                                                            1
## Year_2012!X.cor                                                            1
## Year_2015!variance                                                         1
## Year_2015!Y.cor                                                            1
## Year_2015!X.cor                                                            1
```

## 5.3 Compute genetic correlation for Elisa

```
m0 <- asreml(Elisa ~ -1+popmean,rcov=~at(Year):ar1(Y):ar1(X),random=~corgh(Year):at(pop,"Dic2 x Lloyd"):genotype+corgh(Year):at(pop,"Dic2 x Silur"):genotype+at(Year):X_Y,na.method.X="include",data=data,maxiter=40,trace=F)
```

```
## LogLikelihood not converged
```

```
m0 <- update(m0)
```

```
## LogLikelihood not converged
```

```
m0 <- update(m0)
```

```
## LogLikelihood not converged
```

```
summary(m0)$varcomp[c(1,4),2:3]
```

```
##                                                              component
## Year:at(pop, Dic2 x Lloyd):genotype!Year.2015:!Year.2012.cor 0.7846853
## Year:at(pop, Dic2 x Silur):genotype!Year.2015:!Year.2012.cor 0.7790734
##                                                              std.error
## Year:at(pop, Dic2 x Lloyd):genotype!Year.2015:!Year.2012.cor 0.1398226
## Year:at(pop, Dic2 x Silur):genotype!Year.2015:!Year.2012.cor 0.1028380
```

## 5.4 Compute heritabilities for Elisa

```
## Heritability Dic2 x Lloyd 2012
summary(m0)$varcomp[2,2]/sum(summary(m0)$varcomp[c(2,7),2])
```

```
## [1] 0.3709113
```

```
Heritability <- as.numeric(pin(m0,prop~(V2)/(V2+V7)))
CI<-c(Heritability[1]-qnorm(0.975)* Heritability[2], Heritability[1]+qnorm(0.975)* Heritability[2])
names(CI) <- c("Lower95%CI","Upper95%CI")
CI
```

```
## Lower95%CI Upper95%CI 
##  0.1811381  0.5606844
```

```
## Heritability Dic2 x Lloyd 2015
summary(m0)$varcomp[3,2]/sum(summary(m0)$varcomp[c(3,8),2])
```

```
## [1] 0.7720964
```

```
Heritability <- as.numeric(pin(m0,prop~(V3)/(V3+V8)))
CI<-c(Heritability[1]-qnorm(0.975)* Heritability[2], Heritability[1]+qnorm(0.975)* Heritability[2])
names(CI) <- c("Lower95%CI","Upper95%CI")
CI
```

```
## Lower95%CI Upper95%CI 
##  0.7018252  0.8423675
```

```
## Heritability Dic2 x Silur 2012
summary(m0)$varcomp[5,2]/sum(summary(m0)$varcomp[c(5,7),2])
```

```
## [1] 0.4062105
```

```
Heritability <- as.numeric(pin(m0,prop~(V5)/(V5+V7)))
CI<-c(Heritability[1]-qnorm(0.975)* Heritability[2], Heritability[1]+qnorm(0.975)* Heritability[2])
names(CI) <- c("Lower95%CI","Upper95%CI")
CI
```

```
## Lower95%CI Upper95%CI 
##  0.2586374  0.5537837
```

```
## Heritability Dic2 x Silur 2015
summary(m0)$varcomp[6,2]/sum(summary(m0)$varcomp[c(6,8),2])
```

```
## [1] 0.78727
```

```
Heritability <- as.numeric(pin(m0,prop~(V6)/(V6+V8)))
CI<-c(Heritability[1]-qnorm(0.975)* Heritability[2], Heritability[1]+qnorm(0.975)* Heritability[2])
names(CI) <- c("Lower95%CI","Upper95%CI")
CI
```

```
## Lower95%CI Upper95%CI 
##  0.7196401  0.8548999
```

## 5.5 Proportion of total variance explained by spatial autocorrelation for Elisa

```
## Spatial autocorrelation Dic2 x Lloyd 2012
summary(m0)$varcomp[9,2]/sum(summary(m0)$varcomp[c(2,7,9),2])
```

```
## [1] 0.3489646
```

```
## Spatial autocorrelation Dic2 x Silur 2012
summary(m0)$varcomp[9,2]/sum(summary(m0)$varcomp[c(5,7,9),2])
```

```
## [1] 0.335962
```

```
## Spatial autocorrelation Dic2 x Lloyd 2012
summary(m0)$varcomp[12,2]/sum(summary(m0)$varcomp[c(3,8,12),2])
```

```
## [1] 0.08297877
```

```
## Spatial autocorrelation  Dic2 x Silur 2015
summary(m0)$varcomp[12,2]/sum(summary(m0)$varcomp[c(6,8,12),2])
```

```
## [1] 0.0778844
```

## 5.6 Extract BLUPs

```
hist(m0$coefficient$random[grepl("genotype",names(m0$coefficient$random))&m0$coefficient$random!=0],main="BLUP distribution")
```

```
## 357 genotypes in total
length(unique(data$genotype[data$cross=="cross"&!is.na(data$cross)&!is.na(data$Elisa)]))
```

```
## [1] 357
```

```
## 15 genotypes without replication
tab <- table(data$genotype[data$cross=="cross"&!is.na(data$cross)&!is.na(data$Elisa)])
length(tab[tab==1])
```

```
## [1] 15
```

```
## Extract BLUPS
coefElisa <- m0$coefficient$random[grepl("genotype",names(m0$coefficient$random))&m0$coefficient$random!=0]
## Extract genotype, pop, year
coefElisaname <- strsplit(names(coefElisa),":")
coefElisaname <- lapply(coefElisaname,function(x){strsplit(x,"_")})

year = do.call("rbind", lapply(coefElisaname, "[[", 1))
pop = do.call("rbind", lapply(coefElisaname, "[[", 2))
geno = do.call("rbind", lapply(coefElisaname, "[[", 3))

BLUPElisa <- data.frame(pop[,3],geno[,2],year[,2],coefElisa)

names(BLUPElisa) <- c("pop","geno","year","BLUP")
head(BLUPElisa)
```

```
##                                                                                    pop
## Year_2012:at(pop, Dic2 x Lloyd)_pop_Dic2 x Lloyd:genotype_BX07.2.1f1.1.1  Dic2 x Lloyd
## Year_2012:at(pop, Dic2 x Lloyd)_pop_Dic2 x Lloyd:genotype_BX07.2.1f1.1.2  Dic2 x Lloyd
## Year_2012:at(pop, Dic2 x Lloyd)_pop_Dic2 x Lloyd:genotype_BX07.2.1f1.10.1 Dic2 x Lloyd
## Year_2012:at(pop, Dic2 x Lloyd)_pop_Dic2 x Lloyd:genotype_BX07.2.1f1.10.2 Dic2 x Lloyd
## Year_2012:at(pop, Dic2 x Lloyd)_pop_Dic2 x Lloyd:genotype_BX07.2.1f1.11.1 Dic2 x Lloyd
## Year_2012:at(pop, Dic2 x Lloyd)_pop_Dic2 x Lloyd:genotype_BX07.2.1f1.11.2 Dic2 x Lloyd
##                                                                                      geno
## Year_2012:at(pop, Dic2 x Lloyd)_pop_Dic2 x Lloyd:genotype_BX07.2.1f1.1.1   BX07.2.1f1.1.1
## Year_2012:at(pop, Dic2 x Lloyd)_pop_Dic2 x Lloyd:genotype_BX07.2.1f1.1.2   BX07.2.1f1.1.2
## Year_2012:at(pop, Dic2 x Lloyd)_pop_Dic2 x Lloyd:genotype_BX07.2.1f1.10.1 BX07.2.1f1.10.1
## Year_2012:at(pop, Dic2 x Lloyd)_pop_Dic2 x Lloyd:genotype_BX07.2.1f1.10.2 BX07.2.1f1.10.2
## Year_2012:at(pop, Dic2 x Lloyd)_pop_Dic2 x Lloyd:genotype_BX07.2.1f1.11.1 BX07.2.1f1.11.1
## Year_2012:at(pop, Dic2 x Lloyd)_pop_Dic2 x Lloyd:genotype_BX07.2.1f1.11.2 BX07.2.1f1.11.2
##                                                                           year
## Year_2012:at(pop, Dic2 x Lloyd)_pop_Dic2 x Lloyd:genotype_BX07.2.1f1.1.1  2012
## Year_2012:at(pop, Dic2 x Lloyd)_pop_Dic2 x Lloyd:genotype_BX07.2.1f1.1.2  2012
## Year_2012:at(pop, Dic2 x Lloyd)_pop_Dic2 x Lloyd:genotype_BX07.2.1f1.10.1 2012
## Year_2012:at(pop, Dic2 x Lloyd)_pop_Dic2 x Lloyd:genotype_BX07.2.1f1.10.2 2012
## Year_2012:at(pop, Dic2 x Lloyd)_pop_Dic2 x Lloyd:genotype_BX07.2.1f1.11.1 2012
## Year_2012:at(pop, Dic2 x Lloyd)_pop_Dic2 x Lloyd:genotype_BX07.2.1f1.11.2 2012
##                                                                                  BLUP
## Year_2012:at(pop, Dic2 x Lloyd)_pop_Dic2 x Lloyd:genotype_BX07.2.1f1.1.1  -0.25806438
## Year_2012:at(pop, Dic2 x Lloyd)_pop_Dic2 x Lloyd:genotype_BX07.2.1f1.1.2  -0.01563992
## Year_2012:at(pop, Dic2 x Lloyd)_pop_Dic2 x Lloyd:genotype_BX07.2.1f1.10.1 -0.42727275
## Year_2012:at(pop, Dic2 x Lloyd)_pop_Dic2 x Lloyd:genotype_BX07.2.1f1.10.2 -0.34656287
## Year_2012:at(pop, Dic2 x Lloyd)_pop_Dic2 x Lloyd:genotype_BX07.2.1f1.11.1  0.21092182
## Year_2012:at(pop, Dic2 x Lloyd)_pop_Dic2 x Lloyd:genotype_BX07.2.1f1.11.2 -0.12992070
```

```
## 357 genotypes
nrow(BLUPElisa[BLUPElisa$year=="2012",])
```

```
## [1] 357
```

```
nrow(BLUPElisa[BLUPElisa$year=="2015",])
```

```
## [1] 357
```

```
BLUPElisa$genotype_year <- paste(BLUPElisa$geno,BLUPElisa$year,sep="_")

BLUPElisa$real <- BLUPElisa$genotype_year%in%data$genotype_year
head(BLUPElisa[BLUPElisa$real==FALSE,])
```

```
##                                                                                    pop
## Year_2012:at(pop, Dic2 x Lloyd)_pop_Dic2 x Lloyd:genotype_BX07.2.1f1.17.1 Dic2 x Lloyd
## Year_2012:at(pop, Dic2 x Lloyd)_pop_Dic2 x Lloyd:genotype_BX07.2.1f1.46.1 Dic2 x Lloyd
## Year_2012:at(pop, Dic2 x Lloyd)_pop_Dic2 x Lloyd:genotype_BX07.2.1f2.26   Dic2 x Lloyd
## Year_2012:at(pop, Dic2 x Lloyd)_pop_Dic2 x Lloyd:genotype_BX07.2.1f2.81   Dic2 x Lloyd
## Year_2012:at(pop, Dic2 x Lloyd)_pop_Dic2 x Lloyd:genotype_BX07.2.1f2.95   Dic2 x Lloyd
## Year_2015:at(pop, Dic2 x Lloyd)_pop_Dic2 x Lloyd:genotype_BX07.2.1f1.16.1 Dic2 x Lloyd
##                                                                                      geno
## Year_2012:at(pop, Dic2 x Lloyd)_pop_Dic2 x Lloyd:genotype_BX07.2.1f1.17.1 BX07.2.1f1.17.1
## Year_2012:at(pop, Dic2 x Lloyd)_pop_Dic2 x Lloyd:genotype_BX07.2.1f1.46.1 BX07.2.1f1.46.1
## Year_2012:at(pop, Dic2 x Lloyd)_pop_Dic2 x Lloyd:genotype_BX07.2.1f2.26     BX07.2.1f2.26
## Year_2012:at(pop, Dic2 x Lloyd)_pop_Dic2 x Lloyd:genotype_BX07.2.1f2.81     BX07.2.1f2.81
## Year_2012:at(pop, Dic2 x Lloyd)_pop_Dic2 x Lloyd:genotype_BX07.2.1f2.95     BX07.2.1f2.95
## Year_2015:at(pop, Dic2 x Lloyd)_pop_Dic2 x Lloyd:genotype_BX07.2.1f1.16.1 BX07.2.1f1.16.1
##                                                                           year
## Year_2012:at(pop, Dic2 x Lloyd)_pop_Dic2 x Lloyd:genotype_BX07.2.1f1.17.1 2012
## Year_2012:at(pop, Dic2 x Lloyd)_pop_Dic2 x Lloyd:genotype_BX07.2.1f1.46.1 2012
## Year_2012:at(pop, Dic2 x Lloyd)_pop_Dic2 x Lloyd:genotype_BX07.2.1f2.26   2012
## Year_2012:at(pop, Dic2 x Lloyd)_pop_Dic2 x Lloyd:genotype_BX07.2.1f2.81   2012
## Year_2012:at(pop, Dic2 x Lloyd)_pop_Dic2 x Lloyd:genotype_BX07.2.1f2.95   2012
## Year_2015:at(pop, Dic2 x Lloyd)_pop_Dic2 x Lloyd:genotype_BX07.2.1f1.16.1 2015
##                                                                                  BLUP
## Year_2012:at(pop, Dic2 x Lloyd)_pop_Dic2 x Lloyd:genotype_BX07.2.1f1.17.1 -0.27263643
## Year_2012:at(pop, Dic2 x Lloyd)_pop_Dic2 x Lloyd:genotype_BX07.2.1f1.46.1  0.13116397
## Year_2012:at(pop, Dic2 x Lloyd)_pop_Dic2 x Lloyd:genotype_BX07.2.1f2.26    0.04633319
## Year_2012:at(pop, Dic2 x Lloyd)_pop_Dic2 x Lloyd:genotype_BX07.2.1f2.81   -0.32336483
## Year_2012:at(pop, Dic2 x Lloyd)_pop_Dic2 x Lloyd:genotype_BX07.2.1f2.95    0.10534767
## Year_2015:at(pop, Dic2 x Lloyd)_pop_Dic2 x Lloyd:genotype_BX07.2.1f1.16.1  0.10009742
##                                                                                  genotype_year
## Year_2012:at(pop, Dic2 x Lloyd)_pop_Dic2 x Lloyd:genotype_BX07.2.1f1.17.1 BX07.2.1f1.17.1_2012
## Year_2012:at(pop, Dic2 x Lloyd)_pop_Dic2 x Lloyd:genotype_BX07.2.1f1.46.1 BX07.2.1f1.46.1_2012
## Year_2012:at(pop, Dic2 x Lloyd)_pop_Dic2 x Lloyd:genotype_BX07.2.1f2.26     BX07.2.1f2.26_2012
## Year_2012:at(pop, Dic2 x Lloyd)_pop_Dic2 x Lloyd:genotype_BX07.2.1f2.81     BX07.2.1f2.81_2012
## Year_2012:at(pop, Dic2 x Lloyd)_pop_Dic2 x Lloyd:genotype_BX07.2.1f2.95     BX07.2.1f2.95_2012
## Year_2015:at(pop, Dic2 x Lloyd)_pop_Dic2 x Lloyd:genotype_BX07.2.1f1.16.1 BX07.2.1f1.16.1_2015
##                                                                            real
## Year_2012:at(pop, Dic2 x Lloyd)_pop_Dic2 x Lloyd:genotype_BX07.2.1f1.17.1 FALSE
## Year_2012:at(pop, Dic2 x Lloyd)_pop_Dic2 x Lloyd:genotype_BX07.2.1f1.46.1 FALSE
## Year_2012:at(pop, Dic2 x Lloyd)_pop_Dic2 x Lloyd:genotype_BX07.2.1f2.26   FALSE
## Year_2012:at(pop, Dic2 x Lloyd)_pop_Dic2 x Lloyd:genotype_BX07.2.1f2.81   FALSE
## Year_2012:at(pop, Dic2 x Lloyd)_pop_Dic2 x Lloyd:genotype_BX07.2.1f2.95   FALSE
## Year_2015:at(pop, Dic2 x Lloyd)_pop_Dic2 x Lloyd:genotype_BX07.2.1f1.16.1 FALSE
```

```
## Example of an unreplicated genotype
data[data$genotype=="BX07.2.1f1.17.1"&!is.na(data$genotype),]
```

```
##         plante          pop        genotype NV Elisa QPCR  X  Y
## 1239 2307-2308 Dic2 x Lloyd BX07.2.1f1.17.1  0  0.12   NA 14 26
##            neigh1        neigh2          neigh3        neigh4       neigh5
## 1239 TT06DC 44.39 BX07.2.1f2.70 BX07.2.1f1.44.1 BX07.3.2f2.84 TT06DC 38.23
##             neigh6   neigh7       neigh8   X_Y Year cross  Obs
## 1239 BX07.3.2f2.58 pescadou TT06DC 39.17 14_26 2015 cross 1239
##             genotype_year      pop2015 pop2015mean cross2015 popmean
## 1239 BX07.2.1f1.17.1_2015 Dic2 x Lloyd        DLDS     cross    DLDS
##      Year2012 Year2015
## 1239        0        1
```

```
BLUPElisa[BLUPElisa$geno=="BX07.2.1f1.17.1"&!is.na(BLUPElisa$geno),]
```

```
##                                                                                    pop
## Year_2012:at(pop, Dic2 x Lloyd)_pop_Dic2 x Lloyd:genotype_BX07.2.1f1.17.1 Dic2 x Lloyd
## Year_2015:at(pop, Dic2 x Lloyd)_pop_Dic2 x Lloyd:genotype_BX07.2.1f1.17.1 Dic2 x Lloyd
##                                                                                      geno
## Year_2012:at(pop, Dic2 x Lloyd)_pop_Dic2 x Lloyd:genotype_BX07.2.1f1.17.1 BX07.2.1f1.17.1
## Year_2015:at(pop, Dic2 x Lloyd)_pop_Dic2 x Lloyd:genotype_BX07.2.1f1.17.1 BX07.2.1f1.17.1
##                                                                           year
## Year_2012:at(pop, Dic2 x Lloyd)_pop_Dic2 x Lloyd:genotype_BX07.2.1f1.17.1 2012
## Year_2015:at(pop, Dic2 x Lloyd)_pop_Dic2 x Lloyd:genotype_BX07.2.1f1.17.1 2015
##                                                                                 BLUP
## Year_2012:at(pop, Dic2 x Lloyd)_pop_Dic2 x Lloyd:genotype_BX07.2.1f1.17.1 -0.2726364
## Year_2015:at(pop, Dic2 x Lloyd)_pop_Dic2 x Lloyd:genotype_BX07.2.1f1.17.1 -0.4703403
##                                                                                  genotype_year
## Year_2012:at(pop, Dic2 x Lloyd)_pop_Dic2 x Lloyd:genotype_BX07.2.1f1.17.1 BX07.2.1f1.17.1_2012
## Year_2015:at(pop, Dic2 x Lloyd)_pop_Dic2 x Lloyd:genotype_BX07.2.1f1.17.1 BX07.2.1f1.17.1_2015
##                                                                            real
## Year_2012:at(pop, Dic2 x Lloyd)_pop_Dic2 x Lloyd:genotype_BX07.2.1f1.17.1 FALSE
## Year_2015:at(pop, Dic2 x Lloyd)_pop_Dic2 x Lloyd:genotype_BX07.2.1f1.17.1  TRUE
```

```
## Get rid of genotypes with infered BLUPS (i.e. that were not observed that year)
BLUPElisa <- BLUPElisa[BLUPElisa$real==T,]

write.csv(BLUPElisa,file="BLUPsElisa2012_2015.csv",row.names=F,quote=F)
```

# 6 qPCR

## 6.1 Model selection

```
data2015 <- data[data$Year=="2015",]
## Spatially uncorrelated and correlated (AR1) environmental variance on row (X) and column (Y) , genotype and no linear X effect (model A for direct genetic effects and model 3 for residuals) (not converged)
## By default, only 13 iterations, so increase this number with maxiter
m0 <- asreml(QPCR ~ -1+pop2015mean+Year+pol(X,-1)+pol(Y,-1),rcov=~ar1(Y):ar1(X),random=~at(pop2015,"Dic2 x Lloyd"):genotype+at(pop2015,"Dic2 x Silur"):genotype+X_Y,na.method.X="include",data=data2015,maxiter=40,trace=F)
summary(m0)$varcomp
```

```
##                                                     gamma  component
## at(pop2015, Dic2 x Lloyd):genotype!genotype.var 6.2333342 14.5646023
## at(pop2015, Dic2 x Silur):genotype!genotype.var 6.4823248 15.1463856
## X_Y!X_Y.var                                     3.6759551  8.5891150
## R!variance                                      1.0000000  2.3365669
## R!Y.cor                                         0.8728997  0.8728997
## R!X.cor                                         0.6767655  0.6767655
##                                                 std.error  z.ratio
## at(pop2015, Dic2 x Lloyd):genotype!genotype.var 2.8311628 5.144389
## at(pop2015, Dic2 x Silur):genotype!genotype.var 3.0240048 5.008717
## X_Y!X_Y.var                                     1.8371196 4.675316
## R!variance                                      1.3489364 1.732155
## R!Y.cor                                         0.1417033 6.160052
## R!X.cor                                         0.3159802 2.141797
##                                                    constraint
## at(pop2015, Dic2 x Lloyd):genotype!genotype.var      Positive
## at(pop2015, Dic2 x Silur):genotype!genotype.var      Positive
## X_Y!X_Y.var                                          Positive
## R!variance                                           Positive
## R!Y.cor                                         Unconstrained
## R!X.cor                                         Unconstrained
```

```
## Distribution of breeding values for DL
hist(m0$coefficients$random[grepl("_pop2015_Dic2 x Lloyd",names(m0$coefficients$random))&m0$coefficient$random!=0],main="Dic2 x Lloyd",xlab=NA)
```

```
## Distribution of breeding values for DS
hist(m0$coefficients$random[grepl("_pop2015_Dic2 x Silur",names(m0$coefficients$random))&m0$coefficient$random!=0],main="Dic2 x Silur",xlab=NA)
```

```
##  No spatially correlated environmental variance (model 1 for residuals)
m1 <- asreml(QPCR ~ -1+pop2015mean+Year+pol(X,-1)+pol(Y,-1),rcov=~id(Y):id(X),random=~at(pop2015,"Dic2 x Lloyd"):genotype+at(pop2015,"Dic2 x Silur"):genotype,na.method.X="include",data=data2015,maxiter=40,trace=F)
summary(m1)$varcomp
```

```
##                                                    gamma component
## at(pop2015, Dic2 x Lloyd):genotype!genotype.var 1.563240 15.580573
## at(pop2015, Dic2 x Silur):genotype!genotype.var 1.552432 15.472848
## R!variance                                      1.000000  9.966845
##                                                 std.error  z.ratio
## at(pop2015, Dic2 x Lloyd):genotype!genotype.var  2.929692 5.318160
## at(pop2015, Dic2 x Silur):genotype!genotype.var  3.047544 5.077154
## R!variance                                       1.626497 6.127796
##                                                 constraint
## at(pop2015, Dic2 x Lloyd):genotype!genotype.var   Positive
## at(pop2015, Dic2 x Silur):genotype!genotype.var   Positive
## R!variance                                        Positive
```

```
## No spatially uncorrelated environmental variance (model 2 for residuals)
m2 <- asreml(QPCR ~ -1+pop2015mean+Year+pol(X,-1)+pol(Y,-1),rcov=~ar1(Y):ar1(X),random=~at(pop2015,"Dic2 x Lloyd"):genotype+at(pop2015,"Dic2 x Silur"):genotype,na.method.X="include",data=data2015,maxiter=40,trace=F)

##AR1 on X only
m3 <- asreml(QPCR ~ -1+pop2015mean+Year+pol(X,-1)+pol(Y,-1),rcov=~id(Y):ar1(X),random=~at(pop2015,"Dic2 x Lloyd"):genotype+at(pop2015,"Dic2 x Silur"):genotype+X_Y,na.method.X="include",data=data2015,maxiter=40,trace=F)
summary(m3)$varcomp
```

```
##                                                    gamma component
## at(pop2015, Dic2 x Lloyd):genotype!genotype.var 3.542809 15.395197
## at(pop2015, Dic2 x Silur):genotype!genotype.var 3.598173 15.635779
## X_Y!X_Y.var                                     1.308146  5.684519
## R!variance                                      1.000000  4.345478
## R!X.cor                                         0.516060  0.516060
##                                                 std.error   z.ratio
## at(pop2015, Dic2 x Lloyd):genotype!genotype.var 2.8885638 5.3297062
## at(pop2015, Dic2 x Silur):genotype!genotype.var 3.0242543 5.1701269
## X_Y!X_Y.var                                     5.2306402 1.0867730
## R!variance                                      5.1590822 0.8422966
## R!X.cor                                         0.4400748 1.1726643
##                                                    constraint
## at(pop2015, Dic2 x Lloyd):genotype!genotype.var      Positive
## at(pop2015, Dic2 x Silur):genotype!genotype.var      Positive
## X_Y!X_Y.var                                          Positive
## R!variance                                           Positive
## R!X.cor                                         Unconstrained
```

```
##AR1 on Y only
m4 <- asreml(QPCR ~ -1+pop2015mean+Year+pol(X,-1)+pol(Y,-1),rcov=~id(X):ar1(Y),random=~at(pop2015,"Dic2 x Lloyd"):genotype+at(pop2015,"Dic2 x Silur"):genotype+X_Y,na.method.X="include",data=data2[data2$Year=="2015",],maxiter=40,trace=F)
summary(m4)$varcomp
```

```
##                                                      gamma    component
## at(pop2015, Dic2 x Lloyd):genotype!genotype.var 1.51839147 1.535343e+01
## at(pop2015, Dic2 x Silur):genotype!genotype.var 1.51505354 1.531968e+01
## X_Y!X_Y.var                                     0.00000160 1.617863e-05
## R!variance                                      1.00000000 1.011164e+01
## R!Y.cor                                         0.08569433 8.569433e-02
##                                                    std.error   z.ratio
## at(pop2015, Dic2 x Lloyd):genotype!genotype.var 2.930087e+00 5.2399235
## at(pop2015, Dic2 x Silur):genotype!genotype.var 3.058034e+00 5.0096514
## X_Y!X_Y.var                                     2.655670e-06 6.0921086
## R!variance                                      1.659794e+00 6.0921086
## R!Y.cor                                         1.273244e-01 0.6730392
##                                                    constraint
## at(pop2015, Dic2 x Lloyd):genotype!genotype.var      Positive
## at(pop2015, Dic2 x Silur):genotype!genotype.var      Positive
## X_Y!X_Y.var                                          Boundary
## R!variance                                           Positive
## R!Y.cor                                         Unconstrained
```

```
AICcASRemlRmd(paste("m",0:4,sep=""))
```

```
##   modnames ParNum       LL      AIC     AICc DeltaAICc EvRatio weight
## 1       m1      9 -867.814 1753.628 1754.067     0.000   1.000  0.333
## 2       m0     12 -864.653 1753.306 1754.073     0.006   1.003  0.332
## 3       m3     11 -866.553 1755.106 1755.753     1.686   2.323  0.143
## 4       m2     11 -866.564 1755.128 1755.775     1.708   2.349  0.142
## 5       m4     11 -867.579 1757.158 1757.805     3.738   6.482  0.051
##                                               fixed
## 1 -1 + pop2015mean + Year + pol(X, -1) + pol(Y, -1)
## 2 -1 + pop2015mean + Year + pol(X, -1) + pol(Y, -1)
## 3 -1 + pop2015mean + Year + pol(X, -1) + pol(Y, -1)
## 4 -1 + pop2015mean + Year + pol(X, -1) + pol(Y, -1)
## 5 -1 + pop2015mean + Year + pol(X, -1) + pol(Y, -1)
##                                                                               Gside
## 1       at(pop2015, "Dic2 x Lloyd"):genotype + at(pop2015, "Dic2 x Silur"):genotype
## 2 at(pop2015, "Dic2 x Lloyd"):genotype + at(pop2015, "Dic2 x Silur"):genotype + X_Y
## 3 at(pop2015, "Dic2 x Lloyd"):genotype + at(pop2015, "Dic2 x Silur"):genotype + X_Y
## 4       at(pop2015, "Dic2 x Lloyd"):genotype + at(pop2015, "Dic2 x Silur"):genotype
## 5 at(pop2015, "Dic2 x Lloyd"):genotype + at(pop2015, "Dic2 x Silur"):genotype + X_Y
##            Rside
## 1   ~id(Y):id(X)
## 2 ~ar1(Y):ar1(X)
## 3  ~id(Y):ar1(X)
## 4 ~ar1(Y):ar1(X)
## 5  ~id(X):ar1(Y)
```

```
filename<-paste('AICcTable_ASReml-R_Analyses_', Sys.Date(),".csv",sep="")
file.rename(from=filename, to=paste('AICcTable_ASReml-R_Analyses_', Sys.Date(),"qPCR",".csv",sep=""))
```

```
## [1] TRUE
```

```
#plot.asrVariogram(variogram(m0))
hist(m0$residuals,main="Autocorrelated residual distribution")
```

```
hist(m0$coefficient$random[grep("X_Y",names(m0$coefficient$random))],main="Non-autocorrelated residual  distribution")
```

```
## Check spatial auto-correlation
y <- -100:100*0.1
plot(y,summary(m0)$varcomp[5,2]^abs(y))
```

```
x <- -10:10*2
plot(x,summary(m0)$varcomp[6,2]^abs(x))
```

## Fixed effects for qPCR

```
## Fixed effects
m1 <- asreml(QPCR ~ 1+pop2015mean+Year+pol(X,-1)+pol(Y,-1),rcov=~id(Y):id(X),random=~at(pop2015,"Dic2 x Lloyd"):genotype+at(pop2015,"Dic2 x Silur"):genotype,na.method.X="include",data=data2015,maxiter=40,trace=F)

m1$coefficients$fixed
```

```
##    pol(Y, -1)_order1    pol(X, -1)_order1            Year_2012 
##           -0.1152609           -0.3558944            0.0000000 
##            Year_2015     pop2015mean_DLDS pop2015mean_pescadou 
##            0.0000000            0.0000000            5.1611800 
##          (Intercept) 
##            9.3323272
```

```
##Conditional Wald F-test to test factors and their interaction
wald.asreml(m1, ssType="conditional", denDF="numeric")
```

```
## $Wald
##             Df denDF     F.inc     F.con Margin            Pr
## (Intercept)  1 373.1 1.384e+03 1.365e+03        9.827933e-127
## pop2015mean  1  83.8 1.983e+01 2.008e+01      A  2.332925e-05
## pol(X, -1)   1 329.9 5.446e-01 6.285e-01      A  4.284800e-01
## pol(Y, -1)   1 324.2 8.896e-02 8.896e-02      A  7.656988e-01
## 
## $stratumVariances
##                                           df  Variance
## at(pop2015, Dic2 x Lloyd):genotype 183.56584 27.678367
## at(pop2015, Dic2 x Silur):genotype 155.33439 28.182172
## R!variance                          75.09978  9.966845
##                                    at(pop2015, Dic2 x Lloyd):genotype
## at(pop2015, Dic2 x Lloyd):genotype                           1.130154
## at(pop2015, Dic2 x Silur):genotype                           0.000000
## R!variance                                                   0.000000
##                                    at(pop2015, Dic2 x Silur):genotype
## at(pop2015, Dic2 x Lloyd):genotype                        0.006661388
## at(pop2015, Dic2 x Silur):genotype                        1.177244683
## R!variance                                                0.000000000
##                                    R!variance
## at(pop2015, Dic2 x Lloyd):genotype          1
## at(pop2015, Dic2 x Silur):genotype          1
## R!variance                                  1
```

```
##Predictions for the interaction
pred <- predict(m1,classify="pop2015mean",sed=list("pop2015mean"=T))
```

```
## Predict terminating with 2 errors
## Error flags: 7 9
```

```
## Warning: Abnormal termination
## LogLikelihood not converged
## Results may be erroneous
```

```
pred$predictions
```

```
## $pvals
## 
## Notes:
## 
##   pop2015mean predicted.value standard.error est.status
## 1        DLDS               0              0         NA
## 2    pescadou               0              0         NA
## 
## $sed
##      [,1] [,2]
## [1,]    0    0
## [2,]    0    0
## 
## $avsed
##  min mean  max 
##    0    0    0
```

```
## Covariance between estimates
svc(m1)
```

```
##                                                        df variance
## at(pop2015, Dic2 x Lloyd):genotype!genotype.var 183.56563 27.67837
## at(pop2015, Dic2 x Silur):genotype!genotype.var 155.33443 28.18217
## R!variance                                       75.09978  9.96684
##                                                 at(pop2015, Dic2 x Lloyd):genotype!genotype.var
## at(pop2015, Dic2 x Lloyd):genotype!genotype.var                                         1.13015
## at(pop2015, Dic2 x Silur):genotype!genotype.var                                         0.00000
## R!variance                                                                              0.00000
##                                                 at(pop2015, Dic2 x Silur):genotype!genotype.var
## at(pop2015, Dic2 x Lloyd):genotype!genotype.var                                         0.00666
## at(pop2015, Dic2 x Silur):genotype!genotype.var                                         1.17724
## R!variance                                                                              0.00000
##                                                 R!variance
## at(pop2015, Dic2 x Lloyd):genotype!genotype.var          1
## at(pop2015, Dic2 x Silur):genotype!genotype.var          1
## R!variance                                               1
```

## 6.2 Model with lowest AICc

```
m1 <- asreml(QPCR ~ -1+pop2015mean,rcov=~id(Y):id(X),random=~at(pop2015,"Dic2 x Lloyd"):genotype+at(pop2015,"Dic2 x Silur"):genotype,na.method.X="include",data=data[data$Year=="2015",],maxiter=40,trace=F)
summary(m1)$varcomp
```

```
##                                                    gamma component
## at(pop2015, Dic2 x Lloyd):genotype!genotype.var 1.582219 15.587544
## at(pop2015, Dic2 x Silur):genotype!genotype.var 1.583907 15.604171
## R!variance                                      1.000000  9.851698
##                                                 std.error  z.ratio
## at(pop2015, Dic2 x Lloyd):genotype!genotype.var  2.909324 5.357789
## at(pop2015, Dic2 x Silur):genotype!genotype.var  3.033549 5.143867
## R!variance                                       1.600542 6.155226
##                                                 constraint
## at(pop2015, Dic2 x Lloyd):genotype!genotype.var   Positive
## at(pop2015, Dic2 x Silur):genotype!genotype.var   Positive
## R!variance                                        Positive
```

```
summary(m1)$varcomp[,2:3]
```

```
##                                                 component std.error
## at(pop2015, Dic2 x Lloyd):genotype!genotype.var 15.587544  2.909324
## at(pop2015, Dic2 x Silur):genotype!genotype.var 15.604171  3.033549
## R!variance                                       9.851698  1.600542
```

## 6.3 Compute heritabilities for qPCR

```
## Heritability Dic2 x Lloyd 2012
summary(m1)$varcomp[1,2]/sum(summary(m1)$varcomp[c(1,3),2])
```

```
## [1] 0.6127362
```

```
Heritability <- as.numeric(pin(m1,prop~(V1)/(V1+V3)))
CI<-c(Heritability[1]-qnorm(0.975)* Heritability[2], Heritability[1]+qnorm(0.975)* Heritability[2])
names(CI) <- c("Lower95%CI","Upper95%CI")
CI
```

```
## Lower95%CI Upper95%CI 
## -0.2527022  1.4781745
```

```
## Heritability Dic2 x Silur 2012
summary(m1)$varcomp[2,2]/sum(summary(m1)$varcomp[c(2,3),2])
```

```
## [1] 0.6129891
```

```
Heritability <- as.numeric(pin(m1,prop~(V2)/(V2+V3)))
CI<-c(Heritability[1]-qnorm(0.975)* Heritability[2], Heritability[1]+qnorm(0.975)* Heritability[2])
names(CI) <- c("Lower95%CI","Upper95%CI")
CI
```

```
## Lower95%CI Upper95%CI 
## -0.2509534  1.4769316
```

### 6.3.1 Spatial autocorrelation for qPCR (second best model)

```
## Spatial autocorrelation Dic2 x Lloyd 2012
summary(m0)$varcomp[4,2]/sum(summary(m0)$varcomp[c(1,3,4),2])
```

```
## [1] 0.091665
```

```
## Spatial autocorrelation Dic2 x Silur 2012
summary(m0)$varcomp[4,2]/sum(summary(m0)$varcomp[c(2,3,4),2])
```

```
## [1] 0.08961955
```

## 6.4 Extract BLUPs

```
hist(m1$coefficient$random[grepl("genotype",names(m1$coefficient$random))&m1$coefficient$random!=0],main="BLUP distribution")
```

```
## 344 genotypes in total
length(unique(data$genotype[data$cross2015=="cross"&!is.na(data$cross2015)&!is.na(data$QPCR)]))
```

```
## [1] 344
```

```
## 278 genotypes without replication
tab <- table(data$genotype[data$cross2015=="cross"&!is.na(data$cross)&!is.na(data$QPCR)])
length(tab[tab==1])
```

```
## [1] 278
```

```
## Extract BLUPS
coefqPCR <- m1$coefficient$random[grepl("genotype",names(m1$coefficient$random))&m1$coefficient$random!=0]
## Extract genotype, pop, year
coefqPCRname <- strsplit(names(coefqPCR),":")
coefqPCRname <- lapply(coefqPCRname,function(x){strsplit(x,"_")})

pop = do.call("rbind", lapply(coefqPCRname, "[[", 1))
geno = do.call("rbind", lapply(coefqPCRname, "[[", 2))

BLUPqPCR <- data.frame(pop[,3],geno[,2],coefqPCR)

names(BLUPqPCR) <- c("pop","geno","BLUP")
head(BLUPqPCR)
```

```
##                                                                                  pop
## at(pop2015, Dic2 x Lloyd)_pop2015_Dic2 x Lloyd:genotype_BX07.2.1f1.1.1  Dic2 x Lloyd
## at(pop2015, Dic2 x Lloyd)_pop2015_Dic2 x Lloyd:genotype_BX07.2.1f1.10.1 Dic2 x Lloyd
## at(pop2015, Dic2 x Lloyd)_pop2015_Dic2 x Lloyd:genotype_BX07.2.1f1.10.2 Dic2 x Lloyd
## at(pop2015, Dic2 x Lloyd)_pop2015_Dic2 x Lloyd:genotype_BX07.2.1f1.11.1 Dic2 x Lloyd
## at(pop2015, Dic2 x Lloyd)_pop2015_Dic2 x Lloyd:genotype_BX07.2.1f1.11.2 Dic2 x Lloyd
## at(pop2015, Dic2 x Lloyd)_pop2015_Dic2 x Lloyd:genotype_BX07.2.1f1.12.1 Dic2 x Lloyd
##                                                                                    geno
## at(pop2015, Dic2 x Lloyd)_pop2015_Dic2 x Lloyd:genotype_BX07.2.1f1.1.1   BX07.2.1f1.1.1
## at(pop2015, Dic2 x Lloyd)_pop2015_Dic2 x Lloyd:genotype_BX07.2.1f1.10.1 BX07.2.1f1.10.1
## at(pop2015, Dic2 x Lloyd)_pop2015_Dic2 x Lloyd:genotype_BX07.2.1f1.10.2 BX07.2.1f1.10.2
## at(pop2015, Dic2 x Lloyd)_pop2015_Dic2 x Lloyd:genotype_BX07.2.1f1.11.1 BX07.2.1f1.11.1
## at(pop2015, Dic2 x Lloyd)_pop2015_Dic2 x Lloyd:genotype_BX07.2.1f1.11.2 BX07.2.1f1.11.2
## at(pop2015, Dic2 x Lloyd)_pop2015_Dic2 x Lloyd:genotype_BX07.2.1f1.12.1 BX07.2.1f1.12.1
##                                                                              BLUP
## at(pop2015, Dic2 x Lloyd)_pop2015_Dic2 x Lloyd:genotype_BX07.2.1f1.1.1  -5.560290
## at(pop2015, Dic2 x Lloyd)_pop2015_Dic2 x Lloyd:genotype_BX07.2.1f1.10.1 -3.717269
## at(pop2015, Dic2 x Lloyd)_pop2015_Dic2 x Lloyd:genotype_BX07.2.1f1.10.2 -5.564058
## at(pop2015, Dic2 x Lloyd)_pop2015_Dic2 x Lloyd:genotype_BX07.2.1f1.11.1  1.968214
## at(pop2015, Dic2 x Lloyd)_pop2015_Dic2 x Lloyd:genotype_BX07.2.1f1.11.2  1.694360
## at(pop2015, Dic2 x Lloyd)_pop2015_Dic2 x Lloyd:genotype_BX07.2.1f1.12.1 -6.065238
```

```
## 344 genotypes
BLUPqPCR$year <- as.factor(rep("2015",nrow(BLUPqPCR)))
nrow(BLUPqPCR[BLUPqPCR$year=="2015",])
```

```
## [1] 344
```

```
BLUPqPCR$genotype_year <- paste(BLUPqPCR$geno,BLUPqPCR$year,sep="_")

BLUPqPCR$real <- BLUPqPCR$genotype_year%in%data$genotype_year

## Get rid of genotypes with infered BLUPS (i.e. that were not observed that year)
BLUPqPCR <- BLUPqPCR[BLUPqPCR$real==T,]
write.csv(BLUPqPCR,file="BLUPsqPCR2015.csv",row.names=F,quote=F)
```
