# Supplementary material for: Epistatic determinism of durum wheat resistance to the wheat spindle streak mosaic virus
Source: Theor Appl Genet. 2017 Apr 27;130(7):1491–505. doi: 10.1007/s00122-017-2904-6 (PMC5487696; doi:10.1007/s00122-017-2904-6)
Supplement: Supplementary file 4 — Online Resource 4: Data and R scripts for reproducible QTL detection. Data and R script (.csv and.rmd format) are provided in this tar archive. A scheme aims to explain the content of each file and its role in the QTL detection pipeline. The upstream bioinformatic steps (from raw reads to consensus genetic map) are not included (GZ 72829 kb) [file 122_2017_2904_MOESM4_ESM.gz › TMP/SCRIPT/0_Preparing_data/PrepDataWheatResistance2012-2015.html]

WheatResistance


# WheatResistance

#### *Nicolas O. Rode*

#### *24 June 2016*

# 1 Data 2012

## 1.1 Set working directory and load dataset

```
setwd("/homedir/rode/Rstudio/Yan/2012-2015/24062016")
data <- read.table("~/Rstudio/Yan/2012/bilan_WSSMV.csv" , header=T , sep=";" , na.strings="NA")

## Replace the name of the dic2 genotypes
data$Num <- as.character(data$Num)
data$Num[data$Genealogie=="dic2"&!is.na(data$Genealogie)] <- as.character(data$N_preparation_semis_Melgueil_2010_2011[data$Genealogie=="dic2"&!is.na(data$Genealogie)])

# Je garde seulement les colonnes utilise :
data=data[ , c(1,5,6,19,21,26,27)]
head(data)
```

```
##   genotype Genealogie  Num note_18_04 DO_pondere_positif  X  Y
## 1     <NA>       <NA> <NA>         NA                 NA  2 73
## 2     <NA>       <NA> <NA>         NA                 NA 10 66
## 3     <NA>       <NA> <NA>         NA                 NA 10 67
## 4     <NA>       <NA> <NA>         NA                 NA 10 68
## 5     <NA>       <NA> <NA>         NA                 NA  6 73
## 6     <NA>       <NA> <NA>         NA                 NA  6 74
```

## 1.2 Data preparation

```
#Ajout des colonnes des voisins ? --> 9 colonnes à ajouter !
Neighbour <- data.frame(matrix(c("neigh1","neigh2","neigh3","neigh8","focal genotype","neigh4","neigh7","neigh6","neigh5"),byrow=F,ncol=3))
colnames(Neighbour) <- c("Y1","Y2","Y3")
rownames(Neighbour) <- c("X1","X2","X3")
Neighbour
```

```
##        Y1             Y2     Y3
## X1 neigh1         neigh8 neigh7
## X2 neigh2 focal genotype neigh6
## X3 neigh3         neigh4 neigh5
```

```
voisin=as.data.frame(matrix(0 , ncol=8 ,  nrow=750))
for(i in 1:nrow(data)){
    #print(i)
    ind=as.character(data$Num[data$X==data$X[i]-1 & data$Y==data$Y[i]-1]) ; if(length(ind)==0) { voisin[i,1]= "side1" } else { voisin[i,1]=ind }
    ind=as.character(data$Num[data$X==data$X[i]+0 & data$Y==data$Y[i]-1]) ; if(length(ind)==0) { voisin[i,2]= "side2" } else { voisin[i,2]=ind }
    ind=as.character(data$Num[data$X==data$X[i]+1 & data$Y==data$Y[i]-1]) ; if(length(ind)==0) { voisin[i,3]= "side3" } else { voisin[i,3]=ind }
    ind=as.character(data$Num[data$X==data$X[i]+1 & data$Y==data$Y[i]+0]) ; if(length(ind)==0) { voisin[i,4]= "side4" } else { voisin[i,4]=ind }
    ind=as.character(data$Num[data$X==data$X[i]+1 & data$Y==data$Y[i]+1]) ; if(length(ind)==0) { voisin[i,5]= "side5" } else { voisin[i,5]=ind }
    ind=as.character(data$Num[data$X==data$X[i]+0 & data$Y==data$Y[i]+1]) ; if(length(ind)==0) { voisin[i,6]= "side6" } else { voisin[i,6]=ind }
    ind=as.character(data$Num[data$X==data$X[i]-1 & data$Y==data$Y[i]+1]) ; if(length(ind)==0) { voisin[i,7]= "side7" } else { voisin[i,7]=ind }
    ind=as.character(data$Num[data$X==data$X[i]-1 & data$Y==data$Y[i]+0]) ; if(length(ind)==0) { voisin[i,8]= "side8" } else { voisin[i,8]=ind }
}
head(voisin)
```

```
##                V1              V2              V3    V4       V5       V6
## 1    TT06DC 44.42 BX07.2.1f1.21.1 BX07.2.1f1.12.2  <NA>     <NA>     <NA>
## 2        pescadou        pescadou           side3 side4    side5     <NA>
## 3 BX07.2.1f1.25.2            <NA>           side3 side4    side5     <NA>
## 4    TT06DC 38.29            <NA>           side3 side4    side5     <NA>
## 5    TT06DC 42.24   BX07.3.2f1.37    TT06DC 44.10  <NA>     <NA>     <NA>
## 6            <NA>            <NA>            <NA>  <NA> pescadou pescadou
##              V7              V8
## 1          <NA>            <NA>
## 2  TT06DC 38.29 BX07.2.1f1.25.2
## 3 BX07.2.1f2.43    TT06DC 38.29
## 4 BX07.2.1f2.61   BX07.2.1f2.43
## 5          <NA>            <NA>
## 6      pescadou            <NA>
```

```
data=cbind(data,voisin)
colnames(data)=c("plante","pop","genotype","NV","Elisa","X","Y","neigh1","neigh2","neigh3","neigh4","neigh5","neigh6","neigh7","neigh8")

data$neigh1<-factor(data$neigh1)
data$neigh2<-factor(data$neigh2)
data$neigh3<-factor(data$neigh3)
data$neigh4<-factor(data$neigh4)
data$neigh5<-factor(data$neigh5)
data$neigh6<-factor(data$neigh6)
data$neigh7<-factor(data$neigh7)
data$neigh8<-factor(data$neigh8)


head(data)
```

```
##   plante  pop genotype NV Elisa  X  Y          neigh1          neigh2
## 1   <NA> <NA>     <NA> NA    NA  2 73    TT06DC 44.42 BX07.2.1f1.21.1
## 2   <NA> <NA>     <NA> NA    NA 10 66        pescadou        pescadou
## 3   <NA> <NA>     <NA> NA    NA 10 67 BX07.2.1f1.25.2            <NA>
## 4   <NA> <NA>     <NA> NA    NA 10 68    TT06DC 38.29            <NA>
## 5   <NA> <NA>     <NA> NA    NA  6 73    TT06DC 42.24   BX07.3.2f1.37
## 6   <NA> <NA>     <NA> NA    NA  6 74            <NA>            <NA>
##            neigh3 neigh4   neigh5   neigh6        neigh7          neigh8
## 1 BX07.2.1f1.12.2   <NA>     <NA>     <NA>          <NA>            <NA>
## 2           side3  side4    side5     <NA>  TT06DC 38.29 BX07.2.1f1.25.2
## 3           side3  side4    side5     <NA> BX07.2.1f2.43    TT06DC 38.29
## 4           side3  side4    side5     <NA> BX07.2.1f2.61   BX07.2.1f2.43
## 5    TT06DC 44.10   <NA>     <NA>     <NA>          <NA>            <NA>
## 6            <NA>   <NA> pescadou pescadou      pescadou            <NA>
```

```
tail(data)
```

```
##     plante      pop genotype  NV     Elisa X  Y         neigh1
## 745   pp94 pescadou pescadou 3.0 0.9053404 7 20  BX07.3.2f1.86
## 746   pp95 pescadou pescadou 2.5 0.3991395 7 25  BX07.3.2f1.31
## 747   pp96 pescadou pescadou 2.0 0.9951175 7 30    TT06DC 40.1
## 748   pp97 pescadou pescadou 2.5 0.1333537 7 35   BX07.2.1f2.2
## 749   pp98 pescadou pescadou 2.5 1.8302439 7 40  BX07.3.2f1.44
## 750   pp99 pescadou pescadou 3.0 0.9398601 7 45 BX07.2.1f1.5.2
##            neigh2          neigh3   neigh4        neigh5        neigh6
## 745         lloyd            dicF pescadou BX07.3.2f1.42 BX07.3.2f2.43
## 746   TT06DC 44.7     TT06DC 40.8 pescadou  TT06DC 42.53  TT06DC 42.49
## 747 BX07.3.2f1.41    TT06DC 44.43 pescadou BX07.3.2f2.10  TT06DC 44.50
## 748 BX07.3.2f1.73   BX07.2.1f2.77 pescadou   TT06DC 42.5 BX07.3.2f2.22
## 749   TT06DC 42.4 BX07.2.1f1.30.2 pescadou  TT06DC 40.24 BX07.3.2f2.80
## 750 BX07.3.2f1.50   BX07.3.2f1.18 pescadou  TT06DC 44.19   TT06DC 38.7
##              neigh7   neigh8
## 745    BX07.2.1f2.1 pescadou
## 746    TT06DC 39.16 pescadou
## 747     TT06DC 42.4 pescadou
## 748    TT06DC 44.22 pescadou
## 749 BX07.2.1f1.33.2 pescadou
## 750    TT06DC 42.49 pescadou
```

```
data$X_Y <- as.factor(paste(data$X,data$Y,sep="_"))

## 750 rows
nrow(data)
```

```
## [1] 750
```

```
## Get rid of one genotype that is not replicated in 2012/2015
data$NV[data$genotype=="dic74031"&!is.na(data$genotype)] <- NA
data$Elisa[data$genotype=="dic74031"&!is.na(data$genotype)] <- NA


## Change name for Dic2
data$genotype[data$genotype=="dic2"&!is.na(data$pop)] <- rep("Dic2",length(data$genotype[data$genotype=="dic2"&!is.na(data$pop)]))
data$genotype[data$genotype=="lloyd"&!is.na(data$pop)] <- rep("Lloyd",length(data$genotype[data$genotype=="lloyd"&!is.na(data$pop)]))

popnew <- as.character(data$genotype)
popnew[data$pop=="Dic 2 x Lloyd"&!is.na(data$pop)] <- rep("Dic2 x Lloyd",length(popnew[data$pop=="Dic 2 x Lloyd"&!is.na(data$pop)]))
popnew[data$pop=="Dic 2 x Silur"&!is.na(data$pop)] <- rep("Dic2 x Silur",length(popnew[data$pop=="Dic 2 x Silur"&!is.na(data$pop)]))
popnew[data$pop=="Dic 2 x Soldur"&!is.na(data$pop)] <- rep("Dic2 x Soldur",length(popnew[data$pop=="Dic 2 x Soldur"&!is.na(data$pop)]))

table(popnew)
```

```
## popnew
##          Dic2  Dic2 x Lloyd  Dic2 x Silur Dic2 x Soldur          dicA 
##             1           187           246           100             2 
##          dicB          dicC          dicD          dicE          dicF 
##             2             2             1             2             2 
##          dicG          dicH          dicI          dicJ         Lloyd 
##             2             2             2             2             5 
##      pescadou         Silur        Soldur 
##           149             4            10
```

```
data$pop <- as.factor(popnew)

## Get rid of genotype with no replicate in 2012 or 2015
data$Elisa[data$pop=="dicD"&!is.na(data$pop)] <- rep(NA,length(data$pop[data$pop=="dicD"&!is.na(data$pop)]))
data$NV[data$pop=="dicD"&!is.na(data$pop)] <- rep(NA,length(data$pop[data$pop=="dicD"&!is.na(data$pop)]))
data$genotype[data$genotype=="dicD"&!is.na(data$genotype)] <- NA
data$pop[data$pop=="dicD"&!is.na(data$pop)] <- NA

## 54 rows with missing data
nrow(data[is.na(data$NV)|is.na(data$Elisa)|is.na(data$genotype),])
```

```
## [1] 54
```

```
## Positive correlation between the two descriptors of the disease
cor.test(data$NV,data$Elisa)
```

```
## 
##  Pearson's product-moment correlation
## 
## data:  data$NV and data$Elisa
## t = 16.295, df = 694, p-value < 2.2e-16
## alternative hypothesis: true correlation is not equal to 0
## 95 percent confidence interval:
##  0.4701151 0.5777800
## sample estimates:
##      cor 
## 0.526052
```

```
## NA for missing/unknown genotypes in genotype and visual scores
data$NV <- ifelse(is.na(data$genotype),NA,data$NV)
data$Elisa<- ifelse(is.na(data$genotype),NA,data$Elisa)

## 30 missing genotypes
sum(is.na(data$genotype))
```

```
## [1] 30
```

```
## 35 missing visual scores
sum(is.na(data$NV))
```

```
## [1] 35
```

```
## 52 missing visual scores
sum(is.na(data$Elisa))
```

```
## [1] 52
```

```
data <- data[order(data$Y,data$X),]

data2012<-data

write.csv(data2012,file="Data2012.csv",row.names=F,quote=F)
```

# 2 Data 2015

## 2.1 Load the data

```
getwd()
```

```
## [1] "/homedir/rode/Rstudio/Yan/2012-2015/24062016"
```

```
data <- read.table("~/Rstudio/Yan/2015/Info_Pray_2015_lignes_dedoublees.csv" , header=T , sep=";" , na.strings="NA")

head(data)
```

```
##   Position_Arvalis Code_INRA cadrillage genealogie num_prepa_Gerard
## 1            NA-NA      <NA>        a-1       <NA>             <NA>
## 2            NA-NA      <NA>        a-2       <NA>             <NA>
## 3            NA-NA      <NA>        a-3       <NA>             <NA>
## 4            NA-NA      <NA>        a-4       <NA>             <NA>
## 5            NA-NA      <NA>        a-5       <NA>             <NA>
## 6            NA-NA      <NA>        a-6       <NA>             <NA>
##   croisement  Rep Lettre code_semis lieu annee num_plaque num_traitement
## 1       <NA> <NA>   <NA>       <NA> <NA>  <NA>         NA           <NA>
## 2       <NA> <NA>   <NA>       <NA> <NA>  <NA>         NA           <NA>
## 3       <NA> <NA>   <NA>       <NA> <NA>  <NA>         NA           <NA>
## 4       <NA> <NA>   <NA>       <NA> <NA>  <NA>         NA           <NA>
## 5       <NA> <NA>   <NA>       <NA> <NA>  <NA>         NA           <NA>
## 6       <NA> <NA>   <NA>       <NA> <NA>  <NA>         NA           <NA>
##   note_27_03_2015 n_plaque n_traitement note2 plaque lecture virus
## 1              NA       NA         <NA>    NA   <NA>    <NA>  <NA>
## 2              NA       NA         <NA>    NA   <NA>    <NA>  <NA>
## 3              NA       NA         <NA>    NA   <NA>    <NA>  <NA>
## 4              NA       NA         <NA>    NA   <NA>    <NA>  <NA>
## 5              NA       NA         <NA>    NA   <NA>    <NA>  <NA>
## 6              NA       NA         <NA>    NA   <NA>    <NA>  <NA>
##   anticorps blanc negatif positif moyenne coef_var moy_pondere_negatif
## 1      <NA>    NA      NA      NA      NA       NA                  NA
## 2      <NA>    NA      NA      NA      NA       NA                  NA
## 3      <NA>    NA      NA      NA      NA       NA                  NA
## 4      <NA>    NA      NA      NA      NA       NA                  NA
## 5      <NA>    NA      NA      NA      NA       NA                  NA
## 6      <NA>    NA      NA      NA      NA       NA                  NA
##   moy_pondere_positif Ct QuantityQPCR Tm1_t Tm2_t Tm3_t
## 1                  NA NA           NA    NA    NA    NA
## 2                  NA NA           NA    NA    NA    NA
## 3                  NA NA           NA    NA    NA    NA
## 4                  NA NA           NA    NA    NA    NA
## 5                  NA NA           NA    NA    NA    NA
## 6                  NA NA           NA    NA    NA    NA
```

```
# Ajout de colonne de position : A-10 --> X=1 , Y=10
data$cadrillage=as.character(data$cadrillage)
#Fabrication de 2 petites fonctions qui récupèrent les coordon?es x et y de la colonne de cadrillage
fun_x=function(x){strsplit(x,"-")[[1]][1]}
fun_y=function(x){strsplit(x,"-")[[1]][2] }
# Récupèration des informations concernant le cadrillage. (Je transforme le format A,B,C... en 1,2,3... + je retourne le vecteur pour que le 1 soit en haut (par d?fault la fonction levelplot me met le A en bas...)) :

#! order revrsed compared to 2012
data$Y=as.character(lapply(data$cadrillage , fun_x))
data$Y=match(data$Y, sort(unique(data$Y)))
data$X=as.numeric(lapply(data$cadrillage , fun_y))

# Je garde seulement les colonnes utilise :
data=data[,c(1,6,4,17,28,30,34,35)]
head(data)
```

```
##   Position_Arvalis croisement genealogie note2 moy_pondere_positif
## 1            NA-NA       <NA>       <NA>    NA                  NA
## 2            NA-NA       <NA>       <NA>    NA                  NA
## 3            NA-NA       <NA>       <NA>    NA                  NA
## 4            NA-NA       <NA>       <NA>    NA                  NA
## 5            NA-NA       <NA>       <NA>    NA                  NA
## 6            NA-NA       <NA>       <NA>    NA                  NA
##   QuantityQPCR Y X
## 1           NA 1 1
## 2           NA 1 2
## 3           NA 1 3
## 4           NA 1 4
## 5           NA 1 5
## 6           NA 1 6
```

## 2.2 Data preparation

```
#Ajout des colonnes des voisins ? --> 9 colonnes ? ajouter !
voisin=as.data.frame(matrix(0 , ncol=8 ,  nrow=750))
for(i in 1:nrow(data)){
    #print(i)
    ind=as.character(data$genealogie[data$X==data$X[i]-1 & data$Y==data$Y[i]-1]) ; if(length(ind)==0) { voisin[i,1]= "side1" } else { voisin[i,1]=ind }
    ind=as.character(data$genealogie[data$X==data$X[i]+0 & data$Y==data$Y[i]-1]) ; if(length(ind)==0) { voisin[i,2]= "side2" } else { voisin[i,2]=ind }
    ind=as.character(data$genealogie[data$X==data$X[i]+1 & data$Y==data$Y[i]-1]) ; if(length(ind)==0) { voisin[i,3]= "side3" } else { voisin[i,3]=ind }
    ind=as.character(data$genealogie[data$X==data$X[i]+1 & data$Y==data$Y[i]+0]) ; if(length(ind)==0) { voisin[i,4]= "side4" } else { voisin[i,4]=ind }
    ind=as.character(data$genealogie[data$X==data$X[i]+1 & data$Y==data$Y[i]+1]) ; if(length(ind)==0) { voisin[i,5]= "side5" } else { voisin[i,5]=ind }
    ind=as.character(data$genealogie[data$X==data$X[i]+0 & data$Y==data$Y[i]+1]) ; if(length(ind)==0) { voisin[i,6]= "side6" } else { voisin[i,6]=ind }
    ind=as.character(data$genealogie[data$X==data$X[i]-1 & data$Y==data$Y[i]+1]) ; if(length(ind)==0) { voisin[i,7]= "side7" } else { voisin[i,7]=ind }
    ind=as.character(data$genealogie[data$X==data$X[i]-1 & data$Y==data$Y[i]+0]) ; if(length(ind)==0) { voisin[i,8]= "side8" } else { voisin[i,8]=ind }
}

head(voisin)
```

```
##      V1    V2    V3   V4   V5   V6    V7    V8
## 1 side1 side2 side3 <NA> <NA> <NA> side7 side8
## 2 side1 side2 side3 <NA> <NA> <NA>  <NA>  <NA>
## 3 side1 side2 side3 <NA> <NA> <NA>  <NA>  <NA>
## 4 side1 side2 side3 <NA> <NA> <NA>  <NA>  <NA>
## 5 side1 side2 side3 <NA> <NA> <NA>  <NA>  <NA>
## 6 side1 side2 side3 <NA> <NA> <NA>  <NA>  <NA>
```

```
data=cbind(data,voisin)
colnames(data)=c("plante","pop","genotype","NV","Elisa","QPCR","X","Y","neigh1","neigh2","neigh3","neigh4","neigh5","neigh6","neigh7","neigh8")

data$neigh1<-factor(data$neigh1)
data$neigh2<-factor(data$neigh2)
data$neigh3<-factor(data$neigh3)
data$neigh4<-factor(data$neigh4)
data$neigh5<-factor(data$neigh5)
data$neigh6<-factor(data$neigh6)
data$neigh7<-factor(data$neigh7)
data$neigh8<-factor(data$neigh8)


levels(data$pop) <- c("Dic2 x Lloyd", "Dic2" , "Dic2 x Soldur", "Dic2 x Silur", "Lloyd", "pescadou","Silur","Soldur")

head(data)
```

```
##   plante  pop genotype NV Elisa QPCR X Y neigh1 neigh2 neigh3 neigh4
## 1  NA-NA <NA>     <NA> NA    NA   NA 1 1  side1  side2  side3   <NA>
## 2  NA-NA <NA>     <NA> NA    NA   NA 1 2  side1  side2  side3   <NA>
## 3  NA-NA <NA>     <NA> NA    NA   NA 1 3  side1  side2  side3   <NA>
## 4  NA-NA <NA>     <NA> NA    NA   NA 1 4  side1  side2  side3   <NA>
## 5  NA-NA <NA>     <NA> NA    NA   NA 1 5  side1  side2  side3   <NA>
## 6  NA-NA <NA>     <NA> NA    NA   NA 1 6  side1  side2  side3   <NA>
##   neigh5 neigh6 neigh7 neigh8
## 1   <NA>   <NA>  side7  side8
## 2   <NA>   <NA>   <NA>   <NA>
## 3   <NA>   <NA>   <NA>   <NA>
## 4   <NA>   <NA>   <NA>   <NA>
## 5   <NA>   <NA>   <NA>   <NA>
## 6   <NA>   <NA>   <NA>   <NA>
```

```
tail(data)
```

```
##     plante  pop genotype NV Elisa QPCR  X  Y neigh1 neigh2 neigh3 neigh4
## 774  NA-NA <NA>     <NA> NA    NA   NA 19 36   <NA>   <NA>   <NA>   <NA>
## 775  NA-NA <NA>     <NA> NA    NA   NA 19 37   <NA>   <NA>   <NA>   <NA>
## 776  NA-NA <NA>     <NA> NA    NA   NA 19 38   <NA>   <NA>   <NA>   <NA>
## 777  NA-NA <NA>     <NA> NA    NA   NA 19 39   <NA>   <NA>   <NA>   <NA>
## 778  NA-NA <NA>     <NA> NA    NA   NA 19 40   <NA>   <NA>   <NA>   <NA>
## 779  NA-NA <NA>     <NA> NA    NA   NA 19 41   <NA>   <NA>  side3  side4
##     neigh5 neigh6 neigh7 neigh8
## 774  side5  side6  side7   <NA>
## 775  side5  side6  side7   <NA>
## 776  side5  side6  side7   <NA>
## 777  side5  side6  side7   <NA>
## 778  side5  side6  side7   <NA>
## 779  side5  side6  side7   <NA>
```

```
hist(data$NV)
```

```
hist(data$Elisa)
```

```
hist(log(data$QPCR))
```

```
plot(log(data$QPCR)~data$Elisa)
```

```
cor.test(log(data$QPCR),data$Elisa)
```

```
## 
##  Pearson's product-moment correlation
## 
## data:  log(data$QPCR) and data$Elisa
## t = 22.317, df = 453, p-value < 2.2e-16
## alternative hypothesis: true correlation is not equal to 0
## 95 percent confidence interval:
##  0.6767519 0.7647156
## sample estimates:
##       cor 
## 0.7236596
```

```
data$X_Y <- as.factor(paste(data$X,data$Y,sep="_"))
data$QPCR<-log(data$QPCR)

## 779 rows
nrow(data)
```

```
## [1] 779
```

```
## 194 rows with missing data
nrow(data[is.na(data$NV)|is.na(data$Elisa)|is.na(data$genotype),])
```

```
## [1] 194
```

```
## Positive correlation between the two descriptors of the disease
cor.test(data$NV,data$Elisa)
```

```
## 
##  Pearson's product-moment correlation
## 
## data:  data$NV and data$Elisa
## t = 20.995, df = 583, p-value < 2.2e-16
## alternative hypothesis: true correlation is not equal to 0
## 95 percent confidence interval:
##  0.6074085 0.6999950
## sample estimates:
##       cor 
## 0.6561641
```

```
## NA for missing/unknown genotypes in genotype and visual scores
data$NV <- ifelse(is.na(data$genotype),NA,data$NV)
data$Elisa<- ifelse(is.na(data$genotype),NA,data$Elisa)

## 179 missing genotypes
sum(is.na(data$genotype))
```

```
## [1] 179
```

```
## 185 missing visual scores
sum(is.na(data$NV))
```

```
## [1] 185
```

```
## 189 missing visual scores
sum(is.na(data$Elisa))
```

```
## [1] 189
```

```
## 324 missing qPCR values
sum(is.na(data$QPCR))
```

```
## [1] 324
```

```
data <- data[order(data$Y,data$X),]

data2015<-data
write.csv(data2015,file="Data2015.csv",row.names=F,quote=F)
```

# 3 Data 2012-2015

```
QPCR<-rep(NA,nrow(data2012))

data2012<-data.frame(data2012[,1:5],QPCR,data2012[,6:16])
data2012$Year<-rep("2012",nrow(data2012))

data2015<-data2015[,1:17]
data2015$Year<-rep("2015",nrow(data2015))

data.frame(names(data2012),names(data2015))
```

```
##    names.data2012. names.data2015.
## 1           plante          plante
## 2              pop             pop
## 3         genotype        genotype
## 4               NV              NV
## 5            Elisa           Elisa
## 6             QPCR            QPCR
## 7                X               X
## 8                Y               Y
## 9           neigh1          neigh1
## 10          neigh2          neigh2
## 11          neigh3          neigh3
## 12          neigh4          neigh4
## 13          neigh5          neigh5
## 14          neigh6          neigh6
## 15          neigh7          neigh7
## 16          neigh8          neigh8
## 17             X_Y             X_Y
## 18            Year            Year
```

```
data<-data.frame(rbind(data2012,data2015))
nrow(data)
```

```
## [1] 1529
```

```
head(data)
```

```
##     plante          pop     genotype NV     Elisa QPCR X Y neigh1 neigh2
## 27      p1       Soldur       Soldur  2 0.9502664   NA 1 1  side1  side2
## 70    p144 Dic2 x Silur TT06DC 44.28  3 0.7173187   NA 2 1  side1  side2
## 71    p145        Silur        Silur  4 1.2568765   NA 3 1  side1  side2
## 199   p284 Dic2 x Silur TT06DC 44.39  0 1.0147956   NA 4 1  side1  side2
## 200   p285 Dic2 x Silur TT06DC 38.27  3 1.0133161   NA 5 1  side1  side2
## 330   p429 Dic2 x Silur TT06DC 42.15  3 0.2261215   NA 6 1  side1  side2
##     neigh3       neigh4        neigh5        neigh6        neigh7
## 27   side3 TT06DC 44.28         lloyd  TT06DC 39.35         side7
## 70   side3        Silur   TT06DC 40.4         lloyd  TT06DC 39.35
## 71   side3 TT06DC 44.39 BX07.2.1f2.71   TT06DC 40.4         lloyd
## 199  side3 TT06DC 38.27  TT06DC 38.12 BX07.2.1f2.71   TT06DC 40.4
## 200  side3 TT06DC 42.15 BX07.2.1f2.53  TT06DC 38.12 BX07.2.1f2.71
## 330  side3 BX07.3.2f1.7 BX07.2.1f2.91 BX07.2.1f2.53  TT06DC 38.12
##           neigh8 X_Y Year
## 27         side8 1_1 2012
## 70        Soldur 2_1 2012
## 71  TT06DC 44.28 3_1 2012
## 199        Silur 4_1 2012
## 200 TT06DC 44.39 5_1 2012
## 330 TT06DC 38.27 6_1 2012
```

```
levels(data$pop)
```

```
##  [1] "Dic2"          "Dic2 x Lloyd"  "Dic2 x Silur"  "Dic2 x Soldur"
##  [5] "dicA"          "dicB"          "dicC"          "dicD"         
##  [9] "dicE"          "dicF"          "dicG"          "dicH"         
## [13] "dicI"          "dicJ"          "Lloyd"         "pescadou"     
## [17] "Silur"         "Soldur"
```

```
data$genotype[data$pop=="Dic2"&!is.na(data$pop)]
```

```
## [1] "Dic2" "Dic2" "Dic2"
```

```
data$genotype<-factor(data$genotype)

tapply(data$Year,list(data$Year,data$genotype),length)
```

```
##      BX07.2.1f1.1.1 BX07.2.1f1.1.2 BX07.2.1f1.10.1 BX07.2.1f1.10.2
## 2012              1              1               1               1
## 2015              1              1               1               1
##      BX07.2.1f1.11.1 BX07.2.1f1.11.2 BX07.2.1f1.12.1 BX07.2.1f1.12.2
## 2012               1               1               1               1
## 2015               1               2               1               2
##      BX07.2.1f1.13.1 BX07.2.1f1.13.2 BX07.2.1f1.14.1 BX07.2.1f1.14.2
## 2012               1               1               1               1
## 2015               2               1               1               1
##      BX07.2.1f1.15.1 BX07.2.1f1.15.2 BX07.2.1f1.16.1 BX07.2.1f1.16.2
## 2012               1               1               1               1
## 2015               2               1              NA               2
##      BX07.2.1f1.17.1 BX07.2.1f1.17.2 BX07.2.1f1.18.1 BX07.2.1f1.18.2
## 2012              NA               1               1               1
## 2015               1               1               2               2
##      BX07.2.1f1.19.1 BX07.2.1f1.19.2 BX07.2.1f1.2.1 BX07.2.1f1.2.2
## 2012               1               1              1              1
## 2015               1               2              1              2
##      BX07.2.1f1.20.1 BX07.2.1f1.20.2 BX07.2.1f1.21.1 BX07.2.1f1.21.2
## 2012               1               1               1               1
## 2015               1               2               1               1
##      BX07.2.1f1.22.1 BX07.2.1f1.22.2 BX07.2.1f1.23.1 BX07.2.1f1.23.2
## 2012               1               1               1               1
## 2015               2               1               1               1
##      BX07.2.1f1.24.1 BX07.2.1f1.24.2 BX07.2.1f1.25.1 BX07.2.1f1.25.2
## 2012               1               1               1               1
## 2015              NA               1               1               1
##      BX07.2.1f1.26.1 BX07.2.1f1.26.2 BX07.2.1f1.27.1 BX07.2.1f1.28.1
## 2012               1               1               1               1
## 2015               1               1               1               1
##      BX07.2.1f1.28.2 BX07.2.1f1.29.1 BX07.2.1f1.29.2 BX07.2.1f1.3.1
## 2012               1               1               1              1
## 2015               1               1               1              2
##      BX07.2.1f1.3.2 BX07.2.1f1.30.1 BX07.2.1f1.30.2 BX07.2.1f1.31.1
## 2012              1               1               1               1
## 2015              1               1               1               1
##      BX07.2.1f1.31.2 BX07.2.1f1.32.1 BX07.2.1f1.32.2 BX07.2.1f1.33.1
## 2012               1               1               1               1
## 2015               1               1               1               1
##      BX07.2.1f1.33.2 BX07.2.1f1.34.1 BX07.2.1f1.34.2 BX07.2.1f1.35.1
## 2012               1               1               1               1
## 2015               1               1               1               1
##      BX07.2.1f1.35.2 BX07.2.1f1.36.1 BX07.2.1f1.36.2 BX07.2.1f1.37.1
## 2012               1               1               1               1
## 2015               1               1               1               1
##      BX07.2.1f1.37.2 BX07.2.1f1.38.1 BX07.2.1f1.38.2 BX07.2.1f1.39.1
## 2012               1               1               1               1
## 2015               1               1               1               1
##      BX07.2.1f1.39.2 BX07.2.1f1.4.1 BX07.2.1f1.4.2 BX07.2.1f1.40.1
## 2012               1              1              1               1
## 2015               1              1              1               1
##      BX07.2.1f1.40.2 BX07.2.1f1.41.2 BX07.2.1f1.42.1 BX07.2.1f1.42.2
## 2012               1               1               1               1
## 2015               1               1               1               1
##      BX07.2.1f1.43.1 BX07.2.1f1.43.2 BX07.2.1f1.44.1 BX07.2.1f1.44.2
## 2012               1               1               1               1
## 2015               1               2               1               1
##      BX07.2.1f1.45.1 BX07.2.1f1.45.2 BX07.2.1f1.46.1 BX07.2.1f1.46.2
## 2012               1               1              NA               1
## 2015               1               1               2               2
##      BX07.2.1f1.47.1 BX07.2.1f1.47.2 BX07.2.1f1.48.1 BX07.2.1f1.48.2
## 2012               1               1               1               1
## 2015               1               1               1               1
##      BX07.2.1f1.49.1 BX07.2.1f1.49.2 BX07.2.1f1.5.1 BX07.2.1f1.5.2
## 2012               1               1              1              1
## 2015               1               1              2              1
##      BX07.2.1f1.50.1 BX07.2.1f1.50.2 BX07.2.1f1.6.1 BX07.2.1f1.6.2
## 2012               1               1              1              1
## 2015               1               1              1              2
##      BX07.2.1f1.7.1 BX07.2.1f1.7.2 BX07.2.1f1.8.1 BX07.2.1f1.8.2
## 2012              1              1              1              1
## 2015              1              2              1              1
##      BX07.2.1f1.9.1 BX07.2.1f1.9.2 BX07.2.1f2.1 BX07.2.1f2.10
## 2012              1              1            1             1
## 2015              2              1            1             1
##      BX07.2.1f2.11 BX07.2.1f2.12 BX07.2.1f2.13 BX07.2.1f2.14 BX07.2.1f2.15
## 2012             1             1             1             1             1
## 2015             1             1             1             1             1
##      BX07.2.1f2.16 BX07.2.1f2.17 BX07.2.1f2.18 BX07.2.1f2.19 BX07.2.1f2.2
## 2012             1             1             1             1            1
## 2015             2             2             2             2            1
##      BX07.2.1f2.20 BX07.2.1f2.21 BX07.2.1f2.25 BX07.2.1f2.26 BX07.2.1f2.27
## 2012             1             1             1            NA             1
## 2015             2             1             2             2             1
##      BX07.2.1f2.28 BX07.2.1f2.29 BX07.2.1f2.3 BX07.2.1f2.30 BX07.2.1f2.31
## 2012             1             1            1             1             1
## 2015             2             1            1             2             2
##      BX07.2.1f2.34 BX07.2.1f2.35 BX07.2.1f2.36 BX07.2.1f2.37 BX07.2.1f2.38
## 2012             1             1             1             1             1
## 2015             2             2             2             1             2
##      BX07.2.1f2.39 BX07.2.1f2.4 BX07.2.1f2.40 BX07.2.1f2.41 BX07.2.1f2.42
## 2012             1            1             1             1             1
## 2015             2            1             2             2             2
##      BX07.2.1f2.43 BX07.2.1f2.44 BX07.2.1f2.45 BX07.2.1f2.46 BX07.2.1f2.47
## 2012             1             1             1             1             1
## 2015             2             1             2             2             2
##      BX07.2.1f2.48 BX07.2.1f2.49 BX07.2.1f2.5 BX07.2.1f2.50 BX07.2.1f2.51
## 2012             1             1            1             1             1
## 2015             2             2            1             1             1
##      BX07.2.1f2.52 BX07.2.1f2.53 BX07.2.1f2.54 BX07.2.1f2.55 BX07.2.1f2.56
## 2012             1             1             1             1             1
## 2015             1             1             2             2             1
##      BX07.2.1f2.57 BX07.2.1f2.58 BX07.2.1f2.59 BX07.2.1f2.6 BX07.2.1f2.60
## 2012             1             1             1            1             1
## 2015             1             1             1            1             1
##      BX07.2.1f2.61 BX07.2.1f2.62 BX07.2.1f2.63 BX07.2.1f2.64 BX07.2.1f2.65
## 2012             1             1             1             1             1
## 2015             1             1             1             1             1
##      BX07.2.1f2.66 BX07.2.1f2.67 BX07.2.1f2.68 BX07.2.1f2.69 BX07.2.1f2.7
## 2012             1             1             1             1            1
## 2015             1             1             1             1            1
##      BX07.2.1f2.70 BX07.2.1f2.71 BX07.2.1f2.72 BX07.2.1f2.73 BX07.2.1f2.74
## 2012             1             1             1             1             1
## 2015             2             2             1             2             2
##      BX07.2.1f2.75 BX07.2.1f2.76 BX07.2.1f2.77 BX07.2.1f2.78 BX07.2.1f2.79
## 2012             1             1             1             1             1
## 2015             2             2             1             1             2
##      BX07.2.1f2.8 BX07.2.1f2.80 BX07.2.1f2.81 BX07.2.1f2.82 BX07.2.1f2.83
## 2012            1             1            NA             1             1
## 2015            1             2             2             1             1
##      BX07.2.1f2.84 BX07.2.1f2.85 BX07.2.1f2.86 BX07.2.1f2.87 BX07.2.1f2.88
## 2012             1             1             1             1             1
## 2015             1             1             1             1             1
##      BX07.2.1f2.89 BX07.2.1f2.9 BX07.2.1f2.90 BX07.2.1f2.91 BX07.2.1f2.92
## 2012             1            1             1             1             1
## 2015            NA            1             1             1             1
##      BX07.2.1f2.93 BX07.2.1f2.94 BX07.2.1f2.95 BX07.2.1f2.96 BX07.2.1f2.97
## 2012             1             1            NA             1             1
## 2015             1             1             1             1             1
##      BX07.2.1f2.98 BX07.2.1f2.99 BX07.3.2f1.1 BX07.3.2f1.100 BX07.3.2f1.11
## 2012             1             1            1              1             1
## 2015             1             1           NA             NA            NA
##      BX07.3.2f1.12 BX07.3.2f1.13 BX07.3.2f1.14 BX07.3.2f1.15 BX07.3.2f1.17
## 2012             1             1             1            NA             1
## 2015            NA            NA            NA             1            NA
##      BX07.3.2f1.18 BX07.3.2f1.19 BX07.3.2f1.20 BX07.3.2f1.21 BX07.3.2f1.22
## 2012             1             1             1             1             1
## 2015            NA            NA             1            NA            NA
##      BX07.3.2f1.23 BX07.3.2f1.24 BX07.3.2f1.25 BX07.3.2f1.26 BX07.3.2f1.27
## 2012             1             1             1             1             1
## 2015             1             1            NA            NA            NA
##      BX07.3.2f1.29 BX07.3.2f1.3 BX07.3.2f1.30 BX07.3.2f1.31 BX07.3.2f1.32
## 2012             1            1             1             1             1
## 2015            NA           NA            NA             1             1
##      BX07.3.2f1.34 BX07.3.2f1.36 BX07.3.2f1.37 BX07.3.2f1.39 BX07.3.2f1.4
## 2012             1             1             1             1            1
## 2015            NA             1             1            NA           NA
##      BX07.3.2f1.41 BX07.3.2f1.42 BX07.3.2f1.44 BX07.3.2f1.47 BX07.3.2f1.48
## 2012             1             1             1             1             1
## 2015            NA            NA             1            NA            NA
##      BX07.3.2f1.49 BX07.3.2f1.5 BX07.3.2f1.50 BX07.3.2f1.51 BX07.3.2f1.52
## 2012             1            1             1             1             1
## 2015            NA           NA            NA             1             1
##      BX07.3.2f1.53 BX07.3.2f1.54 BX07.3.2f1.55 BX07.3.2f1.56 BX07.3.2f1.57
## 2012             1             1             1             1             1
## 2015            NA            NA            NA            NA            NA
##      BX07.3.2f1.58 BX07.3.2f1.59 BX07.3.2f1.6 BX07.3.2f1.60 BX07.3.2f1.61
## 2012             1             1            1             1             1
## 2015            NA            NA           NA             1            NA
##      BX07.3.2f1.62 BX07.3.2f1.63 BX07.3.2f1.65 BX07.3.2f1.67 BX07.3.2f1.69
## 2012             1             1             1             1             1
## 2015            NA            NA            NA             1            NA
##      BX07.3.2f1.7 BX07.3.2f1.73 BX07.3.2f1.75 BX07.3.2f1.77 BX07.3.2f1.8
## 2012            1             1             1             1            1
## 2015           NA             1            NA            NA            1
##      BX07.3.2f1.83 BX07.3.2f1.84 BX07.3.2f1.86 BX07.3.2f1.9 BX07.3.2f1.98
## 2012            NA             1             1            1             1
## 2015             1            NA            NA            1            NA
##      BX07.3.2f2.10 BX07.3.2f2.17 BX07.3.2f2.18 BX07.3.2f2.20 BX07.3.2f2.22
## 2012             1             1             1             1             1
## 2015            NA            NA            NA            NA            NA
##      BX07.3.2f2.24 BX07.3.2f2.26 BX07.3.2f2.27 BX07.3.2f2.29 BX07.3.2f2.30
## 2012             1             1             1             1             1
## 2015            NA            NA             1            NA            NA
##      BX07.3.2f2.31 BX07.3.2f2.37 BX07.3.2f2.39 BX07.3.2f2.41 BX07.3.2f2.42
## 2012             1             1             1             1             1
## 2015            NA            NA            NA             1             1
##      BX07.3.2f2.43 BX07.3.2f2.44 BX07.3.2f2.47 BX07.3.2f2.53 BX07.3.2f2.57
## 2012             1            NA             1             1             1
## 2015            NA             1            NA             1             1
##      BX07.3.2f2.58 BX07.3.2f2.59 BX07.3.2f2.61 BX07.3.2f2.62 BX07.3.2f2.67
## 2012             1             1             1             1             1
## 2015             1             1            NA            NA            NA
##      BX07.3.2f2.68 BX07.3.2f2.7 BX07.3.2f2.70 BX07.3.2f2.71 BX07.3.2f2.72
## 2012             1            1             1             1             1
## 2015            NA            1            NA            NA            NA
##      BX07.3.2f2.73 BX07.3.2f2.76 BX07.3.2f2.78 BX07.3.2f2.80 BX07.3.2f2.83
## 2012             1             1             1             1             1
## 2015            NA            NA            NA             1             1
##      BX07.3.2f2.84 BX07.3.2f2.86 BX07.3.2f2.87 BX07.3.2f2.89 BX07.3.2f2.91
## 2012             1             1             1             1             1
## 2015             1            NA            NA             1            NA
##      Dic2 dicA dicB dicC dicE dicF dicG dicH dicI dicJ Lloyd pescadou
## 2012    1    2    2    2    2    2    2    2    2    2     5      149
## 2015    2   NA   NA   NA   NA   NA   NA   NA   NA   NA     3      100
##      Silur Soldur TT06DC 38.11 TT06DC 38.12 TT06DC 38.13 TT06DC 38.15
## 2012     4     10            1            1            1            1
## 2015     2      2            1            1            2            2
##      TT06DC 38.18 TT06DC 38.19 TT06DC 38.23 TT06DC 38.24 TT06DC 38.25
## 2012            1            1            1            2            1
## 2015           NA            2            2            2            1
##      TT06DC 38.27 TT06DC 38.29 TT06DC 38.3 TT06DC 38.32 TT06DC 38.34
## 2012            1            2           2            2            2
## 2015            1            1           1            1            1
##      TT06DC 38.35 TT06DC 38.36 TT06DC 38.37 TT06DC 38.38 TT06DC 38.39
## 2012            1            2            1            2            1
## 2015            2            2            2            2            2
##      TT06DC 38.41 TT06DC 38.42 TT06DC 38.44 TT06DC 38.47 TT06DC 38.49
## 2012            1            1            2            2            2
## 2015            1            1            1            1            1
##      TT06DC 38.50 TT06DC 38.51 TT06DC 38.52 TT06DC 38.53 TT06DC 38.54
## 2012            2            2            2            1            2
## 2015            2            2            2            2            2
##      TT06DC 38.7 TT06DC 38.8 TT06DC 38.9 TT06DC 39.10 TT06DC 39.11
## 2012           1           2           1            1            2
## 2015           1           1           1            1            2
##      TT06DC 39.13 TT06DC 39.16 TT06DC 39.17 TT06DC 39.18 TT06DC 39.20
## 2012            1            2            2            1            1
## 2015            2            2            2            2            1
##      TT06DC 39.21 TT06DC 39.23 TT06DC 39.24 TT06DC 39.25 TT06DC 39.26
## 2012            1            1            1            1            2
## 2015            1            1            1            1            1
##      TT06DC 39.27 TT06DC 39.28 TT06DC 39.29 TT06DC 39.3 TT06DC 39.30
## 2012            2            2            2           1            1
## 2015            1            1            1           1            1
##      TT06DC 39.31 TT06DC 39.32 TT06DC 39.34 TT06DC 39.35 TT06DC 39.36
## 2012            2            2            1            2            1
## 2015            1            1            1            2            2
##      TT06DC 39.37 TT06DC 39.4 TT06DC 39.6 TT06DC 39.7 TT06DC 39.8
## 2012            1           2           2           1           1
## 2015            2           1           1           1           1
##      TT06DC 39.9 TT06DC 40.1 TT06DC 40.10 TT06DC 40.11 TT06DC 40.12
## 2012           2           2            2            1            2
## 2015           1           2            1            1            1
##      TT06DC 40.14 TT06DC 40.15 TT06DC 40.16 TT06DC 40.17 TT06DC 40.18
## 2012            1            1            3            2            2
## 2015            1            1            1            1            1
##      TT06DC 40.22 TT06DC 40.23 TT06DC 40.24 TT06DC 40.27 TT06DC 40.29
## 2012            1            1            3            2            1
## 2015            1            1            1            1            2
##      TT06DC 40.31 TT06DC 40.32 TT06DC 40.33 TT06DC 40.34 TT06DC 40.35
## 2012            1            1            2            1            1
## 2015            2            2            2            2            1
##      TT06DC 40.37 TT06DC 40.38 TT06DC 40.39 TT06DC 40.4 TT06DC 40.40
## 2012            2            1            1           2            1
## 2015            1            1            1           2            1
##      TT06DC 40.41 TT06DC 40.45 TT06DC 40.46 TT06DC 40.47 TT06DC 40.49
## 2012            2            2            2            2            1
## 2015            1            1            1            1            1
##      TT06DC 40.5 TT06DC 40.52 TT06DC 40.53 TT06DC 40.6 TT06DC 40.7
## 2012           2            2            1           1           2
## 2015           1            1            1           1           1
##      TT06DC 40.8 TT06DC 42.1 TT06DC 42.11 TT06DC 42.13 TT06DC 42.14
## 2012           2           2            1            1            2
## 2015           1           1            2            2            2
##      TT06DC 42.15 TT06DC 42.16 TT06DC 42.17 TT06DC 42.18 TT06DC 42.19
## 2012            2            1            1            2            2
## 2015            2            2            1            1            1
##      TT06DC 42.2 TT06DC 42.21 TT06DC 42.22 TT06DC 42.23 TT06DC 42.24
## 2012           2            1            2            1            1
## 2015           1            1            1            1            2
##      TT06DC 42.25 TT06DC 42.26 TT06DC 42.28 TT06DC 42.3 TT06DC 42.35
## 2012            1            1            1           1            1
## 2015            1            1            2           1            1
##      TT06DC 42.36 TT06DC 42.38 TT06DC 42.4 TT06DC 42.40 TT06DC 42.41
## 2012            2            1           2            1            2
## 2015            1            2           1            1            1
##      TT06DC 42.43 TT06DC 42.44 TT06DC 42.45 TT06DC 42.46 TT06DC 42.48
## 2012            2            2            1            2            2
## 2015            1            2            1            1            1
##      TT06DC 42.49 TT06DC 42.5 TT06DC 42.53 TT06DC 42.55 TT06DC 42.6
## 2012            2           1            1            1           1
## 2015            1           1            1            1           1
##      TT06DC 42.7 TT06DC 42.9 TT06DC 44.1 TT06DC 44.10 TT06DC 44.11
## 2012           2           1           2            2            1
## 2015           1           1           2            2            1
##      TT06DC 44.13 TT06DC 44.14 TT06DC 44.19 TT06DC 44.22 TT06DC 44.24
## 2012            2            1            2            1            1
## 2015            1            2            2            2            2
##      TT06DC 44.28 TT06DC 44.3 TT06DC 44.31 TT06DC 44.33 TT06DC 44.34
## 2012            2           2            1            1            1
## 2015            2           2            1            1            1
##      TT06DC 44.35 TT06DC 44.36 TT06DC 44.38 TT06DC 44.39 TT06DC 44.41
## 2012            1            2            1            2            1
## 2015            1            1            2            2            2
##      TT06DC 44.42 TT06DC 44.43 TT06DC 44.45 TT06DC 44.47 TT06DC 44.48
## 2012            2            2            2            1            1
## 2015            2            2            1            1            1
##      TT06DC 44.5 TT06DC 44.50 TT06DC 44.52 TT06DC 44.54 TT06DC 44.55
## 2012           1            1            1            2            2
## 2015           2            1            1            1            1
##      TT06DC 44.56 TT06DC 44.61 TT06DC 44.62 TT06DC 44.64 TT06DC 44.65
## 2012            2            1            2            1            1
## 2015            1            1            1            1            1
##      TT06DC 44.7
## 2012           2
## 2015           2
```

```
levels(data$pop)
```

```
##  [1] "Dic2"          "Dic2 x Lloyd"  "Dic2 x Silur"  "Dic2 x Soldur"
##  [5] "dicA"          "dicB"          "dicC"          "dicD"         
##  [9] "dicE"          "dicF"          "dicG"          "dicH"         
## [13] "dicI"          "dicJ"          "Lloyd"         "pescadou"     
## [17] "Silur"         "Soldur"
```

```
## Same level for all crosses
data$cross <- data$pop
levels(data$cross)[2:4] <- c("cross","cross","cross")


head(data)
```

```
##     plante          pop     genotype NV     Elisa QPCR X Y neigh1 neigh2
## 27      p1       Soldur       Soldur  2 0.9502664   NA 1 1  side1  side2
## 70    p144 Dic2 x Silur TT06DC 44.28  3 0.7173187   NA 2 1  side1  side2
## 71    p145        Silur        Silur  4 1.2568765   NA 3 1  side1  side2
## 199   p284 Dic2 x Silur TT06DC 44.39  0 1.0147956   NA 4 1  side1  side2
## 200   p285 Dic2 x Silur TT06DC 38.27  3 1.0133161   NA 5 1  side1  side2
## 330   p429 Dic2 x Silur TT06DC 42.15  3 0.2261215   NA 6 1  side1  side2
##     neigh3       neigh4        neigh5        neigh6        neigh7
## 27   side3 TT06DC 44.28         lloyd  TT06DC 39.35         side7
## 70   side3        Silur   TT06DC 40.4         lloyd  TT06DC 39.35
## 71   side3 TT06DC 44.39 BX07.2.1f2.71   TT06DC 40.4         lloyd
## 199  side3 TT06DC 38.27  TT06DC 38.12 BX07.2.1f2.71   TT06DC 40.4
## 200  side3 TT06DC 42.15 BX07.2.1f2.53  TT06DC 38.12 BX07.2.1f2.71
## 330  side3 BX07.3.2f1.7 BX07.2.1f2.91 BX07.2.1f2.53  TT06DC 38.12
##           neigh8 X_Y Year  cross
## 27         side8 1_1 2012 Soldur
## 70        Soldur 2_1 2012  cross
## 71  TT06DC 44.28 3_1 2012  Silur
## 199        Silur 4_1 2012  cross
## 200 TT06DC 44.39 5_1 2012  cross
## 330 TT06DC 38.27 6_1 2012  cross
```

```
levels(data$pop)
```

```
##  [1] "Dic2"          "Dic2 x Lloyd"  "Dic2 x Silur"  "Dic2 x Soldur"
##  [5] "dicA"          "dicB"          "dicC"          "dicD"         
##  [9] "dicE"          "dicF"          "dicG"          "dicH"         
## [13] "dicI"          "dicJ"          "Lloyd"         "pescadou"     
## [17] "Silur"         "Soldur"
```

```
paper <- c("pescadou","Dic2 x Lloyd","Dic2 x Silur")
#paper <- c("Dic2","Lloyd","Silur","pescadou","Dic2 x Lloyd","Dic2 x Silur")

## Exclude data not used in the paper
data$NV <- ifelse(data$pop%in%paper,data$NV,NA)
data$Elisa <- ifelse(data$pop%in%paper,data$Elisa,NA)
data$QPCR <- ifelse(data$pop%in%paper,data$QPCR,NA)
data$pop <- as.factor(ifelse(data$pop%in%paper,as.character(data$pop),NA))


head(data)
```

```
##     plante          pop     genotype NV     Elisa QPCR X Y neigh1 neigh2
## 27      p1         <NA>       Soldur NA        NA   NA 1 1  side1  side2
## 70    p144 Dic2 x Silur TT06DC 44.28  3 0.7173187   NA 2 1  side1  side2
## 71    p145         <NA>        Silur NA        NA   NA 3 1  side1  side2
## 199   p284 Dic2 x Silur TT06DC 44.39  0 1.0147956   NA 4 1  side1  side2
## 200   p285 Dic2 x Silur TT06DC 38.27  3 1.0133161   NA 5 1  side1  side2
## 330   p429 Dic2 x Silur TT06DC 42.15  3 0.2261215   NA 6 1  side1  side2
##     neigh3       neigh4        neigh5        neigh6        neigh7
## 27   side3 TT06DC 44.28         lloyd  TT06DC 39.35         side7
## 70   side3        Silur   TT06DC 40.4         lloyd  TT06DC 39.35
## 71   side3 TT06DC 44.39 BX07.2.1f2.71   TT06DC 40.4         lloyd
## 199  side3 TT06DC 38.27  TT06DC 38.12 BX07.2.1f2.71   TT06DC 40.4
## 200  side3 TT06DC 42.15 BX07.2.1f2.53  TT06DC 38.12 BX07.2.1f2.71
## 330  side3 BX07.3.2f1.7 BX07.2.1f2.91 BX07.2.1f2.53  TT06DC 38.12
##           neigh8 X_Y Year  cross
## 27         side8 1_1 2012 Soldur
## 70        Soldur 2_1 2012  cross
## 71  TT06DC 44.28 3_1 2012  Silur
## 199        Silur 4_1 2012  cross
## 200 TT06DC 44.39 5_1 2012  cross
## 330 TT06DC 38.27 6_1 2012  cross
```

```
write.csv(data,file="Data2012_2015.csv",row.names=F,quote=F)
```
