# Supplementary figures and images for: Epistatic determinism of durum wheat resistance to the wheat spindle streak mosaic virus
Source: Theor Appl Genet. 2017 Apr 27;130(7):1491–505. doi: 10.1007/s00122-017-2904-6 (PMC5487696; doi:10.1007/s00122-017-2904-6)

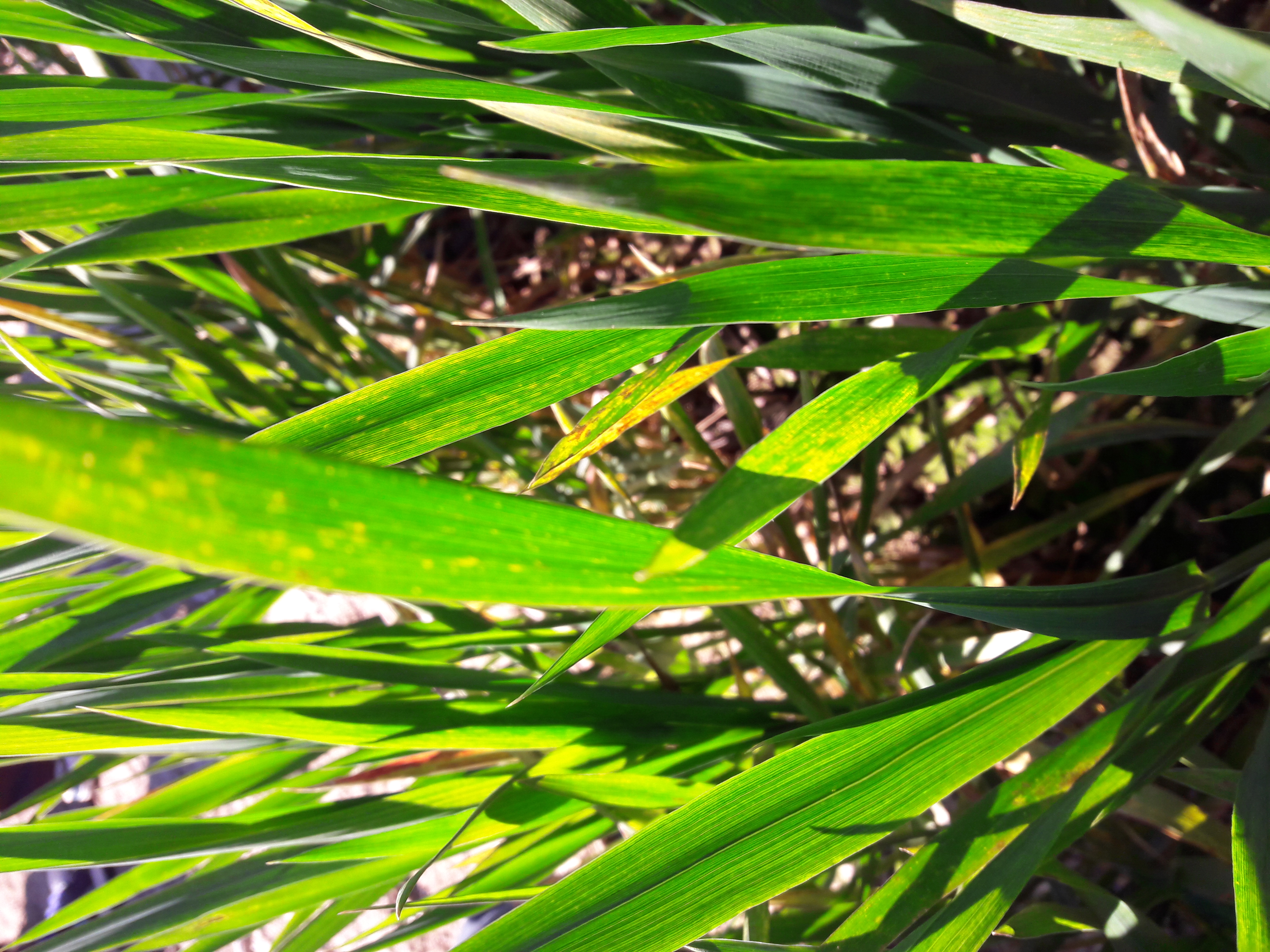

Supplement: Supplementary file 4 — Online Resource 4: Data and R scripts for reproducible QTL detection. Data and R script (.csv and.rmd format) are provided in this tar archive. A scheme aims to explain the content of each file and its role in the QTL detection pipeline. The upstream bioinformatic steps (from raw reads to consensus genetic map) are not included (GZ 72829 kb) [file 122_2017_2904_MOESM4_ESM.gz › TMP/pic_WSSMV.jpg]

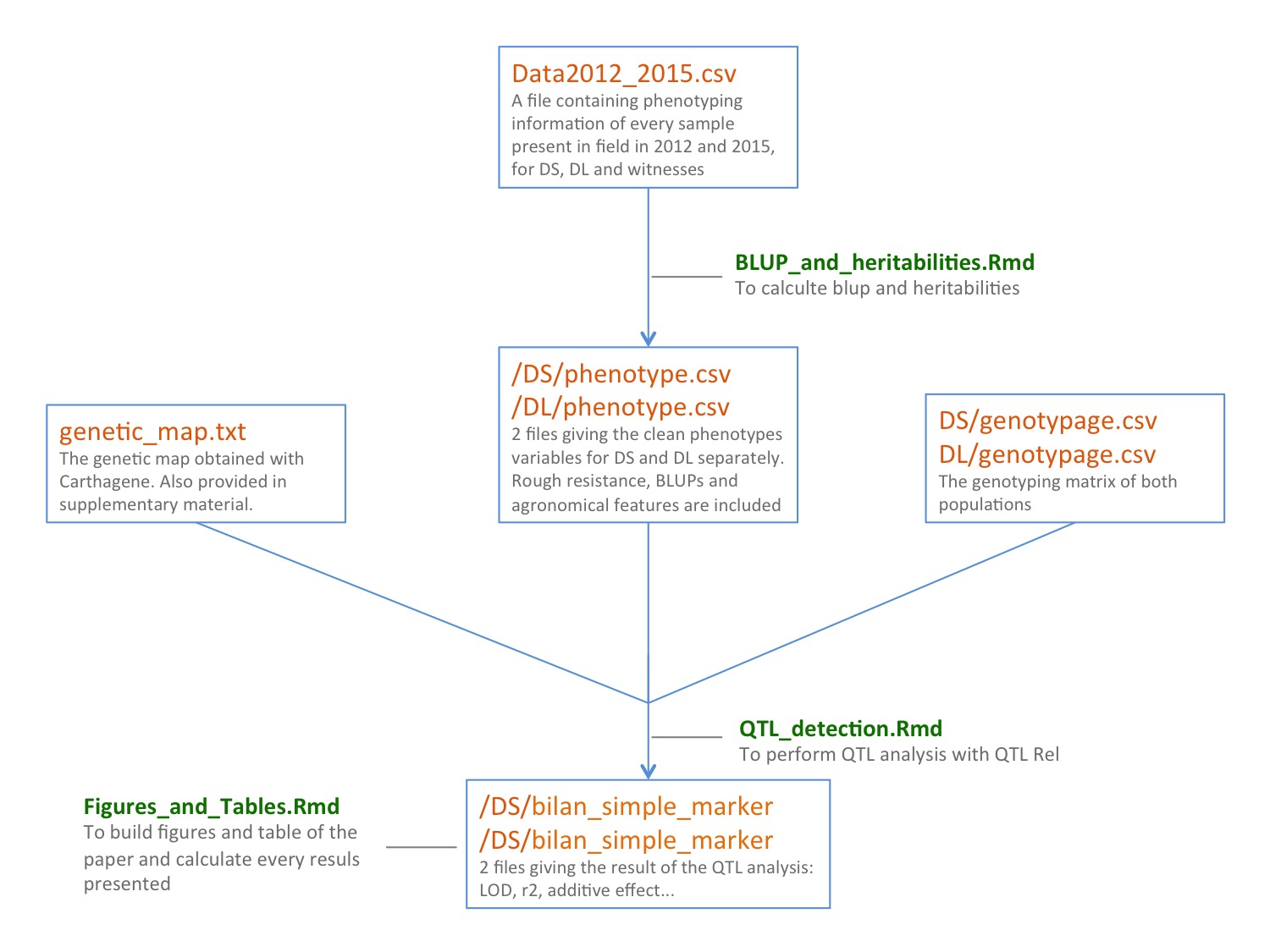

Supplement: Supplementary file 4 — Online Resource 4: Data and R scripts for reproducible QTL detection. Data and R script (.csv and.rmd format) are provided in this tar archive. A scheme aims to explain the content of each file and its role in the QTL detection pipeline. The upstream bioinformatic steps (from raw reads to consensus genetic map) are not included (GZ 72829 kb) [file 122_2017_2904_MOESM4_ESM.gz › TMP/SCRIPT/Explanation_Pipeline_Analysis.jpg]

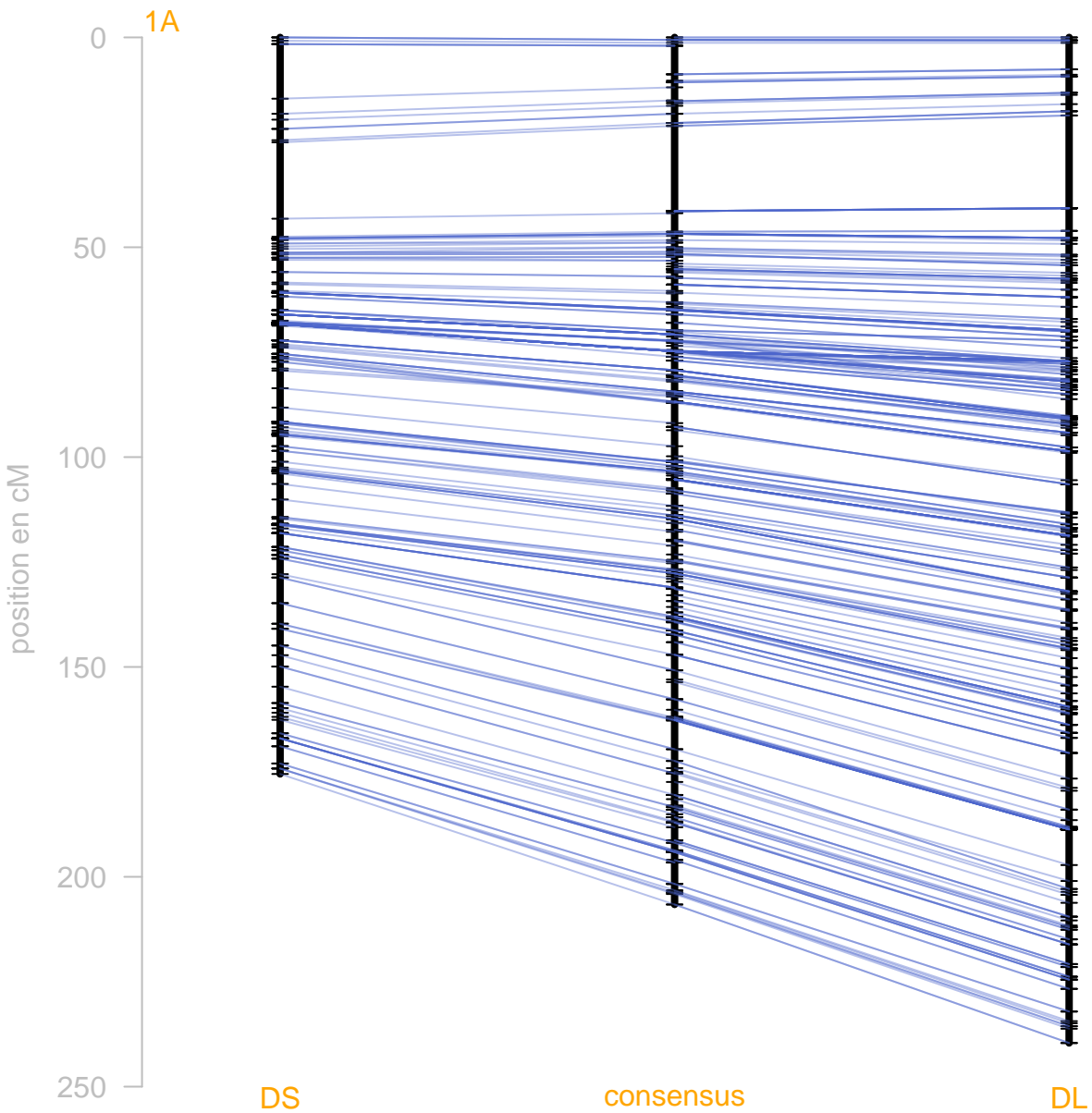

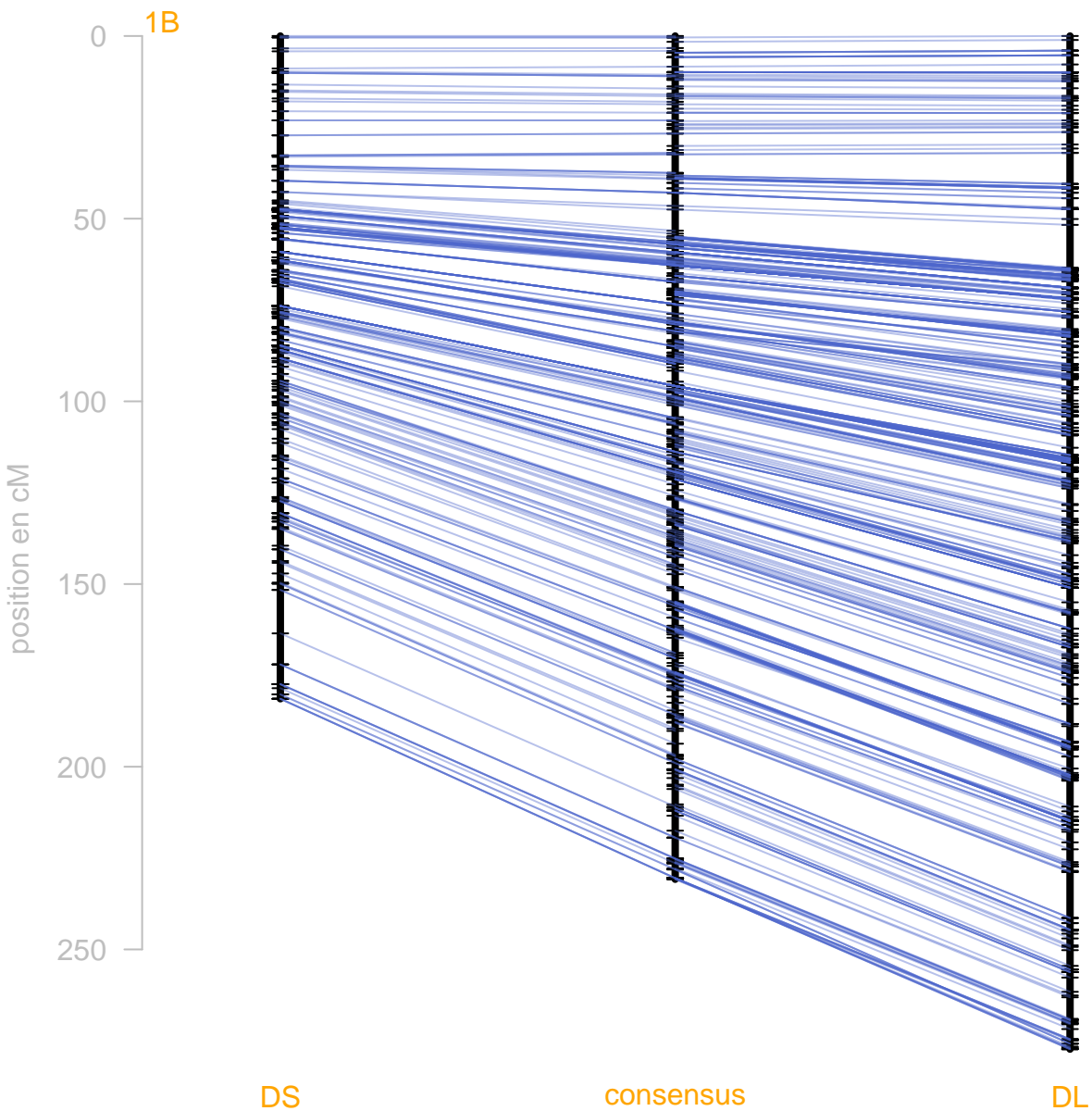

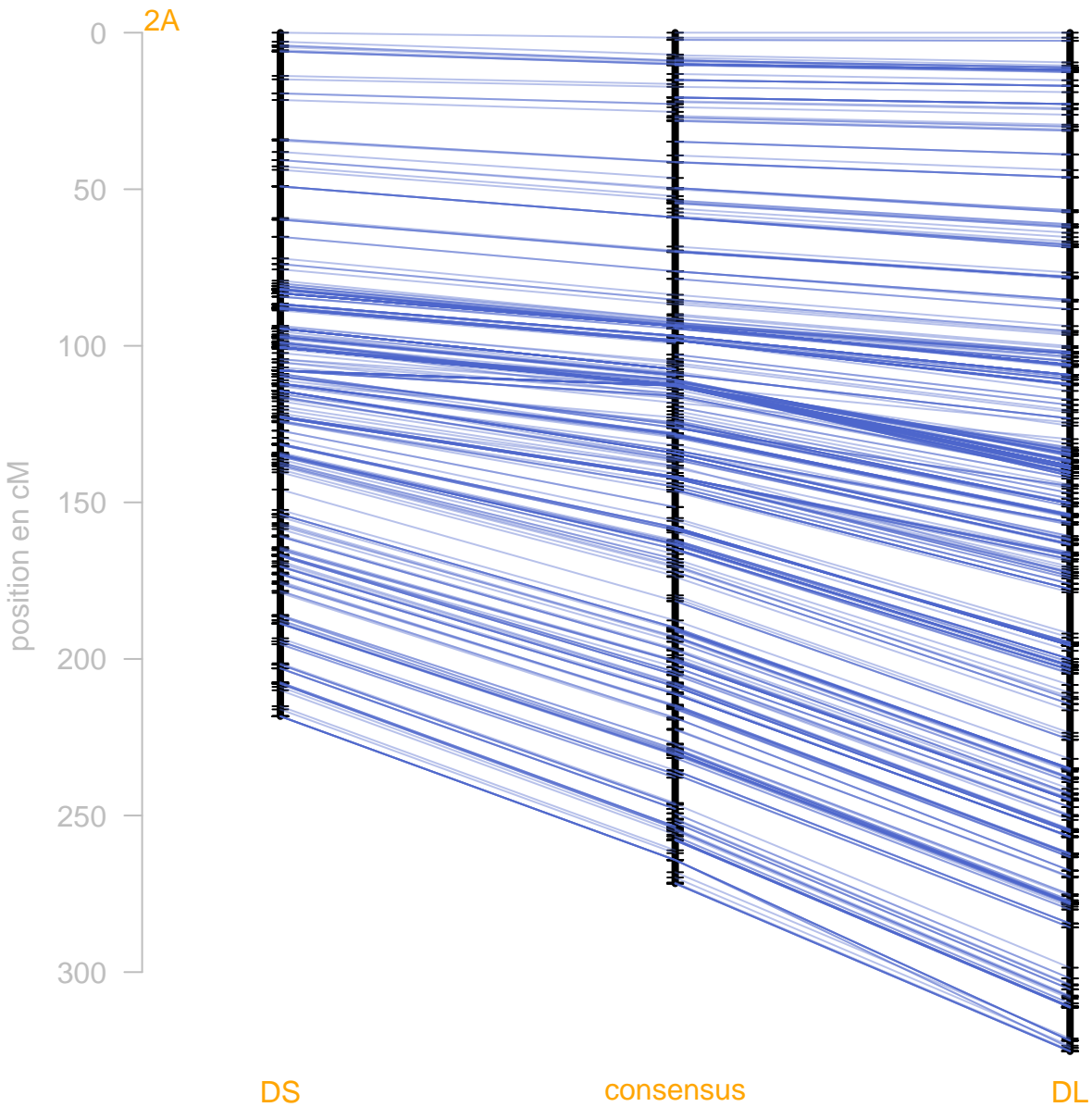

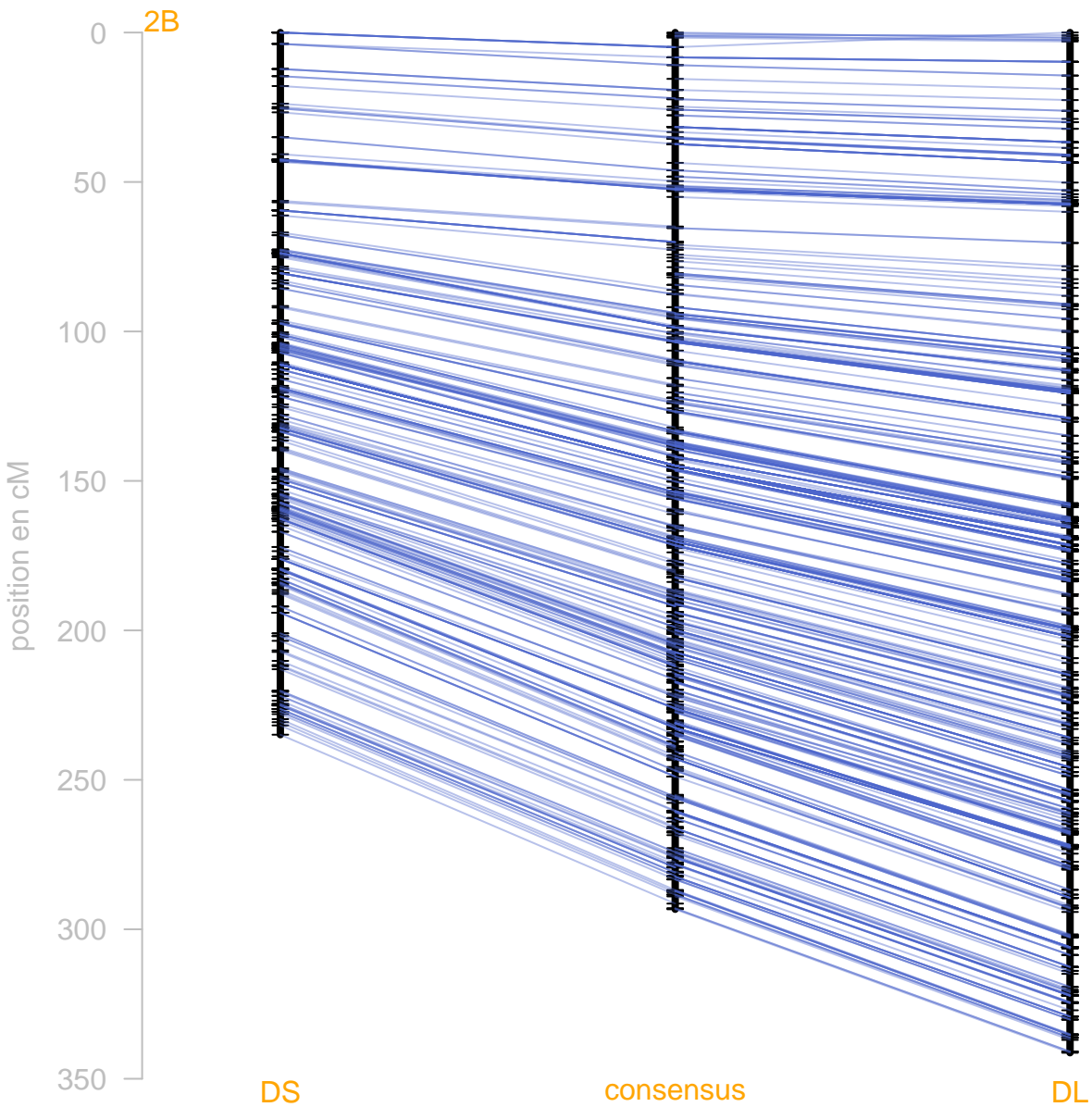

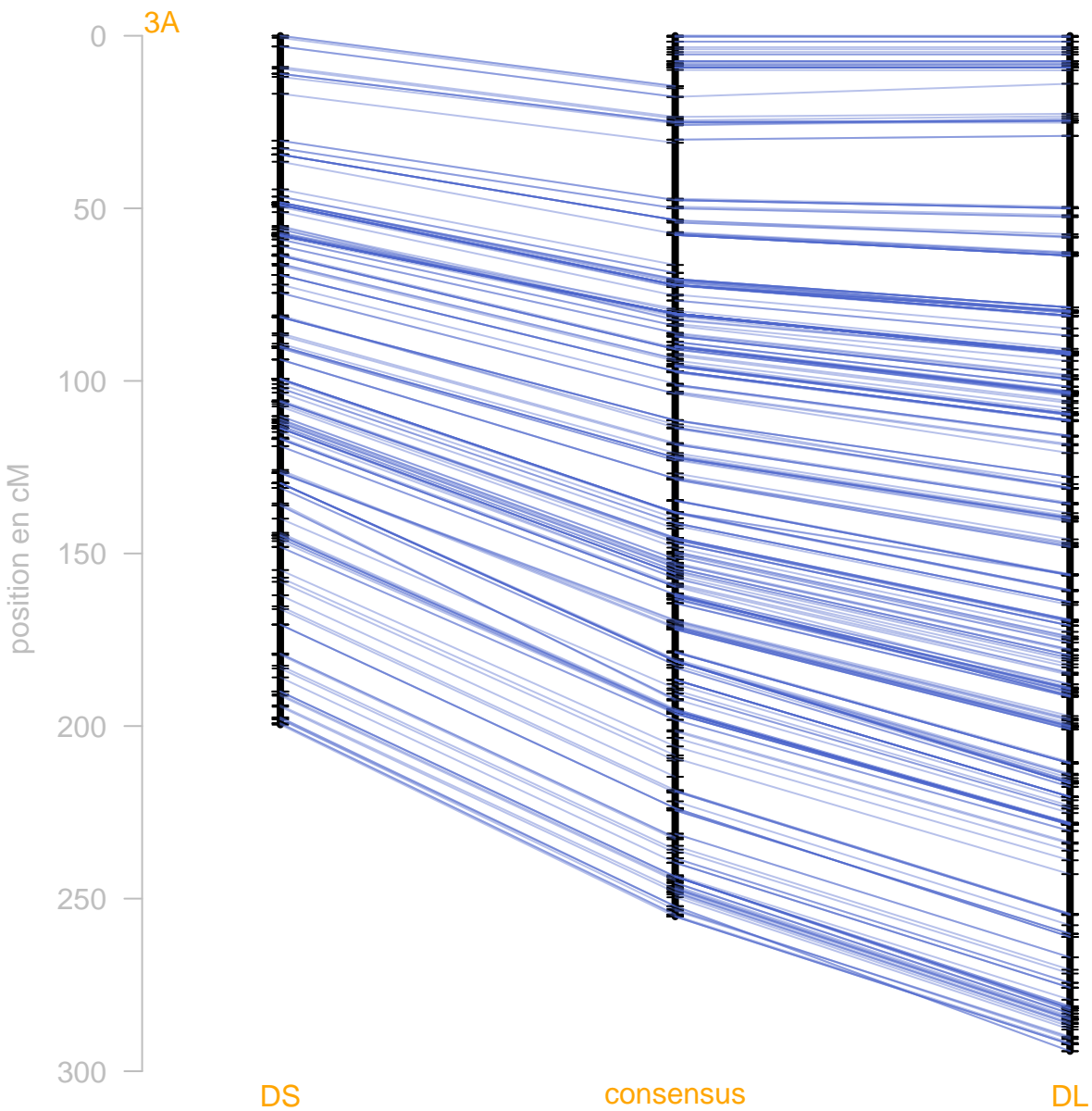

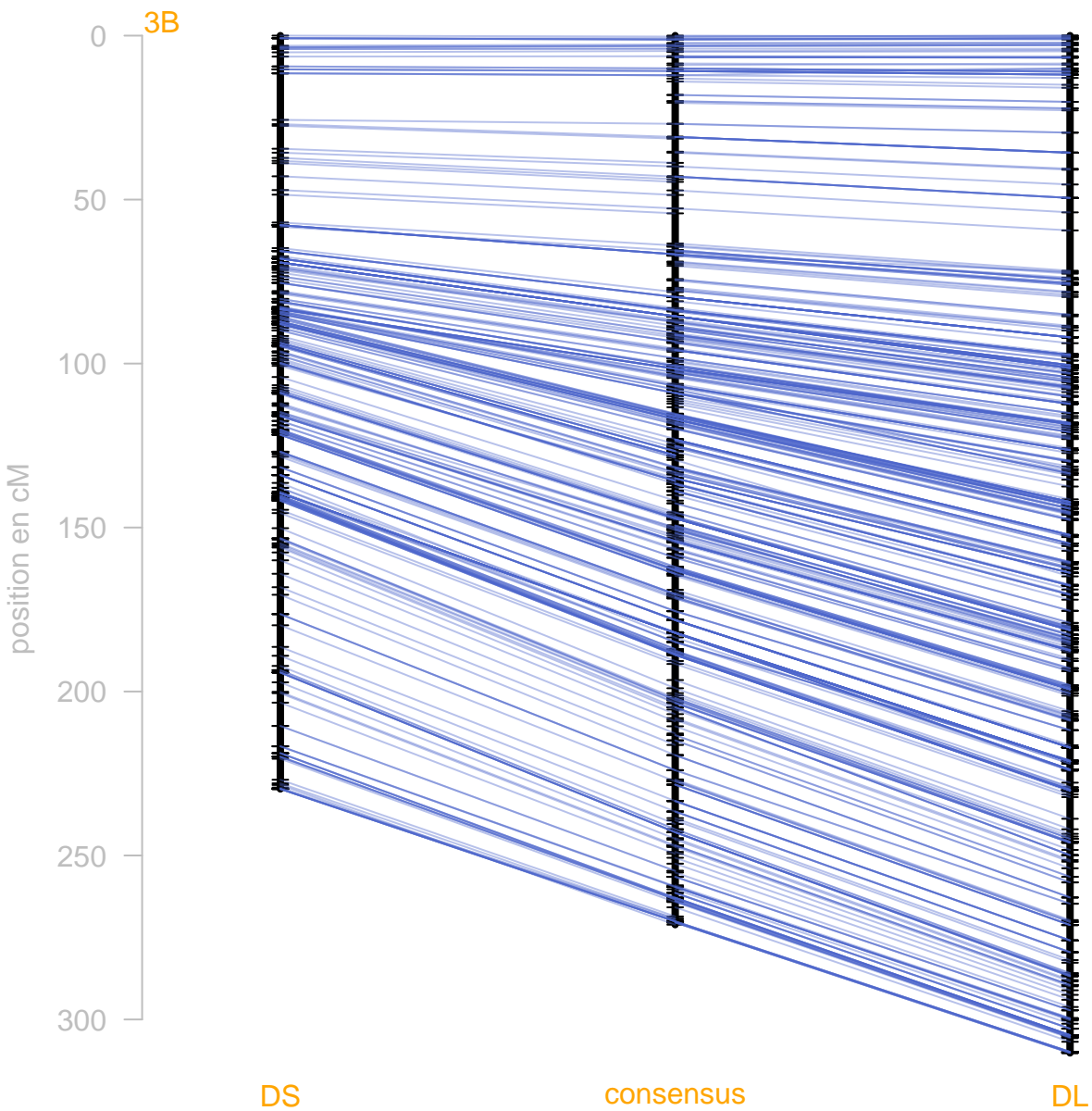

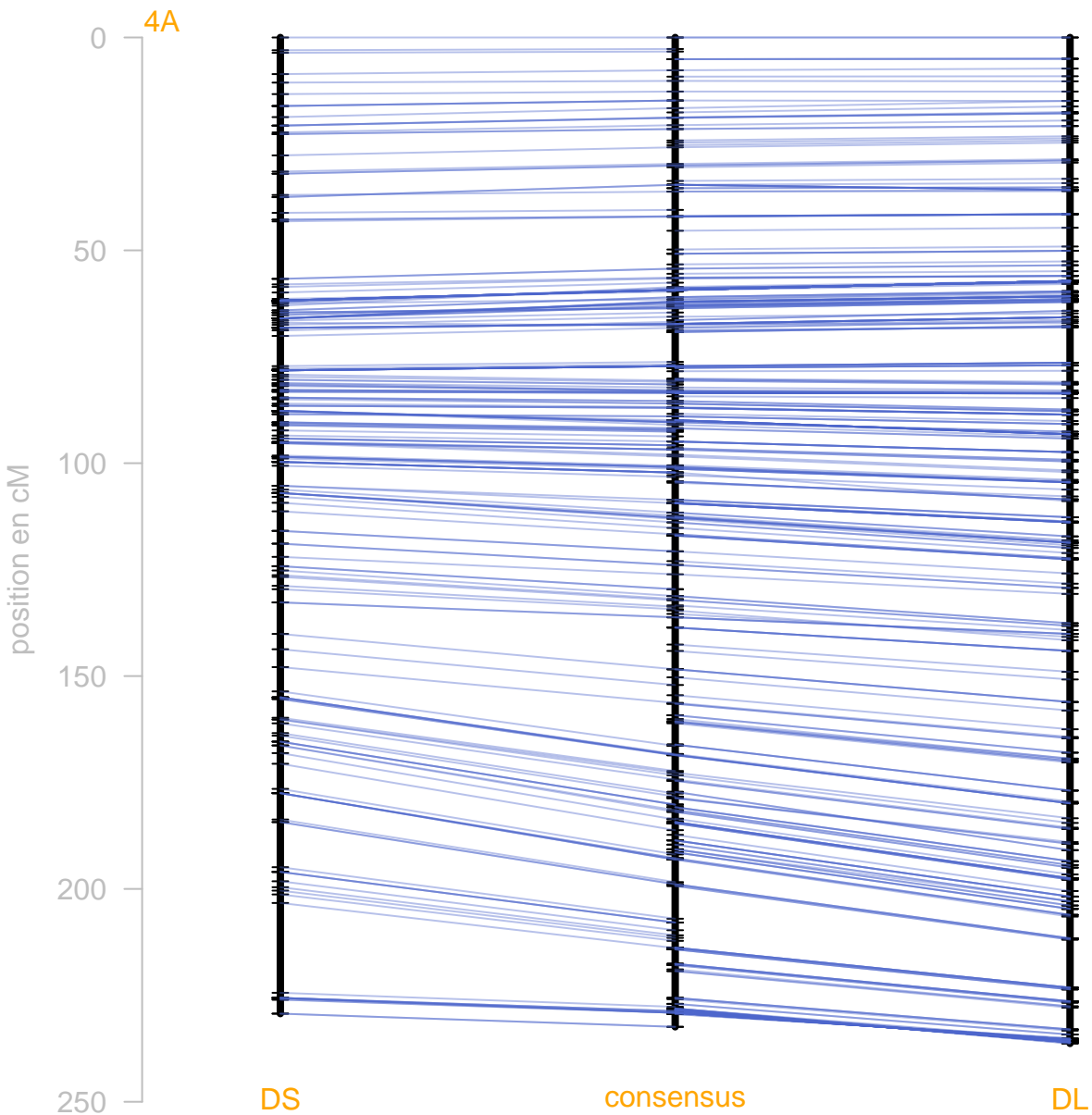

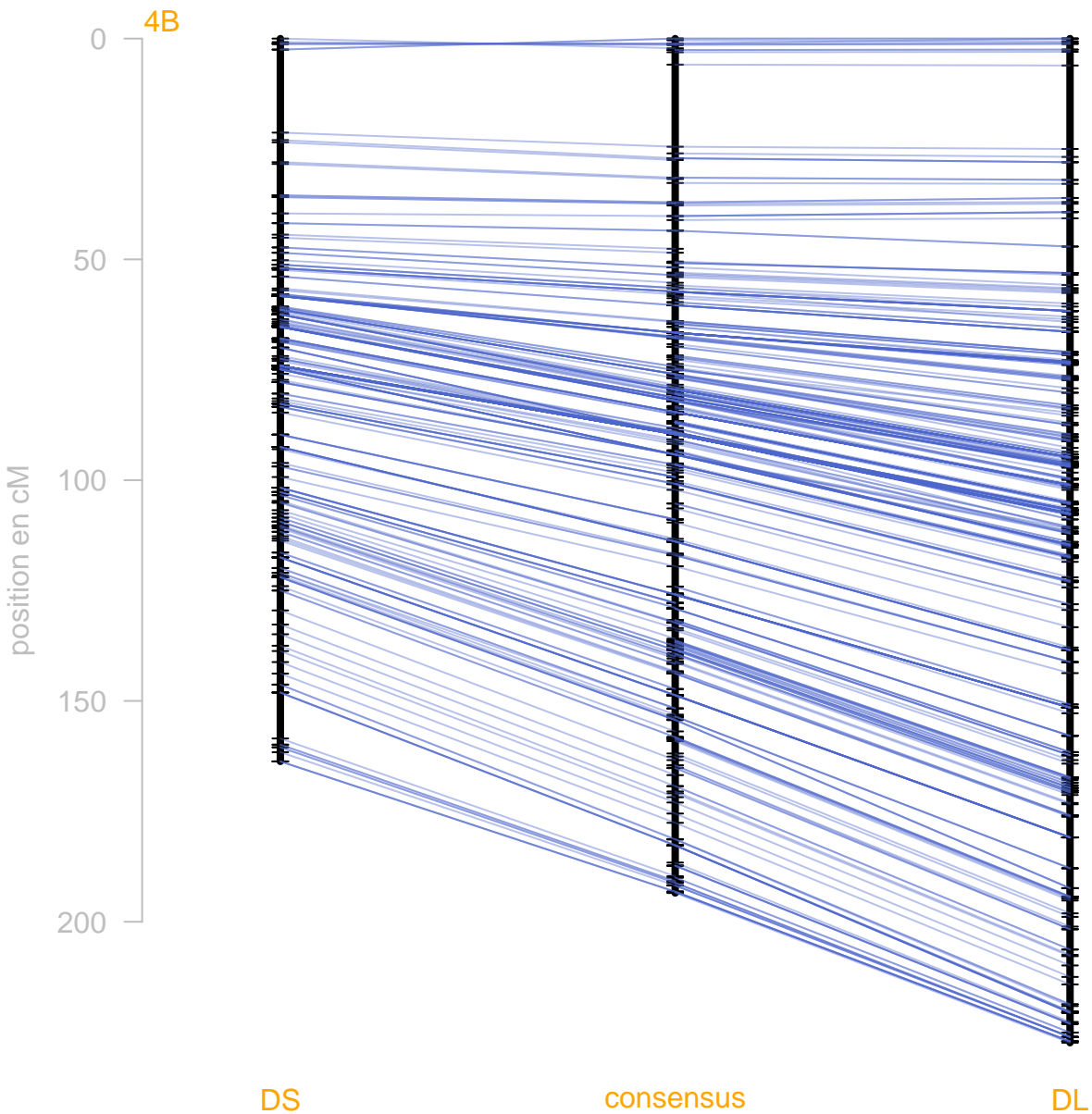

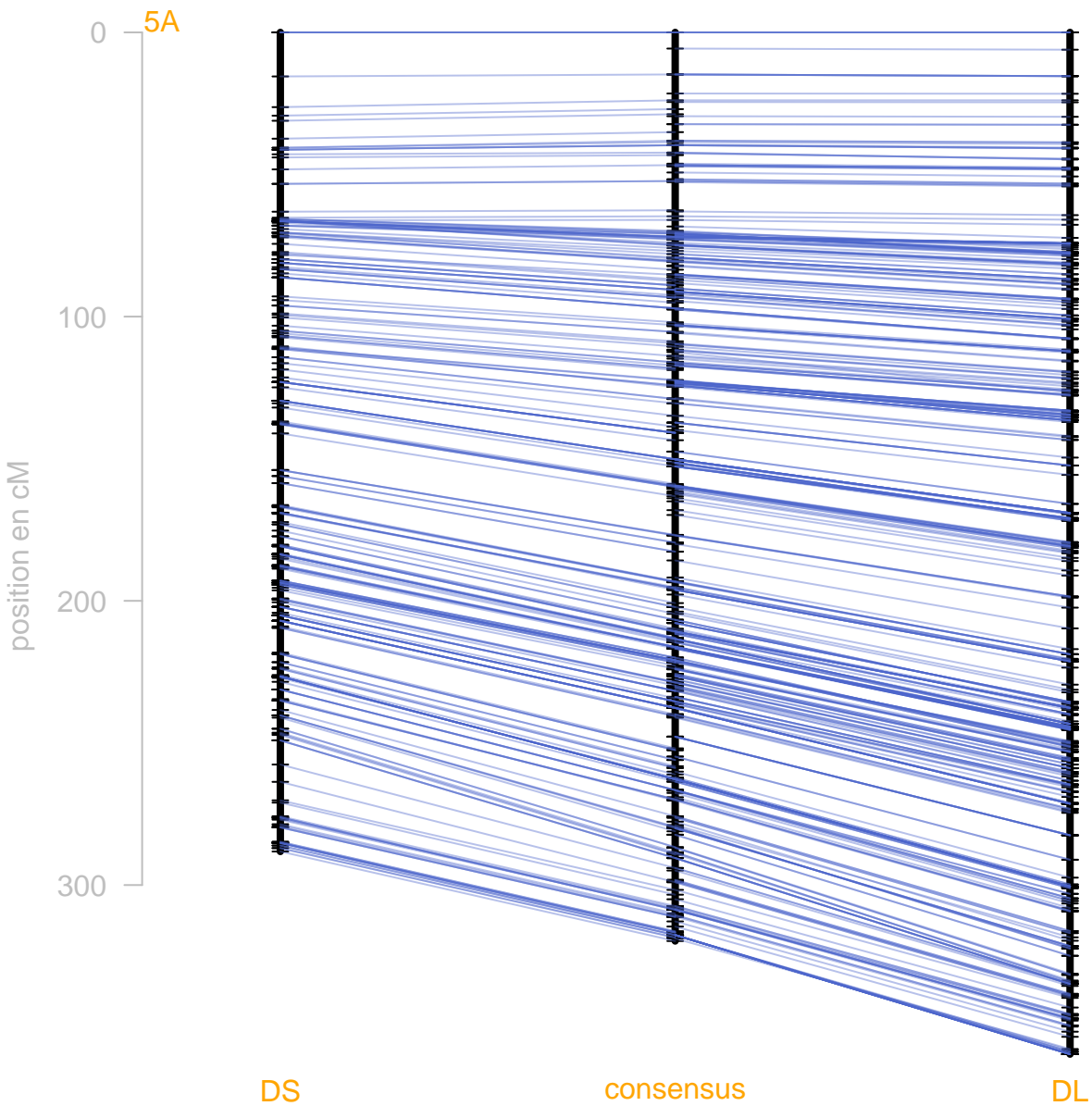

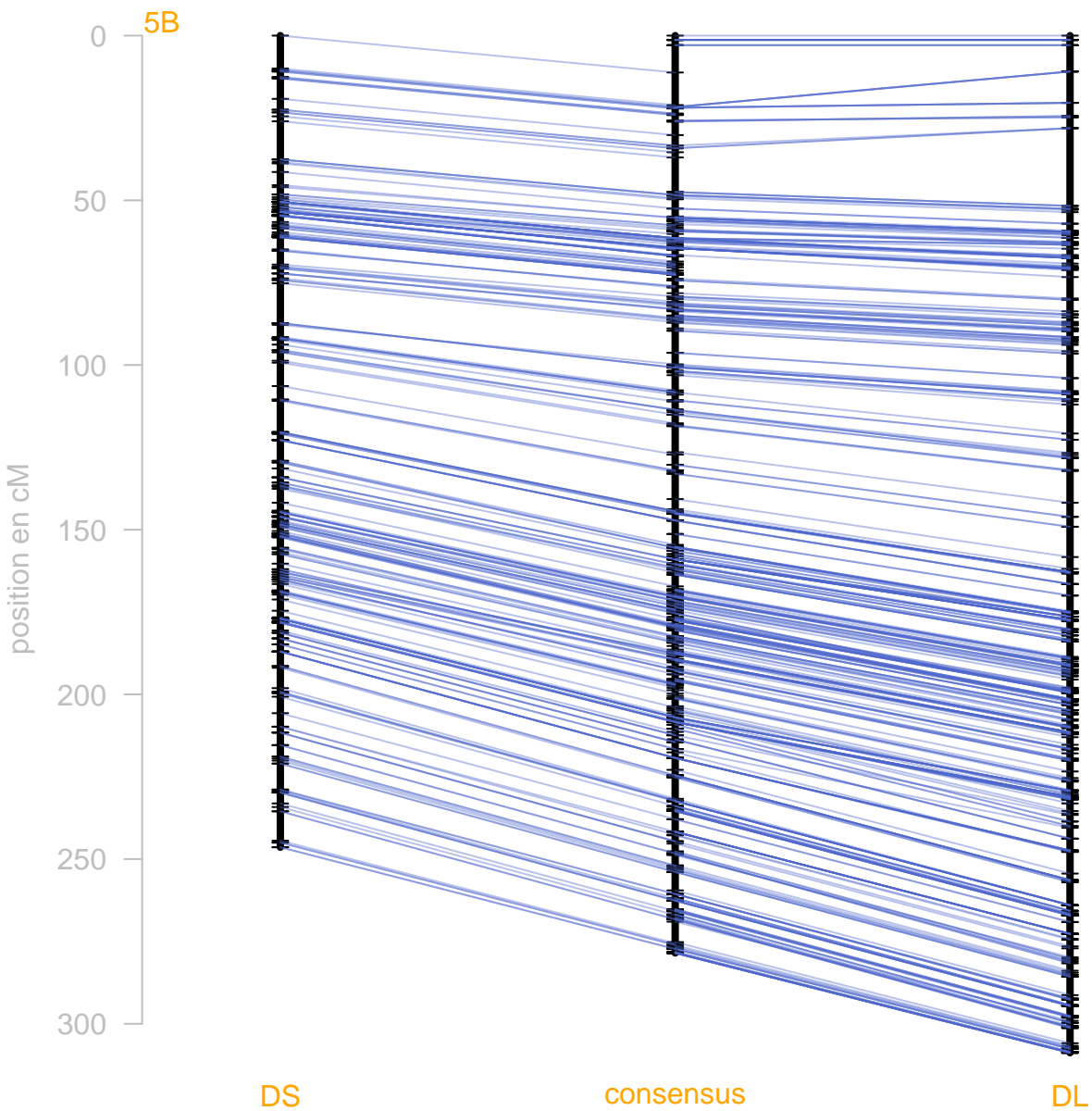

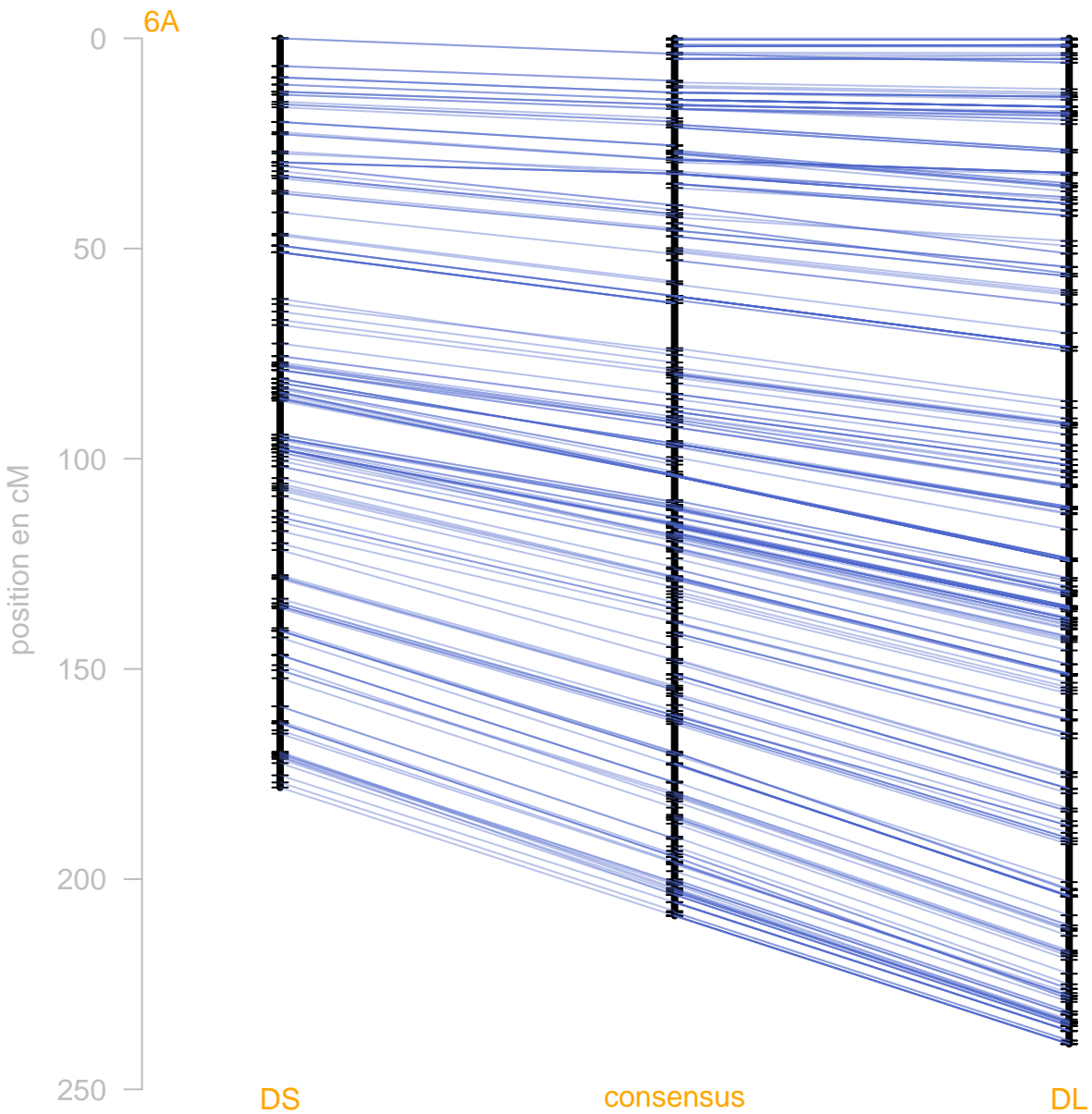

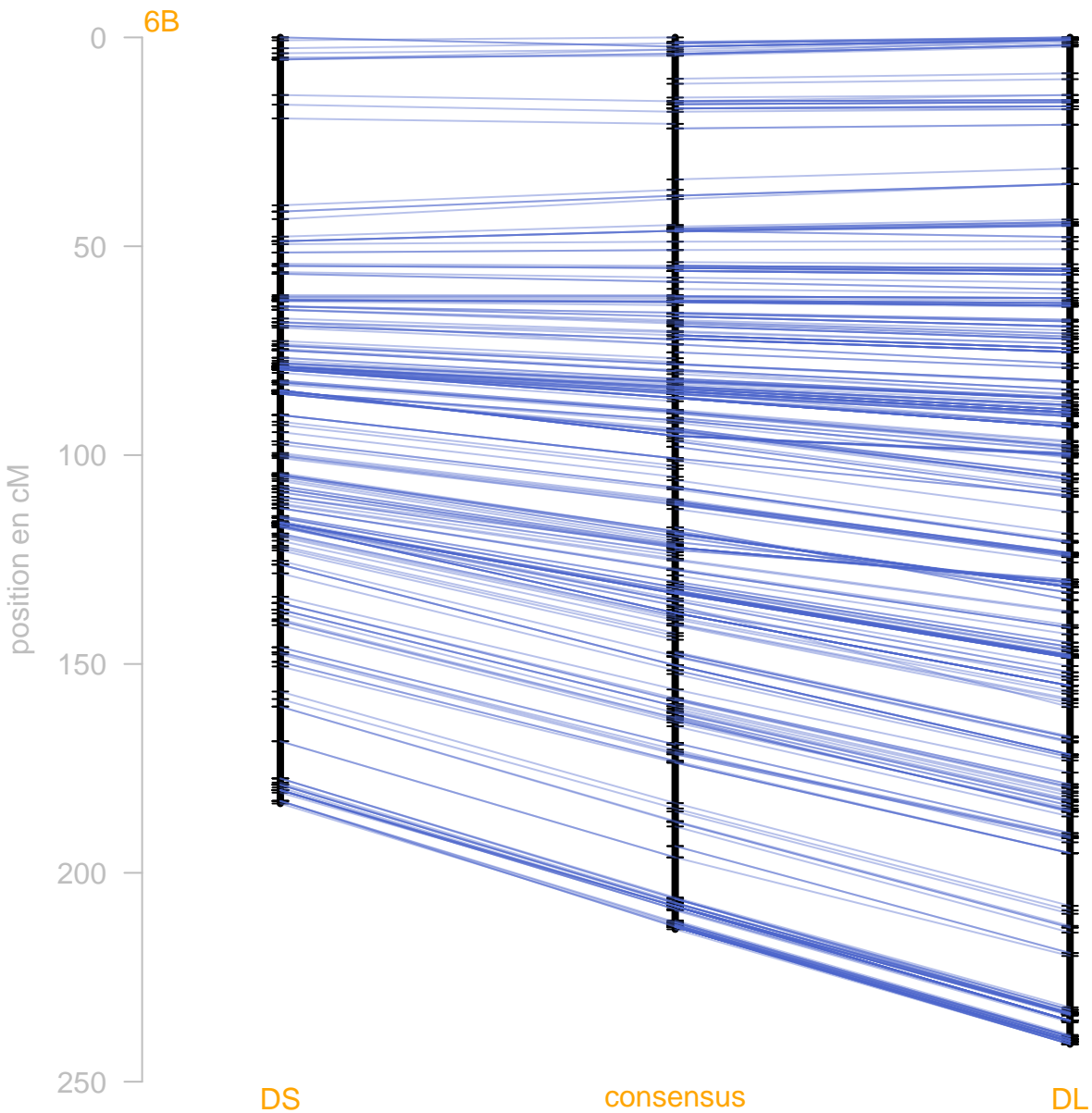

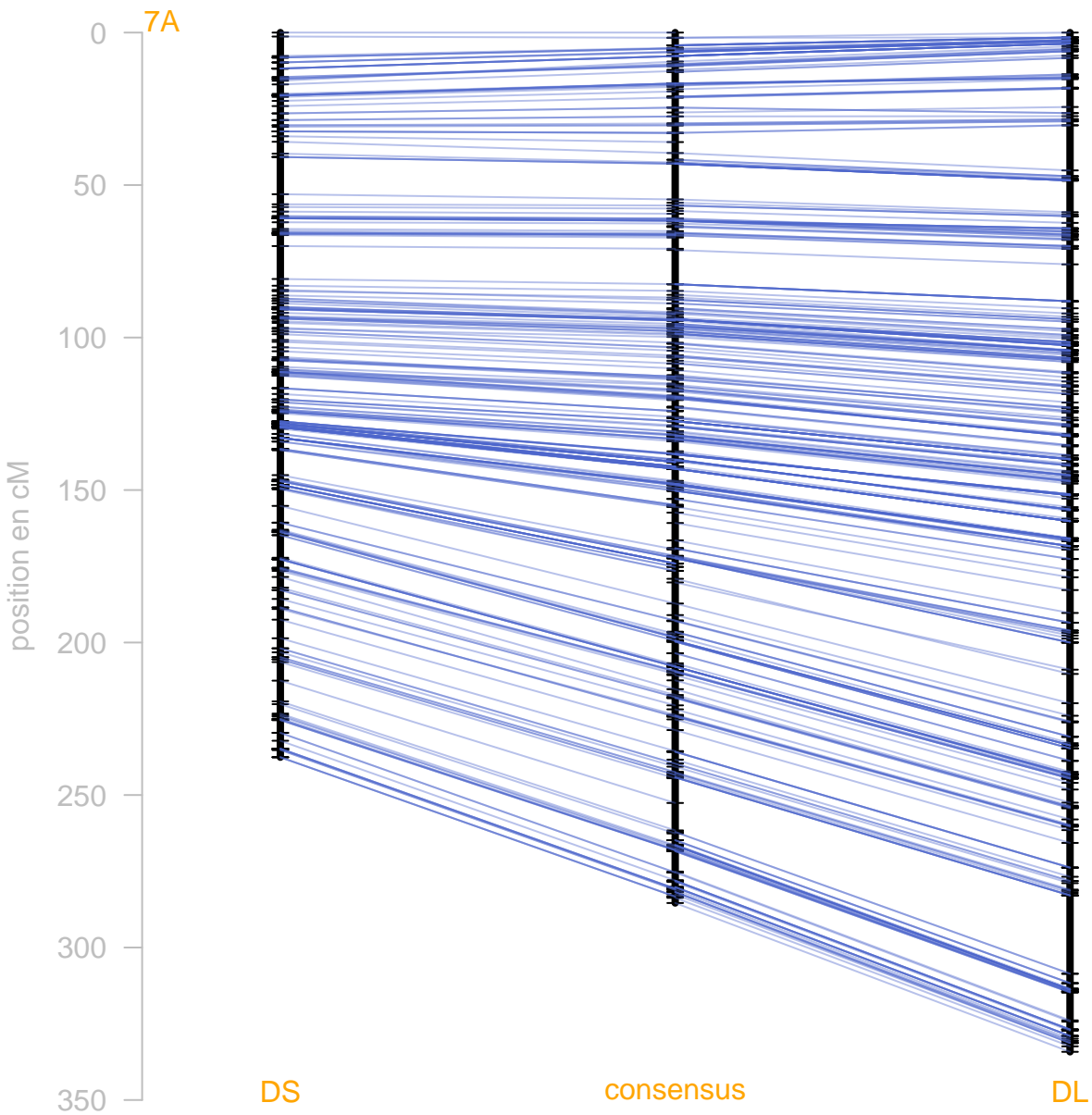

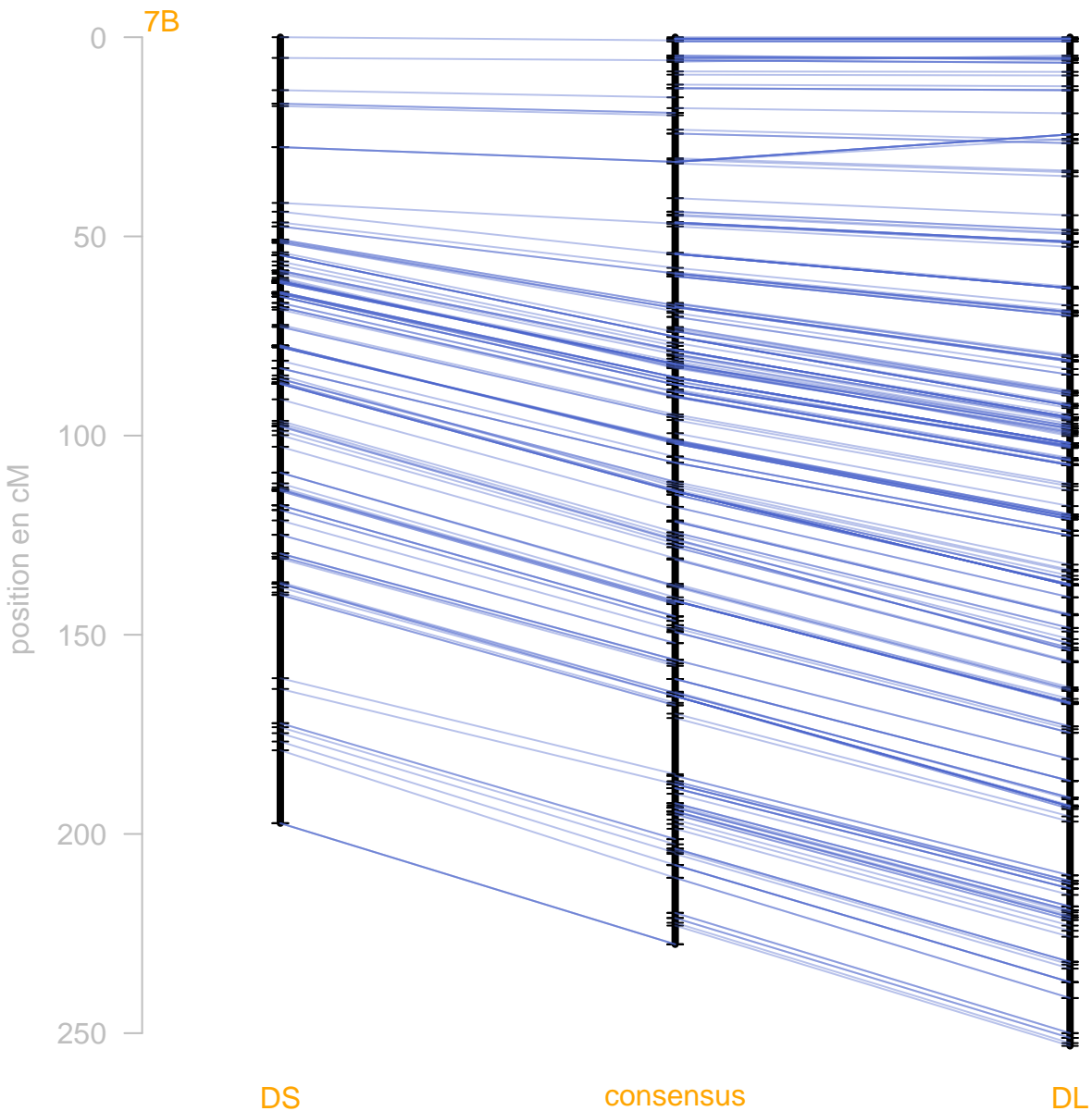

Supplement: Supplementary file 6 — Online Resource 6: Visualization of the genetic maps. For each chromosome, three parallel black lines represent the three genetic maps (DL, consensus and DS) with lengths represented in cM. The consensus map is represented in the middle, with the DS map on its left and the DL map on its right. Each marker is represented by a black point, indicating its position along the chromosome. Blue lines link common markers between two adjacent maps (PDF 139 kb) [file 122_2017_2904_MOESM6_ESM.pdf]

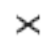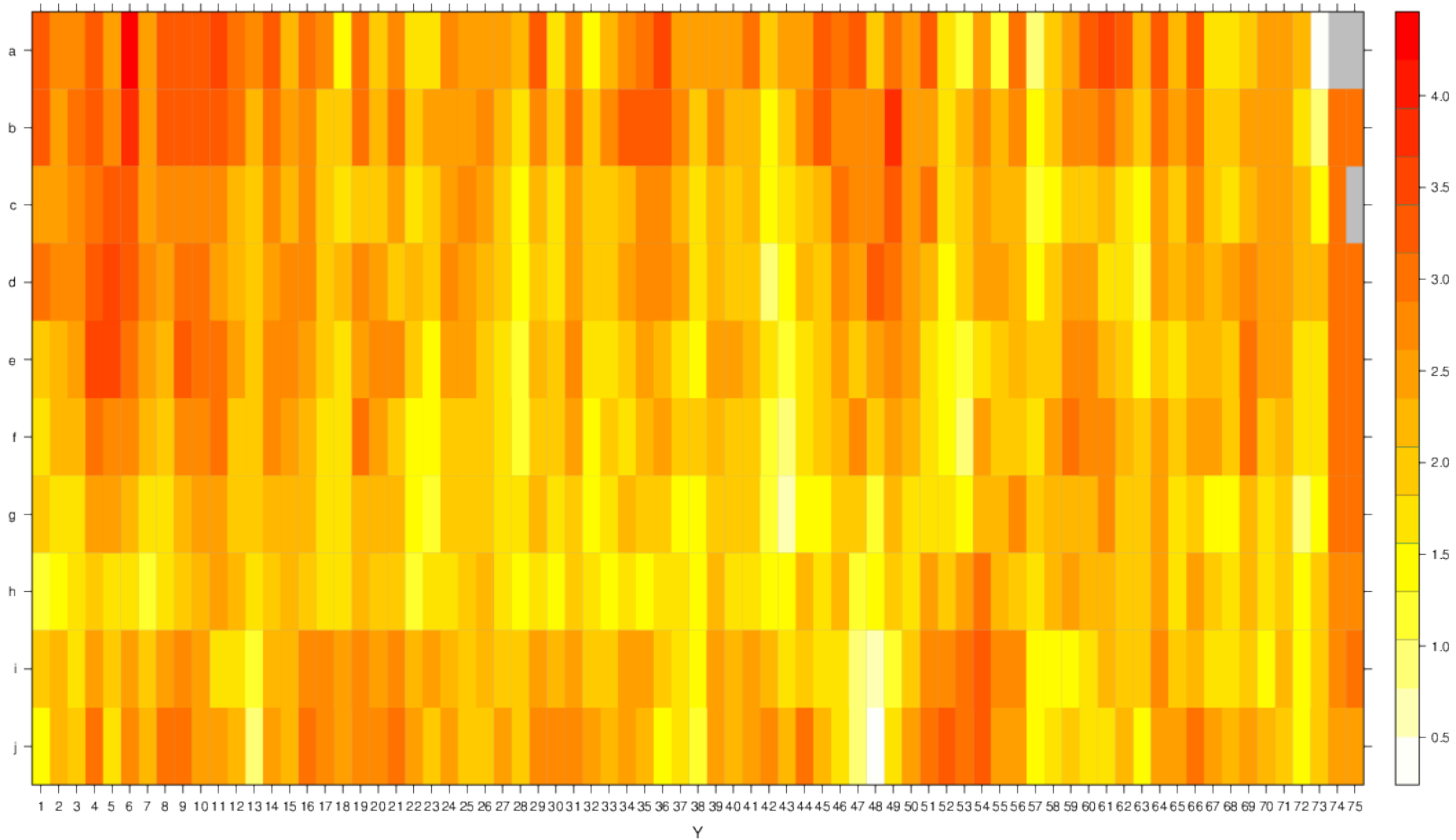

X

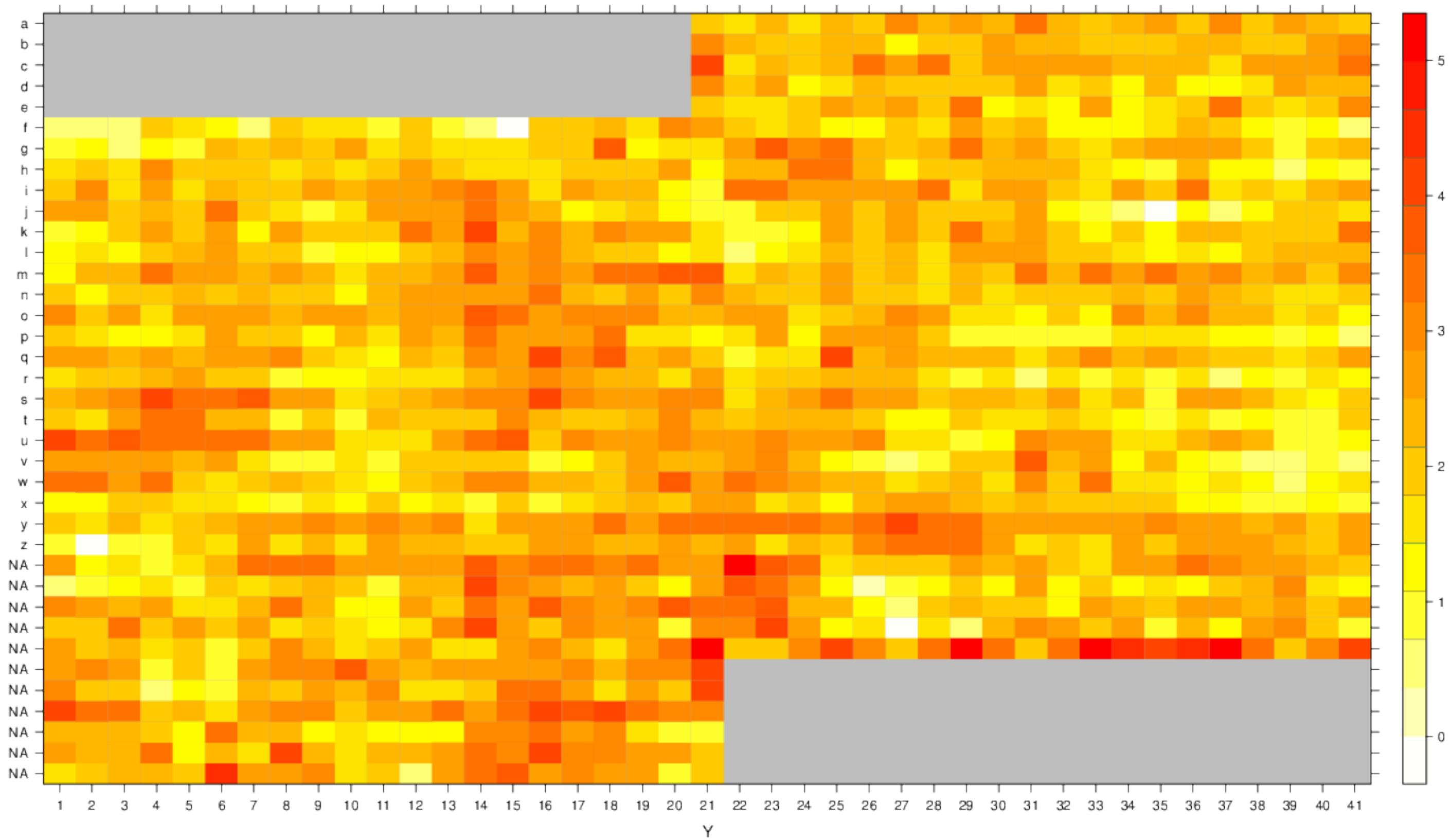

Supplement: Supplementary file 8 — Online Resource 8: Observation of the spatial heterogeneity of WSSMV infection. Experiments of 2012 and 2015 are represented in two distinct sheets. Each cell represents an accession. The cell color reflects the mean symptom severity observed in the direct neighborhood of the corresponding accession (including itself). Red indicates a strong infection (SS = 5) and white indicates no infection (SS = 0) (PDF 3167 kb) [file 122_2017_2904_MOESM8_ESM.pdf]

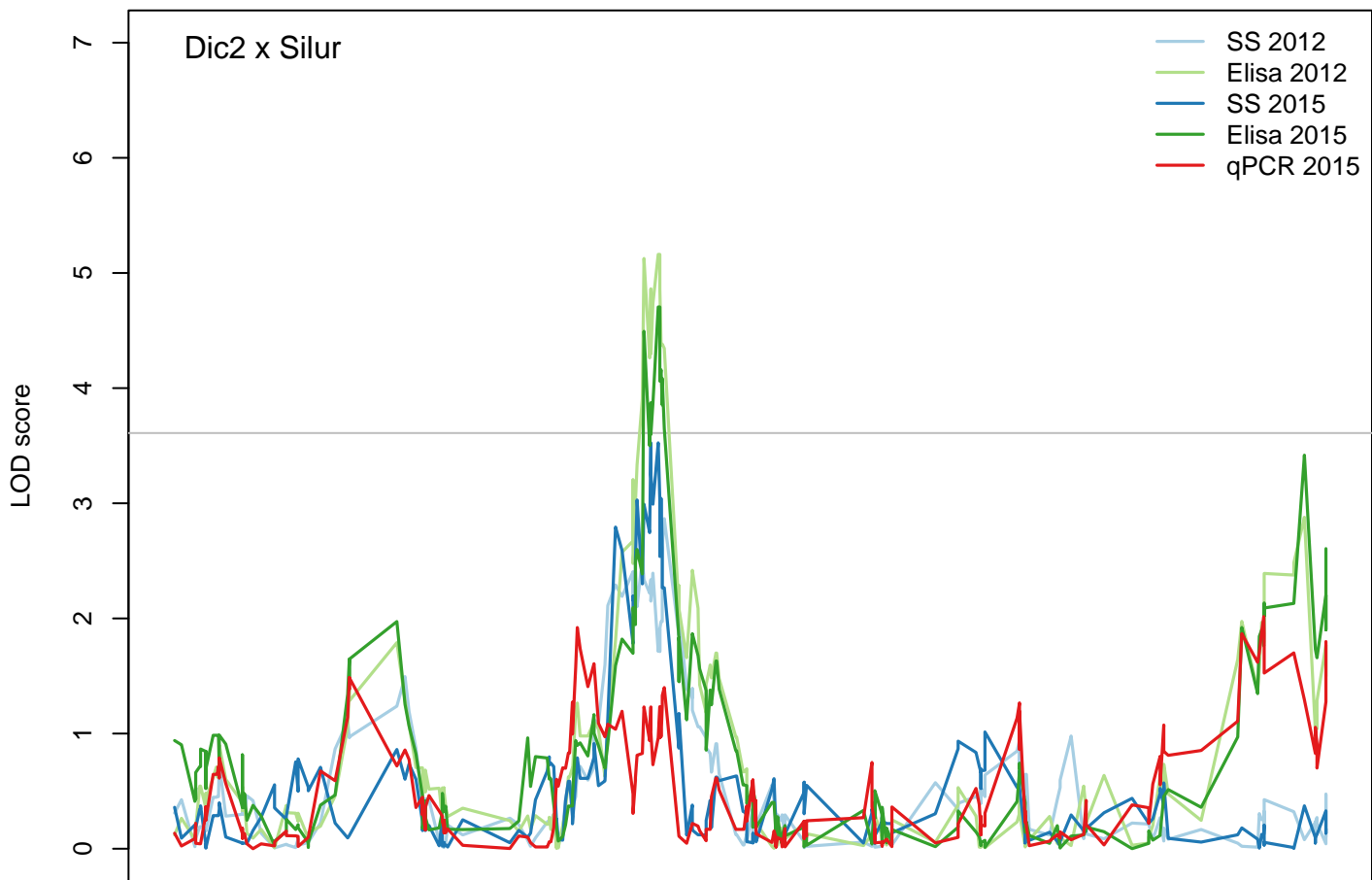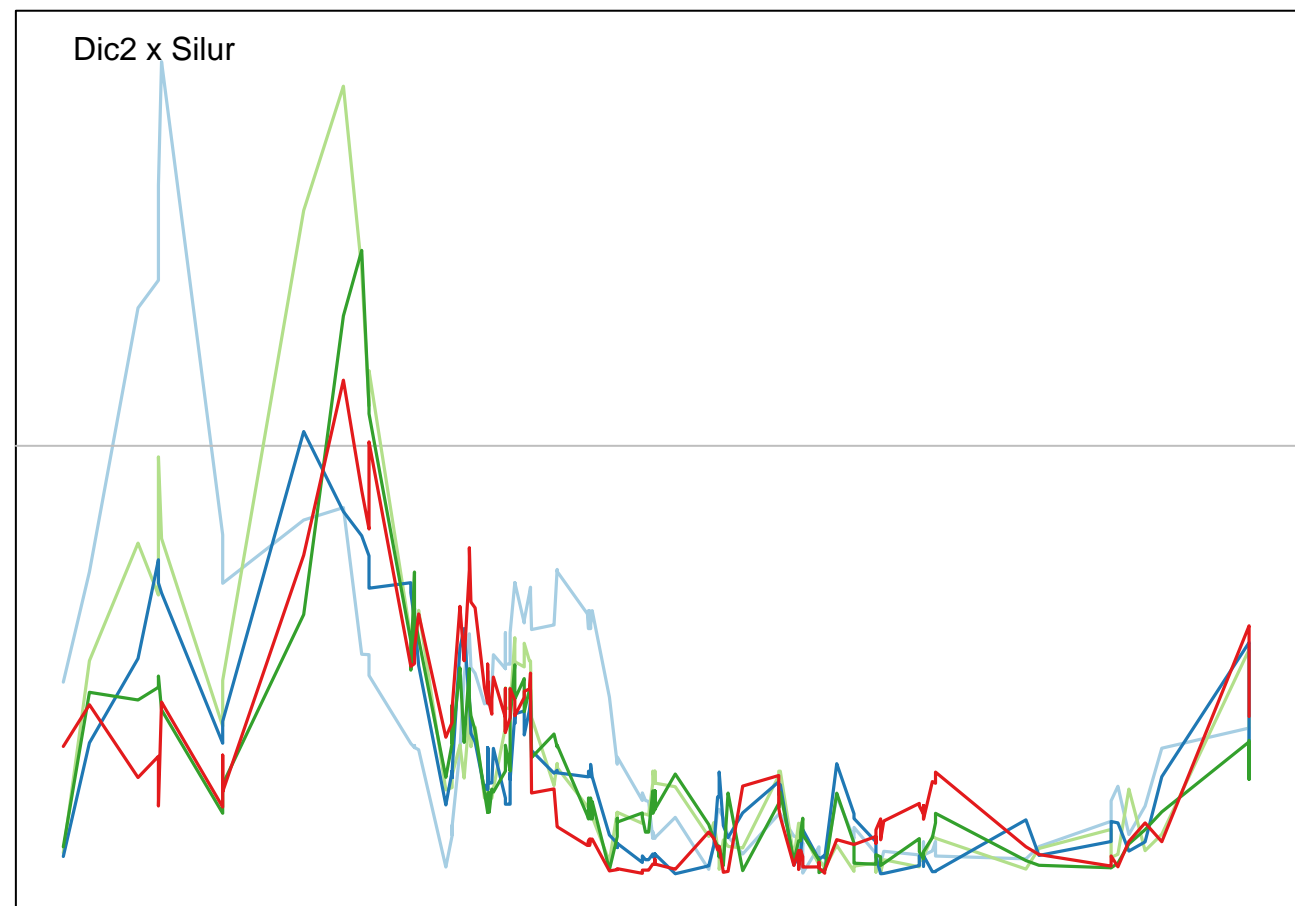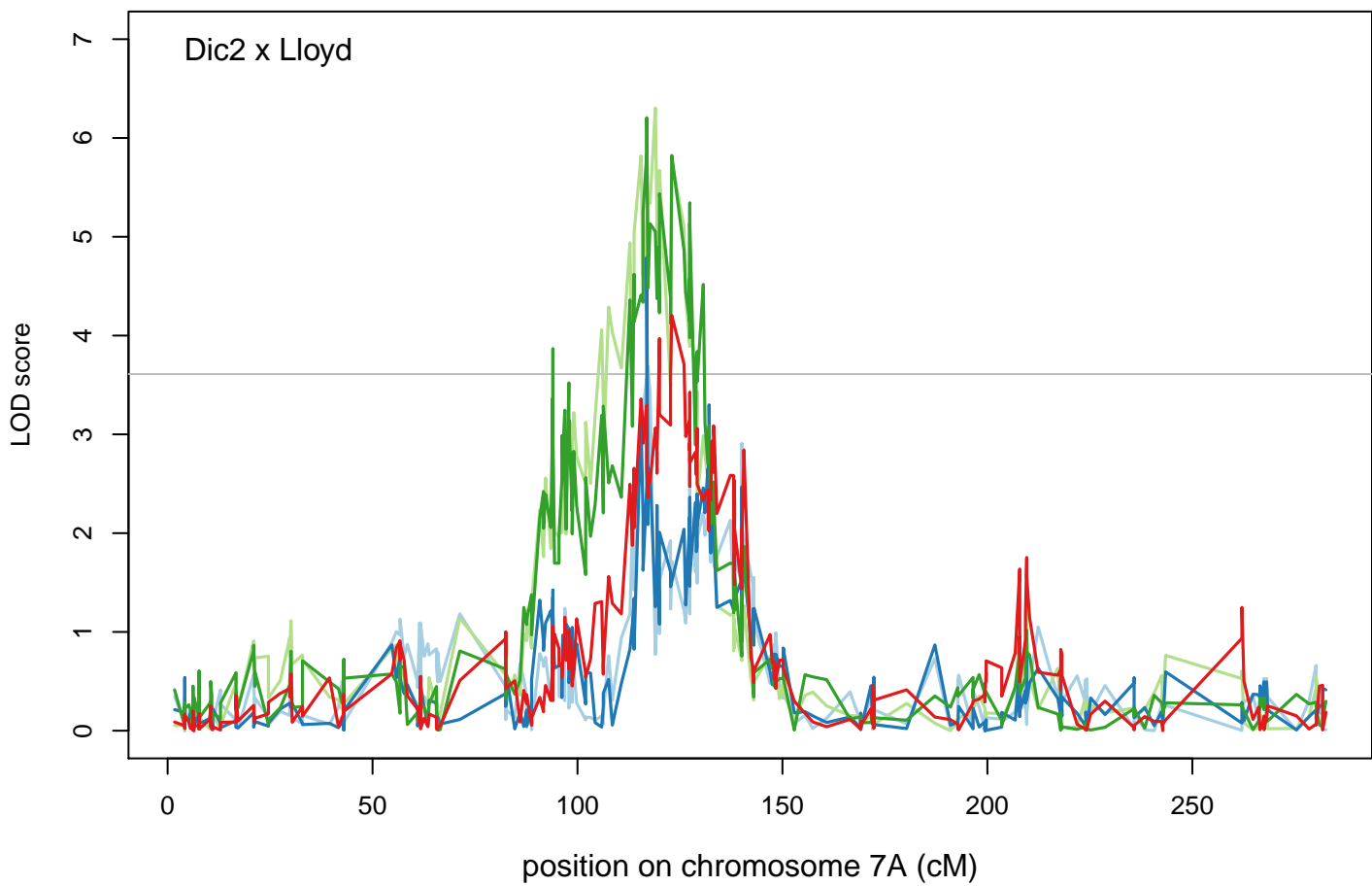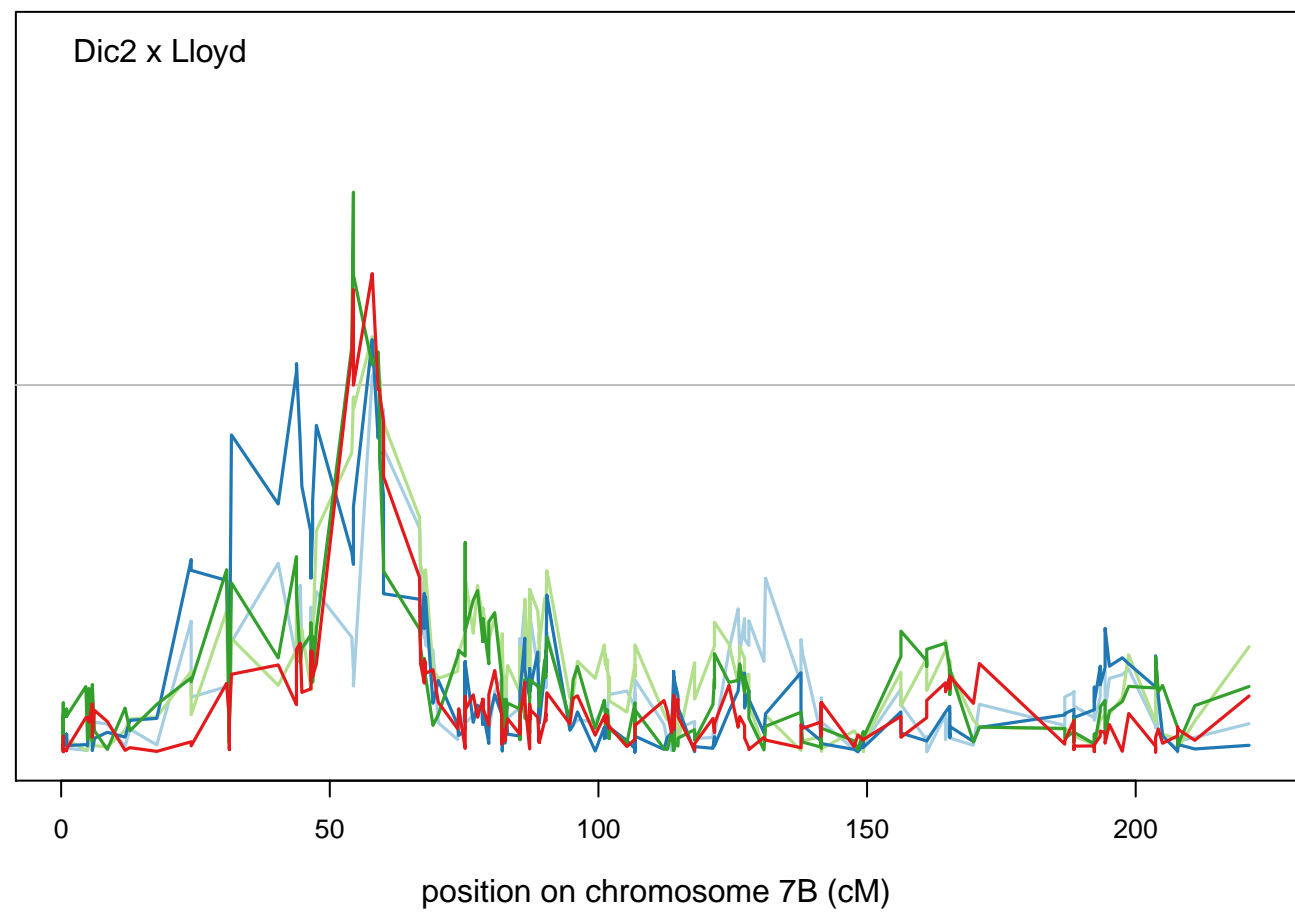

Supplement: Supplementary file 10 — Online Resource 10: Visualization of QTLs along chromosome 7A and 7B for DS and DL. Four graphics are provided that depict LOD scores observed for DS (two graphics on top) or DL (two graphics on bottom) along chromosome 7A (left graphics) or 7B (right). In each graphic, LOD scores for association with WSSMV resistance detected by simple interval mapping with QTL Rel are represented for every marker. The LOD scores of each phenotypic variable (SS, ELISA and qPCR in 2012 and 2015) are represented by a specific color (PDF 26 kb) [file 122_2017_2904_MOESM10_ESM.pdf]
